# Author Correction: Organocatalytic enantioselective [2*π* + 2*σ*] cycloaddition reactions of bicyclo[1.1.0]butanes with *α*,*β*-unsaturated aldehydes

**DOI:** 10.1038/s41467-026-69148-y

**Published:** 2026-03-16

**Authors:** Yi-Xiang Geng, Teng-Fei Xiao, Dong Xie, Ming-Ming Li, Pan-Pan Zhou, Guo-Qiang Xu, Peng-Fei Xu

**Affiliations:** State Key Laboratory of Nature Product Chemistry, College of Chemistry and Chemical Engineering, Lanzhou University, Lanzhou, PR China

**Keywords:** Synthetic chemistry methodology, Asymmetric catalysis, Asymmetric synthesis

Correction to: *Nature Communications* 10.1038/s41467-025-64399-7, published online 23 October 2025

This article was submitted to the editorial office on May 15, 2025. During the peer-review of this work, a related study was submitted and published in *Chemical Science* (*Chem*. *Sci*. **16**, 16567–16572 (2025), 10.1039/D5SC05477J) on August 12, 2025. These works were conducted independently.

