# Supplementary material for: Organocatalytic enantioselective [2π + 2σ] cycloaddition reactions of bicyclo[1.1.0]butanes with α,β-unsaturated aldehydes
Source: Nat Commun. 2025 Oct 23;16:9361. doi: 10.1038/s41467-025-64399-7 (PMC12549830; doi:10.1038/s41467-025-64399-7)
Supplement: Supplementary file 1 — Supplementary Information [file 41467_2025_64399_MOESM1_ESM.pdf]

Supplementary information for

Organocatalytic Enantioselective [2π + 2σ] Cycloaddition Reactions of Bicyclo[1.1.0]butanes with α,β-Unsaturated Aldehydes

Yi-Xiang Geng[1], Teng-Fei Xiao[1], Dong Xie[1], Ming-Ming Li[1], Pan-Pan Zhou[1]
Guo-Qiang Xu*[1], and Peng-Fei Xu*[1]

[1] State Key Laboratory of Nature Product Chemistry, College of Chemistry and Chemical Engineering, Lanzhou University, Lanzhou 730000 (P.R. China), E-mail: xupf@lzu.edu.cn, gqxu@lzu.edu.cn.

Content

1. General Information 3

2. Supplementary Figure and Table for Experiments[1–4] 4

3. Experimental Procedure and Analytical Data of Products 5

3.1 Synthesis of Aldehydes[5] 5

3.2 Synthesis of BCBs[6–8] 6

3.3 Enantioselective [$2\pi + 2\sigma$] Cycloaddition of BCB with *α,β*-Unsaturated Aldehydes ... 15

3.4 Unsuccessful substrates 37

4. Mechanistic Investigations 38

4.1 Mechanistic Study 38

4.2 DFT calculations 39

4.3 Plausible Mechanism 39

5. Synthetic Application 41

5.1 Scale-up Synthesis 41

5.2 Derivatization of the Cycloadducts 41

5.3 Synthesis of Bio-relevant BCHs 49

6. X-ray Crystallographic Data 63

7. NMR Spectra of Compound 65

8. HPLC Spectra of Compound 121

9. References 160

1. General Information

All reactions and manipulations were performed using standard Schlenk techniques. Liquids and solutions were transferred with syringes. Bicyclo[1.1.0]butanes (BCBs) and aldehydes were prepared according to reported procedures. Chemicals and solvents were either purchased from commercial suppliers or purified by standard techniques. Thin-layer chromatography (TLC) plates were visualized by exposure to ultraviolet light and/or staining with phosphomolybdic acid followed by heating on a hot plate. Flash chromatography was carried out using silica gel (200-300 mesh). NMR spectra were recorded with a Bruker AM-600 spectrometer at 600 MHz (^{1}H NMR), 151 MHz (^{13}C NMR), 376 MHz (^{19}F NMR). Chemical shifts (δ values) were reported in ppm down field from internal Me_4Si. The residual solvent signals were used as references and the chemical shifts were converted to the TMS scale ($CDCl_3$: δ_H = 7.26 ppm, δ_C = 77.00 ppm). Data for ^{1}H NMR are reported as follows: chemical shift (δ ppm), multiplicity (s = singlet, d = doublet, t = triplet, m = multiplet, dd = double doublet, dt = doublet of triplet), integration, coupling constant (Hz) and assignment. Data for ^{13}C NMR are reported as chemical shift. HRMS were performed on a Bruker Apex II mass instrument (ESI). Enantiomeric excess values were determined by HPLC with a Daicel Chirapak IA/IC-3/IF-3 column on Agilent 1260 series with i-PrOH and n-hexane. Optical rotation was measured on the Perkin Elmer 341 polarimeter with $[\alpha]_D$ values reported in degrees. The crystal measurement is performed on XtaLAB Synergy-DW.

2. Supplementary Figure and Table for Experiments[1–4]

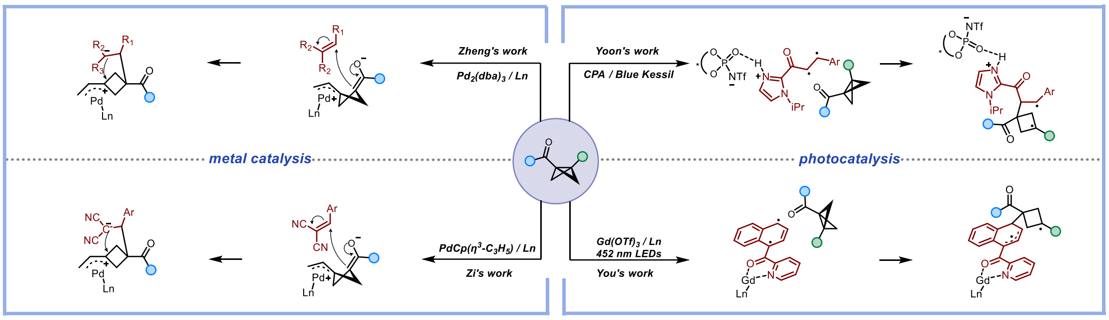

Figure S1. Recent works on enantioselective [2π + 2σ] cycloaddition reaction

Table S1. Reaction condition optimization with BCB **1a** and aldehyde **2a**: screening of different hydrogen bond catalysts[a]

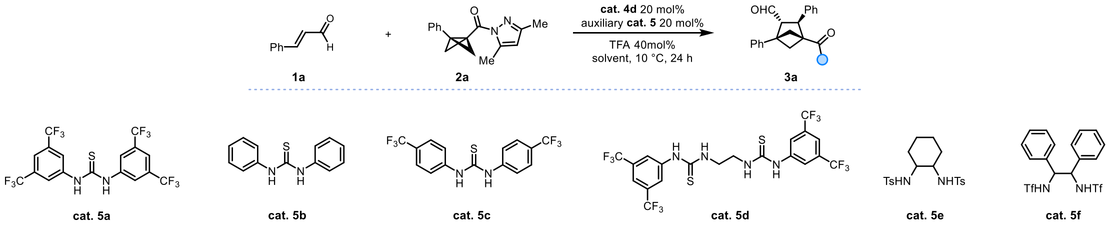

Entry	Catalyst **5**	Yield[b]	ee[c]
1	**5a**	82	99
2	**5b**	32	99
3	**5c**	67	99
4	**5d**	78	99
5	**5e**	73	99
6	**5f**	71	99

[a]Reaction conditions: Reactions performed with **1a** (0.1 mmol), **2a** (0.15 mmol), catalyst **4d** (20 mol%), catalyst **5** (20 mol%), TFA (40 mol%) in solvent (2 mL) at 10 °C for 24 h. [b]Yield of isolated product. [c]Determined through high-performance liquid chromatography (HPLC) analysis.

3. Experimental Procedure and Analytical Data of Products

3.1 Synthesis of Aldehydes[5]

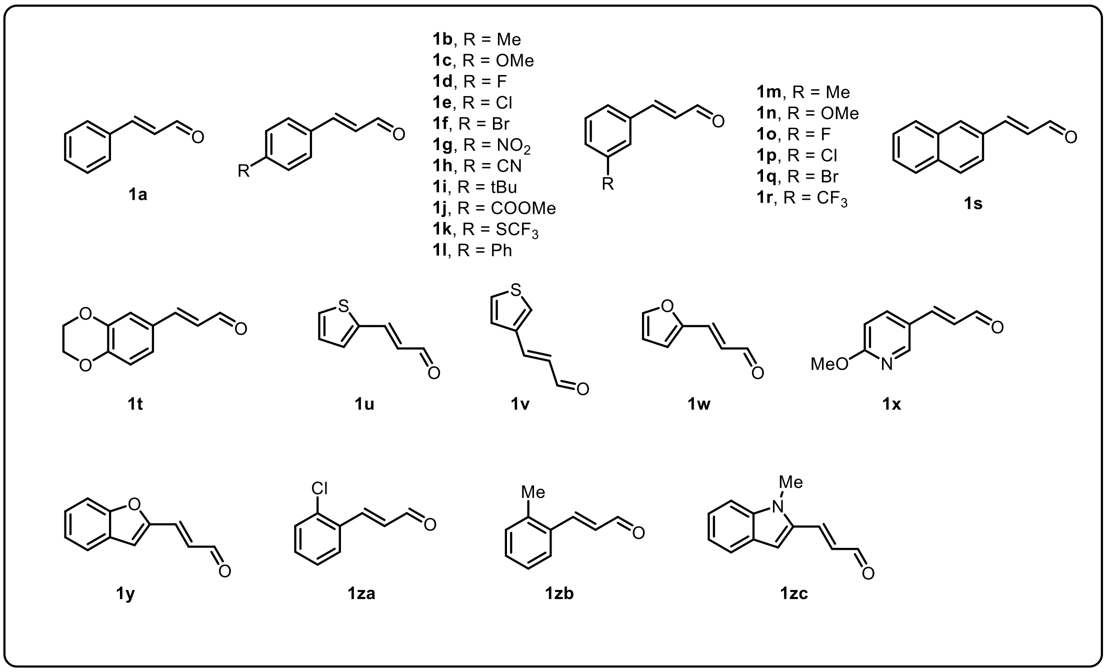

Figure S2. Overview of aldehyde substrates.

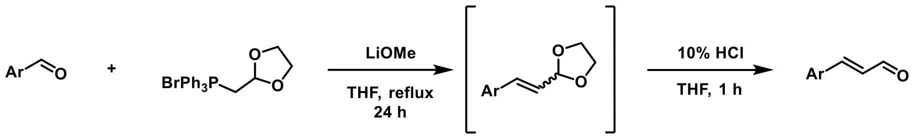

General Procedure for Wittig Homologation of Aldehydes: Lithium methoxide (0.494 g, 13 mmol, 2.6 eq.) was added to a stirring solution of (1,3 dioxolan-2-yl)methyl-triphenylphosphonium bromide (5.47 g, 12.5 mmol, 2.5 eq.) in 50 mL of anhydrous tetrahydrofuran (THF) in a 250 mL round bottom flask. The suspension was heated to reflux stirred for 30 minutes, changing color from off-white to light orange/pink. Aromatic aldehyde (5 mmol, 1.0 eq.) in 30 mL of dry THF solution was added dropwise over 30-60 min. The suspension was refluxed for 24 h. The reaction suspension was then cooled to room temperature, at which point 10%

aqueous hydrochloric acid (HCl) was added. Stirring was continued for 1 hour in order to hydrolyze the intermediate acetals (mixture of E- and Z-stereoisomers) to the all-trans configuration. The organic layer was extracted with CH_2Cl_2 (50 mL x 3) and the combined fractions were washed with water, sat. aqueous sodium bicarbonate solution and brine, and then dried over Na_2SO_4. Solvent was removed by rotary evaporation and the product was purified silica gel column chromatography (PE/EA = 19:1, v/v) over 25 column volumes to give the aldehydes as known compounds.

3.2 Synthesis of BCBs[6–8]

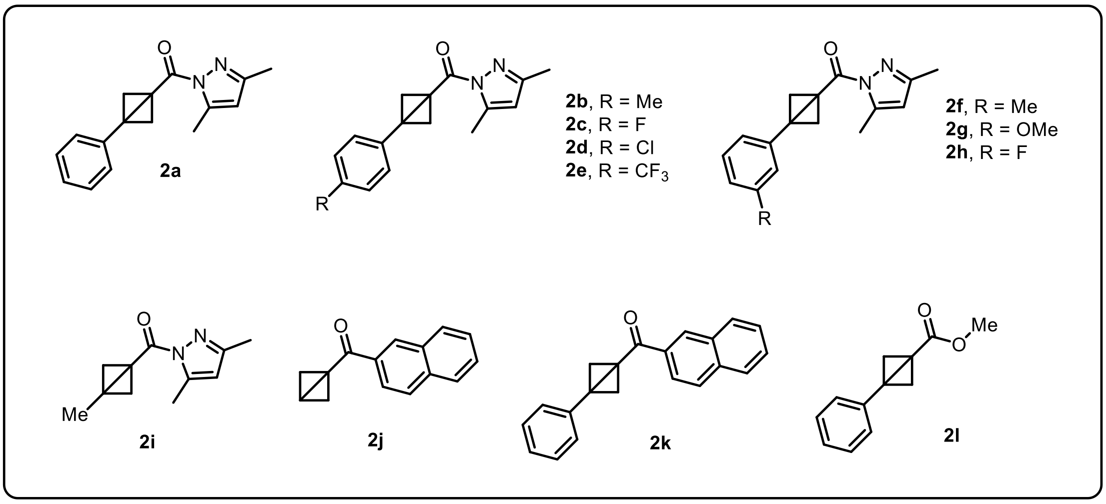

Figure S3. Overview of BCB substrates.

BCB 2a-2i from **2a to 2d, 2f** to **2h**, were prepared according to **general procedure A**, **2e** was prepared according to **general procedure B**, **2i** was prepared according to **general procedure C**, **2j** was prepared according to **general procedure D**, **2k to 2l** were prepared according to **general procedure E,** all BCBs are known compounds.

General Procedure A

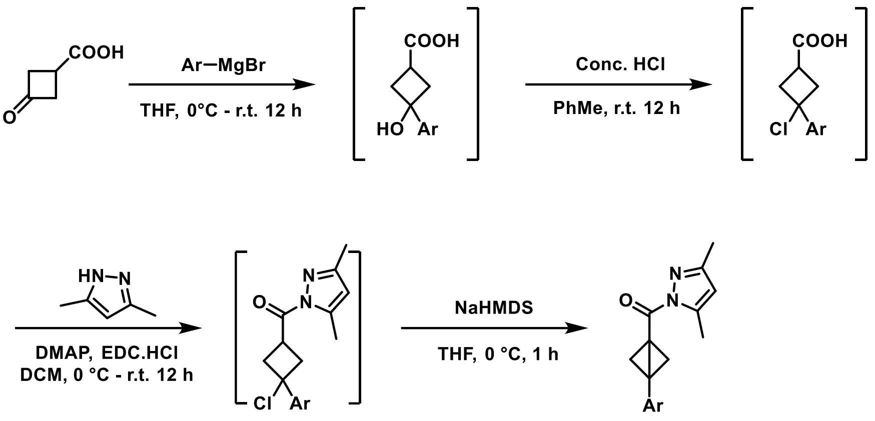

A 100 mL Schlenk flask charged with 3-oxocyclobutane-1-carboxylic acid (1.14 g, 10 mmol, 1.0 eq.) and a stir bar was evacuated and backfilled with Ar three times and dry THF (10 mL) was added. The solution was cooled to 0 °C and phenylmagnesium bromide (22 mmol, 2.2 eq.) was added dropwisely via a syringe. The reaction mixture was warmed up to room temperature and allowed to stir for 12 h. Upon completion, the reaction was quenched with sat. NH_4Cl (10 mL) and water (10 mL) and transferred to a separatory funnul. The aqueous phase was collected, washed with EA (10 mL) and acidified with conc. HCl to pH = 1. Then the aqueous phase was extracted with EA (20 mL × 5) and the combined organic phase was dried over anhydrous Na_2SO_4 and concentrated under vacuo to give hydroxyl acid intermidiate as a white solid, which was directly used in the next step. To a 250 mL round-bottom flask containing the above solid and a stir bar was added toluene (50 mL) and conc. HCl (10 mL). The two-phased liquid was stirred vigorously at room temperature for 12 h, until which the organic layer was collected and the aqueous layer was extrated with toluene (10 mL). The combined organic phase was dried over anhydrous Na_2SO_4 and concentrated under vacuo to give the chlorinated acid as a white solid (1.43 g, 6.8 mmol). To a 250 mL round-bottom flask was added the above solid, 3,5-dimethyl-1H-pyrazole (7.5 mmol, 0.72 g, 1.1 eq.), DMAP (6.8 mmol, 0.83 g, 1 eq.), DCM (20 mL) and a stir bar. The solution was cooled to 0 °C and EDC hydrochloride (8.2 mmol, 1.57 g, 1.2 eq.) was added portionwisely. The resulted mixture was warmed up to room temperature, stirred for 12 h, and transferred to a separatory funnel. 1 M HCl (10 mL) and brine (20 mL) was added to wash the

solution, which was then dried over anhydrous Na_2SO_4 and concentrated under vacuo to give the crude pyrazol amide without further purification. A 50 mL Schlenk flask was added the above compound and a stir bar. THF (20 mL) was added and the solution was cooled down to 0 °C. NaHMDS (2 M, 3.4 mL) was added dropwisely via a syringe. The reaction mixture was stirred at this temperature for 30 min. sat. NH_4Cl (10 mL) and water (10 mL) was added to quench the reaction. The mixture was transferred to a separatory funnel and the organic layer was collected. The aqueous was extracted with EA (20 mL × 3) and the combine organic phase was dried over anhydrous Na_2SO_4, concentrated by rotary evaporation (water bath temperature < 45 °C) and purified by silica gel column chromatography (PE/EA = 30:1, v/v) to afford **BCB 2a**.

General Procedure B

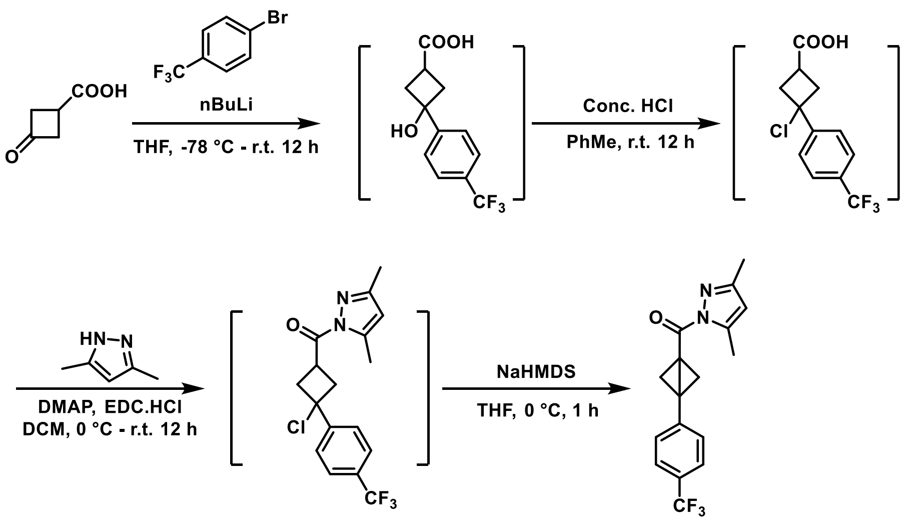

A 100 mL Schlenk tube containing 1-bromo-4-(trifluoromethyl)benzene (22 mmol, 4.95 g) and THF (20 mL) was cooled to -78 °C, n-BuLi (1.6 M, 1.5 eq., 20.6 mL) was added dropwise to above solution under argon and stirred at this temperature for 2 h. This freshly made aryllithium reagent was added dropwise to a 100 mL round-bottomed flask containing 3-oxocyclobutane-1-carboxylic acid (1.14 g, 10 mmol, 1 eq.) and THF (10 mL) at 0 °C under argon. Upon completion of addition, the cooling bath was removed. The reaction mixture was gradually warmed up to room

temperature and stirred overnight. Quenched with sat. NH_4Cl (10 mL) and water (10 mL). The product mixture was transferred to a separatory funnel with EA (10 mL), and the organic phase was removed. The aqueous phase was acidified with concentrated HCl to PH = 1. Extracted the aqueous phase with EA (20 mL x 5), and after evaporation of the solvent afforded a crude solid. The above solid was dissolved with toluene (50 mL) and concentrated HCl (10 mL) and stirred vigorously at room temperature overnight. The toluene phase was collected and the aqueous phase was extracted with toluene (10 mL) once. The combined toluene phase was dried over anhydrous $MgSO_4$, filtered and concentrated to dryness by rotary evaporation to afford a white solid (0.83 g, ~3.0 mmol), which was used directly in next step. To a 100 mL round-bottomed flask the above solid was added followed by 3,5-dimethyl-1H-pyrazole (3.3 mmol, 317 mg, 1.1 eq.), DMAP (3.0 mmol, 366 mg, 1 eq.), and DCM (20 mL), and the solution was cooled to 0 °C. EDC hydrochloride (3.6 mmol, 688 mg, 1.2 eq.) was added portionwisely. The resulted mixture was warmed up to room temperature, stirred for 12 h, and transferred to a separatory funnel. 1 M HCl (10 mL) and brine (20 mL) was added to wash the solution, which was then dried over anhydrous Na_2SO_4 and concentrated under vacuo to give the crude pyrazol amide without further purification. A 50 mL Schlenk flask was added the above compound and a stir bar. THF (20 mL) was added and the solution was cooled down to 0 °C. NaHMDS (2 M, 1.5 mL) was added dropwisely via a syringe. The reaction mixture was stirred at this temperature for 30 min. sat. NH_4Cl (10 mL) and water (10 mL) was added to quench the reaction. The mixture was transferred to a separatory funnel and the organic layer was collected. The aqueous was extracted with EA (20 mL × 3) and the combine organic phase was dried over anhydrous Na_2SO_4, concentrated by rotary evaporation (water bath temperature < 45 °C) and purified by silica gel column chromatography (PE/EA = 30:1, v/v) to afford **BCB 2e**.

General Procedure C

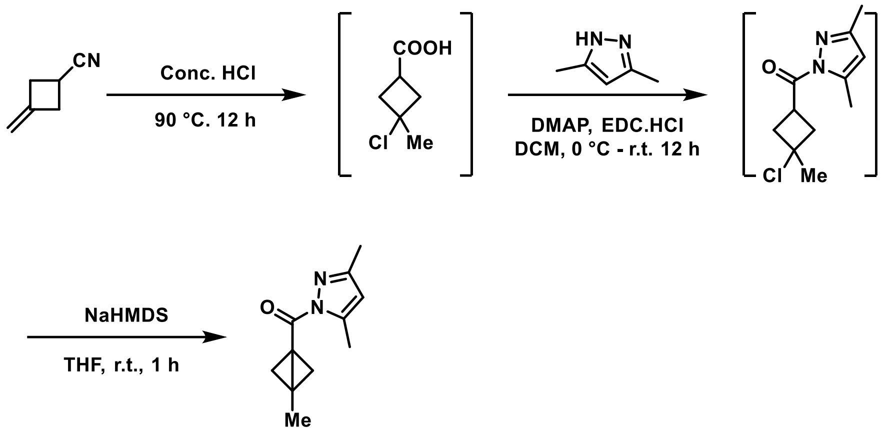

A 100 mL round bottom flask equipped with a magnetic stir bar was charged with 3-methylenecyclobutanecarbonitrile (1.86 g, 20.0 mmol, 1 eq.) and concentrated HCl solution (20 mL). The flask was equipped with a reflux condenser and heated to 90 °C for 16 h. After cooled back to room temperature, the solution was diluted with H_2O (50 mL). The aqueous layer was extracted with Et_2O (50 mL x 3). The combined organic layers were washed with brine (100 mL), dried over anhydrous $MgSO_4$, filtered and concentrated by rotary evaporation to afford a colorless oil (2.3 g, ~15.5 mmol), which was used directly in next step. To a 100 mL round-bottomed flask containing the above colorless oil was added 3,5-dimethyl-1H-pyrazole (17.05 mmol, 1.64 g, 1.1 eq.), DMAP (15.5 mmol, 1.89, 1 eq.), and DCM (20 mL), and the solution was cooled to 0 °C. EDC hydrochloride (18.6 mmol, 3.55 g, 1.2 eq.) was added and the solution was stirred at room temperature overnight. The product mixture was transferred to a separatory funnel with DCM (10 mL), and then washed with 1N HCl solution (10 mL x 2) and brine (20 mL x 2). The organic phase was dried over anhydrous $MgSO_4$, filtered and concentrated to dryness by rotary evaporation to afford an oily mixture which was used directly in next step. The above mixture (assuming 15.5 mmol) was dissolved with THF (10 mL) and NaHMDS (2 M, 7.8 mL) was added dropwise to this solution at room temperature. After stirring at room temperature for 1 h, quenched with sat. NH_4Cl (10 mL) and water (10 mL), and extracted with EA (20 mL x 3). The combined organic layers were dried over anhydrous Na_2SO_4, filtered and concentrated by rotary evaporation (water bath

temperature < 40 °C). The mixture was purified by silica gel column chromatography (PE/EA = 30:1, v/v) to afford **BCB 2i**.

General Procedure D

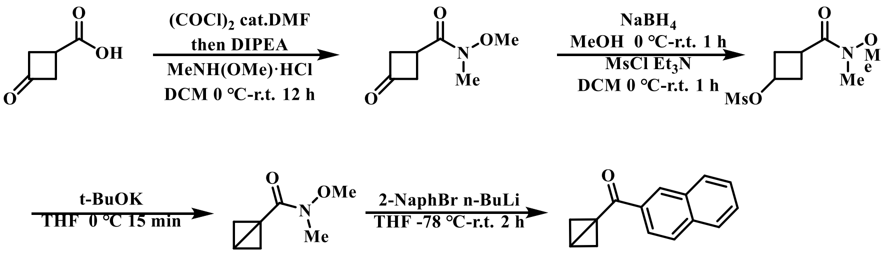

A 100 mL round bottom flask equipped with a magnetic stir bar was charged with 3-oxocyclobutanecarboxylic acid 6 (1.14 g, 10 mmol, 1.0 eq.) and DCM (40 mL). DMF (5 drops) and oxalyl chloride (0.94 mL, 11 mmol, 1.1 eq.) were slowly added to the solution over 5 min at 0 °C in an ice/water bath. The flask was capped with a septum. The reaction was naturally warmed to rt and stirred for 12 h. Then the reaction was cooled to 0 °C again in an ice/water bath. MeNH(OMe)·HCl (1.1 g, 11 mmol, 1.0 eq.) and DIPEA (6.1 mL, 35 mmol, 3.5 eq.) were slowly added over 5 min. The reaction was naturally warmed to room temperature again and stirred for 12 h before quenched with H_2O (200 mL). The aqueous layer was extracted with DCM (10 mL x 3). The combined organic layers were washed with brine (25 mL), dried over anhydrous $MgSO_4$, filtered and concentrated by rotary evaporation. The crude amide was directly used in next reaction without further purification. A 100 mL round bottom flask equipped with a magnetic stir bar was charged with above crude ketone (assuming 10 mmol, 1.00 eq.) and MeOH (30 mL). The flask was capped with a septum. $NaBH_4$ (0. 1 g x 4, 2.5 mmol x 4, 0.25 eq. x 4) was added in 4 portions over 10 min at 0 °C in an ice/water bath. Then ice/water bath was removed and the reaction was stirred at room temperature for 1 h before quenched with H_2O (5 mL). The solution was concentrated by rotary evaporation to remove most MeOH. The residue was diluted with H_2O (20 mL) and extracted with EA (15 mL x 3). The combined organic layers were washed with brine (25 mL), dried over anhydrous Na_2SO_4,

filtered and concentrated by rotary evaporation. The crude alcohol was directly used in next reaction without further purification. A 100 mL round bottom flask charged with a magnetic stir bar was charged with above crude alcohol (assuming 10 mmol, 1.0 eq.) and DCM (30 mL). Et_3N (1.4 mL, 10 mmol, 1.0 eq.) and MsCl (0.78 mL, 10 mmol, 1.0 eq.) were added at 0 °C in an ice/water bath. The flask was capped with a septum. The reaction was naturally warmed to room temperature again and stirred for 12 h before quenched with H_2O (20 mL). The aqueous layer was extracted with DCM (10 mL x 3). The combined organic layers were washed with brine (25 mL), dried over anhydrous Na_2SO_4, filtered and concentrated by rotary evaporation. The residue was purified by silica gel column chromatography (PE/EA = 1:1, v/v) to afford methylsulfonate as a pale yellow solid. An oven-dried 100 mL round bottom flask equipped with a stir bar was cooled under vacuum. After backfilled with Ar (x 3) and capped with a septum, methylsulfonate (1.19 g, 5 mmol, 1.00 eq.) and THF (35 mL) were added. The reaction was cooled to 0 °C in an ice/water bath and t-BuOK (freshly made 1 M solution in THF, 5.5 mL, 1.05 eq.) was added in one portion. The mixture was vigorously stirred for 15 min at the same temperature and quenched with saturated NH_4Cl solution (20 mL). The aqueous layer was extracted with EA (10 mL x 3). The combined organic layers were washed with brine (30 mL), dried over anhydrous Na_2SO_4, filtered and concentrated by rotary evaporation. The crude amide was dissolved in 5 mL THF immediately after concentration and the solution was directly used in next reaction without further purification. An oven-dried 100 mL round bottom flask equipped with a stir bar was cooled under vacuum. After backfilled with Ar (x 3) and capped with a septum, 2-NaphBr (1.14 g, 5.5 mmol, 1.1 eq.) and THF (25 mL) were added. The solution was cooled to -78 °C and n-BuLi (2.5 M in Hex, 2.0 mL, 5 mmol, 1.0 eq.) was added. After stirred at the same temperature for 0.5 h, a solution of above crude amide (assuming 5 mmol, 1.0 eq.) in THF (5 mL) was added. After 0.5 h, the reaction was warmed up at room temperature stirred for 1.5 h before quenched with saturated NH_4Cl solution (20 mL). The aqueous layer was extracted with EA (10 mL x 3). The combined organic layers were washed with brine (100 mL), dried over anhydrous Na_2SO_4, filtered and concentrated by rotary

evaporation. The residue was purified by silica gel column chromatography (PE/EA = 19:1, v/v) and further purified by recrystallization in DCM/Hex to afford desired **BCB 2j**.

General Procedure E

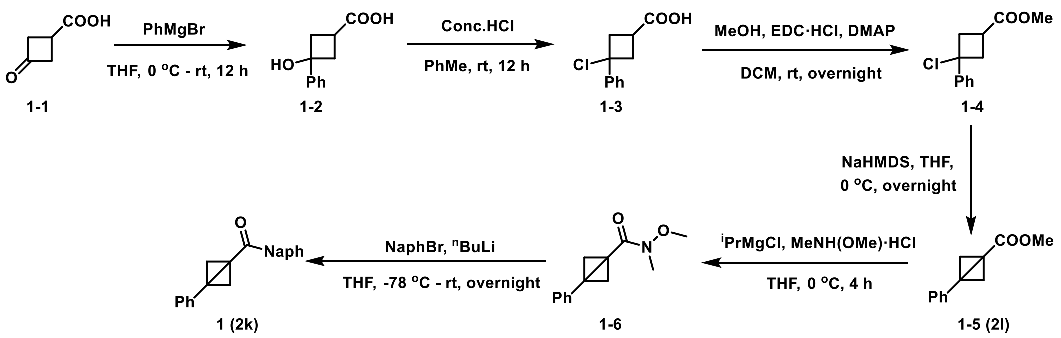

An oven-dried 100 mL round bottom flask equipped with a stir bar was cooled under vacuum. After backfilled with Ar (x 3) and capped with a septum, 3-oxocyclobutanecarboxylic acid **1-1** (1.71 g, 15.0 mmol, 1.00 eq.) and THF (30 mL) were added. The reaction was cooled to 0 °C in an ice/water bath and PhMgBr (11 mL, 3.0 M in THF, 33 mmol, 2.2 eq.) was added to the solution. The ice/water bath was then removed and the reaction was stirred for 12 h at room temperature before quenched with saturated NH_4Cl solution (30 mL). The product mixture was transferred to a separatory funnel with EA (10 mL), and the organic phase was removed. The aqueous phase was acidified with concentrated HCl to PH = 1. Extracted the aqueous phase with EA (20 mL x 5), and after evaporation of the solvent afforded a crude solid **1-2**. A 100 mL round bottom flask equipped with a magnetic stir bar was charged with above crude acid **1-2** (assuming 15 mmol, 1.0 eq.), concentrated HCl solution (15 mL) and toluene (50 mL). The flask was capped with a septum. The reaction was vigorously stirred for 12 h at room temperature. The aqueous layer was extracted with toluene (50 mL). The combined toluene phase was dried over anhydrous $MgSO_4$, filtered and concentrated to dryness by rotary evaporation to afford a white solid **1-3**, which was used directly in next step. A magnetically stirred solution of **1-3** (2.10 g, 10 mmol, 1.0 eq.) in DCM (44 mL),

maintained under argon, was charged with EDC hydrochloride (2.86 g, 15 mmol, 1.5 eq.), methyl alcohol (0.41 mL, 10 mmol, 1.0 eq.) and DMAP (0.24 g, 2 mmol, 0.2 eq.). The mixture was left stirring overnight. DCM (20 mL) was added to the mixture, which was then quenched with a saturated aqueous NH_4Cl solution (50 mL). The mixture was extracted with DCM (50 mL × 2), dried over anhydrous $MgSO_4$, filtered and concentrated by rotary evaporation. The residue was purified by silica gel column chromatography (PE/EA = 9:1, v/v) to afford ester **1-4**. An oven-dried 100 mL round bottom flask equipped with a stir bar was cooled under vacuum. After backfilled with Ar (x 3) and capped with a septum, ester **1-4** (2.24 g, 10.00 mmol, 1.00 eq.) and THF (35 mL) were added. The reaction was cooled to 0 °C in an ice/water bath and NaHMDS (6.0 mL, 2.0 M in THF, 12.0 mmol, 1.2 eq.) was added to the solution. The reaction was stirred at the same temperature overnight before quenched with saturated NH_4Cl solution (30 mL). The mixture was extracted with EA (10 mL x 3). The combined organic layers were washed with brine (25 mL), dried over anhydrous $MgSO_4$, filtered and concentrated by rotary evaporation. The residue was purified by silica gel column chromatography (PE/EA = 19:1, v/v) to afford **ester 1-5 (2l)**. An oven-dried 100 mL round bottom flask equipped with a stir bar was cooled under vacuum. After backfilled with Ar (x 3) and capped with a septum, above crude ester **1-5** (1.13 g, 6.00 mmol, 1.00 eq.) and THF (35 mL) were added. The reaction was cooled to 0 °C in an ice/water bath. MeNH(OMe)·HCl (0.88 g, 9 mmol, 1.5 eq.) and i-PrMgCl (9 mL, 2.0 M in THF, 18.0 mmol, 3.0 eq.) were sequentially added to the solution. After stirred at the same temperature for 4 h, the reaction was quenched by saturated NH_4Cl solution (20 mL). The aqueous layer was extracted with EA (10 mL x 3). The combined organic layers were washed with brine (25 mL), dried over anhydrous $MgSO_4$, filtered and concentrated by rotary evaporation. The residue was purified by silica gel column chromatography (PE/EA = 3:1, v/v) to afford ester **1-6**. An oven-dried 100 mL round bottom flask equipped with a stir bar was cooled under vacuum. After backfilled with Ar (x 3) and capped with a septum, 2-NaphBr (3.0 mmol, 1.5 eq.) and THF (20 mL) were added. The solution was cooled to -78 °C and n-BuLi (1.2 mL, 2.5 M in hexane, 3.0 mmol, 1.5 eq.) was added. After stirred at the

same temperature for 2 h, a solution of 1-6 (0.43 g, 2.0 mmol, 1.0 eq.) in THF (10 mL) was added. After 1.5 h, the resulted mixture was warmed up to room temperature and the mixture was stirred at the same temperature overnight before quenched with saturated NH_4Cl solution (20 mL). The aqueous layer was extracted with EA (10 mL x 3). The combined organic layers were washed with brine (25 mL), dried over anhydrous $MgSO_4$, filtered and concentrated by rotary evaporation. The residue was purified by silica gel column chromatography (PE/EA = 9:1, v/v) to afford **BCB compounds 1 (2k)**.

3.3 Enantioselective [2π + 2σ] Cycloaddition of BCB with α,β-Unsaturated Aldehydes

General Procedure for Enantioselective Cycloadditions.

To a 10 mL Schlenk tube equipped with a magnetic stir bar was added **cat.4d** (0.02 mmol, 0.2 eq.), **cat.5a** (0.02 mmol, 0.2 eq.), aldehydes **1** (0.1 mmol, 1.0 eq.), BCBs **2** (0.15 mmol, 1.5 eq.) and TFA (0.04 mmol, 0.4 eq.), then acetone (2 mL) was added. The resulting mixture was stirred at 10 °C for 24 h. Upon completion of the reaction, the reaction mixture was concentrated under reduced pressure, and the resulting crude mixture was purified by silica gel column chromatography to afford the pure product **3**.

(2R,3S)-4-(3,5-dimethyl-1H-pyrazole-1-carbonyl)-1,3-diphenylbicyclo[2.1.1]hexane-2-carbaldehyde (3a)

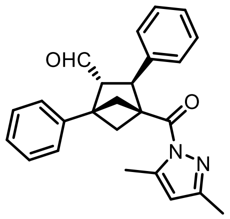

The product was purified by flash column chromatography on silica gel (PE/EA = 100:3-100:6) as a colourless oil at room temperature, 82% yield. R_f = 0.51 (PE/EA = 10:1). $[\alpha]_D^{22}$ = +107 (*c 1.0 (10mg/mL)*, CH_2Cl_2, 99% ee).

^{1}H NMR (600 MHz, CDCl3) δ 9.79 (d, J = 2.7 Hz, 1H), 7.40 – 7.31 (m, 2H), 7.29 – 7.23 (m, 5H), 7.19 (m, 3H), 5.92 (s, 1H), 4.83 (d, J = 5.1 Hz, 1H), 3.37 (dt, J = 4.7, 2.3 Hz, 1H), 2.90 (dd, J = 9.4, 7.4 Hz, 1H), 2.72 – 2.57 (m, 1H), 2.48 (dd, J = 7.4, 2.0 Hz, 1H), 2.43 (s, 3H), 2.26 (m, 4H).

^{13}C NMR (151 MHz, CDCl3) δ 202.8, 172.3, 152.4, 144.0, 140.4, 139.7, 128.7, 128.6, 127.4, 127.1, 126.8, 126.0, 110.7, 63.7, 55.5, 52.4, 50.3, 46.9, 44.6, 14.2, 14.0.

HPLC: Chiral IA-3 column, (n-hexane/i-PrOH = 90:10), flow rate1 mL/min, I = 250 nm; $t_{Renten\text{-}major}$ = 5.75 min, $t_{Renten\text{-}minor}$ = 6.77 min.

HRMS (ESI): $[M+H]^+$ calcd for $[C_{25}H_{25}N_2O_2]$: 385.1911, Found: 385.1907.

(2R,3S)-4-(3,5-dimethyl-1H-pyrazole-1-carbonyl)-1-phenyl-3-(p-tolyl)bicyclo[2.1.1]hexane-2-carbaldehyde (3b)

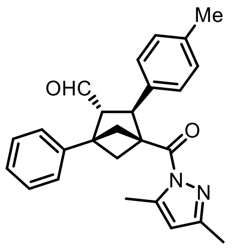

The product was purified by flash column chromatography on silica gel (PE/EA = 100:3-100:6) as a colourless oil at room temperature, 63% yield. R_f = 0.46 (PE/EA = 10:1). $[\alpha]_D^{22}$ = +37 (*c 1.0 (10mg/mL)*, CH_2Cl_2, 99% ee).

^{1}H NMR (600 MHz, CDCl3) δ 9.79 (d, J = 2.5 Hz, 1H), 7.35 (t, J = 7.6 Hz, 2H), 7.26 (m, 3H), 7.09 – 7.05 (m, 4H), 5.92 (s, 1H), 4.78 (d, J = 4.2 Hz, 1H), 3.37 – 3.34 (m, 1H), 2.90 (dd, J = 9.2, 7.6 Hz, 1H), 2.64 (dd, J = 9.1, 7.8 Hz, 1H), 2.48 (dd, J = 7.4, 1.8 Hz, 1H), 2.43 (s, 3H), 2.28 (s, 3H), 2.26 – 2.22 (m, 4H).

^{13}C NMR (151 MHz, CDCl3) δ 202.8, 172.4, 152.4, 144.0, 139.8, 137.3, 136.4, 129.3, 128.7, 127.3, 127.1, 126.0, 110.7, 63.7, 55.6, 52.4, 50.1, 47.0, 44.6, 20.9, 14.2, 14.0.

HPLC: Chiral IA-3 column, (n-hexane/i-PrOH = 95:5), flow rate1 mL/min, I = 250 nm; $t_{Renten\text{-}major}$ = 7.75 min, $t_{Renten\text{-}minor}$ = 10.46 min.

HRMS (ESI): $[M+H]^+$ calcd for $[C_{26}H_{27}N_2O_2]$: 399.2067, Found: 399.2065.

(2R,3S)-4-(3,5-dimethyl-1H-pyrazole-1-carbonyl)-3-(4-methoxyphenyl)-1-phenyl bicyclo[2.1.1]hexane-2-carbaldehyde (3c)

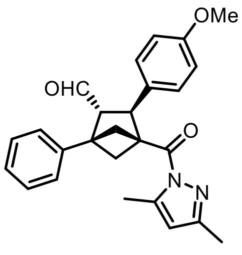

The product was purified by flash column chromatography on silica gel (PE/EA = 100:3-100:6) as a colourless oil at room temperature, 54% yield. R_f = 0.28 (PE/EA = 10:1). $[\alpha]_D^{22}$ = +46 (*c 1.0 (10mg/mL)*, CH_2Cl_2, 99% ee).

^{1}H NMR (600 MHz, $CDCl_3$) δ 9.78 (d, J = 2.6 Hz, 1H), 7.35 (t, J = 7.7 Hz, 2H), 7.27 – 7.24 (m, 3H), 7.10 (d, J = 8.6 Hz, 2H), 6.79 (d, J = 8.7 Hz, 2H), 5.92 (s, 1H), 4.74 (d, J = 4.2 Hz, 1H), 3.76 (s, 3H), 3.36 – 3.32 (m, 1H), 2.89 (dd, J = 9.2, 7.6 Hz, 1H), 2.64 (dd, J = 9.1, 7.8 Hz, 1H), 2.48 (dd, J = 7.4, 1.8 Hz, 1H), 2.43 (s, 3H), 2.26 (s, 3H), 2.22 (dd, J = 7.5, 1.3 Hz, 1H).

^{13}C NMR (151 MHz, $CDCl_3$) δ 202.9, 172.4, 158.5, 152.4, 144.0, 139.9, 132.3, 128.7, 128.5, 127.1, 126.1, 114.0, 110.7, 63.7, 55.8, 55.2, 52.3, 49.9, 47.0, 44.5, 14.2, 14.0.

HPLC: Chiral IA-3 column, (n-hexane/i-PrOH = 95:5), flow rate1 mL/min, I = 250 nm; $t_{Renten\text{-}major}$ = 10.84 min, $t_{Renten\text{-}minor}$ = 14.88 min.

HRMS (ESI): $[M+H]^+$ calcd for $[C_{26}H_{27}N_2O_3]$: 415.2016, Found: 415.2015.

(2R,3S)-4-(3,5-dimethyl-1H-pyrazole-1-carbonyl)-3-(4-fluorophenyl)-1-phenylbicyclo[2.1.1]hexane-2-carbaldehyde (3d)

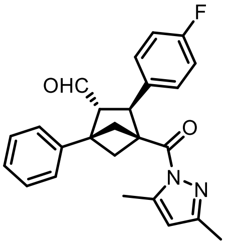

The product was purified by flash column chromatography on silica gel (PE/EA = 100:3-100:6) as a colourless oil at room temperature, 60% yield. R_f = 0.41 (PE/EA = 10:1). $[\alpha]_D^{22}$ = +49 (*c 1.0 (10mg/mL)*, CH_2Cl_2, 99% ee).

^{1}H NMR (600 MHz, $CDCl_3$) δ 9.77 (d, J = 2.4 Hz, 1H), 7.36 (t, J = 7.6 Hz, 2H), 7.29 – 7.24 (m, 3H), 7.19 – 7.13 (m, 2H), 6.98 – 6.90 (m, 2H), 5.93 (s, 1H), 4.79 (d, J = 4.4 Hz, 1H), 3.34 – 3.30 (m, 1H), 2.87 (dd, J = 9.3, 7.5 Hz, 1H), 2.63 (dd, J = 9.3, 7.8 Hz, 1H), 2.48 (dd, J = 7.4, 2.0 Hz, 1H), 2.43 (s, 3H), 2.29 – 2.21 (m, 4H).

^{13}C NMR (151 MHz, $CDCl_3$) δ 202.6, 172.2, 162.5, 160.9, 152.6, 144.1, 139.6, 136.0, 136.0, 129.0, 129.0, 128.7, 127.2, 126.0, 115.5, 115.3, 110.8, 63.7, 55.6, 52.4, 49.7, 46.9, 44.6, 14.2, 14.0.

^{19}F NMR (565 MHz, $CDCl_3$) δ -115.91.

HPLC: Chiral IC-3 column, (n-hexane/i-PrOH = 98:8), flow rate1 mL/min, I = 250 nm; $t_{Renten\text{-}major}$ = 11.60 min, $t_{Renten\text{-}minor}$ = 10.43 min.

HRMS (ESI): $[M+H]^+$ calcd for $[C_{25}H_{24}FN_2O_2]$: 403.1816, Found: 403.1817.

(2R,3S)-3-(4-chlorophenyl)-4-(3,5-dimethyl-1H-pyrazole-1-carbonyl)-1-phenylbicyclo[2.1.1]hexane-2-carbaldehyde (3e)

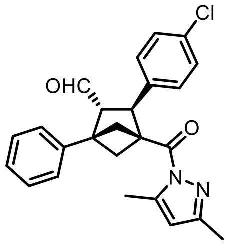

The product was purified by flash column chromatography on silica gel (PE/EA = 100:3-100:6) as a colourless oil at room temperature, 64% yield. R_f = 0.44 (PE/EA = 10:1). $[\alpha]_D^{22}$ = +31 (*c 1.0 (10mg/mL)*, CH_2Cl_2, 98% ee).

^{1}H NMR (600 MHz, $CDCl_3$) δ 9.76 (d, J = 2.2 Hz, 1H), 7.36 (t, J = 7.6 Hz, 2H), 7.28 (d, J = 7.4 Hz, 1H), 7.25 (m, 2H), 7.22 (d, J = 8.5 Hz, 2H), 7.14 (d, J = 8.5 Hz, 2H), 5.93 (s, 1H), 4.79 (d, J = 4.3 Hz, 1H), 3.31 – 3.29 (m, 1H), 2.85 (dd, J = 9.2, 7.7 Hz, 1H), 2.63 – 2.60 (m, 1H), 2.48 (dd, J = 7.4, 1.8 Hz, 1H), 2.44 (s, 3H), 2.28 – 2.24 (m, 4H).

^{13}C NMR (151 MHz, $CDCl_3$) δ 202.5, 172.1, 152.6, 144.1, 139.5, 138.9, 132.7, 128.8, 128.7, 128.7, 127.2, 126.0, 110.8, 63.7, 55.4, 52.4, 49.7, 46.9, 44.6, 29.7, 14.2, 14.0.

HPLC: Chiral IC-3 column, (n-hexane/i-PrOH = 98:2), flow rate1 mL/min, I = 250 nm; $t_{Renten\text{-}major}$ = 13.12 min, $t_{Renten\text{-}minor}$ = 11.31 min.

HRMS (ESI): $[M+H]^+$ calcd for $[C_{25}H_{24}ClN_2O_2]$: 419.1521, Found: 419.1520.

(2R,3S)-3-(4-bromophenyl)-4-(3,5-dimethyl-1H-pyrazole-1-carbonyl)-1-phenylbicyclo[2.1.1]hexane-2-carbaldehyde (3f)

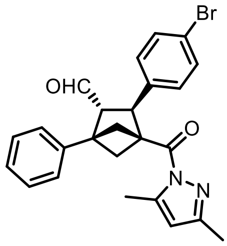

The product was purified by flash column chromatography on silica gel (PE/EA = 100:3-100:6) as a colourless oil at room temperature, 49% yield. R_f = 0.44 (PE/EA = 10:1). $[\alpha]_D^{22}$ = +32 (*c 1.0 (10mg/mL)*, CH_2Cl_2, 92% ee).

^{1}H NMR (600 MHz, $CDCl_3$) δ 9.76 (d, J = 2.2 Hz, 1H), 7.36 (m, 4H), 7.28 (d, J = 7.3 Hz, 1H), 7.26 – 7.23 (m, 2H), 7.08 (d, J = 8.4 Hz, 2H), 5.93 (s, 1H), 4.78 (d, J = 4.3 Hz, 1H), 3.31 – 3.28 (m, 1H), 2.84 (dd, J = 9.2, 7.7 Hz, 1H), 2.63 – 2.59 (m, 1H), 2.48 (dd, J = 7.4, 1.8 Hz, 1H), 2.44 (s, 3H), 2.29 – 2.23 (m, 4H).

^{13}C NMR (151 MHz, $CDCl_3$) δ 202.5, 172.1, 152.6, 144.1, 139.5, 139.5, 131.7, 129.2, 128.7, 127.2, 126.0, 120.7, 110.9, 63.8, 55.3, 52.4, 49.7, 46.9, 44.7, 14.2, 14.0.

HPLC: Chiral IC-3 column, (n-hexane/i-PrOH = 98:2), flow rate1 mL/min, I = 250 nm; $t_{Renten\text{-}major}$ = 13.90 min, $t_{Renten\text{-}minor}$ = 11.53 min.

HRMS (ESI): $[M+H]^+$ calcd for $[C_{25}H_{24}BrN_2O_2]$: 463.1016, Found: 463.1015.

(2R,3S)-4-(3,5-dimethyl-1H-pyrazole-1-carbonyl)-3-(4-nitrophenyl)-1-phenylbicyclo[2.1.1]hexane-2-carbaldehyde (3g)

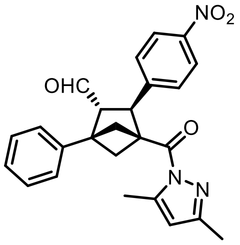

The product was purified by flash column chromatography on silica gel (PE/EA = 100:3-100:10) as a white molten oil at room temperature, 58% yield. R_f = 0.60 (PE/EA = 3:1). $[\alpha]_D^{22}$ = +11 (*c 1.0 (10mg/mL)*, CH_2Cl_2, 99% ee).

^{1}H NMR (600 MHz, $CDCl_3$) δ 9.76 (d, J = 1.8 Hz, 1H), 8.12 (d, J = 8.8 Hz, 2H), 7.42 – 7.36 (m, 4H), 7.28 (m, 3H), 4.97 (d, J = 4.1 Hz, 1H), 3.34 – 3.28 (m, 1H), 2.83 (dd, J = 9.3, 7.7 Hz, 1H), 2.63 (dd, J = 9.3, 8.0 Hz, 1H), 2.51 (dd, J = 7.5, 1.9 Hz, 1H), 2.45 (s, 3H), 2.36 (dd, J = 7.8, 1.6 Hz, 1H), 2.25 (s, 3H).

^{13}C NMR (151 MHz, $CDCl_3$) δ 201.9, 171.8, 152.9, 148.5, 146.8, 144.2, 139.0, 128.8, 128.3, 127.4, 125.9, 123.8, 111.1, 64.0, 55.2, 52.5, 49.9, 46.8, 45.0, 29.7, 14.2, 14.0.

HPLC: Chiral IF-3 column, (n-hexane/i-PrOH = 98:2), flow rate1 mL/min, I = 250 nm; $t_{Renten\text{-}major}$ = 29.66 min, $t_{Renten\text{-}minor}$ = 34.78 min.

HRMS (ESI): $[M+H]^+$ calcd for $[C_{25}H_{24}N_3O_4]$: 430.1761, Found: 430.1760.

4-((2S,3R)-1-(3,5-dimethyl-1H-pyrazole-1-carbonyl)-3-formyl-4-phenylbicyclo[2.1.1]hexan-2-yl)benzonitrile (3h)

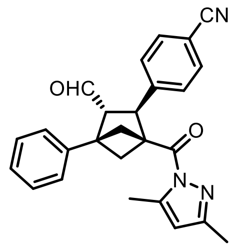

The product was purified by flash column chromatography on silica gel (PE/EA = 100:3-100:6) as a white molten oil at room temperature, 55% yield. R_f = 0.21 (PE/EA = 10:1). $[\alpha]_D^{22}$ = +75 (*c 1.0 (10mg/mL)*, CH_2Cl_2, 99% ee).

^{1}H NMR (600 MHz, $CDCl_3$) δ 9.75 (d, J = 1.9 Hz, 1H), 7.55 (d, J = 8.3 Hz, 2H), 7.39 – 7.32 (m, 4H), 7.29 (t, J = 7.4 Hz, 1H), 7.25 (m, 2H), 5.94 (s, 1H), 4.91 (d, J = 4.2 Hz, 1H), 3.32 – 3.26 (m, 1H), 2.82 (dd, J = 9.3, 7.7 Hz, 1H), 2.63 – 2.58 (m, 1H), 2.49 (dd, J = 7.5, 1.9 Hz, 1H), 2.45 (s, 3H), 2.33 (dd, J = 7.7, 1.5 Hz, 1H), 2.24 (s, 3H).

^{13}C NMR (151 MHz, $CDCl_3$) δ 202.1, 202.1, 171.8, 152.8, 146.3, 144.2, 139.1, 132.4, 128.8, 128.2, 127.4, 125.9, 118.7, 111.0, 110.7, 63.9, 55.1, 52.4, 50.0, 46.8, 44.9, 14.2, 14.0.

HPLC: Chiral IC-3 column, (n-hexane/i-PrOH = 90:10), flow rate1 mL/min, I = 250 nm; $t_{Renten\text{-}major}$ = 34.60 min, $t_{Renten\text{-}minor}$ = 37.61 min.

HRMS (ESI): $[M+H]^+$ calcd for $[C_{26}H_{24}N_3O_2]$: 410.1863, Found: 410.1861.

(2R,3S)-3-(4-(tert-butyl)phenyl)-4-(3,5-dimethyl-1H-pyrazole-1-carbonyl)-1-phenylbicyclo[2.1.1]hexane-2-carbaldehyde (3i)

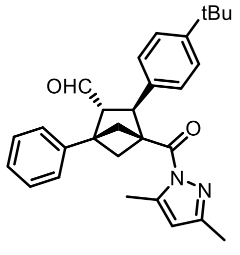

The product was purified by flash column chromatography on silica gel (PE/EA = 100:3-100:6) as a colourless oil at room temperature, 59% yield. R_f = 0.49 (PE/EA = 10:1). $[\alpha]_D^{22}$ = +45 (*c 1.0 (10mg/mL)*, CH_2Cl_2, 96% ee).

^{1}H NMR (600 MHz, $CDCl_3$) δ 9.79 (d, *J* = 2.6 Hz, 1H), 7.34 (t, *J* = 7.6 Hz, 2H), 7.26 (m, 5H), 7.13 (d, *J* = 8.3 Hz, 2H), 5.92 (s, 1H), 4.79 (d, *J* = 4.2 Hz, 1H), 3.35 (dt, *J* = 4.5, 2.2 Hz, 1H), 2.90 (dd, *J* = 9.2, 7.5 Hz, 1H), 2.65 (dd, *J* = 9.2, 7.7 Hz, 1H), 2.47 (dd, *J* = 7.4, 1.9 Hz, 1H), 2.44 (s, 3H), 2.27 – 2.23 (m, 4H), 1.27 (s, 9H).

^{13}C NMR (151 MHz, $CDCl_3$) δ 202.9, 172.5, 152.3, 149.5, 144.0, 139.8, 137.4, 128.6, 127.1, 127.0, 126.0, 125.4, 110.7, 63.9, 55.3, 52.3, 50.0, 46.9, 44.7, 34.4, 31.3, 14.2, 14.0.

HPLC: Chiral IA-3 column, (n-hexane/i-PrOH = 98:2), flow rate1 mL/min, I = 250 nm; $t_{Renten-major}$ = 7.31 min, $t_{Renten-minor}$ = 8.18 min.

HRMS (ESI): $[M+H]^+$ calcd for $[C_{29}H_{33}N_2O_2]$: 441.2537, Found: 441.2534.

Methyl 4-((2S,3R)-1-(3,5-dimethyl-1H-pyrazole-1-carbonyl)-3-formyl-4-phenylbicyclo[2.1.1]hexan-2-yl)benzoate (3j)

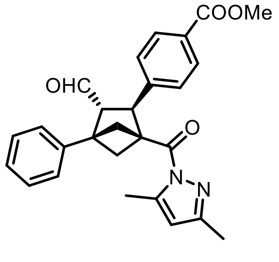

The product was purified by flash column chromatography on silica gel (PE/EA = 100:3-100:6) as a white molten oil at room temperature, 69% yield. R_f = 0.23 (PE/EA = 10:1). $[\alpha]_D^{22}$ = +34 (*c 1.0 (10mg/mL)*, CH_2Cl_2, 98% ee).

^{1}H NMR (600 MHz, $CDCl_3$) δ 9.77 (d, *J* = 2.0 Hz, 1H), 7.92 (d, *J* = 8.2 Hz, 2H), 7.36 (t, *J* = 7.6 Hz, 2H), 7.30 – 7.25 (m, 5H), 5.93 (s, 1H), 4.90 (d, *J* = 4.5 Hz, 1H), 3.89 (s,

3H), 3.38 – 3.33 (m, 1H), 2.89 – 2.84 (m, 1H), 2.66 – 2.62 (m, 1H), 2.49 (dd, J = 7.4, 1.5 Hz, 1H), 2.43 (s, 3H), 2.30 (d, J = 7.7 Hz, 1H), 2.25 (s, 3H).

^{13}C NMR (151 MHz, $CDCl_3$) δ 202.4, 172.0, 166.8, 152.6, 145.9, 144.1, 139.4, 129.9, 128.7, 128.7, 127.5, 127.3, 126.0, 110.9, 63.6, 55.4, 52.4, 52.0, 50.1, 46.9, 44.7, 14.2, 14.0.

HPLC: Chiral IA-3 column, (n-hexane/i-PrOH = 98:2), flow rate1 mL/min, I = 250 nm; $t_{Renten\text{-}major}$ = 11.47 min, $t_{Renten\text{-}minor}$ = 19.97 min.

HRMS (ESI): $[M+H]^+$ calcd for $[C_{27}H_{27}N_2O_4]$: 443.1965, Found: 443.1963.

(2R,3S)-4-(3,5-dimethyl-1H-pyrazole-1-carbonyl)-1-phenyl-3-(4-((trifluoromethyl)thio)phenyl)bicyclo[2.1.1]hexane-2-carbaldehyde (3k)

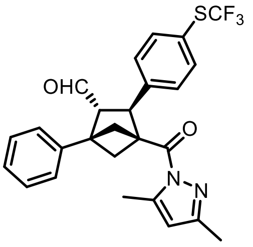

The product was purified by flash column chromatography on silica gel (PE/EA = 100:3-100:6) as a white molten oil at room temperature, 65% yield. R_f = 0.48 (PE/EA = 10:1). $[\alpha]_D^{22}$ = +28 (*c 1.0 (10mg/mL)*, CH_2Cl_2, 98% ee).

^{1}H NMR (600 MHz, $CDCl_3$) δ 9.77 (d, J = 2.1 Hz, 1H), 7.54 (d, J = 8.2 Hz, 2H), 7.36 (t, J = 7.6 Hz, 2H), 7.30 – 7.24 (m, 5H), 5.93 (s, 1H), 4.87 (d, J = 4.3 Hz, 1H), 3.35 – 3.29 (m, 1H), 2.84 (dd, J = 9.3, 7.6 Hz, 1H), 2.63 (dd, J = 9.2, 7.9 Hz, 1H), 2.49 (dd, J = 7.5, 1.9 Hz, 1H), 2.44 (s, 3H), 2.32 (dd, J = 7.7, 1.5 Hz, 1H), 2.24 (s, 3H).

^{13}C NMR (151 MHz, $CDCl_3$) δ 202.3, 172.0, 152.7, 144.1, 144.0, 139.3, 136.5, 130.5, 128.8, 128.6, 128.5, 127.3, 126.0, 122.6, 110.9, 64.0, 55.2, 52.4, 49.9, 46.8, 44.9, 14.2, 14.0.

^{19}F NMR (565 MHz, $CDCl_3$) δ -42.74.

HPLC: Chiral IA-3 column, (n-hexane/i-PrOH = 98:2), flow rate1 mL/min, I = 250 nm; $t_{Renten\text{-}major}$ = 10.25 min, $t_{Renten\text{-}minor}$ = 12.75 min.

HRMS (ESI): $[M+H]^+$ calcd for $[C_{26}H_{24}F3N_2O_2S]$: 485.1505, Found: 485.1503.

(2R,3S)-3-([1,1'-biphenyl]-4-yl)-4-(3,5-dimethyl-1H-pyrazole-1-carbonyl)-1-phenylbicyclo[2.1.1]hexane-2-carbaldehyde (3l)

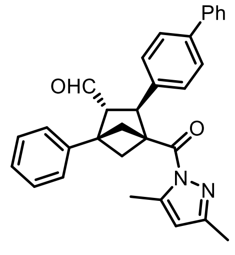

The product was purified by flash column chromatography on silica gel (PE/EA = 100:3-100:6) as a white molten oil at room temperature, 67% yield. R_f = 0.39 (PE/EA = 10:1). $[\alpha]_D^{22}$ = +4 (*c 1.0 (10mg/mL)*, CH_2Cl_2, 98% ee).

^{1}H NMR (600 MHz, $CDCl_3$) δ 9.81 (m, 1H), 7.54 (m, 2H), 7.48 (m, 2H), 7.41 (m, 2H), 7.36 (dd, *J* = 11.4, 4.0 Hz, 2H), 7.34 – 7.30 (m, 1H), 7.29 – 7.25 (m, 5H), 5.93 (s, 1H), 4.90 – 4.85 (m, 1H), 3.41 (m, 1H), 2.93 – 2.88 (m, 1H), 2.73 – 2.67 (m, 1H), 2.50 (m, 1H), 2.45 (s, 3H), 2.29 (m, 1H), 2.26 (d, *J* = 1.1 Hz, 3H).

^{13}C NMR (151 MHz, $CDCl_3$) δ 202.7, 172.3, 152.5, 144.0, 140.7, 139.7, 139.6, 139.5, 128.7, 128.7, 127.8, 127.3, 127.2, 127.1, 127.0, 126.0, 110.8, 63.8, 55.5, 52.4, 50.1, 46.9, 44.7, 14.2, 14.0.

HPLC: Chiral IA-3 column, (n-hexane/i-PrOH = 90:10), flow rate1 mL/min, I = 250 nm; $t_{Renten-major}$ = 9.15 min, $t_{Renten-minor}$ = 12.19 min.

HRMS (ESI): $[M+H]^+$ calcd for $[C_{31}H_{29}N_2O_2]$: 461.2224, Found: 461.2224.

(2R,3S)-4-(3,5-dimethyl-1H-pyrazole-1-carbonyl)-1-phenyl-3-(m-tolyl)bicyclo[2.1.1]hexane-2-carbaldehyde (3m)

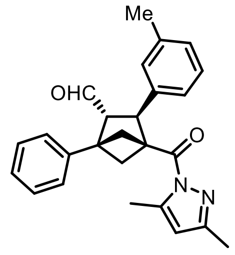

The product was purified by flash column chromatography on silica gel (PE/EA = 100:3-100:6) as a colourless oil at room temperature, 67% yield. R_f = 0.45 (PE/EA = 10:1). $[\alpha]_D^{22}$ = +63 (*c 1.0 (10mg/mL)*, CH_2Cl_2, 98% ee).

^{1}H NMR (600 MHz, $CDCl_3$) δ 9.79 (d, *J* = 2.5 Hz, 1H), 7.35 (t, *J* = 7.7 Hz, 2H), 7.27 (m, 3H), 7.14 (t, *J* = 7.6 Hz, 1H), 6.99 (m, 9.4 Hz, 3H), 5.93 (s, 1H), 4.78 (d, *J* = 4.3

Hz, 1H), 3.37 (dt, J = 4.4, 2.0 Hz, 1H), 2.90 (dd, J = 9.2, 7.6 Hz, 1H), 2.65 (dd, J = 9.2, 7.8 Hz, 1H), 2.48 (dd, J = 7.4, 1.8 Hz, 1H), 2.43 (s, 3H), 2.25 (m, 7H).

^{13}C NMR (151 MHz, $CDCl_3$) δ 202.9, 152.4, 144.0, 140.3, 139.8, 138.1, 128.7, 128.4, 128.3, 127.5, 127.1, 126.0, 124.2, 110.7, 63.5, 55.5, 52.4, 50.2, 46.9, 44.6, 21.5, 14.2, 14.0.

HPLC: Chiral IC-3 column, (n-hexane/i-PrOH = 98:2), flow rate1 mL/min, I = 250 nm; $t_{Renten\text{-}major}$ = 12.68 min, $t_{Renten\text{-}minor}$ = 11.42 min.

HRMS (ESI): $[M+H]^+$ calcd for $[C_{26}H_{27}N_2O_2]$: 399.2067, Found: 399.2065.

(2R,3S)-4-(3,5-dimethyl-1H-pyrazole-1-carbonyl)-3-(3-methoxyphenyl)-1-phenyl bicyclo[2.1.1]hexane-2-carbaldehyde (3n)

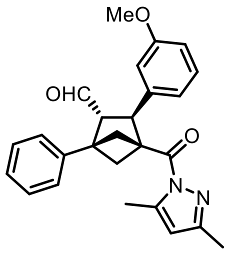

The product was purified by flash column chromatography on silica gel (PE/EA = 100:3-100:6) as a colourless oil at room temperature, 57% yield. R_f = 0.30 (PE/EA = 10:1). $[\alpha]_D^{22}$ = +50 (*c 1.0 (10mg/mL)*, CH_2Cl_2, 98% ee).

^{1}H NMR (600 MHz, $CDCl_3$) δ 9.78 (d, J = 2.4 Hz, 1H), 7.35 (t, J = 7.6 Hz, 2H), 7.29 – 7.23 (m, 3H), 7.20 – 7.15 (m, 1H), 6.79 (d, J = 7.7 Hz, 1H), 6.73 (d, J = 6.1 Hz, 2H), 5.93 (s, 1H), 4.81 (d, J = 4.2 Hz, 1H), 3.71 (s, 3H), 3.39 – 3.33 (m, 1H), 2.88 (dd, J = 9.2, 7.6 Hz, 1H), 2.69 – 2.63 (m, 1H), 2.48 (dd, J = 7.4, 1.8 Hz, 1H), 2.44 (s, 3H), 2.26 (s, 4H).

^{13}C NMR (151 MHz, $CDCl_3$) δ 202.8, 172.3, 159.6, 152.4, 144.0, 142.0, 139.7, 129.5, 128.7, 127.1, 126.0, 119.6, 113.1, 112.3, 110.7, 63.6, 55.4, 55.0, 52.3, 50.2, 46.9, 44.7, 14.2, 14.0.

HPLC: Chiral IA-3 column, (n-hexane/i-PrOH = 98:2), flow rate1 mL/min, I = 250 nm; $t_{Renten\text{-}major}$ = 12.14 min, $t_{Renten\text{-}minor}$ = 15.18 min.

HRMS (ESI): $[M+H]^+$ calcd for $[C_{26}H_{27}N_2O_3]$: 415.2016, Found: 415.2015.

(2R,3S)-4-(3,5-dimethyl-1H-pyrazole-1-carbonyl)-3-(3-fluorophenyl)-1-phenylbicyclo[2.1.1]hexane-2-carbaldehyde (3o)

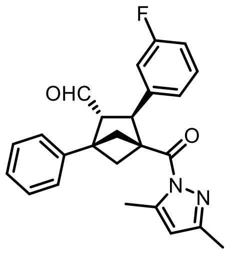

The product was purified by flash column chromatography on silica gel (PE/EA = 100:3-100:6) as a colourless oil at room temperature, 69% yield. R_f = 0.43 (PE/EA = 10:1). $[\alpha]_D^{22}$ = +12 (*c 1.0 (10mg/mL)*, CH_2Cl_2, 97% ee).

^{1}H NMR (600 MHz, $CDCl_3$) δ 9.77 (d, J = 2.2 Hz, 1H), 7.36 (t, J = 7.6 Hz, 2H), 7.30 – 7.24 (m, 3H), 7.21 (dd, J = 14.2, 7.9 Hz, 1H), 6.97 (d, J = 7.8 Hz, 1H), 6.89 (m, 2H), 5.93 (s, 1H), 4.82 (d, J = 4.3 Hz, 1H), 3.36 – 3.31 (m, 1H), 2.85 (dd, J = 9.3, 7.6 Hz, 1H), 2.63 (dd, J = 9.2, 8.0 Hz, 1H), 2.48 (dd, J = 7.4, 1.9 Hz, 1H), 2.44 (s, 3H), 2.29 – 2.24 (m, 4H).

^{13}C NMR (151 MHz, $CDCl_3$) δ 202.4, 172.0, 163.7, 162.1, 152.6, 144.1, 143.1, 143.0, 139.5, 130.1, 130.0, 128.7, 127.2, 126.0, 123.1, 123.1, 114.6, 114.5, 113.8, 113.7, 110.8, 63.6, 55.4, 52.4, 49.9, 46.9, 44.7, 14.2, 14.0.

^{19}F NMR (565 MHz, $CDCl_3$) δ -112.58.

HPLC: Chiral IA-3 column, (n-hexane/i-PrOH = 98:2), flow rate1 mL/min, I = 250 nm; $t_{Renten\text{-}major}$ = 8.76 min, $t_{Renten\text{-}minor}$ = 10.65 min.

HRMS (ESI): $[M+H]^+$ calcd for $[C_{25}H_{24}FN_2O_2]$: 403.1816, Found: 403.1817.

(2R,3S)-3-(3-chlorophenyl)-4-(3,5-dimethyl-1H-pyrazole-1-carbonyl)-1-phenylbicyclo[2.1.1]hexane-2-carbaldehyde (3p)

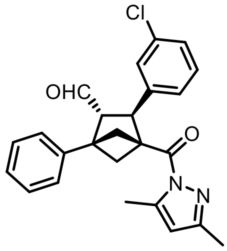

The product was purified by flash column chromatography on silica gel (PE/EA = 100:3-100:6) as a colourless oil at room temperature, 78% yield. R_f = 0.42 (PE/EA = 10:1). $[\alpha]_D^{22}$ = +74 (*c 1.0 (10mg/mL)*, CH_2Cl_2, 97% ee).

^{1}H NMR (600 MHz, $CDCl_3$) δ 9.77 (t, J = 8.6 Hz, 1H), 7.36 (t, J = 7.6 Hz, 2H), 7.30 – 7.24 (m, 3H), 7.21 – 7.13 (m, 3H), 7.10 – 7.05 (m, 1H), 5.94 (s, 1H), 4.79 (d, J = 3.6 Hz, 1H), 3.34 (dt, J = 4.5, 2.1 Hz, 1H), 2.84 (dd, J = 9.4, 7.5 Hz, 1H), 2.62 (dd, J = 9.4, 7.8 Hz, 1H), 2.48 (dd, J = 7.5, 2.0 Hz, 1H), 2.44 (s, 3H), 2.28 (dd, J = 7.7, 1.6 Hz, 1H), 2.26 (s, 3H).

^{13}C NMR (151 MHz, $CDCl_3$) δ 202.3, 172.0, 152.6, 144.1, 142.5, 139.4, 134.4, 129.8, 128.7, 127.8, 127.3, 127.0, 126.0, 125.5, 110.8, 63.5, 55.4, 52.4, 49.8, 46.8, 44.7, 14.2, 14.0.

HPLC: Chiral IF-3 column, (n-hexane/i-PrOH = 98:2), flow rate1 mL/min, I = 250 nm; $t_{Renten\text{-}major}$ = 8.50 min, $t_{Renten\text{-}minor}$ = 9.35 min.

HRMS (ESI): $[M+H]^+$ calcd for $[C_{25}H_{24}ClN_2O_2]$: 419.1521, Found: 419.1520.

(2R,3S)-3-(3-bromophenyl)-4-(3,5-dimethyl-1H-pyrazole-1-carbonyl)-1-phenylbicyclo[2.1.1]hexane-2-carbaldehyde (3q)

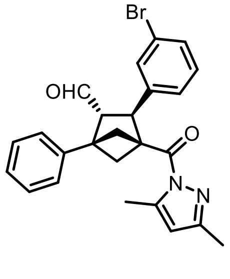

The product was purified by flash column chromatography on silica gel (PE/EA = 100:3-100:6) as a colourless oil at room temperature, 64% yield. R_f = 0.42 (PE/EA = 10:1). $[\alpha]_D^{22}$ = +40 (*c 1.0 (10mg/mL)*, CH_2Cl_2, 95% ee).

^{1}H NMR (600 MHz, CDCl3) δ 9.75 (d, J = 2.2 Hz, 1H), 7.40 – 7.34 (m, 2H), 7.34 – 7.23 (m, 5H), 7.15 – 7.09 (m, 2H), 5.95 (s, 1H), 4.79 (d, J = 3.1 Hz, 1H), 3.34 (dt, J = 4.5, 2.2 Hz, 1H), 2.88 – 2.81 (m, 1H), 2.66 – 2.58 (m, 1H), 2.48 (dd, J = 7.4, 2.2 Hz, 1H), 2.45 (s, 3H), 2.32 – 2.23 (m, 4H).

^{13}C NMR (151 MHz, CDCl3) δ 202.4, 172.0, 152.7, 144.1, 142.8, 139.4, 130.7, 130.1, 129.9, 128.7, 127.3, 126.0, 125.9, 122.7, 110.9, 63.4, 55.4, 52.4, 49.7, 46.8, 44.6, 14.2, 14.0.

HPLC: Chiral IF-3 column, (n-hexane/i-PrOH = 98:2), flow rate1 mL/min, I = 250 nm; $t_{Renten\text{-}major}$ = 9.48 min, $t_{Renten\text{-}minor}$ = 11.04 min.

HRMS (ESI): $[M+H]^+$ calcd for $[C_{25}H_{24}BrN_2O_2]$: 463.1016, Found: 463.1016.

(2R,3S)-4-(3,5-dimethyl-1H-pyrazole-1-carbonyl)-1-phenyl-3-(3-(trifluoromethyl)phenyl)bicyclo[2.1.1]hexane-2-carbaldehyde (3r)

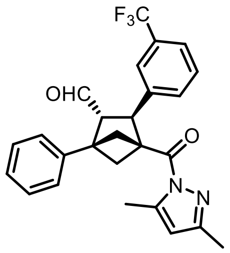

The product was purified by flash column chromatography on silica gel (PE/EA = 100:3-100:6) as a colourless oil at room temperature, 61% yield. R_f = 0.39 (PE/EA = 10:1). $[\alpha]_D^{22}$ = +52 (*c 1.0 (10mg/mL)*, CH_2Cl_2, 99% ee).

^{1}H NMR (600 MHz, $CDCl_3$) δ 9.77 (d, *J* = 1.9 Hz, 1H), 7.44 (m, 2H), 7.38 (m, 4H), 7.28 (m, 3H), 5.94 (s, 1H), 4.86 (d, *J* = 4.1 Hz, 1H), 3.42 – 3.35 (m, 1H), 2.86 (dd, *J* = 9.1, 7.8 Hz, 1H), 2.62 (dd, *J* = 8.1, 1.1 Hz, 1H), 2.50 (dd, *J* = 7.4, 1.6 Hz, 1H), 2.42 (s, 3H), 2.30 (dd, *J* = 7.8, 1.4 Hz, 1H), 2.25 (s, 3H).

^{13}C NMR (151 MHz, $CDCl_3$) δ 202.29, 171.89, 152.77, 144.17, 141.34, 139.33, 130.63, 129.03, 128.77, 127.32, 125.99, 124.45, 124.43, 123.74, 123.71, 123.69, 123.66 (q, *J* = 3.4, 3.4 Hz), 110.89, 63.18, 55.44, 52.53, 49.90, 46.73, 44.69, 14.11, 13.96.

^{19}F NMR (565 MHz, $CDCl_3$) δ -62.71.

HPLC: Chiral IF-3 column, (n-hexane/i-PrOH = 98:2), flow rate1 mL/min, I = 250 nm; $t_{Renten\text{-}major}$ = 7.49 min, $t_{Renten\text{-}minor}$ = 7.93 min.

HRMS (ESI): $[M+H]^+$ calcd for $[C_{26}H_{24}F_3N_2O_2]$: 453.1784, Found: 453.1787.

(2R,3S)-4-(3,5-dimethyl-1H-pyrazole-1-carbonyl)-3-(naphthalen-2-yl)-1-phenylbicyclo[2.1.1]hexane-2-carbaldehyde (3s)

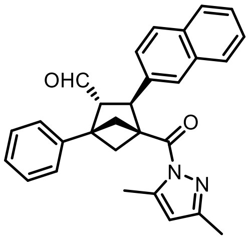

The product was purified by flash column chromatography on silica gel (PE/EA = 100:3-100:6) as a white molten oil at room temperature, 74% yield. R_f = 0.39 (PE/EA = 10:1). $[\alpha]_D^{22}$ = +21 (*c 1.0 (10mg/mL)*, CH_2Cl_2, 98% ee).

^{1}H NMR (600 MHz, $CDCl_3$) δ 9.84 (d, *J* = 2.4 Hz, 1H), 7.76 (d, *J* = 8.4 Hz, 2H), 7.74 – 7.69 (m, 2H), 7.46 – 7.40 (m, 2H), 7.36 (t, *J* = 7.6 Hz, 2H), 7.30 – 7.26 (m, 3H), 7.22 (dd, *J* = 8.5, 1.4 Hz, 1H), 5.91 (s, 1H), 4.99 (d, *J* = 4.4 Hz, 1H), 3.49 – 3.43 (m, 1H), 2.94 (dd, *J* = 9.2, 7.6 Hz, 1H), 2.79 – 2.74 (m, 1H), 2.53 (dd, *J* = 7.3, 1.7 Hz, 1H), 2.42 (s, 3H), 2.33 (dd, *J* = 7.6, 1.2 Hz, 1H), 2.25 (s, 3H).

^{13}C NMR (151 MHz, $CDCl_3$) δ 202.8, 172.4, 152.5, 144.1, 139.7, 138.0, 133.3, 132.3, 128.7, 128.3, 127.8, 127.5, 127.2, 126.4, 126.1, 126.0, 125.7, 125.3, 110.7, 63.8, 55.4, 52.5, 50.4, 47.0, 44.8, 14.2, 14.0.

HPLC: Chiral IC-3 column, (n-hexane/i-PrOH = 98:2), flow rate1 mL/min, I = 250 nm; $t_{Renten-major}$ = 16.04 min, $t_{Renten-minor}$ = 17.66 min.

HRMS (ESI): $[M+H]^+$ calcd for $[C_{29}H_{27}N_2O_2]$: 435.2067, Found: 435.2068.

(2R,3S)-3-(2,3-dihydrobenzo[b][1,4]dioxin-6-yl)-4-(3,5-dimethyl-1H-pyrazole-1-carbonyl)-1-phenylbicyclo[2.1.1]hexane-2-carbaldehyde (3t)

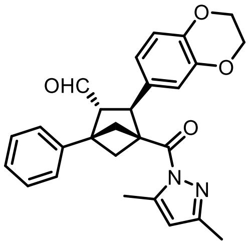

The product was purified by flash column chromatography on silica gel (PE/EA = 100:3-100:6) as a white molten oil at room temperature, 68% yield. R_f = 0.40 (PE/EA = 10:1). $[\alpha]_D^{22}$ = +25 (*c 1.0 (10mg/mL)*, CH_2Cl_2, 99% ee).

^{1}H NMR (600 MHz, $CDCl_3$) δ 9.76 (d, *J* = 2.5 Hz, 1H), 7.35 (t, *J* = 7.6 Hz, 2H), 7.26 (t, *J* = 8.8 Hz, 3H), 6.72 (m, 2H), 6.58 (dd, *J* = 8.4, 2.1 Hz, 1H), 5.93 (s, 1H), 4.69 (d, *J* = 4.3 Hz, 1H), 4.21 (s, 4H), 3.35 – 3.30 (m, 1H), 2.87 (dd, *J* = 9.2, 7.6 Hz, 1H), 2.62

(dd, J = 9.1, 7.9 Hz, 1H), 2.46 (dd, J = 7.4, 1.7 Hz, 1H), 2.44 (s, 3H), 2.26 (s, 3H), 2.20 (dd, J = 7.5, 1.1 Hz, 1H).

^{13}C NMR (151 MHz, $CDCl_3$) δ 202.7, 172.2, 152.4, 144.0, 143.3, 142.4, 139.8, 133.4, 128.7, 127.1, 126.0, 120.5, 117.2, 116.3, 110.7, 64.3, 64.3, 63.4, 55.8, 52.2, 49.8, 46.9, 44.4, 14.2, 14.0.

HPLC: Chiral IA-3 column, (n-hexane/i-PrOH = 90:10), flow rate1 mL/min, I = 250 nm; $t_{Renten\text{-}major}$ = 11.31 min, $t_{Renten\text{-}minor}$ = 12.56 min.

HRMS (ESI): $[M+H]^+$ calcd for $[C_{27}H_{27}N_2O_4]$: 443.1965, Found: 443.1964.

(2R,3R)-4-(3,5-dimethyl-1H-pyrazole-1-carbonyl)-1-phenyl-3-(thiophen-2-yl)bicyclo[2.1.1]hexane-2-carbaldehyde (3u)

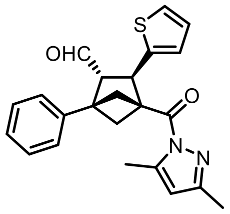

The product was purified by flash column chromatography on silica gel (PE/EA = 100:3-100:6) as a colourless oil at room temperature, 40% yield. R_f = 0.48 (PE/EA = 10:1). $[\alpha]_D^{22}$ = +37 (*c 1.0 (10mg/mL)*, CH_2Cl_2, 97% ee).

^{1}H NMR (600 MHz, $CDCl_3$) δ 9.75 (d, J = 2.2 Hz, 1H), 7.37 (t, J = 7.5 Hz, 2H), 7.28 (m, 3H), 7.14 (d, J = 5.0 Hz, 1H), 6.89 (dd, J = 4.9, 3.7 Hz, 1H), 6.77 (d, J = 3.4 Hz, 1H), 5.96 (s, 1H), 5.05 (d, J = 4.4 Hz, 1H), 3.45 – 3.37 (m, 1H), 2.86 (dd, J = 9.2, 7.7 Hz, 1H), 2.68 – 2.63 (m, 1H), 2.49 (dd, J = 7.4, 1.7 Hz, 1H), 2.45 (s, 3H), 2.28 (s, 3H), 2.25 (dd, J = 7.7, 1.3 Hz, 1H).

^{13}C NMR (151 MHz, $CDCl_3$) δ 201.9, 171.5, 152.6, 144.1, 143.4, 139.5, 128.7, 127.2, 126.8, 126.0, 124.9, 124.2, 110.7, 65.1, 56.4, 52.4, 46.4, 46.3, 44.8, 14.2, 14.0.

HPLC: Chiral IC-3 column, (n-hexane/i-PrOH = 98:2), flow rate1 mL/min, I = 250 nm; $t_{Renten\text{-}major}$ = 16.61 min, $t_{Renten\text{-}minor}$ = 13.13 min.

HRMS (ESI): $[M+H]^+$ calcd for $[C_{23}H_{23}N_2O_2S]$: 391.1475, Found: 391.1473.

(2R,3R)-4-(3,5-dimethyl-1H-pyrazole-1-carbonyl)-1-phenyl-3-(thiophen-3-yl)bicyclo[2.1.1]hexane-2-carbaldehyde (3v)

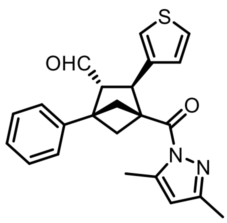

The product was purified by flash column chromatography on silica gel (PE/EA = 100:3-100:6) as a colourless oil at room temperature, 44% yield. R_f = 0.45 (PE/EA = 10:1). $[\alpha]_D^{22}$ = +48 (*c 1.0 (10mg/mL)*, CH_2Cl_2, 98% ee).

^{1}H NMR (600 MHz, $CDCl_3$) δ 9.78 (d, J = 2.3 Hz, 1H), 7.35 (m, 2H), 7.29 – 7.21 (m, 4H), 7.07 (s, 1H), 6.80 (d, J = 4.4 Hz, 1H), 5.94 (s, 1H), 4.82 (d, J = 3.9 Hz, 1H), 3.29 (m, 1H), 2.91 – 2.82 (m, 1H), 2.58 m, 1H), 2.47 (m, 4H), 2.25 (m, 4H).

^{13}C NMR (151 MHz, $CDCl_3$) δ 202.6, 172.3, 152.5, 144.0, 141.2, 139.7, 128.7, 127.5, 127.2, 126.0, 125.9, 120.6, 110.8, 64.0, 55.3, 52.4, 46.6, 46.5, 44.9, 14.3, 14.0.

HPLC: Chiral IC-3 column, (n-hexane/i-PrOH = 98:2), flow rate1 mL/min, I = 250 nm; $t_{Renten-major}$ = 13.90 min, $t_{Renten-minor}$ = 12.49 min.

HRMS (ESI): $[M+H]^+$ calcd for $[C_{23}H_{23}N_2O_2S]$: 391.1475, Found: 391.1474.

(2R,3R)-4-(3,5-dimethyl-1H-pyrazole-1-carbonyl)-3-(furan-2-yl)-1-phenylbicyclo [2.1.1]hexane-2-carbaldehyde (3w)

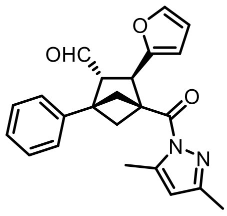

The product was purified by flash column chromatography on silica gel (PE/EA = 100:3-100:6) as a colourless oil at room temperature, 43% yield. R_f = 0.39 (PE/EA = 10:1). $[\alpha]_D^{22}$ = +38 (*c 1.0 (10mg/mL)*, CH_2Cl_2, 97% ee).

^{1}H NMR (600 MHz, $CDCl_3$) δ 9.74 (d, J = 2.2 Hz, 1H), 7.36 (m, 2H), 7.28 (m, 4H), 6.24 (dd, J = 3.1, 1.9 Hz, 1H), 6.04 (d, J = 3.2 Hz, 1H), 5.94 (s, 1H), 4.75 (d, J = 3.5 Hz, 1H), 3.47 (dt, J = 4.3, 2.0 Hz, 1H), 2.81 (dd, J = 9.3, 7.6 Hz, 1H), 2.63 (dd, J = 9.3, 7.5 Hz, 1H), 2.49 (s, 3H), 2.44 (dd, J = 7.5, 1.9 Hz, 1H), 2.25 (s, 3H), 2.19 (dd, J = 7.4, 1.7 Hz, 1H).

^{13}C NMR (151 MHz, $CDCl_3$) δ 202.0, 171.8, 153.9, 152.4, 143.9, 142.0, 139.6, 128.7, 127.2, 126.0, 110.6, 110.1, 106.5, 61.7, 55.0, 52.3, 45.9, 45.2, 44.4, 14.2, 14.0.

HPLC: Chiral IA-3 column, (n-hexane/i-PrOH = 90:10), flow rate1 mL/min, I = 250 nm; $t_{Renten\text{-}major}$ = 6.79 min, $t_{Renten\text{-}minor}$ = 7.19 min.

HRMS (ESI): $[M+H]^+$ calcd for $[C_{23}H_{23}N_2O_3]$: 375.1703, Found: 375.1702.

(2R,3S)-4-(3,5-dimethyl-1H-pyrazole-1-carbonyl)-3-(6-methoxypyridin-3-yl)-1-phenylbicyclo[2.1.1]hexane-2-carbaldehyde (3x)

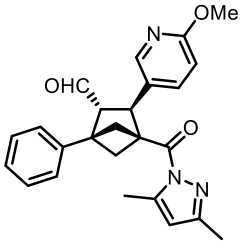

The product was purified by flash column chromatography on silica gel (PE/EA = 100:3-100:6) as a colourless oil at room temperature, 47% yield. R_f = 0.18 (PE/EA = 10:1). $[\alpha]_D^{22}$ = +20 (*c 1.0 (10mg/mL)*, CH_2Cl_2, 99% ee).

^{1}H NMR (600 MHz, $CDCl_3$) δ 9.76 (d, J = 2.0 Hz, 1H), 8.00 (d, J = 2.1 Hz, 1H), 7.42 (dd, J = 8.6, 2.4 Hz, 1H), 7.37 (t, J = 7.6 Hz, 2H), 7.27 (m, 3H), 6.65 (d, J = 8.6 Hz, 1H), 5.93 (s, 1H), 4.75 (d, J = 4.4 Hz, 1H), 3.88 (s, 3H), 3.37 – 3.34 (m, 1H), 2.86 (dd, J = 9.0, 7.9 Hz, 1H), 2.64 – 2.59 (m, 1H), 2.48 (dd, J = 7.4, 1.4 Hz, 1H), 2.43 (s, 3H), 2.25 (m, 4H).

^{13}C NMR (151 MHz, $CDCl_3$) δ 202.3, 171.8, 163.2, 152.7, 145.8, 144.1, 139.5, 138.0, 128.7, 128.2, 127.3, 126.0, 110.9, 110.7, 63.0, 55.7, 53.4, 52.4, 47.6, 46.8, 44.5, 14.2, 14.0.

HPLC: Chiral IA-3 column, (n-hexane/i-PrOH = 90:10), flow rate1 mL/min, I = 250 nm; $t_{Renten\text{-}major}$ = 7.79 min, $t_{Renten\text{-}minor}$ = 8.79 min.

HRMS (ESI): $[M+H]^+$ calcd for $[C_{25}H_{26}N_3O_3]$: 416.1969, Found: 416.1969.

(2R,3R)-3-(benzofuran-2-yl)-4-(3,5-dimethyl-1H-pyrazole-1-carbonyl)-1-phenylbicyclo[2.1.1]hexane-2-carbaldehyde (3y)

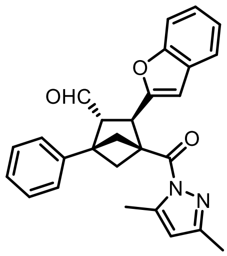

The product was purified by flash column chromatography on silica gel (PE/EA = 100:3-100:6) as a colourless oil at room temperature, 60% yield. R_f = 0.40 (PE/EA = 10:1). $[\alpha]_D^{22}$ = +14 (*c 1.0 (10mg/mL)*, CH_2Cl_2, 96% ee).

^{1}H NMR (600 MHz, $CDCl_3$) δ 9.78 (d, *J* = 1.9 Hz, 1H), 7.45 (d, *J* = 7.6 Hz, 1H), 7.41 – 7.32 (m, 3H), 7.29 (t, *J* = 6.8 Hz, 3H), 7.20 (t, *J* = 7.7 Hz, 1H), 7.16 (t, *J* = 7.4 Hz, 1H), 6.46 (s, 1H), 5.93 (s, 1H), 4.92 (d, *J* = 4.4 Hz, 1H), 3.63 – 3.51 (m, 1H), 2.84 (dd, *J* = 9.3, 7.6 Hz, 1H), 2.74 (dd, *J* = 9.3, 7.7 Hz, 1H), 2.50 (s, 3H), 2.47 (dd, *J* = 7.4, 1.8 Hz, 1H), 2.28 (dd, *J* = 7.5, 1.6 Hz, 1H), 2.19 (s, 3H).

^{13}C NMR (151 MHz, $CDCl_3$) δ 201.7, 171.6, 157.1, 154.8, 152.5, 144.0, 139.4, 128.7, 128.3, 127.3, 126.0, 123.8, 122.6, 120.6, 111.0, 110.7, 103.4, 61.7, 54.8, 52.6, 45.9, 45.5, 44.6, 14.2, 13.9.

HPLC: Chiral IA-3 column, (n-hexane/i-PrOH = 90:10), flow rate1 mL/min, I = 250 nm; $t_{Renten-major}$ = 7.17 min, $t_{Renten-minor}$ = 16.09 min.

HRMS (ESI): $[M+H]^+$ calcd for $[C_{27}H_{25}N_2O_3]$: 425.1860, Found: 425.1861.

(2R,3S)-4-(3,5-dimethyl-1H-pyrazole-1-carbonyl)-3-(naphthalen-2-yl)-1-(p-tolyl) bicyclo[2.1.1]hexane-2-carbaldehyde (3zd)

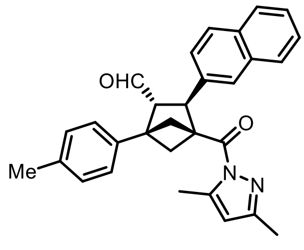

The product was purified by flash column chromatography on silica gel (PE/EA = 100:3-100:6) as a yellow molten oil at room temperature, 42% yield. R_f = 0.42 (PE/EA = 10:1). $[\alpha]_D^{22}$ = +18 (*c 1.0 (10mg/mL)*, CH_2Cl_2, 98% ee).

^{1}H NMR (600 MHz, $CDCl_3$) δ 9.84 (d, *J* = 2.4 Hz, 1H), 7.76 (d, *J* = 8.7 Hz, 2H), 7.71 (d, *J* = 8.8 Hz, 2H), 7.47 – 7.39 (m, 2H), 7.22 (dd, *J* = 8.5, 1.3 Hz, 1H), 7.17 (s, 4H),

5.91 (s, 1H), 4.98 (d, *J* = 4.2 Hz, 1H), 3.50 – 3.39 (m, 1H), 2.92 (dd, *J* = 9.1, 7.7 Hz, 1H), 2.77 – 2.71 (m, 1H), 2.51 (dd, *J* = 7.4, 1.7 Hz, 1H), 2.42 (s, 3H), 2.35 (s, 3H), 2.30 (dd, *J* = 7.5, 1.0 Hz, 1H), 2.25 (s, 3H).

^{13}C NMR (151 MHz, $CDCl_3$) δ 203.0, 172.5, 152.4, 144.0, 138.1, 136.8, 136.7, 133.3, 132.2, 129.4, 128.2, 127.8, 127.5, 126.4, 126.1, 125.9, 125.7, 125.3, 110.7, 63.8, 55.4, 52.3, 50.4, 47.0, 44.8, 21.1, 14.2, 14.0.

HPLC: Chiral IF-3 column, (n-hexane/i-PrOH = 98:2), flow rate1 mL/min, I = 250 nm; $t_{Renten\text{-}major}$ = 11.48 min, $t_{Renten\text{-}minor}$ = 13.80 min.

HRMS (ESI): $[M+H]^+$ calcd for $[C_{30}H_{29}N_2O_2]$: 449.2224, Found: 449.2226.

(2R,3S)-4-(3,5-dimethyl-1H-pyrazole-1-carbonyl)-1-(4-fluorophenyl)-3-(naphthalen-2-yl)bicyclo[2.1.1]hexane-2-carbaldehyde (3ze)

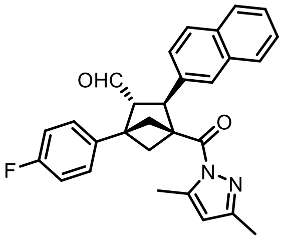

The product was purified by flash column chromatography on silica gel (PE/EA = 100:3-100:6) as a white molten oil at room temperature, 72% yield. R_f = 0.36 (PE/EA = 10:1). $[\alpha]_D^{22}$ = +32 (*c 1.0 (10mg/mL)*, CH_2Cl_2, 98% ee).

^{1}H NMR (600 MHz, $CDCl_3$) δ 9.85 (d, *J* = 2.4 Hz, 1H), 7.79 – 7.71 (m, 4H), 7.48 – 7.41 (m, 2H), 7.26 – 7.20 (m, 3H), 7.06 (t, *J* = 8.6 Hz, 2H), 5.92 (s, 1H), 4.99 (d, *J* = 4.5 Hz, 1H), 3.46 – 3.42 (m, 1H), 2.95 (dd, *J* = 9.1, 7.7 Hz, 1H), 2.78 – 2.72 (m, 1H), 2.51 (dd, *J* = 7.4, 1.7 Hz, 1H), 2.43 (s, 3H), 2.33 – 2.29 (m, 1H), 2.25 (s, 3H).

^{13}C NMR (151 MHz, $CDCl_3$) δ 202.6, 172.2, 162.7, 161.1, 152.5, 144.1, 137.8, 135.5, 135.5, 133.3, 132.3, 128.3, 127.8, 127.7, 127.6, 127.5, 126.3, 126.2, 125.8, 125.3, 115.7, 115.5, 110.8, 63.8, 55.4, 51.9, 50.5, 47.0, 44.9, 14.2, 14.0.

^{19}F NMR (565 MHz, $CDCl_3$) δ -115.02.

HPLC: Chiral IF-3 column, (n-hexane/i-PrOH = 98:2), flow rate1 mL/min, I = 250 nm; $t_{Renten\text{-}major}$ = 12.95 min, $t_{Renten\text{-}minor}$ = 15.50 min.

HRMS (ESI): $[M+H]^+$ calcd for $[C_{29}H_{26}FN_2O_2]$: 453.1973, Found: 453.1975.

(2R,3S)-1-(4-chlorophenyl)-4-(3,5-dimethyl-1H-pyrazole-1-carbonyl)-3-(naphthalen-2-yl)bicyclo[2.1.1]hexane-2-carbaldehyde (3zf)

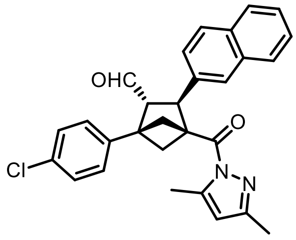

The product was purified by flash column chromatography on silica gel (PE/EA = 100:3-100:6) as a white molten oil at room temperature, 70% yield. R_f = 0.38 (PE/EA = 10:1). $[\alpha]_D^{22}$ = +42 (*c 1.0 (10mg/mL)*, CH_2Cl_2, 98% ee).

^{1}H NMR (600 MHz, $CDCl_3$) δ 9.84 (d, J = 2.3 Hz, 1H), 7.74 (m, 4H), 7.49 – 7.39 (m, 2H), 7.33 (d, J = 8.4 Hz, 2H), 7.21 (m, 3H), 5.92 (s, 1H), 4.98 (d, J = 4.3 Hz, 1H), 3.48 – 3.39 (m, 1H), 2.94 (dd, J = 9.0, 7.8 Hz, 1H), 2.77 – 2.71 (m, 1H), 2.50 (dd, J = 7.4, 1.6 Hz, 1H), 2.42 (s, 3H), 2.30 (d, J = 7.6 Hz, 1H), 2.24 (s, 3H).

^{13}C NMR (151 MHz, $CDCl_3$) δ 202.4, 172.1, 152.6, 144.1, 138.3, 137.8, 133.3, 133.0, 132.3, 128.9, 128.3, 127.8, 127.5, 127.5, 126.3, 126.2, 125.8, 125.3, 110.8, 63.7, 55.4, 51.9, 50.5, 46.9, 44.8, 14.2, 14.0.

HPLC: Chiral IF-3 column, (n-hexane/i-PrOH = 98:2), flow rate1 mL/min, I = 250 nm; $t_{Renten-major}$ = 13.59 min, $t_{Renten-minor}$ = 16.62 min.

HRMS (ESI): $[M+H]^+$ calcd for $[C_{29}H_{26}ClN_2O_2]$: 469.1677, Found: 469.1679.

(2R,3S)-4-(3,5-dimethyl-1H-pyrazole-1-carbonyl)-3-(naphthalen-2-yl)-1-(m-tolyl) bicyclo[2.1.1]hexane-2-carbaldehyde (3zg)

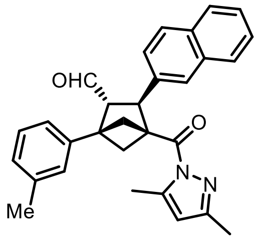

The product was purified by flash column chromatography on silica gel (PE/EA = 100:3-100:6) as a white molten oil at room temperature, 75% yield. R_f = 0.43 (PE/EA = 10:1). $[\alpha]_D^{22}$ = +29 (*c 1.0 (10mg/mL)*, CH_2Cl_2, 98% ee).

^{1}H NMR (600 MHz, CDCl3) δ 9.83 (d, J = 2.4 Hz, 1H), 7.81 – 7.67 (m, 4H), 7.43 (pd, J = 6.9, 1.5 Hz, 2H), 7.28 – 7.19 (m, 2H), 7.16 – 6.94 (m, 3H), 5.92 (s, 1H), 4.99 (d, J

= 5.1 Hz, 1H), 3.44 (dt, J = 4.7, 2.3 Hz, 1H), 2.97 – 2.89 (m, 1H), 2.80 – 2.72 (m, 1H), 2.52 (dd, J = 7.4, 2.2 Hz, 1H), 2.42 (s, 3H), 2.36 (s, 3H), 2.31 (dd, J = 7.5, 1.9 Hz, 1H), 2.25 (s, 3H).

^{13}C NMR (151 MHz, CDCl3) δ 203.0, 172.5, 152.5, 144.0, 139.7, 138.4, 138.1, 133.3, 132.2, 128.6, 128.2, 127.9, 127.8, 127.5, 126.7, 126.4, 126.1, 125.7, 125.3, 123.0, 110.7, 63.8, 55.3, 52.4, 50.3, 47.0, 44.7, 21.5, 14.2, 14.0.

HPLC: Chiral IF-3 column, (n-hexane/i-PrOH = 98:2), flow rate1 mL/min, I = 250 nm; $t_{Renten\text{-}major}$ = 10.07 min, $t_{Renten\text{-}minor}$ = 11.65 min.

HRMS (ESI): $[M+H]^+$ calcd for $[C_{30}H_{29}N_2O_2]$: 449.2224, Found: 449.2226.

(2R,3S)-4-(3,5-dimethyl-1H-pyrazole-1-carbonyl)-1-(3-methoxyphenyl)-3-(naphthalen-2-yl)bicyclo[2.1.1]hexane-2-carbaldehyde (3zh)

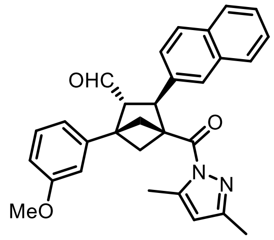

The product was purified by flash column chromatography on silica gel (PE/EA = 100:3-100:6) as a white molten oil at room temperature, 84% yield. R_f = 0.23 (PE/EA = 10:1). $[\alpha]_D^{22}$ = +58 (*c 1.0 (10mg/mL)*, CH_2Cl_2, 98% ee).

^{1}H NMR (600 MHz, $CDCl_3$) δ 9.85 (d, *J* = 2.3 Hz, 1H), 7.75 (m, 4H), 7.48 – 7.40 (m, 2H), 7.29 (t, *J* = 8.0 Hz, 1H), 7.23 (dd, *J* = 8.5, 1.5 Hz, 1H), 6.88 (d, *J* = 7.6 Hz, 1H), 6.84 – 6.80 (m, 2H), 5.92 (s, 1H), 5.00 (d, *J* = 4.4 Hz, 1H), 3.82 (s, 3H), 3.47 – 3.42 (m, 1H), 2.93 (dd, *J* = 9.1, 7.6 Hz, 1H), 2.79 – 2.74 (m, 1H), 2.52 (dd, *J* = 7.3, 1.7 Hz, 1H), 2.43 (s, 3H), 2.34 (dd, *J* = 7.5, 1.2 Hz, 1H), 2.26 (s, 3H).

^{13}C NMR (151 MHz, $CDCl_3$) δ 202.7, 172.4, 159.9, 152.5, 144.0, 141.4, 138.1, 133.3, 132.2, 129.8, 128.3, 127.8, 127.5, 126.4, 126.1, 125.7, 125.3, 118.3, 112.4, 111.9, 110.7, 63.8, 55.3, 55.2, 52.4, 50.3, 46.9, 44.8, 14.2, 14.0.

HPLC: Chiral IF-3 column, (n-hexane/i-PrOH = 90:10), flow rate1 mL/min, I = 250 nm; $t_{Renten\text{-}major}$ = 8.61 min, $t_{Renten\text{-}minor}$ = 10.45 min.

HRMS (ESI): $[M+H]^+$ calcd for $[C_{30}H_{29}N_2O_3]$: 465.2173, Found: 465.2173.

(2R,3S)-4-(3,5-dimethyl-1H-pyrazole-1-carbonyl)-1-(3-fluorophenyl)-3-(naphthalen-2-yl)bicyclo[2.1.1]hexane-2-carbaldehyde (3zi)

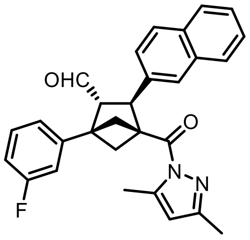

The product was purified by flash column chromatography on silica gel (PE/EA = 100:3-100:6) as a white molten oil at room temperature, 53% yield. R_f = 0.38 (PE/EA = 10:1). $[\alpha]_D^{22}$ = +14 (*c 1.0 (10mg/mL)*, CH_2Cl_2, 98% ee).

^{1}H NMR (600 MHz, $CDCl_3$) δ 9.84 (d, J = 2.4 Hz, 1H), 7.86 – 7.65 (m, 4H), 7.50 – 7.39 (m, 2H), 7.33 (dd, J = 13.8, 7.7 Hz, 1H), 7.21 (dd, J = 8.6, 1.5 Hz, 1H), 7.06 (d, J = 7.7 Hz, 1H), 6.97 (m, 2H), 5.92 (s, 1H), 4.98 (d, J = 4.4 Hz, 1H), 3.45 (dt, J = 4.5, 2.1 Hz, 1H), 2.94 (dd, J = 9.3, 7.5 Hz, 1H), 2.75 (dd, J = 9.2, 7.8 Hz, 1H), 2.52 (dd, J = 7.4, 1.9 Hz, 1H), 2.42 (s, 3H), 2.32 (dd, J = 7.6, 1.5 Hz, 1H), 2.25 (s, 3H).

^{13}C NMR (151 MHz, $CDCl_3$) δ 202.3, 172.1, 163.8, 162.2, 152.6, 144.1, 142.4, 142.4, 137.7, 133.3, 132.3, 130.4, 130.3, 128.3, 127.8, 127.5, 126.3, 126.2, 125.8, 125.3, 121.7, 121.7, 114.3, 114.1, 113.3, 113.1, 110.8, 63.7, 55.4, 52.1, 50.4, 46.9, 44.8, 14.2, 14.0.

^{19}F NMR (565 MHz, $CDCl_3$) δ -112.34.

HPLC: Chiral IF-3 column, (n-hexane/i-PrOH = 98:2), flow rate1 mL/min, I = 250 nm; $t_{Renten\text{-}major}$ = 12.07 min, $t_{Renten\text{-}minor}$ = 14.13 min.

HRMS (ESI): $[M+H]^+$ calcd for $[C_{29}H_{26}FN_2O_2]$: 453.1973, Found: 453.1975.

3.4 Unsuccessful substrates

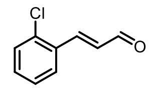

1za <5% y, n.d.

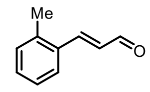

1zb <5% y, n.d.

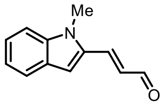

1zc <5% y, n.d.

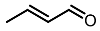

1zd <5% y, n.d.

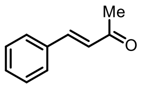

1zf n.p.

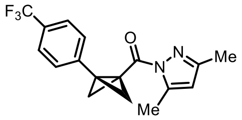

2e <5% y, n.d.

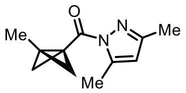

2i <5% y, n.d.

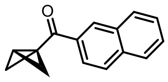

2j <5% y, n.d.

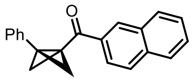

2k <10% y, n.d.

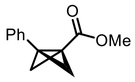

2l <10% y, n.d.

Figure S4. Unsuccessful substrates.

4. Mechanistic Investigations

4.1 Mechanistic Study

To a 10 mL Schlenk tube equipped with a magnetic stir bar was added BCBs **2a** (0.15 mmol) and TFA (0.04 mmol), then acetone (2 mL) was added. The resulting mixture was stirred at 10 °C for 24 h. Upon completion of the reaction, the reaction mixture was concentrated under reduced pressure, and the resulting crude mixture was purified by silica gel column chromatography (PE/EA = 50:1) to afford **27**.

(3,5-dimethyl-1H-pyrazol-1-yl)(3-phenylcyclobut-2-en-1-yl)methanone (27)

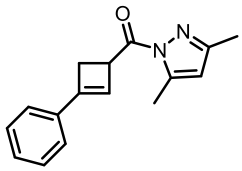

^{1}H NMR (600 MHz, CDCl3) δ 7.39 (d, J = 7.1 Hz, 2H), 7.34 (t, J = 7.4 Hz, 2H), 7.31 – 7.24 (m, 1H), 6.43 (s, 1H), 5.97 (s, 1H), 4.71 (s, 1H), 3.21 – 3.12 (m, 2H), 2.55 (s, 3H), 2.27 (s, 3H).

^{13}C NMR (151 MHz, CDCl3) δ 173.4, 152.1, 148.1, 144.0, 133.9, 128.3, 128.2, 125.3, 124.7, 110.9, 42.7, 32.4, 14.4, 13.9.

HRMS (ESI): $[M+H]^+$ calcd for $[C_{16}H_{16}N_2O]$: 253.1335, Found: 253.1330.

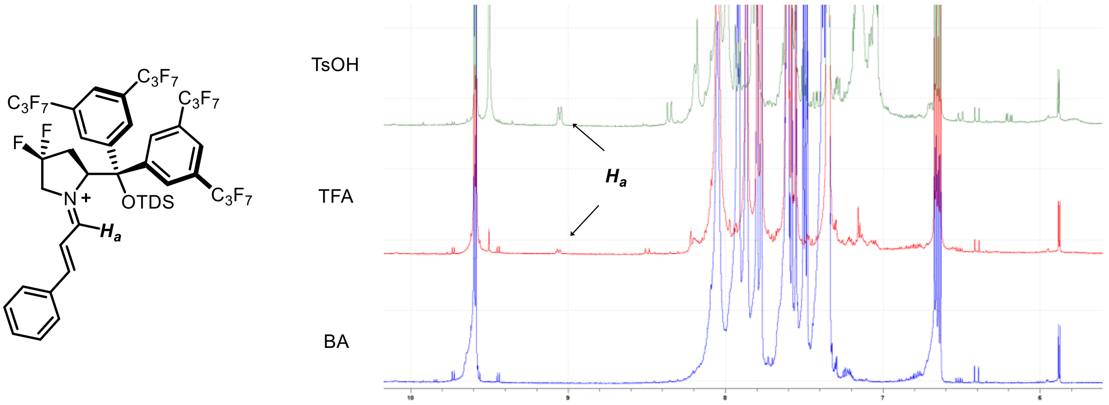

Figure S5. The spectra are for mixed solutions of cinnamic aldehyde/chiral amine/acid = (1:1:1.3) in acetone-d6.

4.2 DFT calculations

All geometric optimizations have been carried out by density functional theory using the M062X functional[9,10] with Grimme's dispersion (D3)[11] using the Gaussian 16 program[12]. The standard 6-31G(d,p) basis set[13,14] was used for structural optimization. Frequency calculations at the same level of theory have also been performed to identify all stationary points as minima (zero imaginary frequencies), the transition state structure has and has only one imaginary frequency, and the single point energy calculation is performed under the def2tzvp basis set. The temperature used in all structural calculations is 283.15 K, and the solvent Acetone is added to all calculation under the IEFPCM[15] model.

Table S2. Electrophilic coefficient at the double bond position

	q(N)	q(N+1)	q(N-1)	ω
C1	0.0189	-0.0860	0.0392	0.32397
C1'(TFA^-)	0.0234	-0.0743	0.0289	0.34432

The Fukui function is calculated at the B3LYP/6-31G* level. The results are shown in Table S2. After the addition of TFA^-, the ω(condensed local electrophilicity index) of the C1 of the imine derivative was significantly increased, indicating that the electrophilic ability of the site became stronger.

4.3 Plausible Mechanism

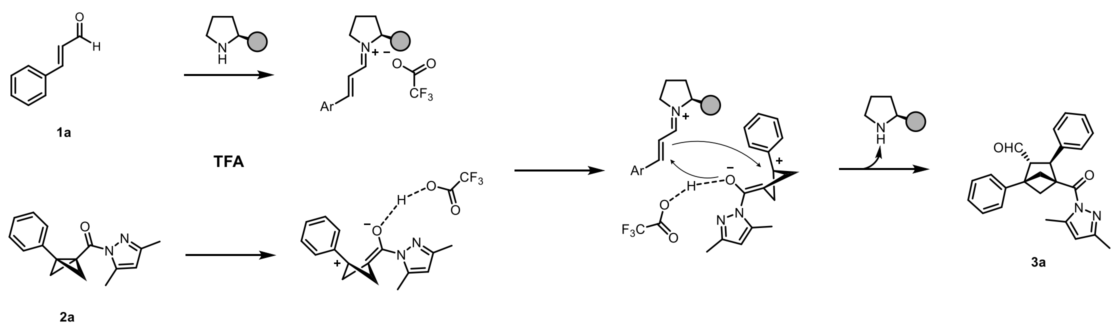

Figure S6. Plausible pathway for product **3a**

5. Synthetic Application

5.1 Scale-up Synthesis

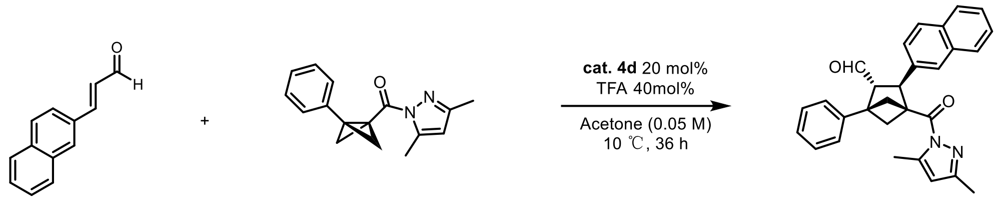

1s 2a 3s

To a 100 mL round bottom flask equipped with a magnetic stir bar was added **cat. 4d** (0.6 mmol, 0.2 eq.), aldehydes **1s** (3 mmol, 1.0 eq.), BCBs **2a** (4.5 mmol, 1.5 eq.) and TFA (1.2 mmol, 0.4 eq.), then acetone (60 mL) was added. The resulting mixture was stirred at 10 °C for 36 h. Upon completion of the reaction, the reaction mixture was concentrated under reduced pressure, and the resulting crude mixture was purified by silica gel column chromatography (PE/EA = 100:3-100:6) to afford the pure product **3s** (2.1 mmol, 70% yield) with 98% ee.

5.2 Derivatization of the Cycloadducts

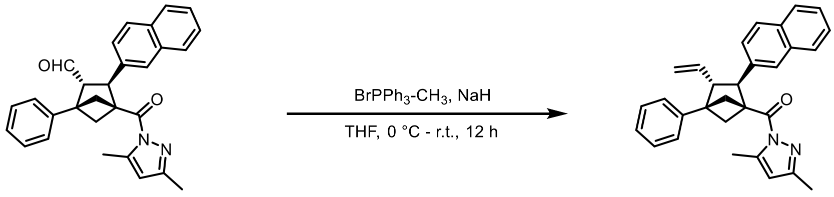

3s 6

Under Ar, to a flame-dried vessel was added methyltriphenylphosphonium bromide (72 mg, 0.20 mmol) and dry THF (1 mL). NaH (8.0 mg, 0.20 mmol, 60% oil dispersion) was added to the mixture at 0 °C. After the mixture was stirred at 0 °C for 4 h, a solution of **3s** (0.1 M in THF, 1.0 mL, 0.1 mmol) was added. Then the mixture was stirred at room temperature for 12 h (mornitored by TLC). Untill full conversion of **3s**, the reaction was quenched by saturated aq NH_4Cl and extracted with diethyl

ether for three times. The combined organic layers were washed with brine, dried over Na_2SO_4, filtered and concentrated under vacuum. The crude residue was purified by silica gel column chromatography (PE/EA = 30:1, v/v) to afford the corresponding alkene **6** (28.1 mg, 65% yield, 98% ee). R_f = 0.60 (PE/EA = 10:1).

(3,5-dimethyl-1H-pyrazol-1-yl)((2S,3R)-2-(naphthalen-2-yl)-4-phenyl-3-vinylbicyclo[2.1.1]hexan-1-yl)methanone (6)

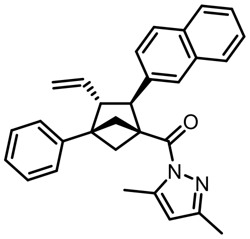

^{1}H NMR (600 MHz, CDCl3) δ 7.81 – 7.76 (m, 3H), 7.74 – 7.69 (m, 1H), 7.48 – 7.37 (m, 2H), 7.37 – 7.28 (m, 3H), 7.25 – 7.18 (m, 3H), 6.09 – 5.98 (m, 1H), 5.90 (s, 1H), 5.11 – 5.01 (m, 2H), 4.45 (d, J = 3.6 Hz, 1H), 3.15 (m, 1H), 2.95 – 2.88 (m, 1H), 2.72 (m, 1H), 2.49 – 2.40 (m, 4H), 2.30 (dd, J = 7.4, 2.0 Hz, 1H), 2.25 (s, 3H).

^{13}C NMR (151 MHz, CDCl3) δ 173.4, 152.0, 144.0, 141.4, 139.5, 139.0, 133.4, 132.1, 128.1, 127.9, 127.8, 127.4, 126.5, 126.3, 126.3, 125.9, 125.4, 124.9, 116.8, 110.5, 58.1, 55.7, 55.5, 53.0, 46.9, 43.9, 14.3, 14.0.

HPLC: Chiral IF-3 column, (n-hexane/i-PrOH = 98:2), flow rate1 mL/min, I = 250 nm; $t_{Renten\text{-}major}$ = 5.70 min, $t_{Renten\text{-}minor}$ = 5.19 min.

HRMS (ESI): $[M+H]^+$ calcd for $[C_{30}H_{29}N_2O]$: 433.2274, Found: 433.2273.

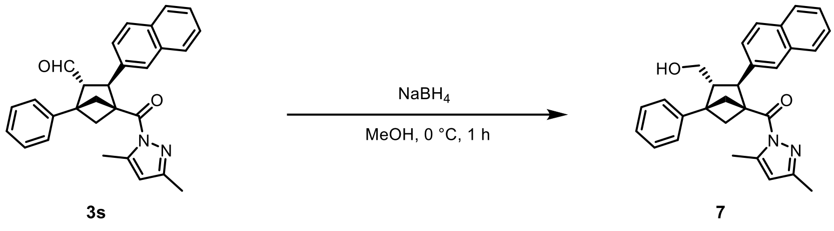

To a suspension of $NaBH_4$ (3.8 mg, 0.1 mmol) in MeOH (1 mL) was added **3s** (43 mg, 0.1 mmol) at 0 °C. Then, the reaction mixture was stirred at 0 °C. After a full

conversion of **3s** was detected by TLC (about 1 hour), the reaction was quenched by saturated NH_4Cl and extracted with EA for three times. The combined organic layers were washed with brine, dried over Na_2SO_4, filtered and concentrated under vacuum. The crude residue was purified by silica gel column chromatography (PE/EA = 7:1, v/v) to afford the corresponding alcohol **7** (37.4 mg, 86% yield, 98% ee). R_f = 0.37 (PE/EA = 3:1).

(3,5-dimethyl-1H-pyrazol-1-yl)((2S,3R)-3-hydroxy-2-(naphthalen-2-yl)-4-phenyl bicyclo[2.1.1]hexan-1-yl)methanone (7)

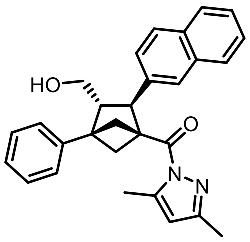

^{1}H NMR (600 MHz, $CDCl_3$) δ 7.82 – 7.72 (m, 3H), 7.69 (d, J = 8.5 Hz, 1H), 7.45 – 7.39 (m, 2H), 7.37 (t, J = 7.5 Hz, 2H), 7.31 – 7.22 (m, 4H), 5.90 (s, 1H), 4.20 (d, J = 5.3 Hz, 1H), 3.92 (dd, J = 11.4, 6.9 Hz, 1H), 3.82 (dd, J = 11.4, 6.3 Hz, 1H), 2.95 – 2.86 (m, 2H), 2.74 (dd, J = 9.4, 7.3 Hz, 1H), 2.41 (dd, J = 7.0, 1.9 Hz, 1H), 2.36 (s, 3H), 2.25 (s, 3H), 2.18 (dd, J = 7.3, 1.8 Hz, 1H).

^{13}C NMR (151 MHz, $CDCl_3$) δ 172.9, 152.1, 144.0, 141.7, 138.8, 133.4, 132.3, 128.7, 127.9, 127.8, 127.4, 126.8, 126.6, 125.9, 125.7, 125.5, 110.5, 65.0, 56.2, 54.9, 54.0, 51.2, 46.5, 44.2, 14.2, 14.0.

HPLC: Chiral IF-3 column, (n-hexane/i-PrOH = 90:10), flow rate1 mL/min, I = 250 nm; $t_{Renten\text{-}major}$ = 13.15min, $t_{Renten\text{-}minor}$ = 11.27 min.

HRMS (ESI): $[M+H]^+$ calcd for $[C_{29}H_{29}N_2O_2]$: 437.2224, Found: 437.2223.

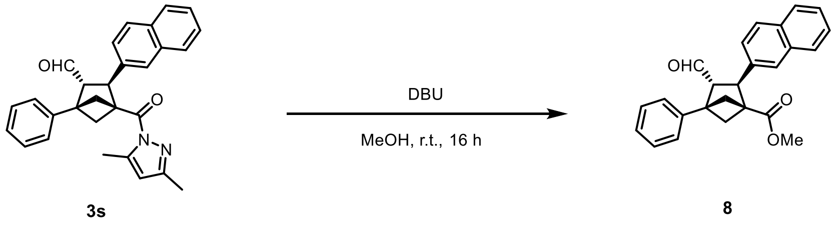

3s (43 mg, 0.1 mmol) was dissolved in MeOH (1 mL) and DBU (17 mg, 0.11 mmol) was added. The resulted mixture was stirred for 16 h and then concentrated. The crude residue was purified by silica gel column chromatography (PE/EA = 10:1, v/v) to afford the corresponding ester **8** (36.5 mg, 99% yield, 98% ee). R_f = 0.32 (PE/EA = 10:1).

Methyl (2S,3R)-3-formyl-2-(naphthalen-2-yl)-4-phenylbicyclo[2.1.1]hexane-1-carboxylate (8)

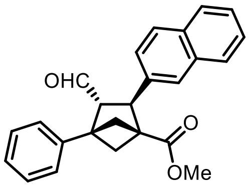

^{1}H NMR (600 MHz, $CDCl_3$) δ 9.77 (d, J = 1.8 Hz, 1H), 7.93 – 7.75 (m, 3H), 7.69 (d, J = 2.0 Hz, 1H), 7.53 – 7.44 (m, 2H), 7.41 – 7.33 (m, 3H), 7.31 – 7.27 (m, 1H), 7.23 (dd, J = 8.3, 1.4 Hz, 2H), 4.46 (dd, J = 4.9, 2.2 Hz, 1H), 3.67 (s, 3H), 3.37 (dt, J = 3.8, 1.9 Hz, 1H), 2.65 (dd, J = 9.4, 7.4 Hz, 1H), 2.45 (dd, J = 9.4, 7.1 Hz, 1H), 2.35 (td, J = 7.5, 2.0 Hz, 2H).

^{13}C NMR (151 MHz, $CDCl_3$) δ 202.6, 172.3, 139.3, 138.1, 133.3, 132.2, 128.7, 128.4, 127.8, 127.5, 127.3, 126.4, 126.2, 125.8, 125.8, 125.0, 64.1, 53.4, 51.8, 51.3, 48.1, 44.7, 44.3.

HPLC: Chiral IF-3 column, (n-hexane/i-PrOH = 90:10), flow rate1 mL/min, I = 250 nm; $t_{Renten-major}$ = 10.99min, $t_{Renten-minor}$ = 12.61 min.

HRMS (ESI): $[M+H]^+$ calcd for $[C_{25}H_{23}O_3]$: 371.1642, Found: 371.1644.

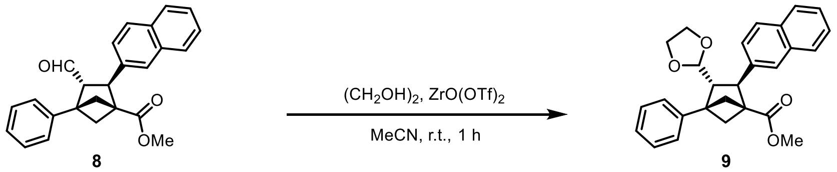

8 (370 mg, 1 mmol) was dissolved in MeCN (5 mL), $(CH_2OH)_2$ (310 mg, 5 mmol) and $ZrO(OTf)_2$ (40 mg, 0.1mmol) were added. The resulted mixture was stirred for 1

h and then concentrated. The crude residue was purified by silica gel column chromatography (PE/EA = 10:1, v/v) to afford the corresponding acetal **9** (410 mg, 99% yield, 93% ee). R_f = 0.28 (PE/EA = 10:1).

Methyl (2S,3R)-3-(1,3-dioxolan-2-yl)-2-(naphthalen-2-yl)-4-phenylbicyclo[2.1.1] hexane-1-carboxylate (9)

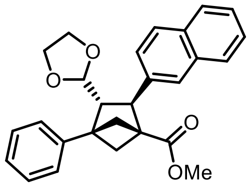

^{1}H NMR (600 MHz, $CDCl_3$) δ 7.86 – 7.74 (m, 4H), 7.63 – 7.58 (m, 1H), 7.49 – 7.40 (m, 2H), 7.36 – 7.31 (m, 2H), 7.26 – 7.20 (m, 3H), 4.81 (m, 1H), 4.09 (dd, J = 4.9, 2.0 Hz, 1H), 4.03 (q, J = 6.7 Hz, 1H), 3.95 (q, J = 6.4 Hz, 1H), 3.88 – 3.83 (m, 1H), 3.72 (q, J = 7.4 Hz, 1H), 3.62 (s, 3H), 2.92 – 2.80 (m, 2H), 2.56 (m, 1H), 2.17 (m, 2H).

^{13}C NMR (151 MHz, $CDCl_3$) δ 173.2, 141.0, 139.6, 133.4, 132.2, 128.3, 127.9, 127.8, 127.4, 127.4, 126.5, 126.0, 125.8, 125.5, 125.4, 103.6, 65.2, 64.4, 54.4, 51.9, 51.8, 51.6, 48.0, 44.6, 44.4.

HPLC: Chiral IC-3 column, (n-hexane/i-PrOH = 90:10), flow rate1 mL/min, I = 250 nm; $t_{Renten\text{-}major}$ = 9.28 min, $t_{Renten\text{-}minor}$ = 16.37 min.

HRMS (ESI): $[M+Na]^+$ calcd for $[C_{27}H_{26}O_4Na]$: 437.1723, Found: 437.1716.

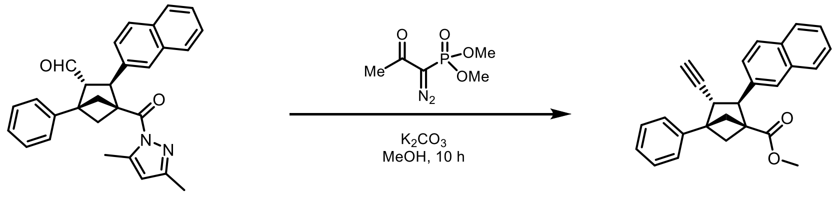

3s **10**

To a 50 mL round bottom flask equipped with a magnetic stir bar was added **3s** (1 mmol, 440 mg), Dimethyl (1-Diazo-2-oxopropyl)phosphonate (1.2 mmol, 230 mg), K_2CO_3 (2 mmol, 300 mg) under Ar, then MeOH (10 mL) was added. The resulting mixture was stirred for 10 h and then concentrated. The crude residue was purified by

silica gel column chromatography (PE/EA = 10:1, v/v) to afford the corresponding alkyne **10** (352 mg, 96% yield, 96% ee). R_f = 0.52 (PE/EA = 10:1).

Methyl (2S,3S)-3-ethynyl-2-(naphthalen-2-yl)-4-phenylbicyclo[2.1.1]hexane-1-carboxylate (10)

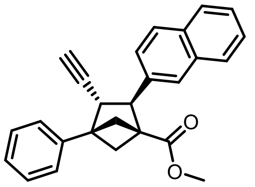

^{1}H NMR (600 MHz, $CDCl_3$) δ 7.85 – 7.79 (m, 3H), 7.69 (s, 1H), 7.52 – 7.42 (m, 3H), 7.34 (t, J = 7.7 Hz, 2H), 7.30 – 7.23 (m, 3H), 4.10 (dd, J = 5.0, 1.5 Hz, 1H), 3.65 (s, 3H), 3.13 (dt, J = 4.7, 2.4 Hz, 1H), 2.64 (dd, J = 9.4, 6.7 Hz, 1H), 2.43 (dd, J = 9.5, 7.5 Hz, 1H), 2.32 (tt, J = 4.5, 2.4 Hz, 2H), 2.25 (d, J = 2.5 Hz, 1H).

^{13}C NMR (151 MHz, $CDCl_3$) δ 172.4, 139.8, 138.3, 133.3, 132.3, 128.3, 128.2, 127.9, 127.5, 127.0, 126.3, 126.2, 125.8, 124.6, 85.2, 72.1, 56.4, 53.9, 51.8, 51.8, 46.5, 45.3, 41.8.

HPLC: Chiral IF-3 column, (n-hexane/i-PrOH = 99:1), flow rate1 mL/min, I = 250 nm; $t_{Renten\text{-}major}$ = 17.27 min, $t_{Renten\text{-}minor}$ = 15.48 min.

HRMS (ESI): $[M+Na]^+$ calcd for $[C_{26}H_{22}O_2Na]$: 389.1512, Found: 389.1504.

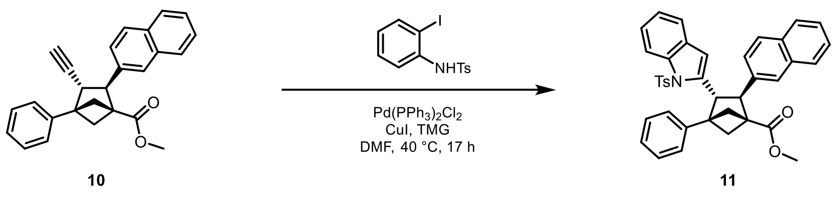

A DMF (0.1 mL) solution of N-(2-iodophenyl)-4-methylbenzenesulfonamide (37.5 mg, 0.1 mmol), **10** (36.6 mg, 0.1 mmol), $Pd(PPh_3)_2Cl_2$ (3.5 mg, 0.005 mmol), CuI (1 mg, 0.005 mmol,), and tetramethylguanidine (TMG, 34.5 mg, 0.3 mmol, 3 equiv) was stirred at 40 °C for 17 h. After being diluted with water, the mixture was extracted with EA. The organic phase was concentrated under reduced pressure and purified by silica gel column chromatography (PE/EA = 10:1, v/v) to afford **11** (56 mg, 92% yield, 94% ee). R_f = 0.2 (PE/EA = 10:1).

Methyl (2S,3R)-2-(naphthalen-2-yl)-4-phenyl-3-(1-tosyl-1H-indol-2-yl)bicyclo[2.1.1]hexane-1-carboxylate (11)

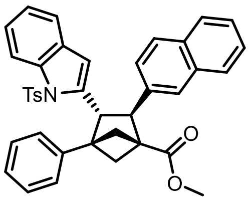

^{1}H NMR (600 MHz, $CDCl_3$) δ 7.98 – 7.93 (m, 1H), 7.86 – 7.80 (m, 1H), 7.78 – 7.73 (m, 3H), 7.54 – 7.50 (m, 1H), 7.50 – 7.45 (m, 2H), 7.44 (dd, J = 8.5, 1.8 Hz, 1H), 7.24 – 7.20 (m, 2H), 7.20 – 7.15 (m, 3H), 7.07 (s, 1H), 7.03 – 7.00 (m, 2H), 6.97 – 6.90 (m, 2H), 6.51 (d, J = 8.4 Hz, 2H), 4.84 (dd, J = 5.8, 1.6 Hz, 1H), 4.21 (dd, J = 5.8, 2.0 Hz, 1H), 3.61 (s, 3H), 2.97 (dd, J = 9.6, 7.3 Hz, 1H), 2.80 (dd, J = 9.6, 7.3 Hz, 1H), 2.54 (dd, J = 7.4, 1.9 Hz, 1H), 2.23 (dd, J = 7.3, 1.6 Hz, 1H), 2.03 (s, 3H).

^{13}C NMR (151 MHz, $CDCl_3$) δ 172.6, 144.0, 142.7, 140.0, 138.5, 137.4, 135.3, 133.5, 132.5, 129.5, 129.2, 128.1, 128.0, 127.7, 127.5, 126.7, 126.1, 126.0, 126.0, 125.8, 125.7, 124.2, 123.6, 120.3, 115.5, 110.5, 56.4, 55.5, 51.9, 51.7, 51.0, 46.1, 43.1, 21.2.

HPLC: Chiral IF-3 column, (n-hexane/i-PrOH = 90:10), flow rate1 mL/min, I = 250 nm; $t_{Renten\text{-}major}$ = 10.10 min, $t_{Renten\text{-}minor}$ = 12.54 min.

HRMS (ESI): $[M+H]^+$ calcd for [$C_{39}H_{34}NO_4S$]: 612.2203, Found: 612.2191. $[M+Na]^+$ calcd for [$C_{39}H_{33}NO_4SNa$]: 634.2023, Found: 634.2011.

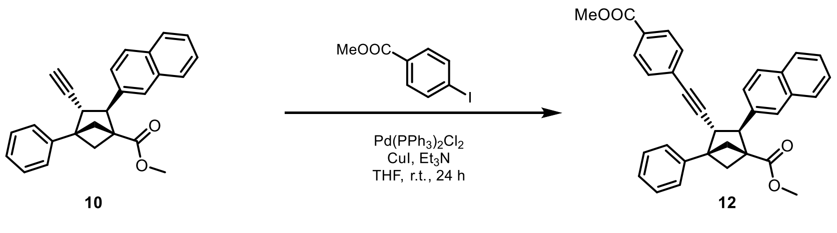

In a N_2-filled glovebox, to a THF (0.5 mL) solution of **10** (36.6 mg, 0.1 mmol) and 4 iodobenzoic acid methyl ester (26 mg, 0.1 mmol,) was added Et_3N (31 mg, 0.3 mmol,), $Pd(PPh_3)_2Cl_2$ (3.5 mg, 0.005 mmol), and CuI (1.9 mg, 0.01 mmo). The reaction was stirred at r.t. for 24 h, and then quenched with saturated aq. NH_4Cl solution (3 mL). The mixture was extracted with EA (3 mL×3). The organic phase

was concentrated under reduced pressure and purified by silica gel column chromatography (PE/EA = 10:1, v/v) to afford **12** (39.5 mg, 79% yield, 93% ee). R_f = 0.3 (PE/EA = 10:1).

Methyl (2S,3S)-3-((4-(methoxycarbonyl)phenyl)ethynyl)-2-(naphthalen-2-yl)-4-phenylbicyclo[2.1.1]hexane-1-carboxylate (12)

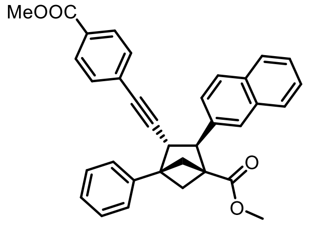

^{1}H NMR (600 MHz, $CDCl_3$) δ 7.98 – 7.92 (m, 2H), 7.83 (t, J = 8.2 Hz, 3H), 7.73 (s, 1H), 7.53 (dd, J = 8.5, 2.0 Hz, 1H), 7.47 (pd, J = 6.8, 1.5 Hz, 2H), 7.41 (d, J = 8.4 Hz, 2H), 7.38 – 7.30 (m, 4H), 7.30 – 7.26 (m, 1H), 4.18 (dd, J = 5.0, 1.5 Hz, 1H), 3.91 (s, 3H), 3.67 (s, 3H), 3.36 (dd, J = 4.6, 2.3 Hz, 1H), 2.72 (dd, J = 9.5, 6.8 Hz, 1H), 2.52 (dd, J = 9.4, 7.6 Hz, 1H), 2.38 (dd, J = 7.6, 2.2 Hz, 1H), 2.35 (dd, J = 6.9, 2.4 Hz, 1H).

^{13}C NMR (151 MHz, $CDCl_3$) δ 172.4, 166.6, 140.0, 138.2, 133.4, 132.4, 131.5, 129.4, 129.2, 128.4, 128.3, 128.2, 127.9, 127.6, 127.0, 126.4, 126.2, 126.2, 125.8, 124.8, 94.2, 83.6, 56.6, 54.3, 52.2, 52.0, 51.8, 46.9, 46.2, 41.5.

HPLC: Chiral IF-3 column, (n-hexane/i-PrOH = 90:10), flow rate1 mL/min, I = 250 nm; $t_{Renten\text{-}major}$ = 9.64 min, $t_{Renten\text{-}minor}$ = 7.66 min.

HRMS (ESI): $[M+H]^+$ calcd for $[C_{34}H_{29}O_4]$: 501.2060, Found: 501.2056. $[M+Na]^+$ calcd for $[C_{34}H_{28}O_4Na]$: 523.1880, Found: 523.1874.

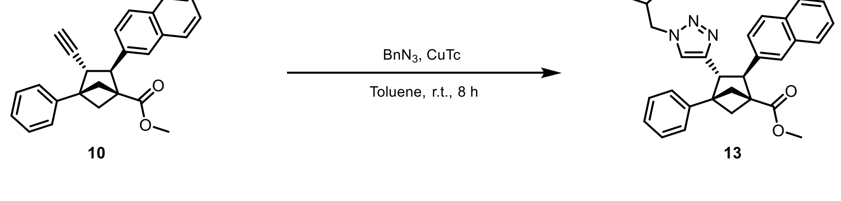

Under argon atmosphere, a flame-dried Schlenk tube was charged with **10** (36.6 mg, 0.1 mmol), copper(I) thiophene-2-carboxylate (CuTc, 3.8 mg, 0.02 mmol) and

anhydrous toluene (1 mL). Subsequently, benzyl azide (15 uL, 0.12mmol,) was added slowly, then the reaction mixture was stirred for 8 h. The reaction was quenched by saturated NH_4Cl aqueous solution (3 mL) and extracted with EA (3 mL x 3). The combined organic layers were dried over Na_2SO_4, filtrated and concentrated in vacuo. The crude residue was purified by silica gel column chromatography (DCM/EA = 10:1, v/v) to afford **13** (42.5 mg, 85% yield, 93% ee). R_f = 0.81 (DCM/EA = 10:1).

Methyl (2S,3R)-3-(1-benzyl-1H-1,2,3-triazol-4-yl)-2-(naphthalen-2-yl)-4-phenylbicyclo[2.1.1]hexane-1-carboxylate (13)

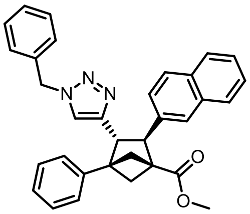

^{1}H NMR (600 MHz, $CDCl_3$) δ 7.91 – 7.68 (m, 4H), 7.59 – 7.37 (m, 3H), 7.32 (dd, J = 5.0, 1.9 Hz, 3H), 7.15 (dd, J = 5.3, 2.0 Hz, 3H), 7.07 – 6.97 (m, 2H), 7.00 – 6.81 (m, 2H), 6.66 (s, 1H), 5.52 – 5.30 (m, 2H), 4.86 (dd, J = 4.9, 2.0 Hz, 1H), 3.66 (s, 3H), 3.60 (dd, J = 4.9, 2.1 Hz, 1H), 2.93 (dd, J = 9.4, 6.7 Hz, 1H), 2.72 (dd, J = 9.4, 7.4 Hz, 1H), 2.41 (dd, J = 7.4, 1.9 Hz, 1H), 2.25 (dd, J = 6.7, 1.9 Hz, 1H).

^{13}C NMR (151 MHz, $CDCl_3$) δ 172.9, 140.6, 139.4, 134.8, 133.4, 132.1, 128.9, 128.4, 128.0, 128.0, 127.8, 127.5, 127.4, 126.6, 126.4, 125.9, 125.9, 125.5, 124.7, 54.6, 53.7, 52.7, 51.7, 51.6, 51.4, 45.0, 43.2.

HPLC: Chiral IF-3 column, (n-hexane/i-PrOH = 70:30), flow rate1 mL/min, I = 250 nm; $t_{Renten\text{-}major}$ = 20.78 min, $t_{Renten\text{-}minor}$ = 14.97 min.

HRMS (ESI): $[M+H]^+$ calcd for $[C_{33}H_{30}N_3O_2]$: 500.2333, Found: 500.2326. $[M+Na]^+$ calcd for $[C_{33}H_{29}N_3O_2Na]$: 522.2152, Found: 522.2147.

5.3 Synthesis of Bio-relevant BCHs

Synthesis of starting material carboxylic acid

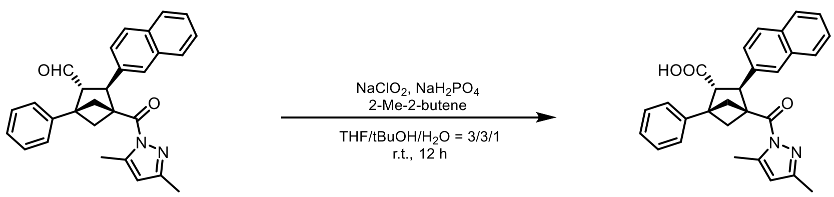

3s **14**

To a 50 mL round bottom flask equipped with a magnetic stir bar was added **3s** (1.0 eq.), $NaClO_2$ (3.0 eq.), NaH_2PO_4 (3.0 eq.) and 2-Me-2-butene (8.0 eq.), then solvent (THF/tBuOH/H_2O = 3/3/1, v/v, 0.1 M) was added. The resulting mixture was stirred for 10 h and then washed with brine. The organic layer was dried over Na_2SO_4, filtrated and concentrated in vacuo. The crude product **14** could be directly used in the next step without further purification.

General Procedure A for the Synthesis of Bio-relevant BCHs

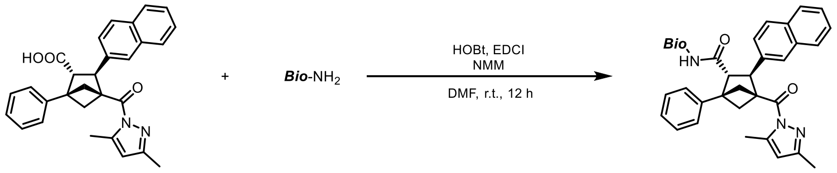

To a 5 mL vial equipped with a magnetic stir bar was added carboxylic acid (1.0 eq.), amine (1.0 eq.), Hydroxybenzotriazole (HOBt) (1.1 eq.), and 1-(3-Dimethylaminopropyl)-3-ethylcarbodiimide hydrochloride (EDCI) (1.2 equiv.). DMF (0.05 M) and 4-Methylmorpholine (NMM) (2.0 eq.) were added and the reaction was stirred at r.t. for 12 h. Afterward, brine (3 mL) was added and the reaction mixture was extracted with EA (3 mL×3). The combined organic layer was washed with brine, dried over Na_2SO_4, filtered, concentrated, and purified by flash column chromatography to yield the desired product.

General Procedure B for the Synthesis of Bio-relevant BCHs

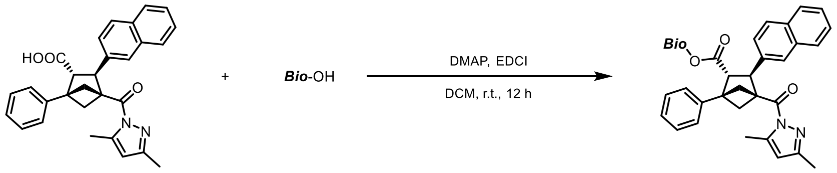

To a 5 mL vial equipped with a magnetic stir bar was added carboxylic acid (1.0 eq.), alcohol (1.0 eq.), 4-Dimethylaminopyridine (DMAP) (0.2 eq.), and 1-(3-Dimethylaminopropyl)-3-ethylcarbodiimide hydrochloride (EDCI) (2.0 equiv.). DCM (0.1 M) was added and the reaction was stirred at r.t. for 12 h. Afterward, saturated NH_4Cl aqueous solution (3 mL) was added and the reaction mixture was extracted with DCM (3 mL×3). The combined organic layer was washed with brine, dried over Na_2SO_4, filtered, concentrated, and purified by flash column chromatography to yield the desired product.

(E)-3,7-dimethylocta-2,6-dien-1-yl (2R,3S)-4-(3,5-dimethyl-1H-pyrazole-1-carbonyl)-3-(naphthalen-2-yl)-1-phenylbicyclo[2.1.1]hexane-2-carboxylate (15)

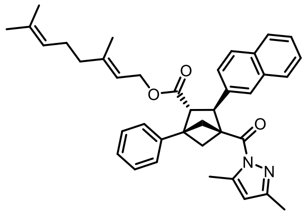

Synthesized according to the **General Procedure B** for the ssynthesis of Bio-relevant BCHs from carboxylic acid **14** (22.5 mg, 0.05 mmol), Geraniol (7.7 mg, 0.05 mmol), DMAP (1.3 mg, 0.01 mmol), EDCI (19 mg, 0.1mmol) and DCM (0.5 mL) to give the desired product **15** (20.8 mg, 71% yield). R_f = 0.58 (PE/EA = 10:1).

^{1}H NMR (600 MHz, $CDCl_3$) δ 7.75 (d, *J* = 7.8 Hz, 2H), 7.72 (s, 1H), 7.70 (d, *J* = 8.5 Hz, 1H), 7.46 – 7.40 (m, 2H), 7.32 (t, *J* = 7.5 Hz, 2H), 7.27 – 7.20 (m, 4H), 5.90 (s, 1H), 5.08 – 4.99 (m, 2H), 4.92 (d, *J* = 4.6 Hz, 1H), 4.51 – 4.42 (m, 2H), 3.46 (dd, *J* = 5.1, 1.6 Hz, 1H), 3.40 (dd, *J* = 9.4, 7.1 Hz, 1H), 2.71 (dd, *J* = 9.3, 7.7 Hz, 1H), 2.44 (dd, *J* = 7.0, 1.6 Hz, 1H), 2.39 (s, 3H), 2.29 (dd, *J* = 7.6, 1.6 Hz, 1H), 2.25 (s, 3H), 2.02 (dd, *J* = 15.3, 7.4 Hz, 2H), 1.97 – 1.91 (m, 2H), 1.66 (s, 3H), 1.58 (s, 3H), 1.54 (s, 3H).

^{13}C NMR (151 MHz, $CDCl_3$) *δ* 173.7, 172.6, 152.3, 144.0, 142.0, 140.4, 138.2, 133.3, 132.3, 131.7, 128.2, 128.0, 127.9, 127.4, 126.8, 126.4, 126.0, 125.6, 125.4, 123.8, 118.1, 110.6, 61.3, 57.0, 55.8, 54.1, 53.5, 47.1, 44.4, 39.4, 26.3, 25.7, 17.7, 16.3, 14.2, 14.0.

HRMS (ESI): $[M+H]^+$ calcd for $[C_{39}H_{43}N_2O_3]$: 587.3268, Found: 587.3256. $[M+Na]^+$ calcd for $[C_{39}H_{42}N_2O_3Na]$: 609.3088, Found: 609.3078.

(2R,3S)-N-(2-(1H-indol-3-yl)ethyl)-4-(3,5-dimethyl-1H-pyrazole-1-carbonyl)-3-(naphthalen-2-yl)-1-phenylbicyclo[2.1.1]hexane-2-carboxamide (16)

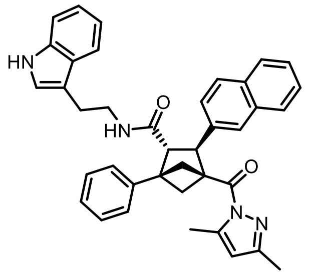

Synthesized according to the **General Procedure A** for the synthesis of Bio-relevant BCHs from carboxylic acid **14** (22.5 mg, 0.05 mmol), Tryptamine (8 mg, 0.05 mmol), HOBt (7.4 mg, 0.055 mmol), EDCI (11.5 mg, 0.06mmol), NMM (10.1 mg, 0.1 mmol) and DMF (0.5 mL) to give the desired product **16** (20.7 mg, 70% yield). R_f = (PE/EA = :).

^{1}H NMR (600 MHz, $CDCl_3$) δ 8.03 – 7.91 (m, 1H), 7.76 – 7.68 (m, 3H), 7.65 (d, J = 8.5 Hz, 1H), 7.40 (m, 3H), 7.32 – 7.24 (m, 3H), 7.24 – 7.16 (m, 4H), 7.12 (t, J = 7.6 Hz, 1H), 7.00 (t, J = 7.5 Hz, 1H), 6.45 (d, J = 2.3 Hz, 1H), 5.87 (s, 1H), 5.13 – 4.99 (m, 2H), 3.55 (dd, J = 9.4, 6.8 Hz, 1H), 3.49 (dq, J = 13.1, 6.6 Hz, 1H), 3.39 – 3.27 (m, 1H), 2.89 (dd, J = 5.1, 1.8 Hz, 1H), 2.72 (dt, J = 9.4, 6.5 Hz, 2H), 2.51 (dt, J = 14.4, 7.0 Hz, 1H), 2.38 (s, 3H), 2.35 (dd, J = 6.9, 1.8 Hz, 1H), 2.31 (dd, J = 7.4, 1.9 Hz, 1H), 2.24 (s, 3H).

^{13}C NMR (151 MHz, $CDCl_3$) δ 173.0, 172.0, 152.4, 143.8, 140.9, 139.0, 136.3, 133.3, 132.2, 128.4, 128.1, 127.8, 127.4, 127.0, 126.8, 126.5, 126.0, 125.9, 125.5, 125.2, 122.0, 121.9, 119.3, 118.5, 112.5, 111.1, 110.6, 60.1, 55.3, 53.3, 53.3, 47.0, 44.4, 39.4, 25.0, 14.2, 14.0.

HRMS (ESI): $[M+H]^+$ calcd for $[C_{39}H_{37}N_4O_2]$: 593.2911, Found: 593.2901. $[M+Na]^+$ calcd for $[C_{39}H_{36}N_4O_2Na]$: 615.2730, Found: 615.2723.

(2R,3S)-4-(3,5-dimethyl-1H-pyrazole-1-carbonyl)-3-(naphthalen-2-yl)-N-(((S)-2-oxo-3-(4-(3-oxomorpholino)phenyl)oxazolidin-5-yl)methyl)-1-phenylbicyclo[2.1.1]hexane-2-carboxamide (17)

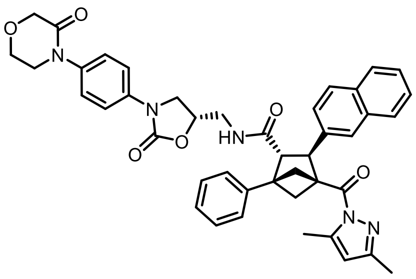

Synthesized according to the **General Procedure A** for the synthesis of Bio-relevant BCHs from carboxylic acid **14** (22.5 mg, 0.05 mmol), (S)-4-(4-(5-(Aminomethyl)-2-oxooxazolidin-3-yl)phenyl)morpholin-3-one (14.6 mg, 0.05 mmol), HOBt (7.4 mg, 0.055 mmol), EDCI (11.5 mg, 0.06mmol), NMM (10.1 mg, 0.1 mmol) and DMF (0.5 mL) to give the desired product **17** (23.5 mg, 65% yield). R_f = (PE/EA = :).

^{1}H NMR (600 MHz, $CDCl_3$) δ 7.74 (dd, J = 7.5, 5.1 Hz, 3H), 7.68 (d, J = 8.5 Hz, 1H), 7.41 (tt, J = 7.1, 5.2 Hz, 2H), 7.36 (d, J = 8.6 Hz, 2H), 7.27 (d, J = 8.9 Hz, 2H), 7.18 (tt, J = 15.0, 7.3 Hz, 5H), 7.05 (t, J = 7.2 Hz, 1H), 6.18 (t, J = 6.0 Hz, 1H), 5.89 (s, 1H), 4.99 (d, J = 5.1 Hz, 1H), 4.40 (tt, J = 8.1, 4.8 Hz, 1H), 4.30 (s, 2H), 3.99 (t, J = 5.0 Hz, 2H), 3.71 (t, J = 5.0 Hz, 2H), 3.54 – 3.41 (m, 3H), 3.27 – 3.19 (m, 2H), 3.09 (t, J = 8.2 Hz, 1H), 2.73 – 2.63 (m, 1H), 2.42 – 2.34 (m, 4H), 2.25 (s, 4H).

^{13}C NMR (151 MHz, $CDCl_3$) δ 173.3, 172.6, 166.8, 154.0, 152.3, 143.8, 140.4, 138.5, 137.0, 136.6, 133.3, 132.2, 128.4, 128.1, 127.8, 127.4, 126.9, 126.3, 126.0, 125.9, 125.7, 125.6, 125.3, 118.7, 110.6, 71.2, 68.4, 64.0, 58.7, 55.5, 53.6, 53.4, 49.6, 46.9, 46.5, 45.0, 40.9, 14.1, 14.0.

HRMS (ESI): $[M+H]^+$ calcd for $[C_{43}H_{42}N_5O_6]$: 724.3130, Found: 724.3118. $[M+Na]^+$ calcd for $[C_{43}H_{41}N_5O_6Na]$: 746.2949, Found: 746.2935.

(2R,3S)-4-(3,5-dimethyl-1H-pyrazole-1-carbonyl)-N-(2-((((E)-5-methoxy-1-(4-(trifluoromethyl)phenyl)pentylidene)amino)oxy)ethyl)-3-(naphthalen-2-yl)-1-phenylbicyclo[2.1.1]hexane-2-carboxamide (18)

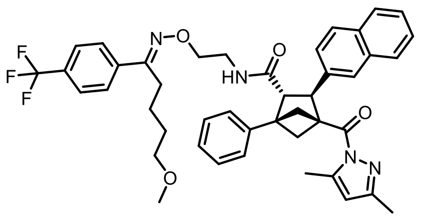

Synthesized according to the **General Procedure A** for the synthesis of Bio-relevant BCHs from carboxylic acid **14** (22.5 mg, 0.05 mmol), Fluvoxamine (15.9 mg, 0.05 mmol), HOBt (7.4 mg, 0.055 mmol), EDCI (11.5 mg, 0.06mmol), NMM (10.1 mg, 0.1 mmol) and DMF (0.5 mL) to give the desired product **18** (28.1 mg, 75% yield). R_f = 0.38 (PE/EA = 3:1).

^{1}H NMR (600 MHz, $CDCl_3$) δ 7.76 – 7.70 (m, 3H), 7.67 (d, J = 8.5 Hz, 1H), 7.63 (d, J = 8.2 Hz, 2H), 7.56 (d, J = 8.3 Hz, 2H), 7.40 (p, J = 7.6 Hz, 2H), 7.31 – 7.22 (m, 5H), 7.16 (t, J = 7.2 Hz, 1H), 5.88 (s, 1H), 5.70 (t, J = 5.2 Hz, 1H), 5.10 (d, J = 4.5 Hz, 1H), 4.15 – 4.05 (m, 1H), 3.86 (ddd, J = 11.0, 7.2, 3.6 Hz, 1H), 3.56 (dd, J = 9.2, 7.0 Hz, 1H), 3.51 (ddd, J = 17.8, 9.7, 5.9 Hz, 1H), 3.41 (ddd, J = 14.3, 8.7, 4.8 Hz, 1H), 3.30 – 3.23 (m, 2H), 3.17 (s, 3H), 3.00 (d, J = 4.2 Hz, 1H), 2.79 – 2.73 (m, 1H), 2.58 (t, J = 7.4 Hz, 2H), 2.39 (s, 3H), 2.37 – 2.32 (m, 2H), 2.26 (s, 3H), 1.50 – 1.37 (m, 4H).

^{13}C NMR (151 MHz, $CDCl_3$) δ 173.0, 172.3, 157.5, 152.3, 143.8, 140.8, 139.1, 138.8, 133.4, 132.2, 128.4, 128.1, 127.7, 127.4, 126.8, 126.4, 126.4, 126.0, 125.8, 125.5, 125.4, 125.3, 125.3, 125.3, 125.2, 110.6, 72.8, 72.0, 60.1, 58.4, 55.3, 53.4, 53.3, 47.1, 44.4, 39.1, 29.1, 25.7, 22.8, 14.2, 14.0.

^{19}F NMR (565 MHz, $CDCl_3$) δ -62.70.

HRMS (ESI): $[M+H]^+$ calcd for $[C_{44}H_{46}F_3N_4O_4]$: 751.3466, Found: 751.3453. $[M+Na]^+$ calcd for $[C_{44}H_{45}F_3N_4O_4Na]$: 773.3285, Found: 773.3272.

(3S,8S,9S,10R,13R,14S,17R)-10,13-dimethyl-17-((R)-6-methylheptan-2-yl)-2,3,4,7,8,9,10,11,12,13,14,15,16,17-tetradecahydro-1H-cyclopenta[a]phenanthren-3-yl (2R,3S)-4-(3,5-dimethyl-1H-pyrazole-1-carbonyl)-3-(naphthalen-2-yl)-1-phenylbicyclo[2.1.1]hexane-2-carboxylate (19)

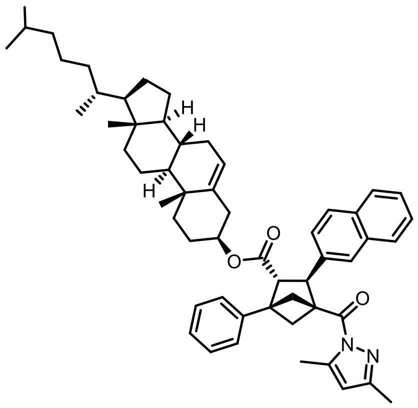

Synthesized according to the **General Procedure B** for the ssynthesis of Bio-relevant BCHs from carboxylic acid **14** (18 mg, 0.04 mmol), Cholesterol (15.5 mg, 0.04 mmol), DMAP (1 mg, 0.008 mmol), EDCI (15.2 mg, 0.08mmol) and DCM (0.4 mL) to give the desired product **19** (25.3 mg, 77% yield). R_f = 0.68 (PE/EA = 10:1).

^{1}H NMR (600 MHz, $CDCl_3$) δ 7.80 – 7.68 (m, 4H), 7.42 (qd, *J* = 6.8, 3.3 Hz, 2H), 7.32 (t, *J* = 7.5 Hz, 2H), 7.28 – 7.20 (m, 4H), 5.90 (s, 1H), 5.35 – 5.28 (m, 1H), 4.94 (d, *J* = 4.6 Hz, 1H), 4.57 – 4.41 (m, 1H), 3.42 (dd, *J* = 4.9, 1.1 Hz, 1H), 3.36 (dd, *J* = 9.2, 7.1 Hz, 1H), 2.75 – 2.68 (m, 1H), 2.44 (dd, *J* = 6.9, 1.2 Hz, 1H), 2.39 (s, 3H), 2.29 (dd, *J* = 7.5, 1.3 Hz, 1H), 2.27 – 2.19 (m, 4H), 2.15 – 2.08 (m, 1H), 1.98 (dd, *J* = 9.5, 3.2 Hz, 1H), 1.92 (m, 1H), 1.85 – 1.80 (m, 1H), 1.71 (dd, *J* = 10.0, 3.3 Hz, 1H), 1.52 – 1.42 (m, 4H), 1.40 – 1.31 (m, 5H), 1.28 – 1.22 (m, 2H), 1.17 – 1.05 (m, 7H), 1.03 – 0.94 (m, 4H), 0.90 (d, *J* = 7.0 Hz, 6H), 0.87 (d, *J* = 2.6 Hz, 3H), 0.85 (d, *J* = 2.6 Hz, 3H), 0.65 (s, 3H).

^{13}C NMR (151 MHz, $CDCl_3$) *δ* 173.0, 172.7, 152.3, 143.9, 140.4, 139.6, 138.4, 133.4, 132.3, 128.1, 128.1, 127.9, 127.5, 126.7, 126.4, 126.1, 126.0, 125.6, 125.4, 122.5, 110.6, 74.1, 57.3, 56.6, 56.1, 55.7, 53.8, 53.5, 49.9, 46.9, 44.6, 42.3, 39.7, 39.5, 38.0, 36.9, 36.5, 36.2, 35.8, 31.8, 31.8, 28.2, 28.0, 27.2, 24.2, 23.8, 22.8, 22.5, 21.0, 19.2, 18.7, 14.2, 14.0, 11.8.

HRMS (ESI): $[M+H]^+$ calcd for $[C_{56}H_{71}N_2O_3]$:819.5459, Found: 819.5458. $[M+Na]^+$ calcd for $[C_{56}H_{70}N_2O_3Na]$: 841.5279, Found: 841.5278.

Ethyl (3R,4R,5S)-4-acetamido-5-((2R,3S)-4-(3,5-dimethyl-1H-pyrazole-1-carbonyl)-3-(naphthalen-2-yl)-1-phenylbicyclo[2.1.1]hexane-2-carboxamido)-3-(pentan-3-yloxy)cyclohex-1-ene-1-carboxylate (20)

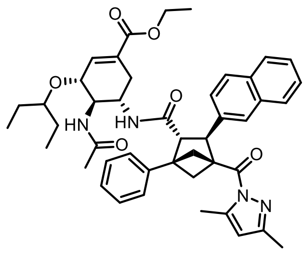

Synthesized according to the **General Procedure A** for the synthesis of Bio-relevant BCHs from carboxylic acid **14** (45 mg, 0.1 mmol), Fluvoxamine (31.2 mg, 0.1 mmol), HOBt (14.8 mg, 0.11 mmol), EDCI (23 mg, 0.12mmol), NMM (20.2 mg, 0.2 mmol) and DMF (1 mL) to give the desired product **20** (54.6 mg, 74% yield). R_f = 0.50 (PE/EA = 1:1).

^{1}H NMR (600 MHz, $CDCl_3$) δ 7.73 (d, J = 7.9 Hz, 1H), 7.69 (s, 1H), 7.64 (d, J = 7.9 Hz, 1H), 7.55 (d, J = 8.5 Hz, 1H), 7.38 (m, 4H), 7.28 – 7.19 (m, 3H), 7.08 (d, J = 8.4 Hz, 1H), 6.63 (s, 1H), 6.15 (d, J = 8.0 Hz, 1H), 5.94 (d, J = 8.5 Hz, 1H), 5.89 (s, 1H), 5.06 (d, J = 4.4 Hz, 1H), 4.26 – 4.14 (m, 2H), 4.01 – 3.92 (m, 2H), 3.83 (dd, J = 19.2, 8.6 Hz, 1H), 3.40 (dd, J = 8.9, 7.2 Hz, 1H), 3.31 (p, J = 5.5 Hz, 1H), 3.05 (d, J = 4.5 Hz, 1H), 2.72 (t, J = 8.4 Hz, 1H), 2.40 (s, 3H), 2.38 – 2.29 (m, 3H), 2.21 (s, 3H), 1.64 (s, 3H), 1.51 – 1.41 (m, 5H), 1.33 (t, J = 7.1 Hz, 3H), 0.86 (t, J = 7.4 Hz, 3H), 0.80 (t, J = 7.4 Hz, 3H).

^{13}C NMR (151 MHz, $CDCl_3$) δ 172.8, 172.3, 171.6, 165.6, 152.2, 143.9, 140.4, 138.3, 136.9, 133.4, 132.2, 129.2, 128.6, 128.1, 127.8, 127.4, 127.0, 126.1, 126.0, 125.8, 125.5, 125.0, 110.6, 81.9, 75.1, 60.8, 59.4, 55.4, 53.7, 53.3, 52.4, 48.8, 46.4, 44.5, 30.2, 26.1, 25.6, 22.8, 14.2, 14.2, 13.9, 9.4,

HRMS (ESI): $[M+H]^+$ calcd for $[C_{45}H_{53}N_4O_6]$:745.3960, Found: 745.3944. $[M+Na]^+$ calcd for $[C_{45}H_{52}N_4O_6Na]$: 767.3779, Found: 767.3766.

(2R,3S)-4-(3,5-dimethyl-1H-pyrazole-1-carbonyl)-N-(((1R,4aS,10aR)-7-isopropyl-1,4a-dimethyl-1,2,3,4,4a,9,10,10a-octahydrophenanthren-1-yl)methyl)-3-(naphthalen-2-yl)-1-phenylbicyclo[2.1.1]hexane-2-carboxamide (21)

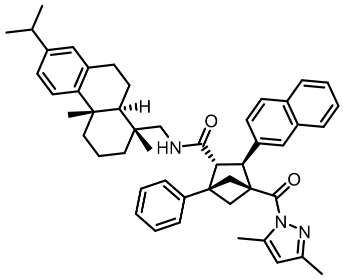

Synthesized according to the **General Procedure A** for the synthesis of Bio-relevant BCHs from carboxylic acid **14** (45 mg, 0.1 mmol), Dehydroabietylamine (28.5 mg, 0.1 mmol), HOBt (14.8 mg, 0.11 mmol), EDCI (23 mg, 0.12mmol), NMM (20.2 mg, 0.2 mmol) and DMF (1 mL) to give the desired product **21** (66 mg, 92% yield). R_f = (PE/EA =:).

^{1}H NMR (600 MHz, $CDCl_3$) δ 7.74 (dd, *J* = 13.3, 7.0 Hz, 3H), 7.65 (d, *J* = 8.5 Hz, 1H), 7.45 – 7.36 (m, 2H), 7.27 (dd, *J* = 8.5, 1.5 Hz, 1H), 7.24 – 7.14 (m, 4H), 7.12 (ddd, *J* = 10.9, 6.0, 3.1 Hz, 2H), 7.03 – 6.93 (m, 1H), 6.86 (s, 1H), 5.88 (s, 1H), 5.11 (d, *J* = 4.4 Hz, 1H), 4.89 (t, *J* = 5.9 Hz, 1H), 3.32 (dd, *J* = 9.2, 7.0 Hz, 1H), 3.10 (dd, *J* = 13.6, 7.3 Hz, 2H), 2.92 – 2.76 (m, 3H), 2.76 – 2.69 (m, 1H), 2.64 – 2.54 (m, 1H), 2.40 (dd, *J* = 6.9, 1.5 Hz, 1H), 2.38 (s, 3H), 2.28 (dd, *J* = 7.5, 1.6 Hz, 1H), 2.25 (s, 3H), 2.15 (d, *J* = 12.8 Hz, 1H), 1.64 – 1.53 (m, 3H), 1.52 – 1.43 (m, 1H), 1.24 (d, *J* = 1.7 Hz, 3H), 1.23 (d, *J* = 1.7 Hz, 3H), 1.19 – 1.14 (m, 1H), 1.12 (s, 3H), 1.05 – 0.95 (m, 2H), 0.82 (td, *J* = 13.1, 3.7 Hz, 1H), 0.76 (s, 3H).

^{13}C NMR (151 MHz, $CDCl_3$) *δ* 172.9, 172.2, 152.3, 147.1, 145.6, 143.8, 140.8, 139.1, 134.7, 133.4, 132.2, 128.6, 128.1, 127.8, 127.5, 126.9, 126.8, 126.6, 126.0, 125.8, 125.5, 125.2, 124.1, 123.8, 110.6, 60.0, 55.3, 53.1, 53.0, 50.1, 46.6, 45.2, 45.1, 38.0, 37.3, 36.8, 35.6, 33.4, 30.0, 25.2, 24.0, 24.0, 18.7, 18.4, 18.4, 14.2, 14.0.

HRMS (ESI): $[M+H]^+$ calcd for $[C_{49}H_{56}N_3O_2]$: 718.4367, Found: 718.4354. $[M+Na]^+$ calcd for $[C_{49}H_{55}N_3O_2Na]$: 740.4186, Found: 740.4175.

(3aR,5R,6S,6aR)-5-(2,2-dimethyl-1,3-dioxolan-4-yl)-2,2-dimethyltetrahydrofuro [2,3-d][1,3]dioxol-6-yl (2R,3S)-4-(3,5-dimethyl-1H-pyrazole-1-carbonyl)-3-(naphthalen-2-yl)-1-phenylbicyclo[2.1.1]hexane-2-carboxylate (22)

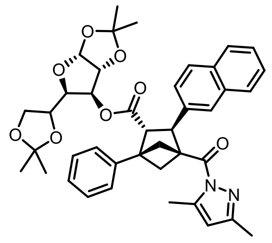

Synthesized according to the **General Procedure B** for the ssynthesis of Bio-relevant BCHs from carboxylic acid **14** (22.5 mg, 0.05 mmol), Diacetone-D-glucose (13 mg, 0.05 mmol), DMAP (1.3 mg, 0.01 mmol), EDCI (19 mg, 0.1mmol) and DCM (0.5 mL) to give the desired product **22** (20 mg, 58% yield). $R_f = 0.27$ (PE/EA = 10:1).

^{1}H NMR (600 MHz, $CDCl_3$) δ 7.77 (dd, *J* = 6.9, 5.4 Hz, 2H), 7.73 (d, *J* = 8.8 Hz, 2H), 7.49 – 7.41 (m, 2H), 7.35 (t, *J* = 7.6 Hz, 2H), 7.28 – 7.24 (m, 4H), 5.91 (s, 1H), 5.73 (d, *J* = 3.6 Hz, 1H), 5.19 (d, *J* = 3.0 Hz, 1H), 4.90 (d, *J* = 4.9 Hz, 1H), 4.19 (dd, *J* = 6.8, 3.0 Hz, 1H), 4.17 (d, *J* = 3.6 Hz, 1H), 3.90 (dd, *J* = 8.0, 4.7 Hz, 1H), 3.84 – 3.74 (m, 2H), 3.52 – 3.46 (m, 1H), 3.31 (dd, *J* = 9.3, 7.3 Hz, 1H), 2.75 – 2.67 (m, 1H), 2.55 – 2.50 (m, 1H), 2.39 (s, 3H), 2.30 (d, *J* = 7.6 Hz, 1H), 2.26 (s, 3H), 1.48 (s, 3H), 1.36 (s, 3H), 1.24 (d, *J* = 4.2 Hz, 6H).

^{13}C NMR (151 MHz, $CDCl_3$) *δ* 172.2, 172.0, 152.4, 144.1, 140.0, 137.8, 133.3, 132.4, 128.5, 128.3, 127.8, 127.5, 127.1, 126.2, 126.2, 125.9, 125.8, 125.3, 112.2, 110.7, 109.1, 104.9, 83.2, 79.6, 76.5, 72.5, 66.8, 57.6, 55.7, 54.4, 53.2, 47.3, 44.9, 26.8, 26.7, 26.1, 25.5, 14.2, 13.9.

HRMS (ESI): $[M+H]^+$ calcd for $[C_{41}H_{45}N_2O_8]$: 693.3170, Found: 693.3159. $[M+Na]^+$ calcd for $[C_{41}H_{44}N_2O_8Na]$: 715.2990, Found: 715.2980.

(3S,8S,9S,10R,13S,14S,17S)-17-acetyl-10,13-dimethyl-2,3,4,7,8,9,10,11,12,13,14,15,16,17-tetradecahydro-1H-cyclopenta[a]phenanthren-3-yl (2R,3S)-4-(3,5-dimethyl-1H-pyrazole-1-carbonyl)-3-(naphthalen-2-yl)-1-phenylbicyclo[2.1.1]hexane-2-carboxylate (23)

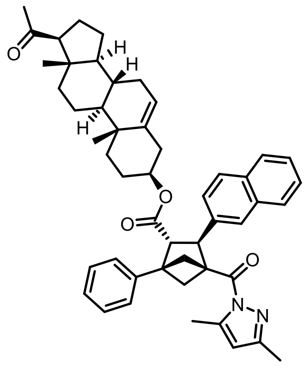

Synthesized according to the **General Procedure B** for the ssynthesis of Bio-relevant BCHs from carboxylic acid **14** (22.5 mg, 0.05 mmol), Pregnenolone (15..8 mg, 0.05 mmol), DMAP (1.3 mg, 0.01 mmol), EDCI (19 mg, 0.1mmol) and DCM (0.5 mL) to give the desired product **23** (36.6 mg, 98% yield). R_f = 0.27 (PE/EA = 10:1).

^{1}H NMR (600 MHz, $CDCl_3$) δ 7.76 – 7.65 (m, 4H), 7.45 – 7.36 (m, 2H), 7.29 (t, *J* = 7.5 Hz, 2H), 7.27 – 7.16 (m, 4H), 5.87 (s, 1H), 5.34 – 5.20 (m, 1H), 4.91 (d, *J* = 4.7 Hz, 1H), 4.54 – 4.38 (m, 1H), 3.40 (d, *J* = 5.0 Hz, 1H), 3.33 (dd, *J* = 9.1, 7.3 Hz, 1H), 2.69 (t, *J* = 8.5 Hz, 1H), 2.53 – 2.44 (m, 1H), 2.41 (t, *J* = 10.5 Hz, 1H), 2.36 (s, 3H), 2.26 (d, *J* = 7.6 Hz, 1H), 2.24 – 2.17 (m, 4H), 2.16 – 2.04 (m, 5H), 2.01 – 1.97 (m, 1H), 1.96 – 1.88 (m, 1H), 1.72 – 1.66 (m, 1H), 1.65 – 1.59 (m, 2H), 1.56 – 1.46 (m, 2H), 1.43 – 1.37 (m, 3H), 1.33 (d, *J* = 12.5 Hz, 1H), 1.17 (ddd, *J* = 17.2, 11.6, 5.7 Hz, 1H), 1.15 – 1.03 (m, 2H), 1.01 – 0.95 (m, 1H), 0.92 (td, *J* = 11.0, 4.8 Hz, 1H), 0.86 (d, *J* = 10.6 Hz, 3H), 0.59 (d, *J* = 14.5 Hz, 3H).

^{13}C NMR (151 MHz, $CDCl_3$) δ 209.5, 173.0, 172.6, 152.3, 143.9, 140.4, 139.6, 138.3, 133.3, 132.3, 128.2, 128.1, 128.0, 127.8, 127.4, 126.7, 126.4, 126.1, 126.0, 125.6, 125.4, 122.1, 110.6, 73.9, 63.6, 57.2, 56.8, 55.7, 53.7, 53.5, 49.8, 46.8, 44.6, 43.9, 38.7, 37.9, 36.9, 36.5, 31.7, 31.7, 31.5, 27.1, 24.4, 22.8, 20.9, 19.1, 14.2, 14.0, 13.2.

HRMS (ESI): $[M+H]^+$ calcd for $[C_{50}H_{57}N_2O_4]$: 749.4313, Found: 749.4302. $[M+Na]^+$ calcd for $[C_{50}H_{56}N_2O_4Na]$: 771.4132, Found: 771.4121.

(1R,2S,5R)-2-isopropyl-5-methylcyclohexyl (2R,3S)-4-(3,5-dimethyl-1H-pyrazole-1-carbonyl)-3-(naphthalen-2-yl)-1-phenylbicyclo[2.1.1]hexane-2-carboxylate (24)

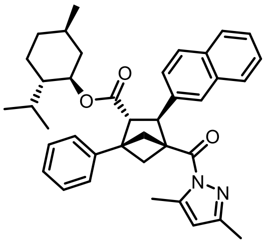

Synthesized according to the **General Procedure B** for the ssynthesis of Bio-relevant BCHs from carboxylic acid **14** (22.5 mg, 0.05 mmol), L-Menthol (7.8 mg, 0.05 mmol), DMAP (1.3 mg, 0.01 mmol), EDCI (19 mg, 0.1mmol) and DCM (0.5 mL) to give the desired product **24** (25.3 mg, 86% yield). $R_f = 0.62$ (PE/EA = 10:1).

^{1}H NMR (600 MHz, $CDCl_3$) δ 7.78 – 7.71 (m, 4H), 7.47 – 7.40 (m, 2H), 7.30 (t, J = 7.5 Hz, 2H), 7.27 – 7.21 (m, 4H), 5.89 (s, 1H), 4.92 (d, J = 4.7 Hz, 1H), 4.60 (td, J = 10.9, 4.4 Hz, 1H), 3.46 (dd, J = 9.4, 7.0 Hz, 1H), 3.41 (dd, J = 5.1, 1.6 Hz, 1H), 2.68 (dd, J = 9.3, 7.7 Hz, 1H), 2.47 (dd, J = 6.9, 1.7 Hz, 1H), 2.41 (s, 3H), 2.27 (dd, J = 7.5, 1.6 Hz, 1H), 2.25 (s, 3H), 1.94 (dd, J = 6.8, 4.4 Hz, 1H), 1.62 (s, 1H), 1.57 – 1.52 (m, 1H), 1.44 – 1.38 (m, 1H), 1.18 – 1.12 (m, 1H), 1.03 – 0.97 (m, 1H), 0.92 (dd, J = 13.0, 3.3 Hz, 1H), 0.90 (s, 1H), 0.85 (d, J = 6.6 Hz, 3H), 0.81 – 0.76 (m, 1H), 0.63 (d, J = 7.0 Hz, 3H), 0.43 (d, J = 6.9 Hz, 3H).

^{13}C NMR (151 MHz, $CDCl_3$) δ 173.4, 172.8, 152.3, 144.0, 140.3, 138.6, 133.4, 132.3, 128.2, 128.1, 127.9, 127.5, 126.8, 126.3, 126.0, 126.0, 125.6, 125.1, 110.6, 74.3, 57.9, 55.4, 54.7, 53.4, 46.8, 46.6, 45.0, 40.9, 34.2, 31.4, 25.1, 22.9, 22.0, 20.9, 15.8, 14.2, 14.0.

HRMS (ESI): $[M+H]^+$ calcd for $[C_{39}H_{45}N_2O_3]$: 589.3425, Found: 589.3416. $[M+Na]^+$ calcd for $[C_{39}H_{44}N_2O_3Na]$: 611.3244, Found: 611.3238.

(2R,3S)-N-((R)-1-(7-(but-2-yn-1-yl)-3-methyl-1-((4-methylquinazolin-2-yl)methyl)-2,6-dioxo-2,3,6,7-tetrahydro-1H-purin-8-yl)piperidin-3-yl)-4-(3,5-dimethyl-1H-pyrazole-1-carbonyl)-3-(naphthalen-2-yl)-1-phenylbicyclo[2.1.1]hexane-2-carboxamide (25)

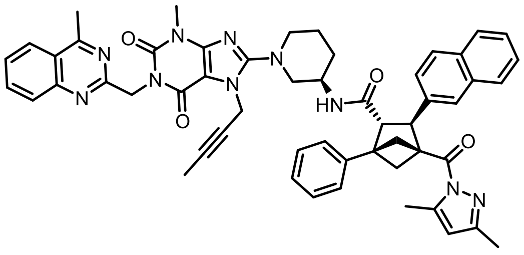

Synthesized according to the **General Procedure A** for the synthesis of Bio-relevant BCHs from carboxylic acid **14** (22.5 mg, 0.05 mmol), Linagliptin (23.6 mg, 0.05 mmol), HOBt (7.4 mg, 0.055 mmol), EDCI (11.5 mg, 0.06mmol), NMM (10.1 mg, 0.1 mmol) and DMF (0.5 mL) to give the desired product **25** (29.4 mg, 65% yield). R_f = (PE/EA =:).

^{1}H NMR (600 MHz, $CDCl_3$) δ 7.99 (dd, J = 8.3, 1.4 Hz, 1H), 7.84 (d, J = 8.5 Hz, 1H), 7.78 – 7.67 (m, 5H), 7.50 (ddd, J = 8.2, 6.8, 1.2 Hz, 1H), 7.44 – 7.37 (m, 2H), 7.31 – 7.18 (m, 5H), 7.14 (td, J = 7.0, 1.5 Hz, 1H), 5.95 (d, J = 7.8 Hz, 1H), 5.87 (s, 1H), 5.64 – 5.49 (m, 2H), 5.09 (dd, J = 4.9, 1.7 Hz, 1H), 4.79 (dq, J = 17.6, 2.4 Hz, 1H), 4.51 – 4.38 (m, 1H), 4.13 (tt, J = 6.3, 3.0 Hz, 1H), 3.57 – 3.47 (m, 4H), 3.27 (td, J = 11.9, 4.0 Hz, 2H), 3.19 (ddd, J = 12.2, 7.4, 3.6 Hz, 1H), 3.03 (dd, J = 4.9, 1.7 Hz, 1H), 2.87 (s, 3H), 2.75 (dd, J = 9.4, 7.4 Hz, 1H), 2.57 (dd, J = 12.7, 5.8 Hz, 1H), 2.42 – 2.31 (m, 5H), 2.25 (s, 3H), 1.77 – 1.57 (m, 7H).

^{13}C NMR (151 MHz, $CDCl_3$) δ 172.7, 171.4, 168.5, 161.0, 155.5, 154.3, 152.3, 151.7, 149.9, 147.4, 143.8, 140.8, 139.0, 133.3, 133.2, 132.1, 128.8, 128.3, 128.1, 127.6, 127.3, 126.7, 126.6, 126.2, 126.0, 125.8, 125.5, 125.2, 124.8, 123.1, 110.6, 104.5, 81.3, 73.0, 60.2, 55.2, 54.0, 53.2, 53.2, 50.8, 47.0, 46.2, 44.7, 44.4, 35.2, 29.6, 28.9, 22.1, 21.7, 14.1, 13.9, 3.5.

HRMS (ESI): $[M+H]^+$ calcd for $[C_{54}H_{53}N_{10}O_4]$: 905.4246, Found: 905.4234. $[M+Na]^+$ calcd for $[C_{54}H_{52}N_{10}O_4Na]$: 927.4065, Found: 927.4055.

(8R,9S,10R,13S,14S,17S)-10,13-dimethyl-3-oxo-2,3,6,7,8,9,10,11,12,13,14,15,16,17-tetradecahydro-1H-cyclopenta[a]phenanthren-17-yl (2R,3S)-4-(3,5-dimethyl-1H-pyrazole-1-carbonyl)-3-(naphthalen-2-yl)-1-phenylbicyclo[2.1.1]hexane-2-carboxylate (26)

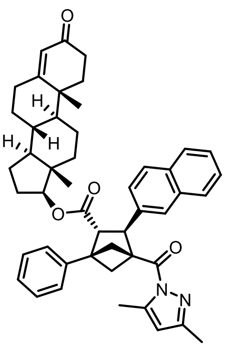

Synthesized according to the **General Procedure B** for the ssynthesis of Bio-relevant BCHs from carboxylic acid **14** (22.5 mg, 0.05 mmol), Testosterone (14.4 mg, 0.05 mmol), DMAP (1.3 mg, 0.01 mmol), EDCI (19 mg, 0.1mmol) and DCM (0.5 mL) to give the desired product **26** (25 mg, 69% yield). R_f = 0.35 (PE/EA = 3:1).

^{1}H NMR **(600 MHz, CDCl$_3$)** δ 7.86 – 7.70 (m, 4H), 7.51 – 7.40 (m, 2H), 7.32 (t, J = 7.5 Hz, 2H), 7.29 – 7.20 (m, 4H), 5.91 (s, 1H), 5.71 (s, 1H), 4.96 (d, J = 4.6 Hz, 1H), 4.56 – 4.44 (m, 1H), 3.46 (dd, J = 5.1, 1.6 Hz, 1H), 3.37 – 3.26 (m, 1H), 2.71 (dd, J = 9.3, 7.8 Hz, 1H), 2.48 (dd, J = 7.0, 1.6 Hz, 1H), 2.40 (s, 3H), 2.38 – 2.27 (m, 4H), 2.25 (s, 3H), 2.05 – 1.92 (m, 2H), 1.82 – 1.76 (m, 1H), 1.75 – 1.62 (m, 3H), 1.59 – 1.47 (m, 3H), 1.33 (ddd, J = 26.3, 13.3, 4.1 Hz, 1H), 1.20 – 1.08 (m, 5H), 1.03 – 0.87 (m, 4H), 0.58 (s, 3H).

^{13}C NMR **(151 MHz, CDCl$_3$)** δ 199.6, 173.6, 172.8, 171.1, 152.5, 144.2, 140.5, 138.6, 133.6, 132.5, 128.4, 128.3, 128.1, 127.7, 127.0, 126.6, 126.3, 126.3, 125.9, 125.5, 124.1, 110.8, 83.0, 57.5, 55.8, 54.0, 53.8, 53.6, 50.2, 47.0, 45.1, 42.5, 38.8, 36.7, 35.8, 35.5, 34.1, 32.9, 31.6, 27.4, 23.6, 20.6, 17.5, 14.4, 14.2, 12.2.

HRMS (ESI): [M+H]$^+$ calcd for [$C_{48}H_{53}N_2O_4$]: 721.4000, Found: 721.3992. [M+Na]$^+$ calcd for [$C_{48}H_{52}N_2O_4Na$]: 743.3819, Found: 743.3813.

6. X-ray Crystallographic Data

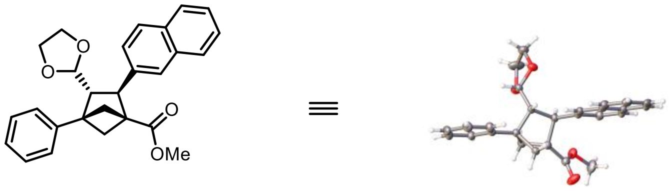

9 CCDC 2441435

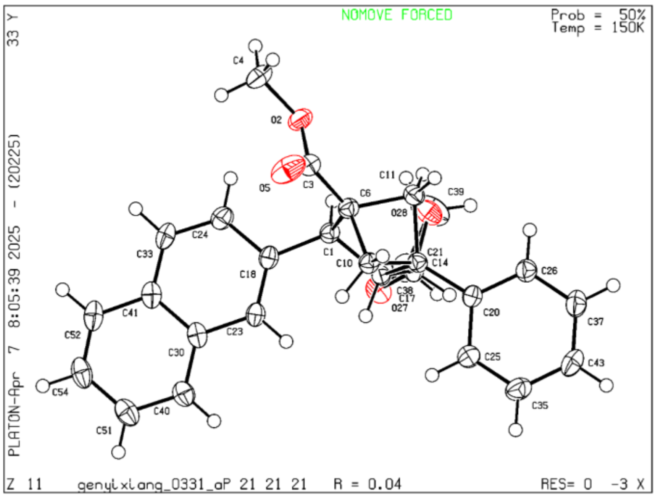

Figure S7. The X-ray structure of **9**

The crystal **9** was grown by dissolving 200 mg of the product in a small amount of ethyl acetate (≤2.5 mL), followed by the addition of approximately 5 mL of hexane. The solution was left partially uncovered in a cabinet at room temperature, and after about two weeks, crystals formed.

Table S3. Crystal data and structure refinement for **9**

Empirical formula	$C_{27}H_{26}O_4$
Formula weight	414.48
Temperature/K	150.00(10)
Crystal system	orthorhombic
Space group	$P2_12_12_1$
a/Å	9.44202(16)
b/Å	11.5317(2)
c/Å	19.5818(4)
α/°	90
β/°	90

γ/°	90
Volume/Å^3	2132.12(7)
Z	4
ρ_{calc}g/cm^3	1.291
μ/mm^{-1}	0.687
F(000)	880.0
Crystal size/mm^3	0.09 × 0.08 × 0.07
Radiation	Cu Kα (λ = 1.54184)
2Θ range for data collection/°	8.9 to 155.464
Index ranges	-9 ≤ h ≤ 11, -13 ≤ k ≤ 14, -24 ≤ l ≤ 20
Reflections collected	12628
Independent reflections	4306 [R_{int} = 0.0367, R_{sigma} = 0.0346]
Data/restraints/parameters	4306/0/281
Goodness-of-fit on F^2	1.079
Final R indexes [I>=2σ (I)]	R_1 = 0.0358, wR_2 = 0.0952
Final R indexes [all data]	R_1 = 0.0381, wR_2 = 0.1006
Largest diff. peak/hole / e Å^{-3}	0.19/-0.18
Flack parameter	0.03(12)

7. NMR Spectra of Compound

^{1}H NMR of **3a** in $CDCl_3$ (600 MHz, $CDCl_3$)

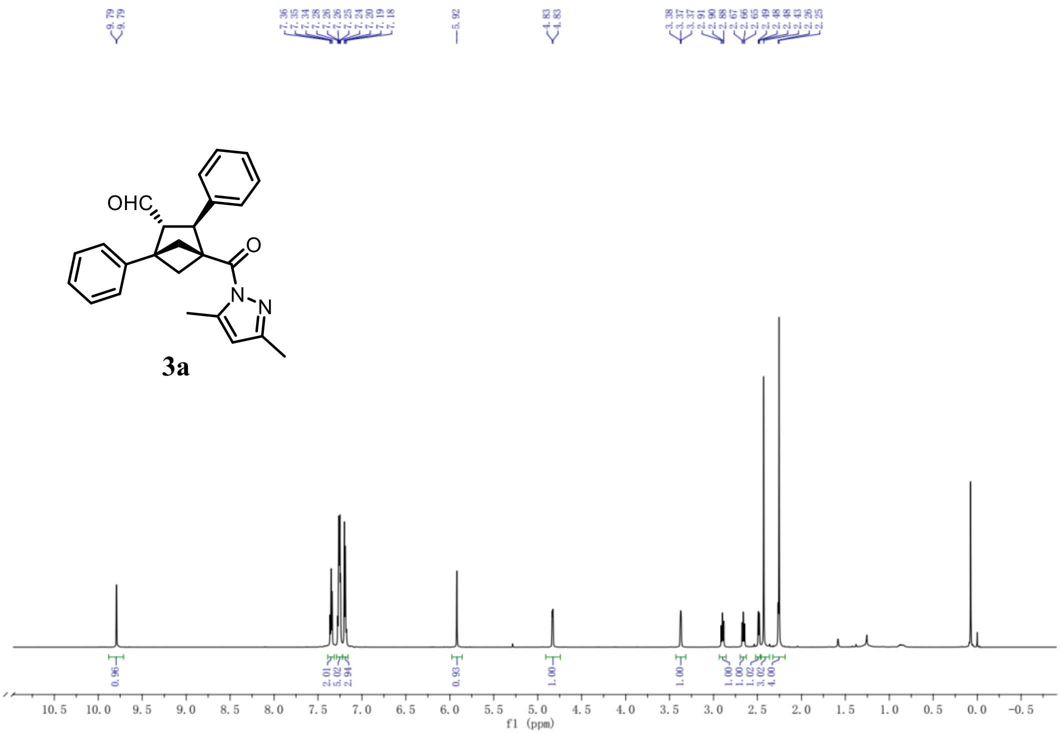

^{13}C NMR of **3a** in $CDCl_3$ (151 MHz, $CDCl_3$)

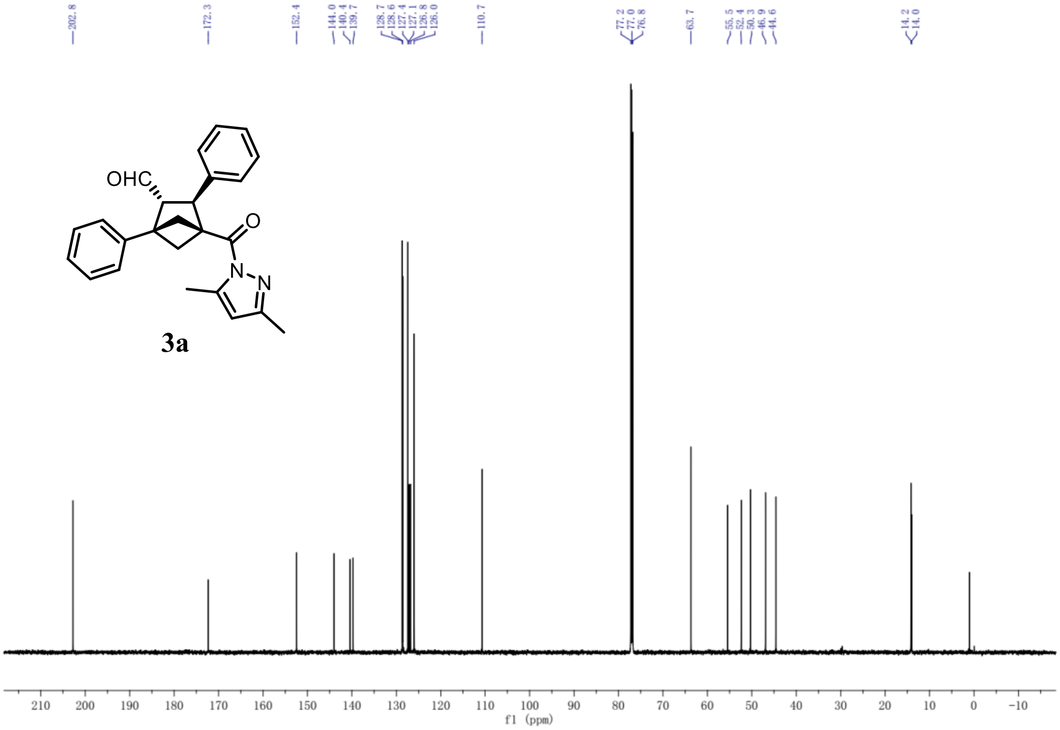

^{1}H NMR of **3b** in $CDCl_3$ (600 MHz, $CDCl_3$)

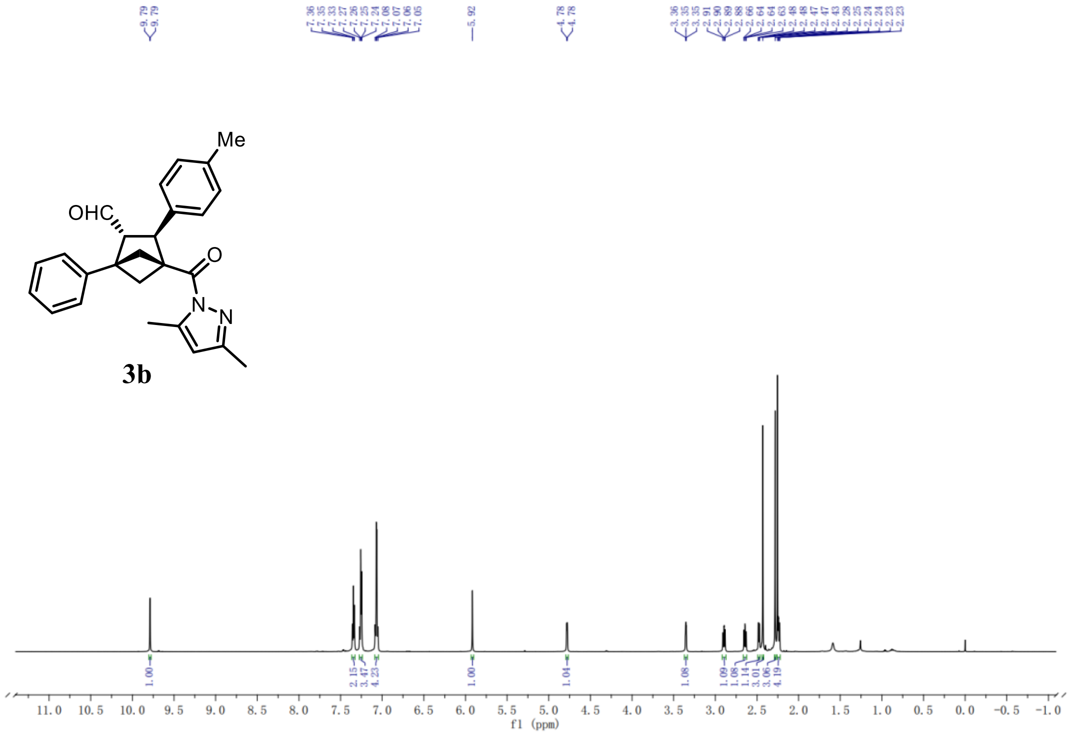

^{13}C NMR of **3b** in $CDCl_3$ (151 MHz, $CDCl_3$)

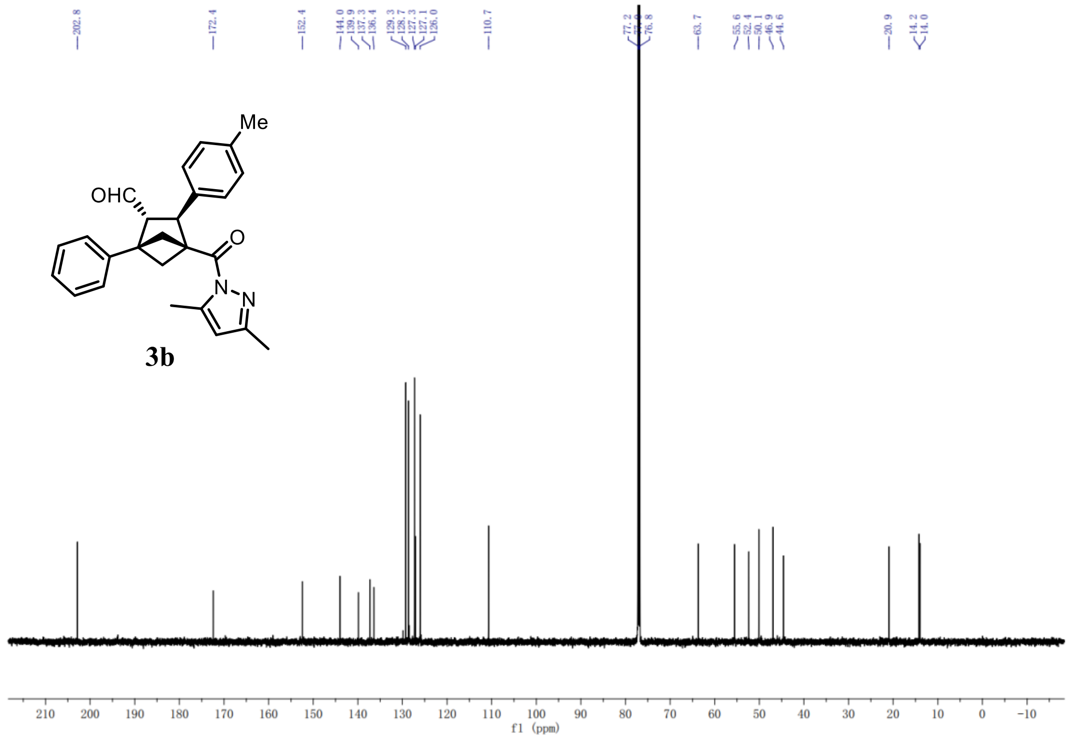

^{1}H NMR of **3c** in $CDCl_3$ (600 MHz, $CDCl_3$)

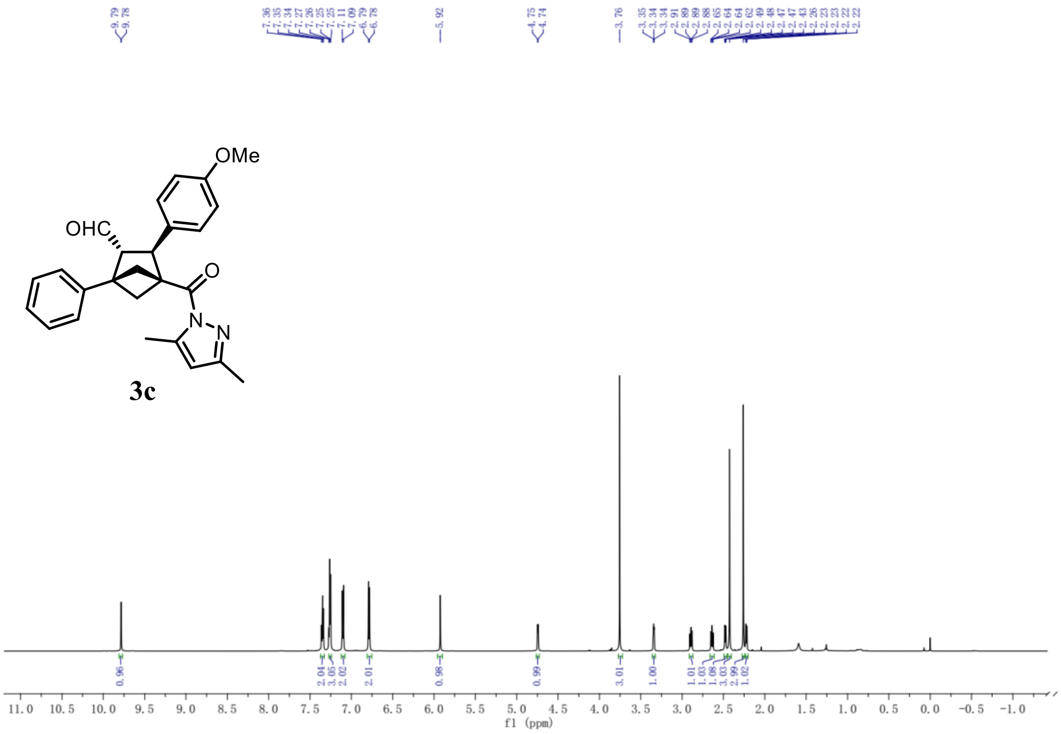

^{13}C NMR of **3c** in $CDCl_3$ (151 MHz, $CDCl_3$)

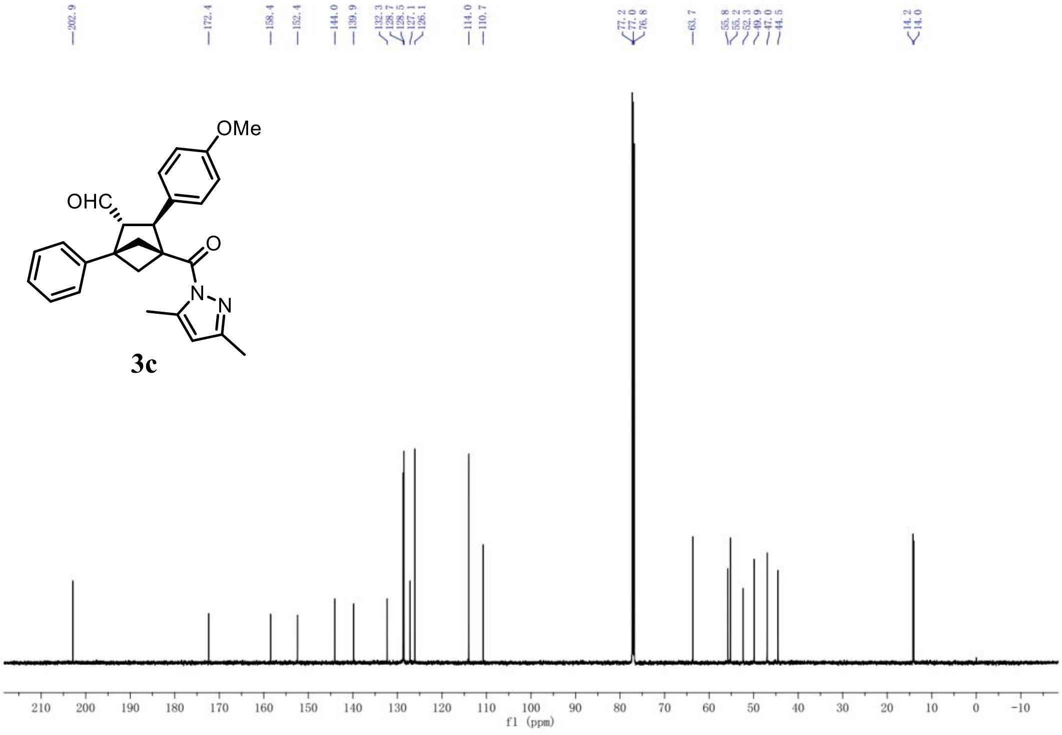

^{1}H NMR of **3d** in $CDCl_3$ (600 MHz, $CDCl_3$)

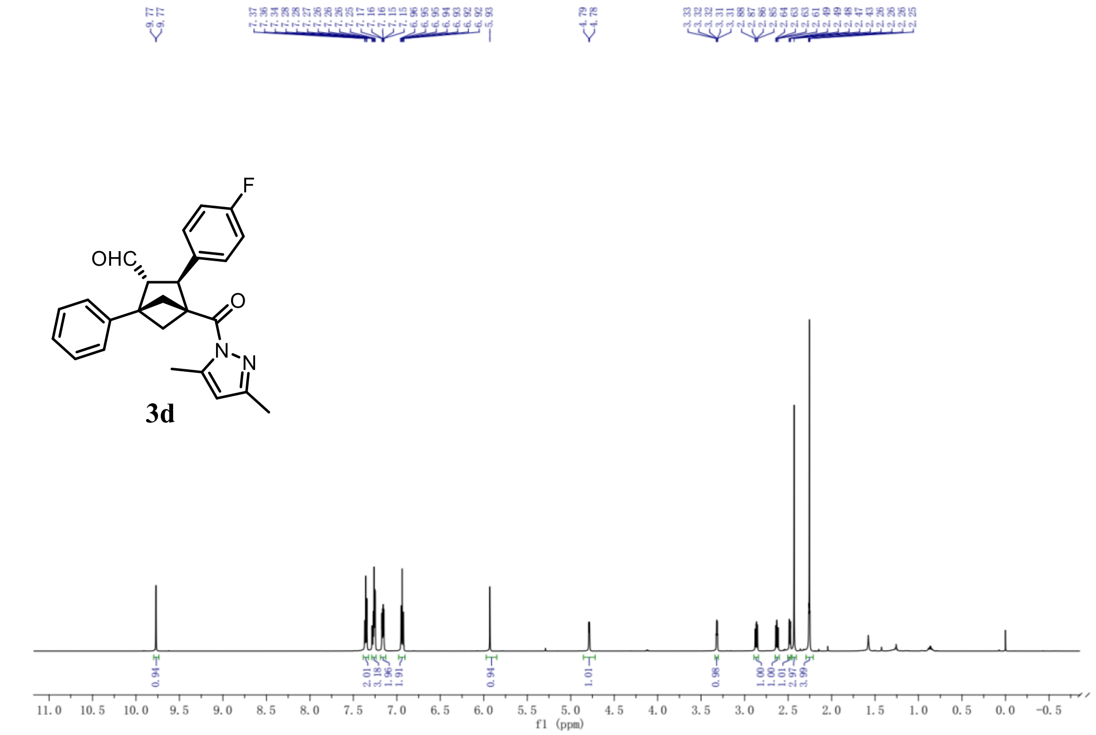

^{13}C NMR of **3d** in $CDCl_3$ (151 MHz, $CDCl_3$)

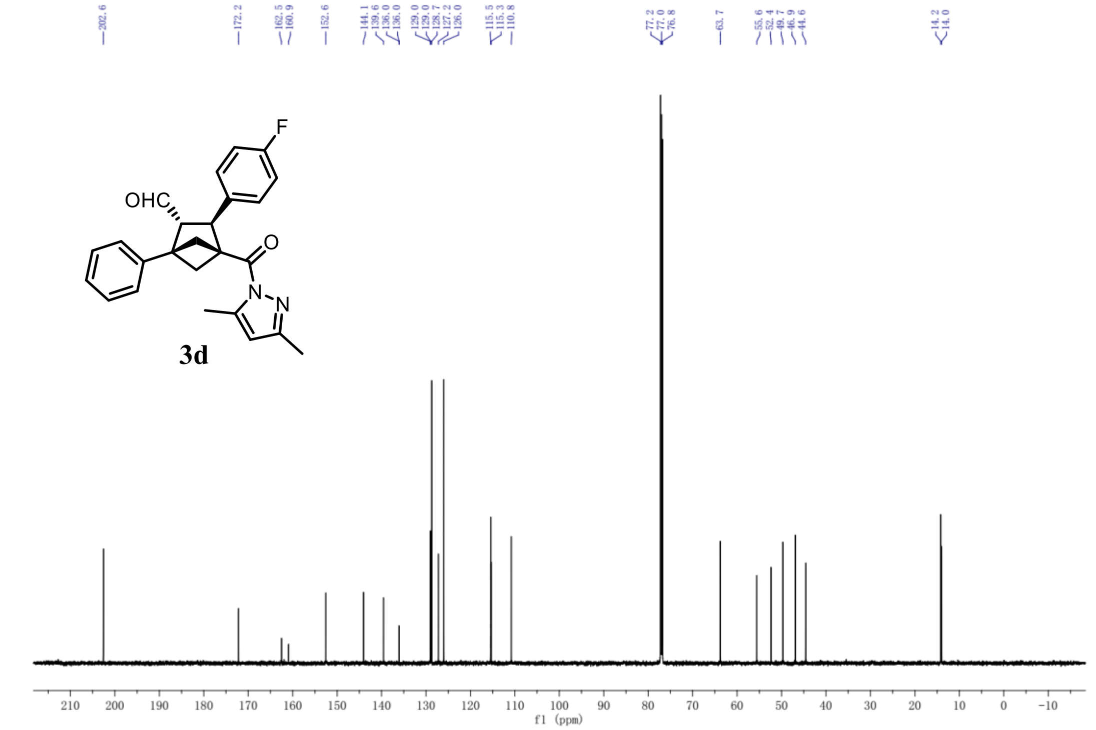

^{19}F NMR of **3d** in $CDCl_3$ (565 MHz, $CDCl_3$)

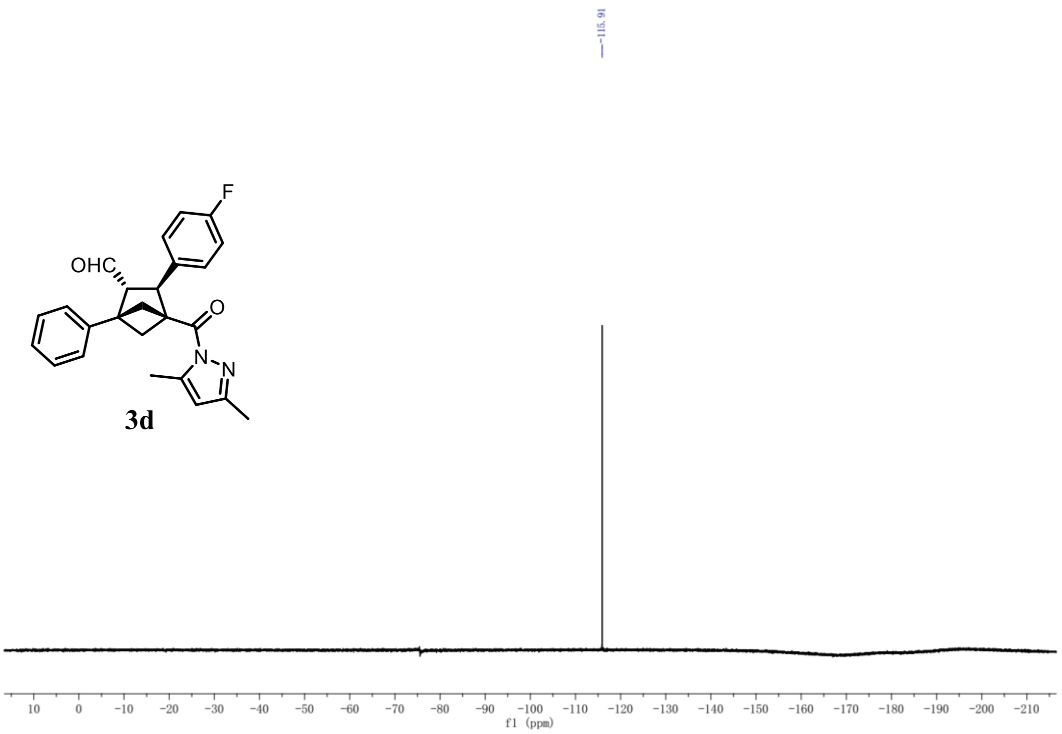

^{1}H NMR of **3e** in $CDCl_3$ (600 MHz, $CDCl_3$)

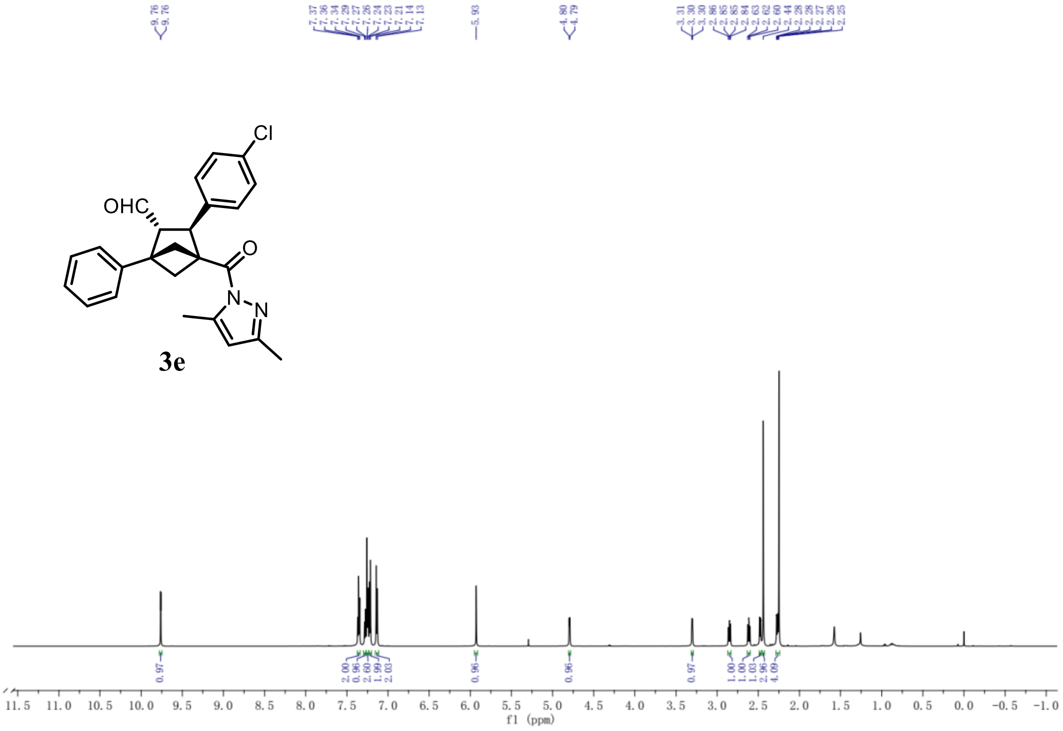

^{13}C NMR of **3e** in $CDCl_3$ (151 MHz, $CDCl_3$)

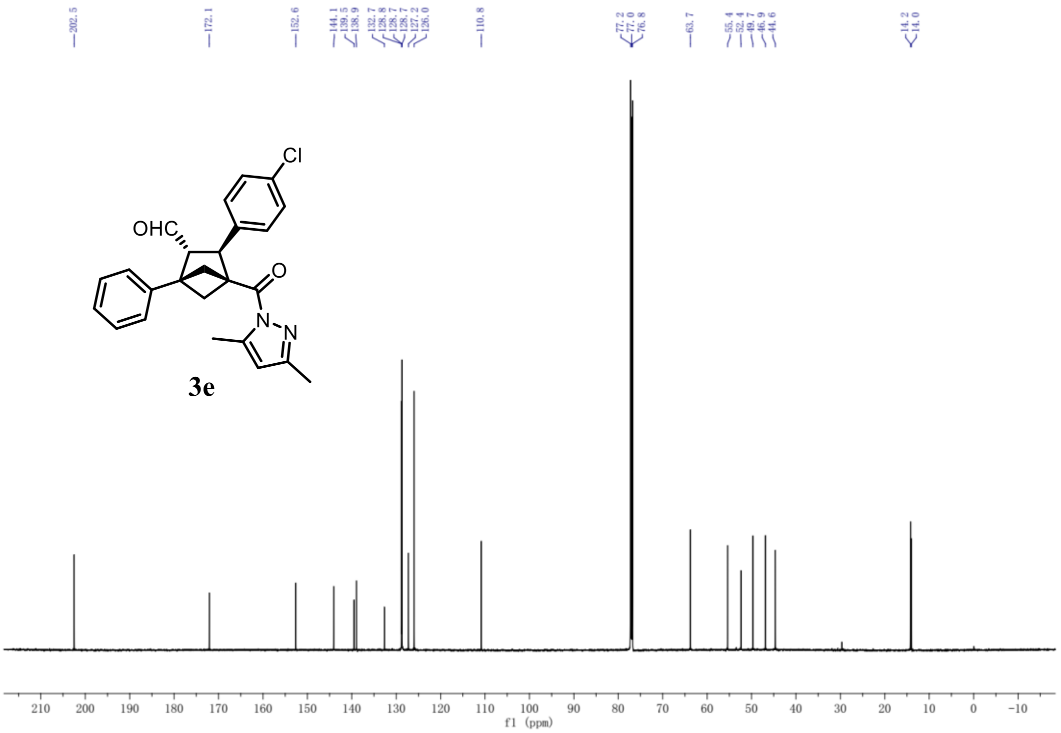

^{1}H NMR of **3f** in $CDCl_3$ (600 MHz, $CDCl_3$)

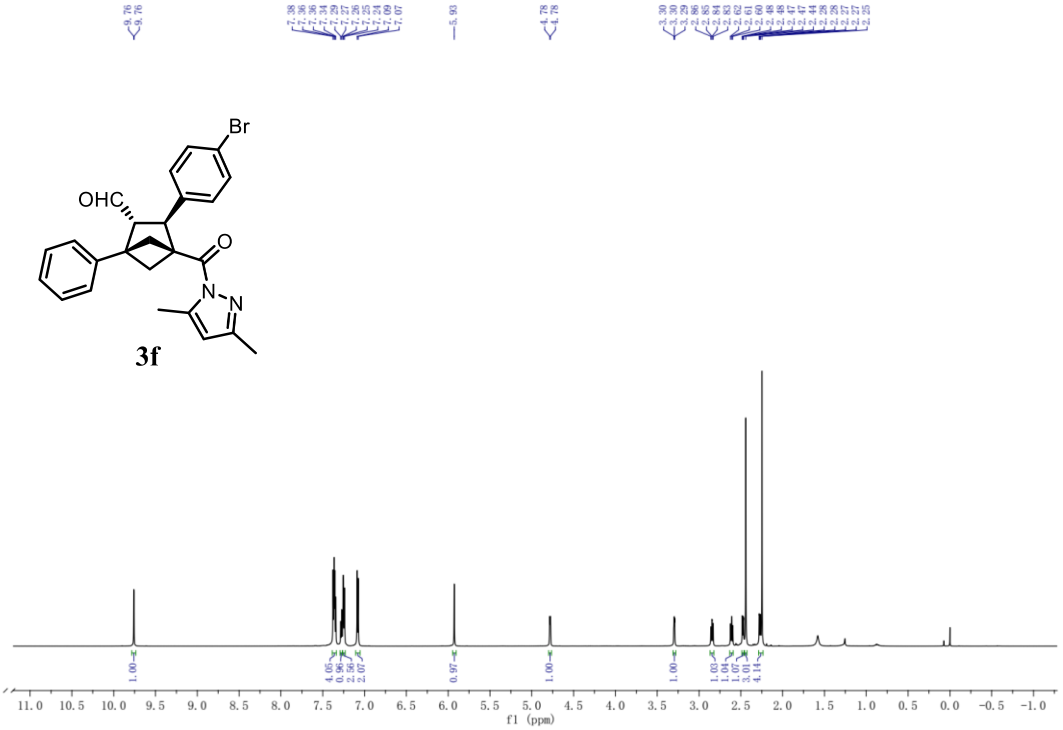

^{13}C NMR of **3f** in $CDCl_3$ (151 MHz, $CDCl_3$)

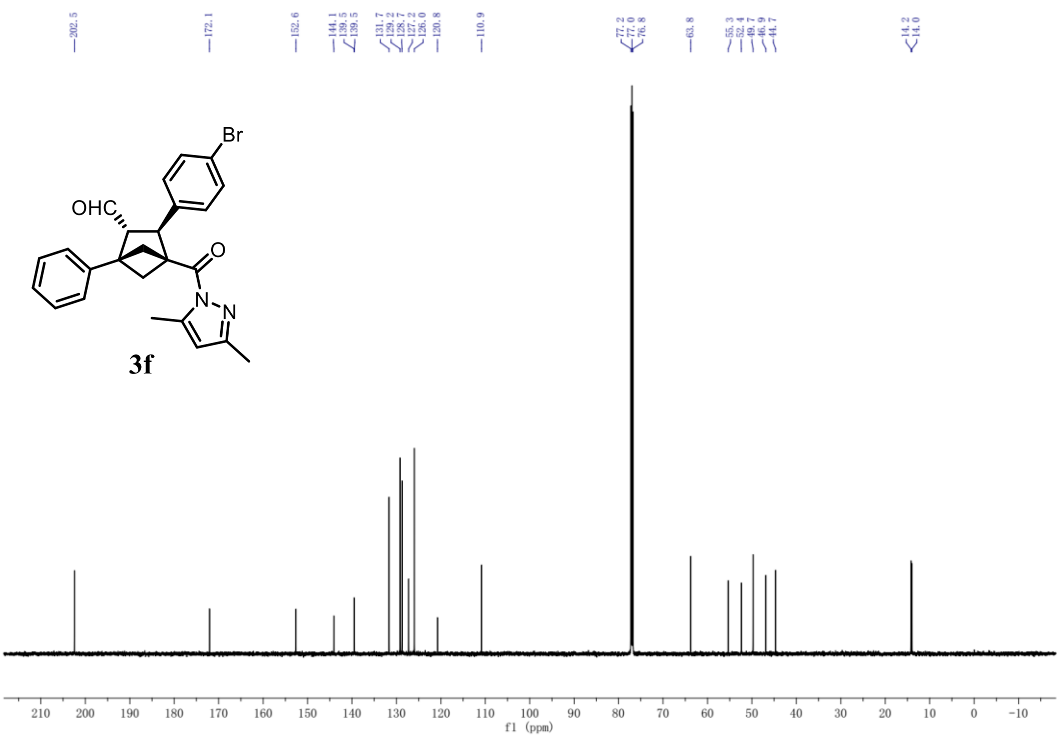

^{1}H NMR of **3g** in $CDCl_3$ (600 MHz, $CDCl_3$)

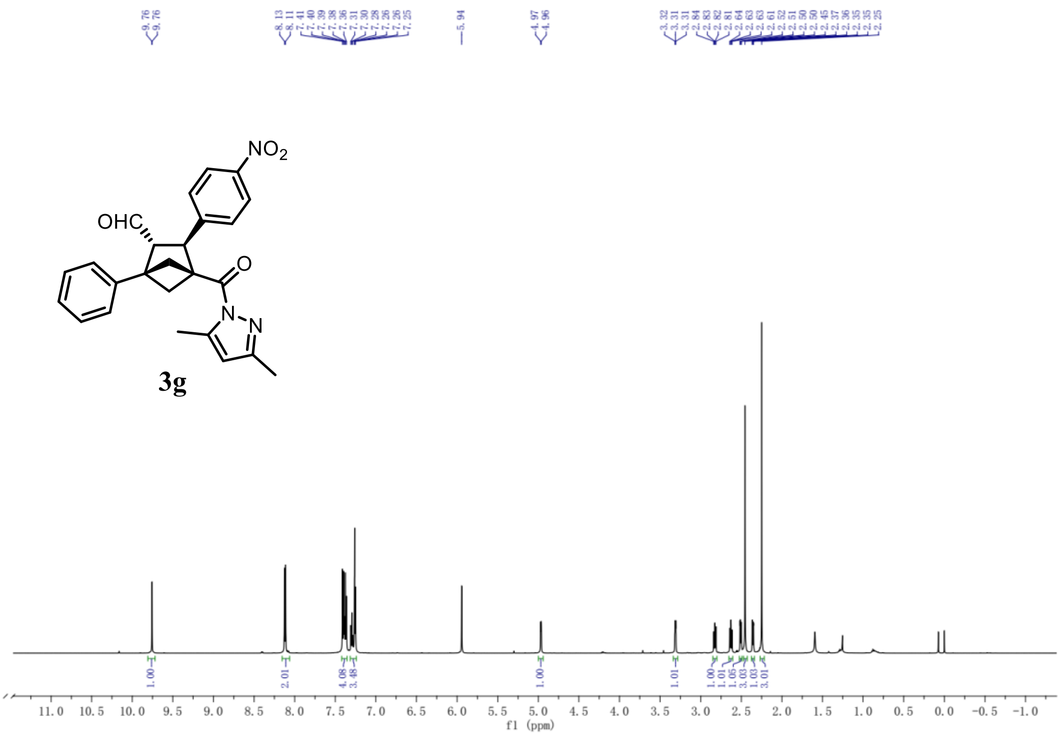

^{13}C NMR of **3g** in $CDCl_3$ (151 MHz, $CDCl_3$)

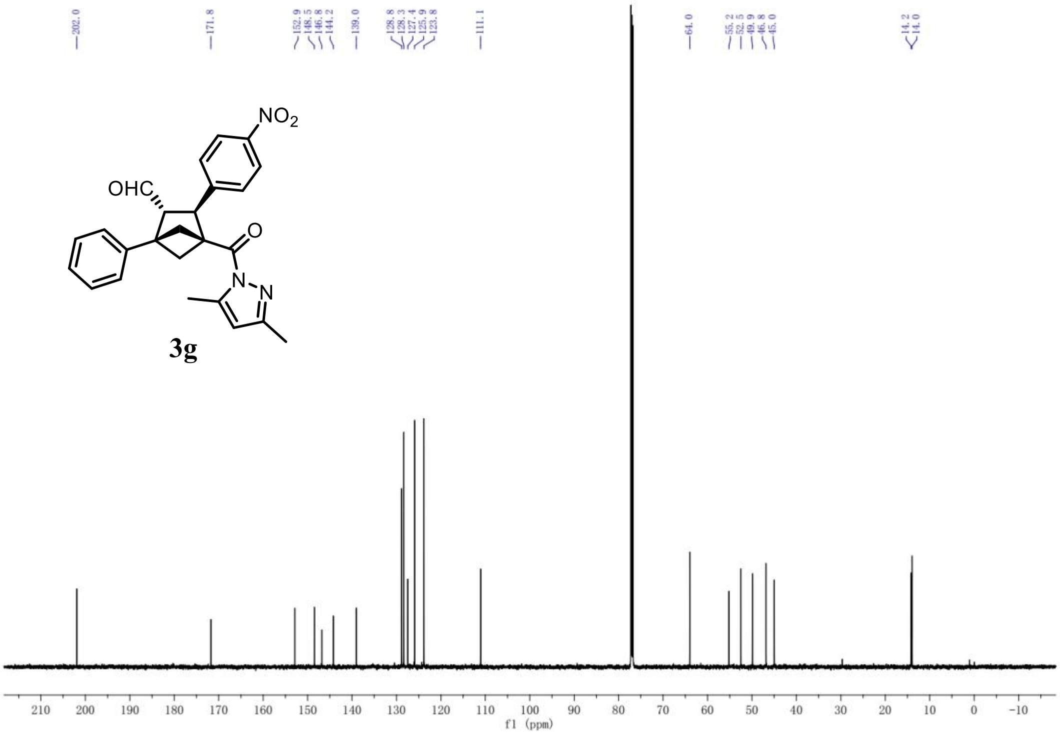

^{1}H NMR of **3h** in $CDCl_3$ (600 MHz, $CDCl_3$)

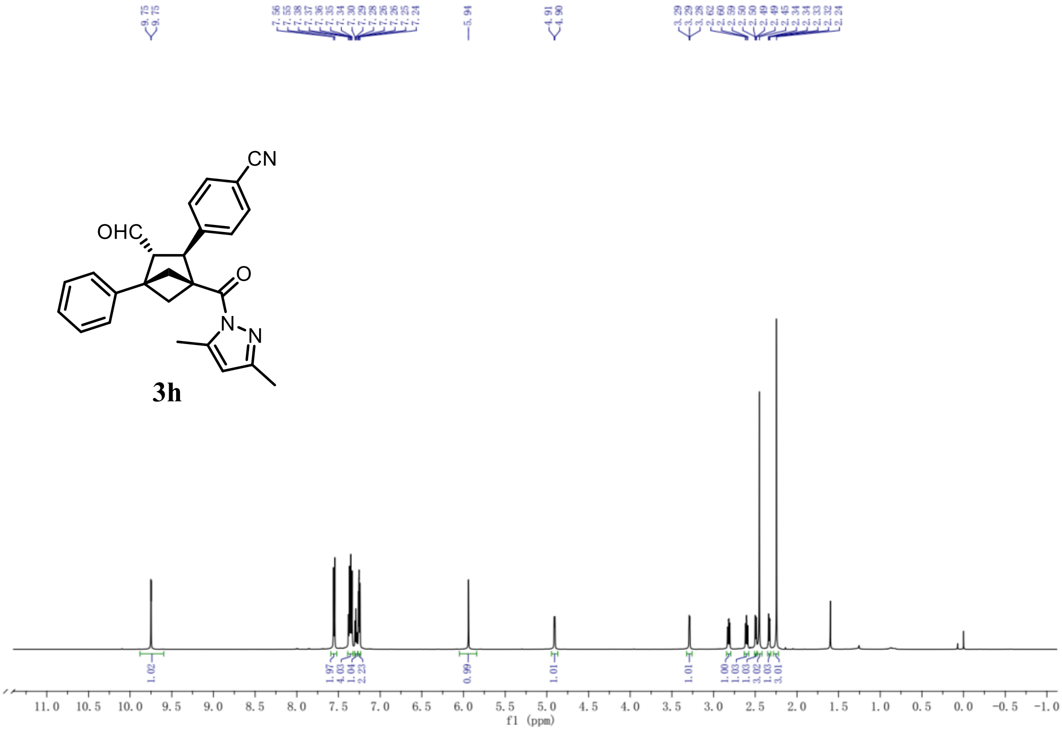

^{13}C NMR of **3h** in $CDCl_3$ (151 MHz, $CDCl_3$)

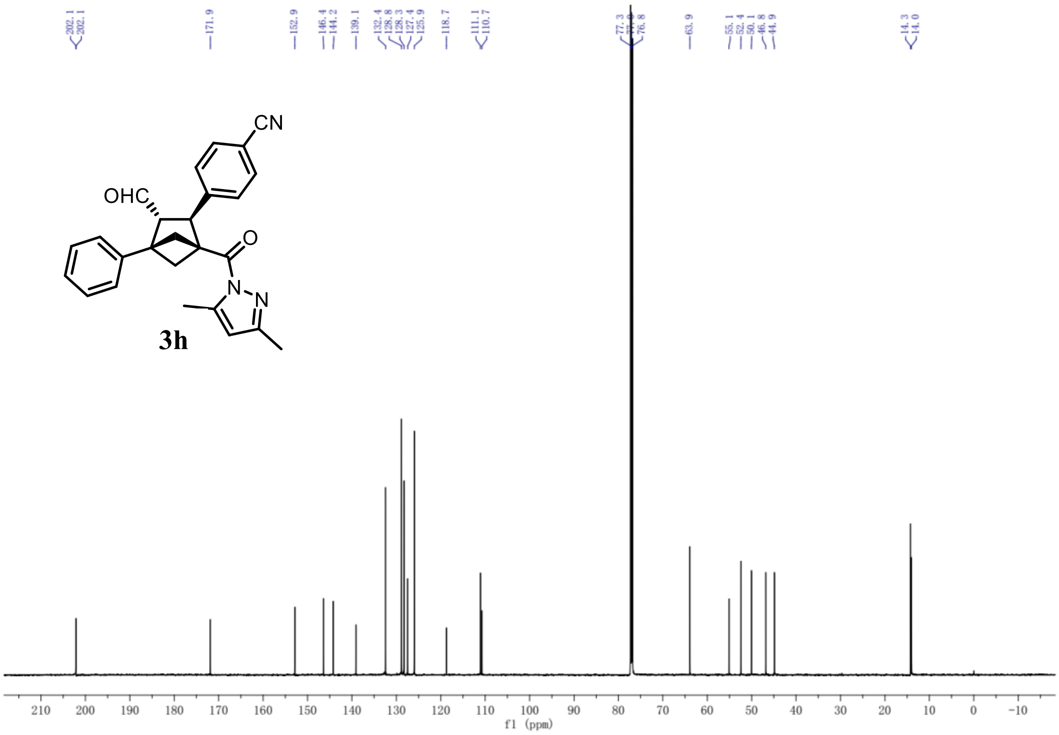

^{1}H NMR of **3i** in $CDCl_3$ (600 MHz, $CDCl_3$)

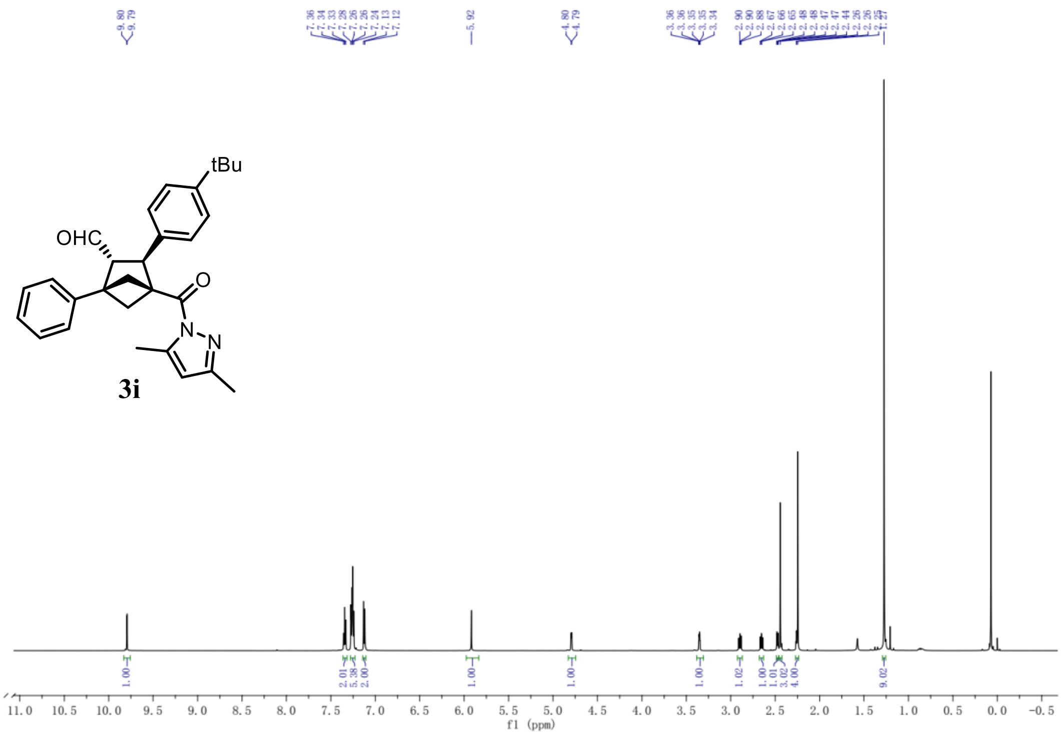

^{13}C NMR of **3i** in $CDCl_3$ (151 MHz, $CDCl_3$)

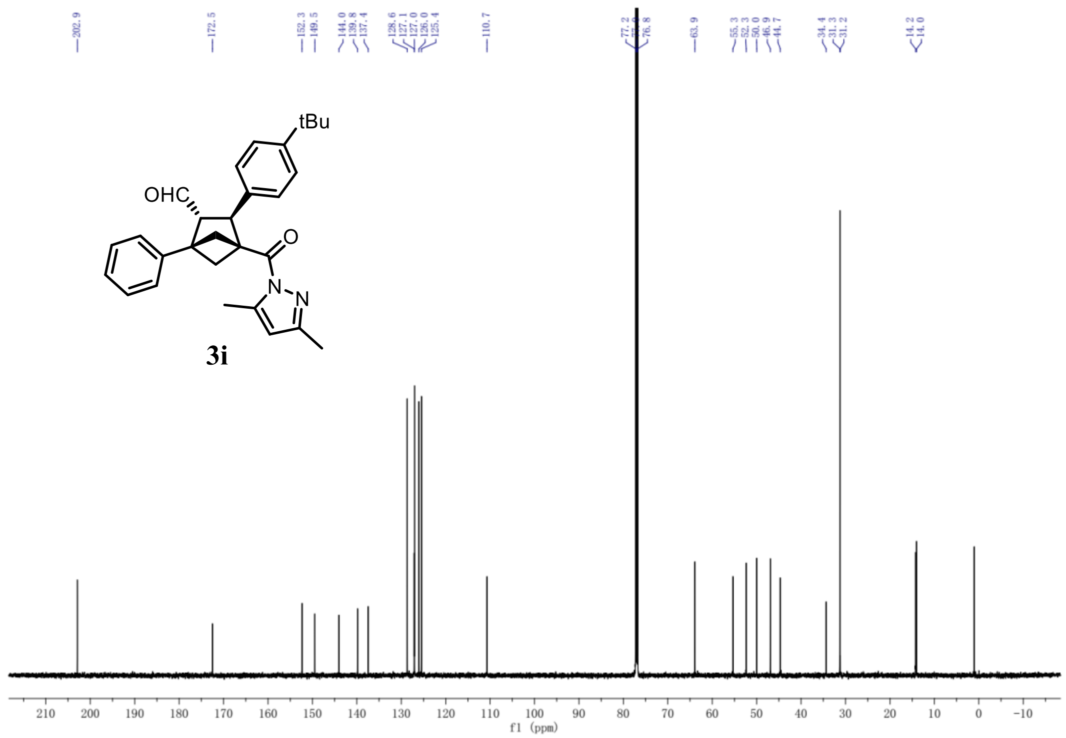

^{1}H NMR of **3j** in $CDCl_3$ (600 MHz, $CDCl_3$)

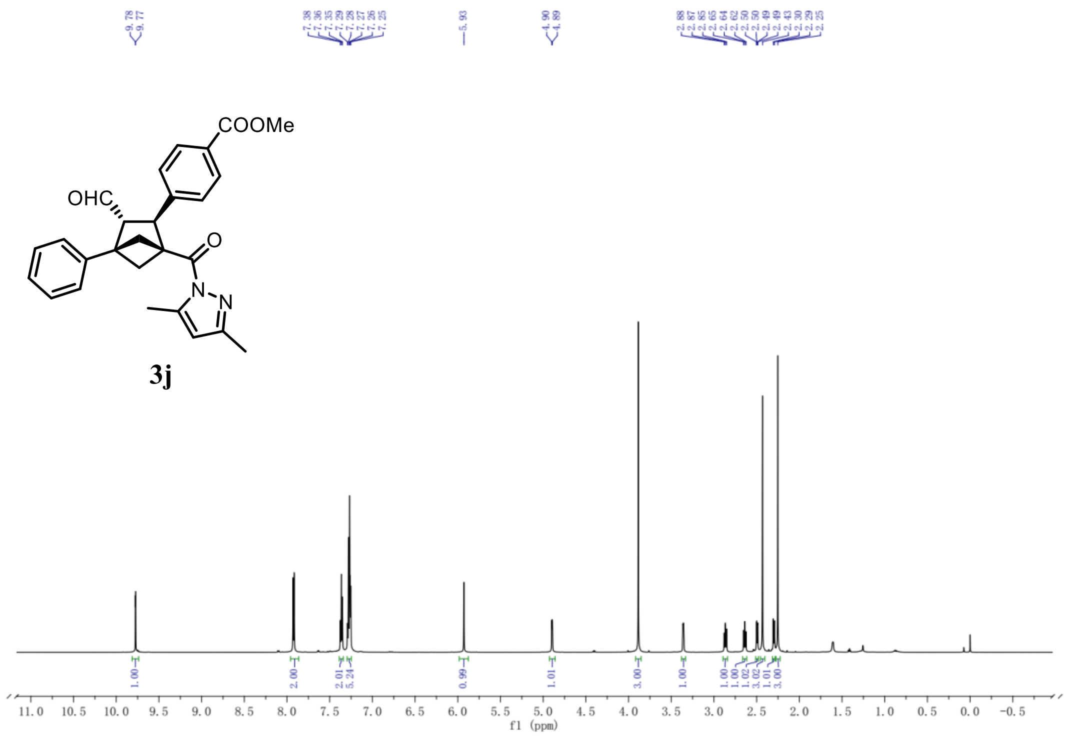

^{13}C NMR of **3j** in $CDCl_3$ (151 MHz, $CDCl_3$)

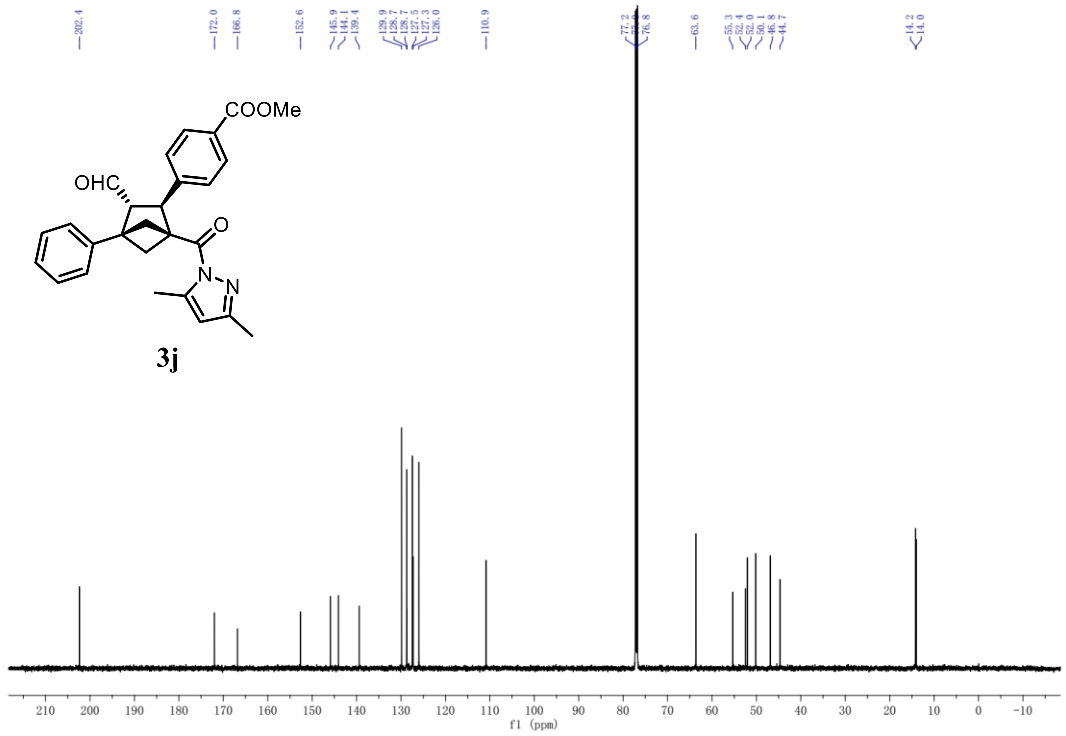

^{1}H NMR of **3k** in $CDCl_3$ (600 MHz, $CDCl_3$)

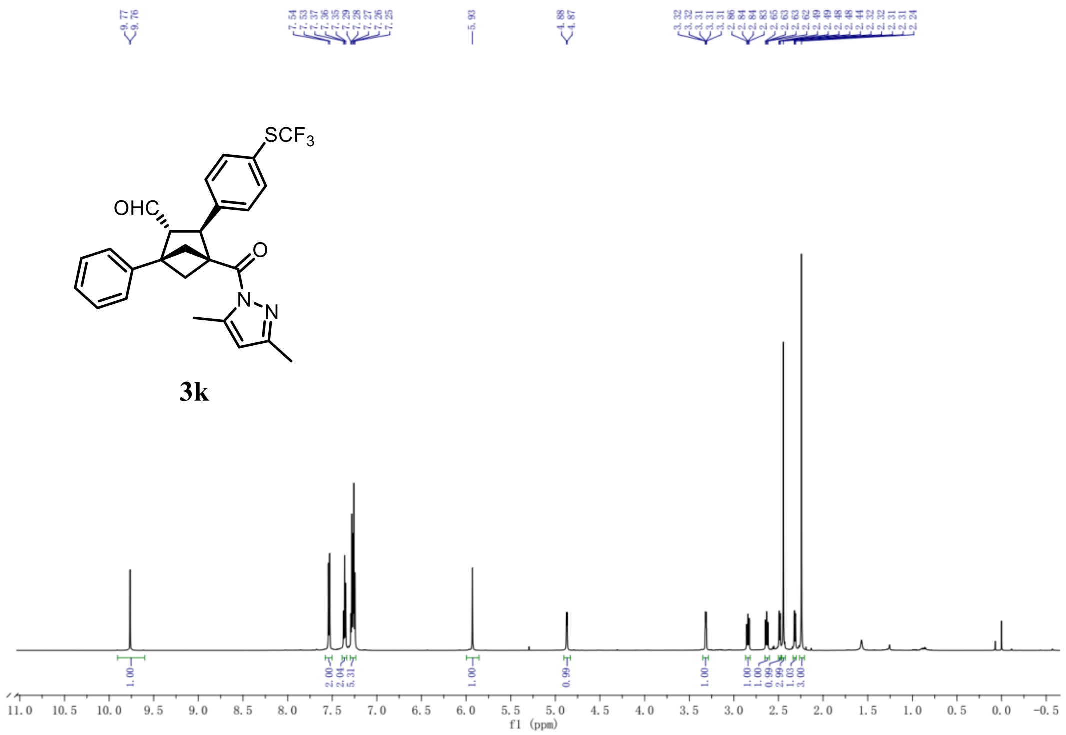

^{13}C NMR of **3k** in $CDCl_3$ (151 MHz, $CDCl_3$)

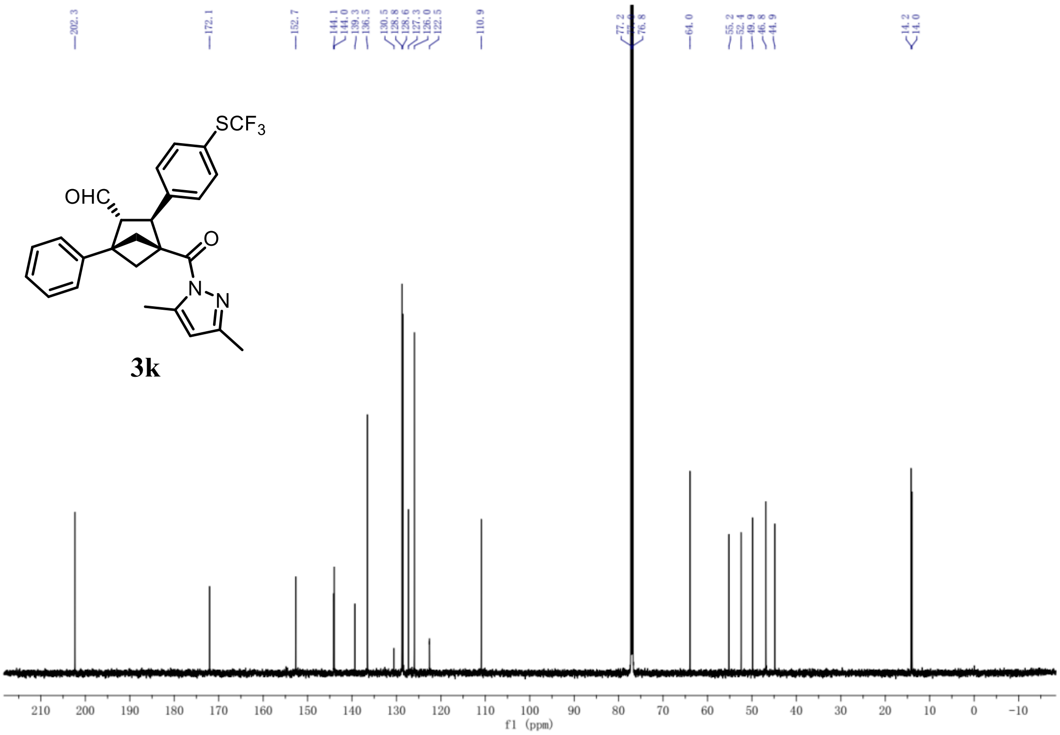

^{19}F NMR of **3k** in $CDCl_3$ (565 MHz, $CDCl_3$)

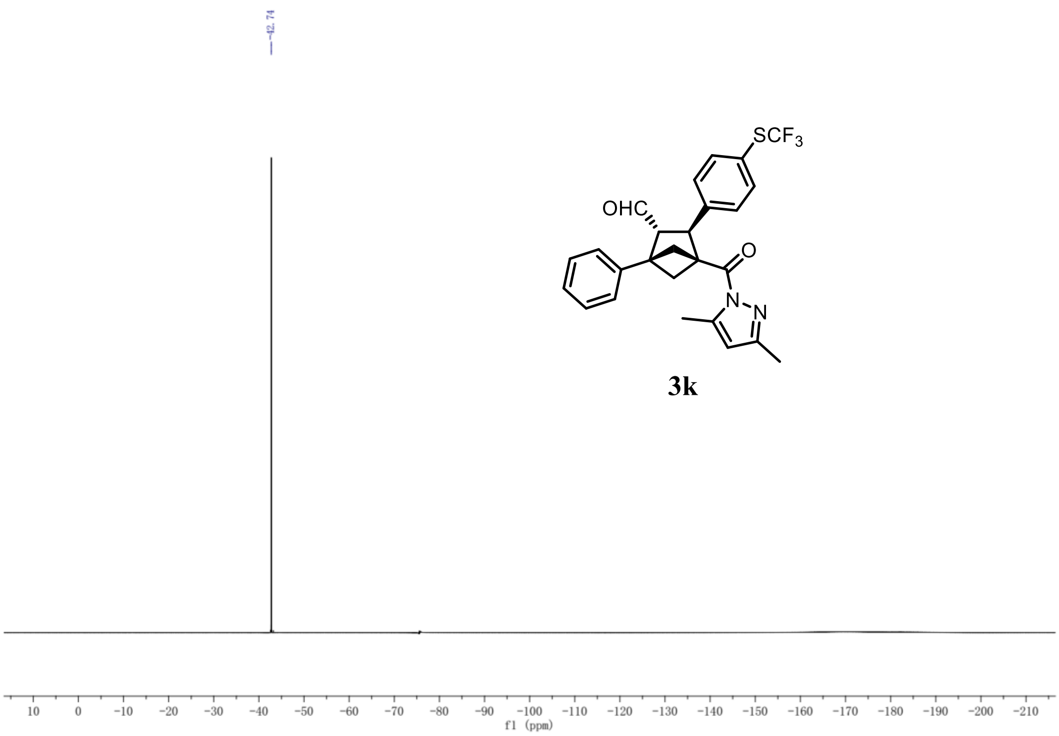

^{1}H NMR of **3l** in $CDCl_3$ (600 MHz, $CDCl_3$)

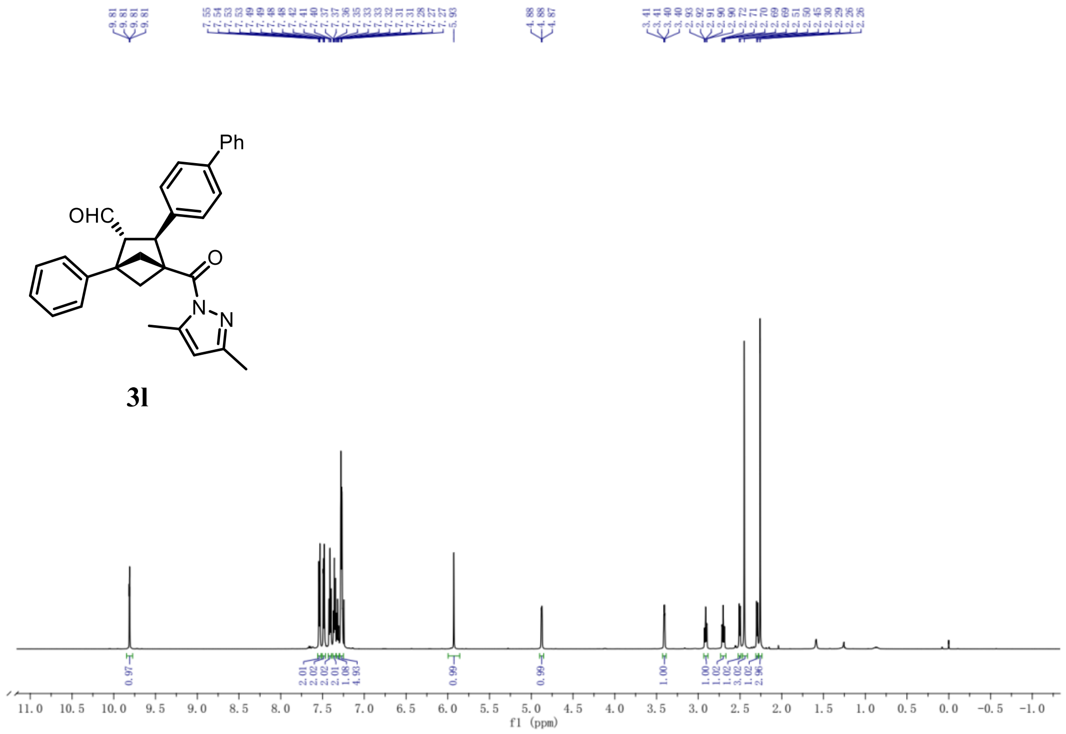

^{13}C NMR of **3l** in $CDCl_3$ (151 MHz, $CDCl_3$)

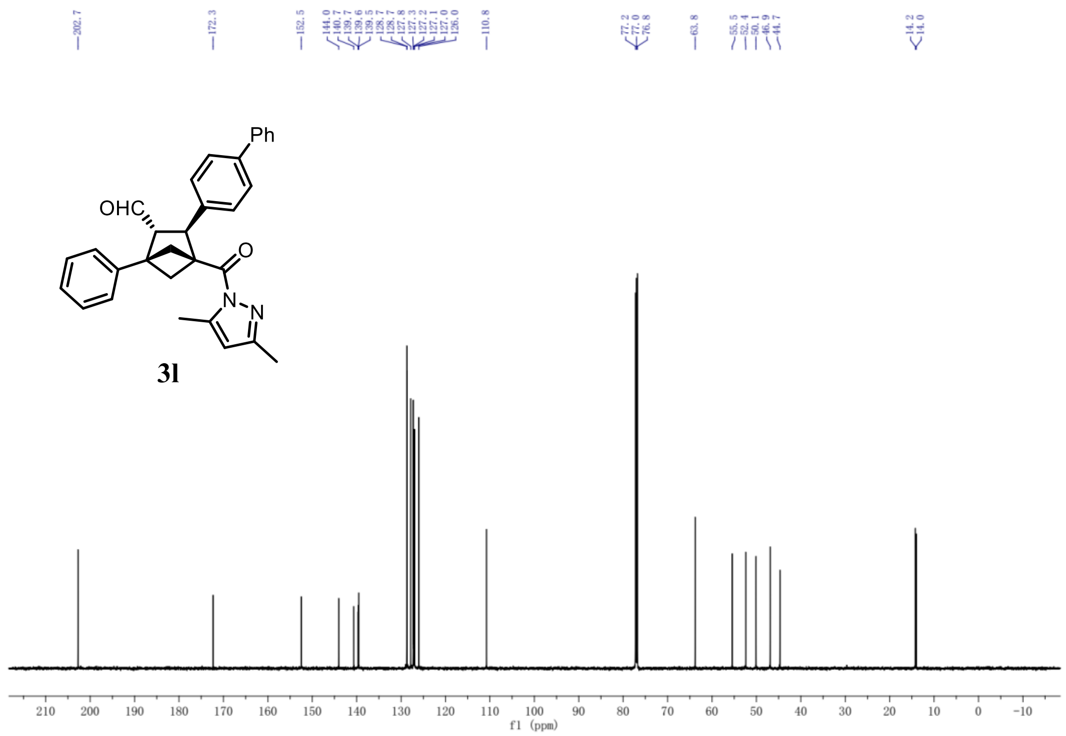

^{1}H NMR of **3m** in $CDCl_3$ (600 MHz, $CDCl_3$)

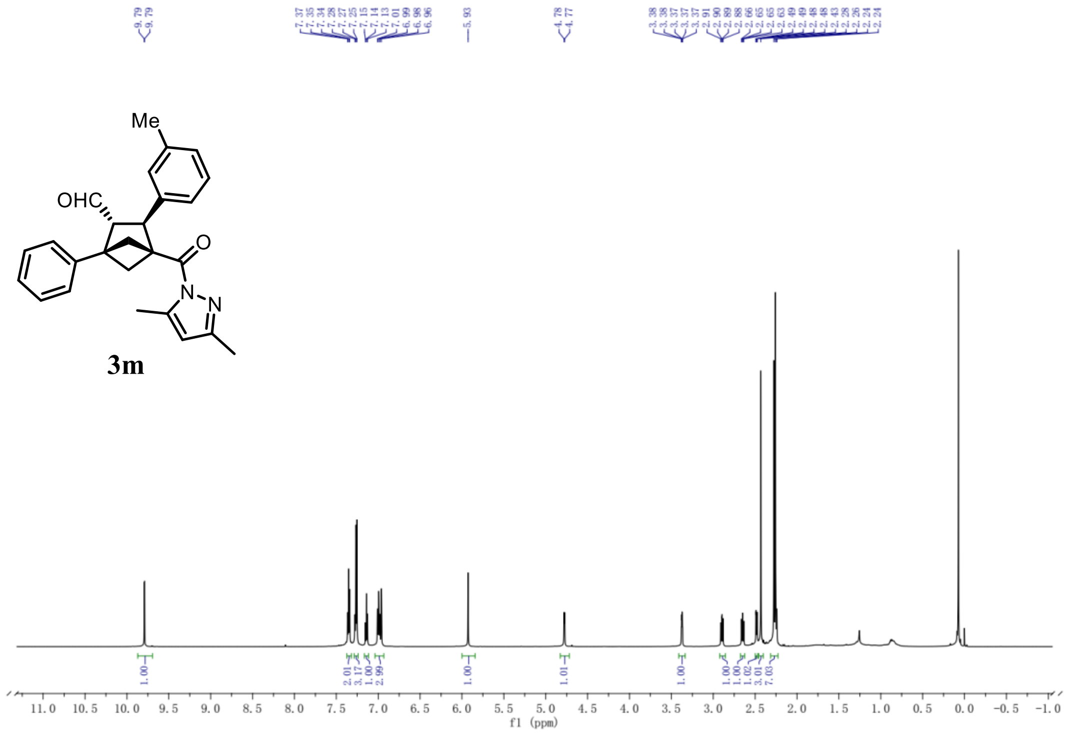

^{13}C NMR of **3m** in $CDCl_3$ (151 MHz, $CDCl_3$)

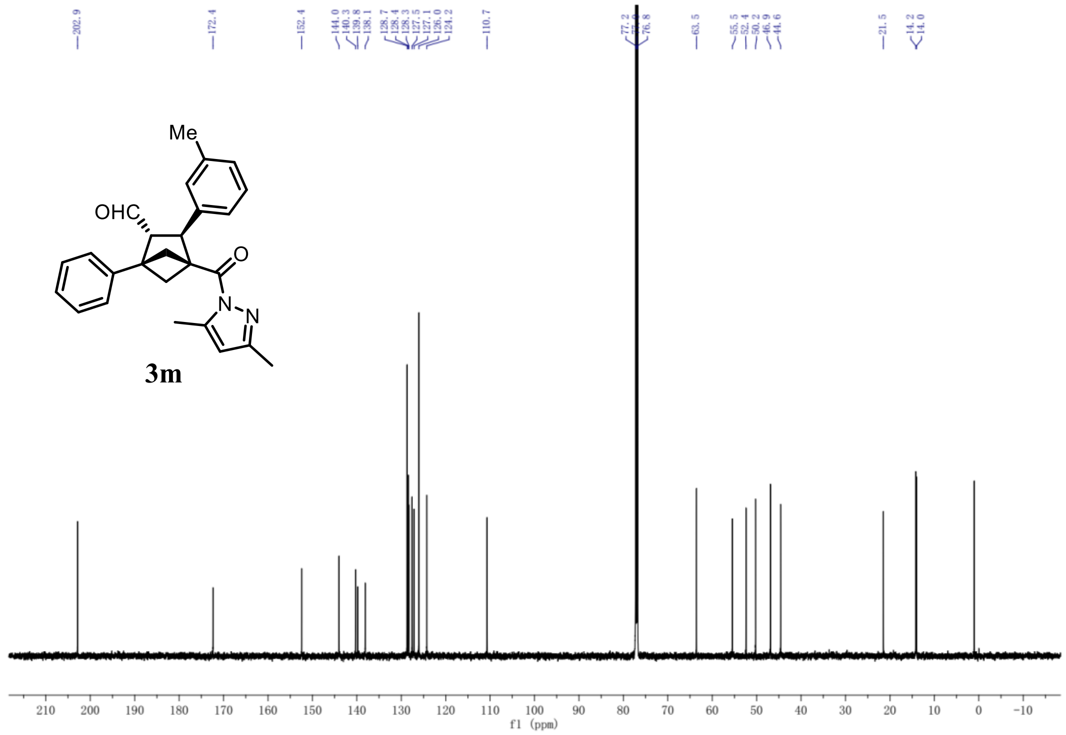

^{1}H NMR of **3n** in $CDCl_3$ (600 MHz, $CDCl_3$)

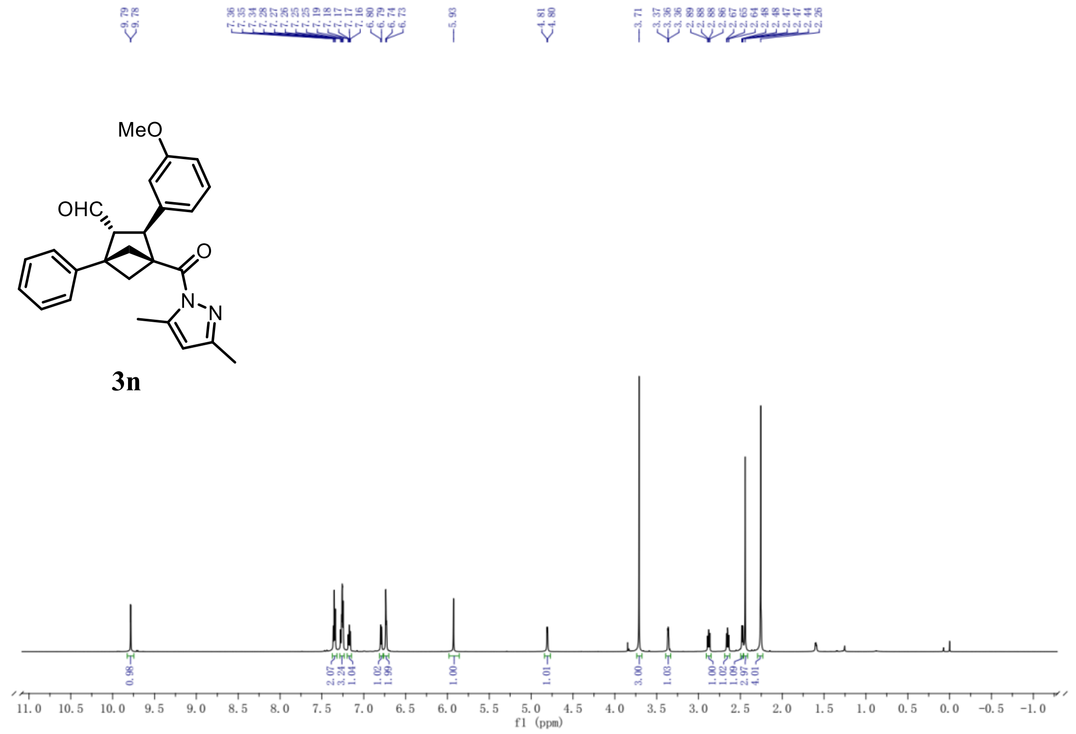

^{13}C NMR of **3n** in $CDCl_3$ (151 MHz, $CDCl_3$)

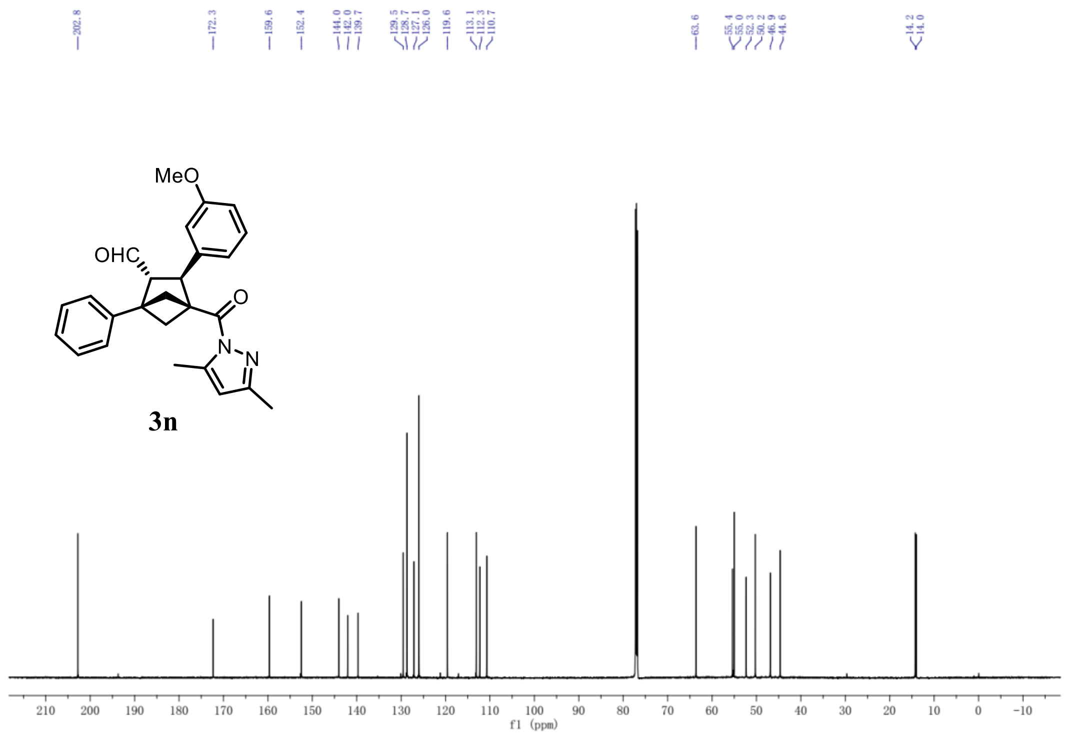

^{1}H NMR of **3o** in CDCl3 (600 MHz, CDCl3)

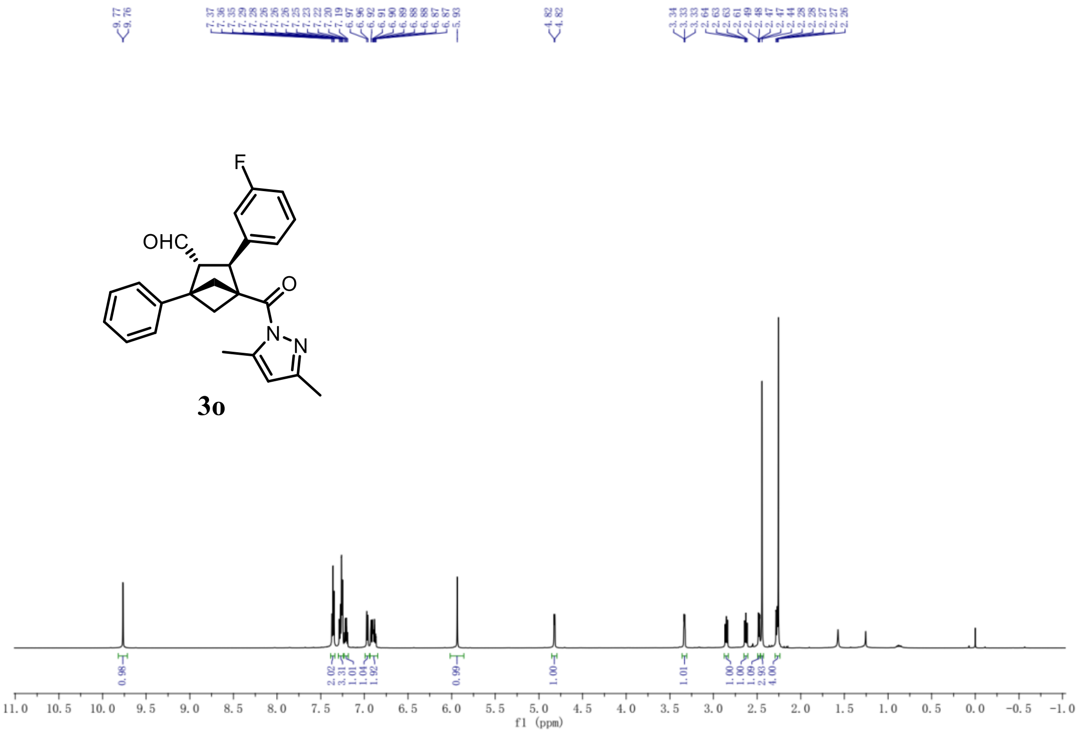

^{13}C NMR of **3o** in CDCl3 (151 MHz, CDCl3)

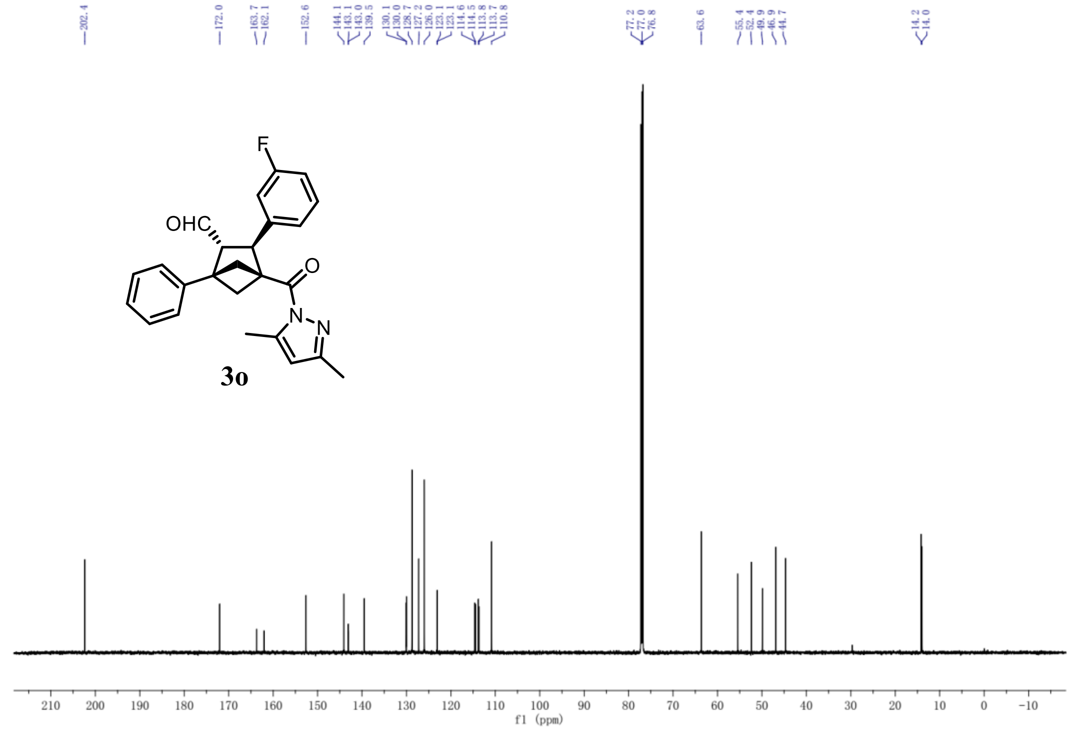

^{19}F NMR of **3o** in $CDCl_3$ (565 MHz, $CDCl_3$)

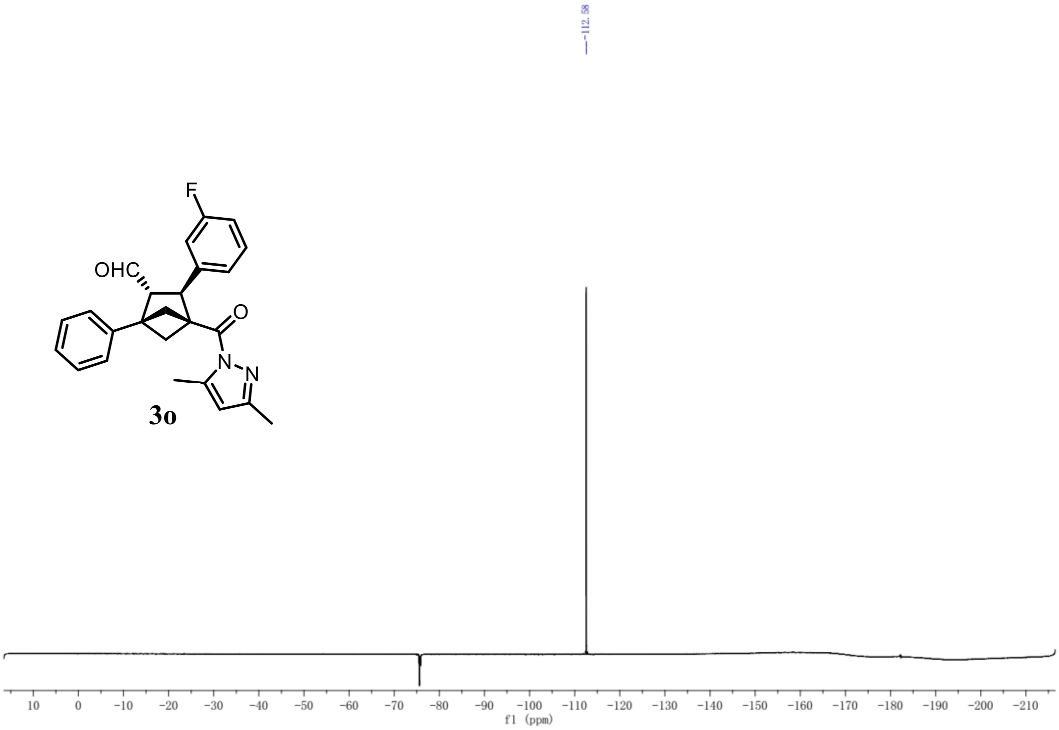

^{1}H NMR of **3p** in $CDCl_3$ (600 MHz, $CDCl_3$)

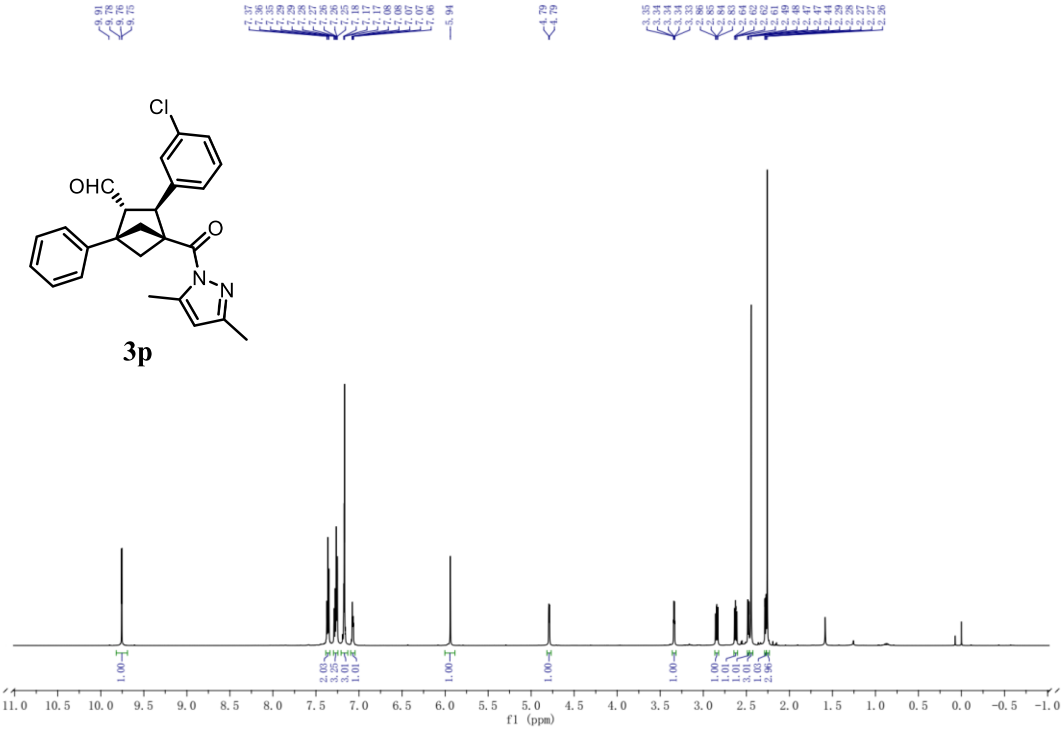

^{13}C NMR of **3p** in $CDCl_3$ (151 MHz, $CDCl_3$)

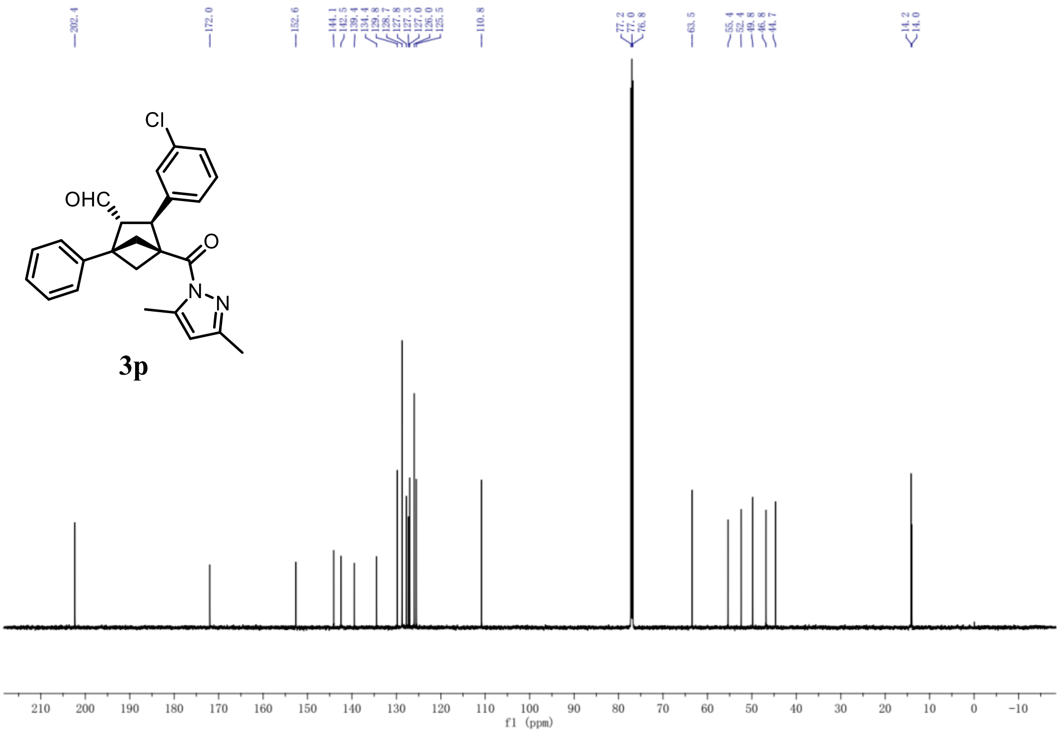

^{1}H NMR of **3q** in $CDCl_3$ (600 MHz, $CDCl_3$)

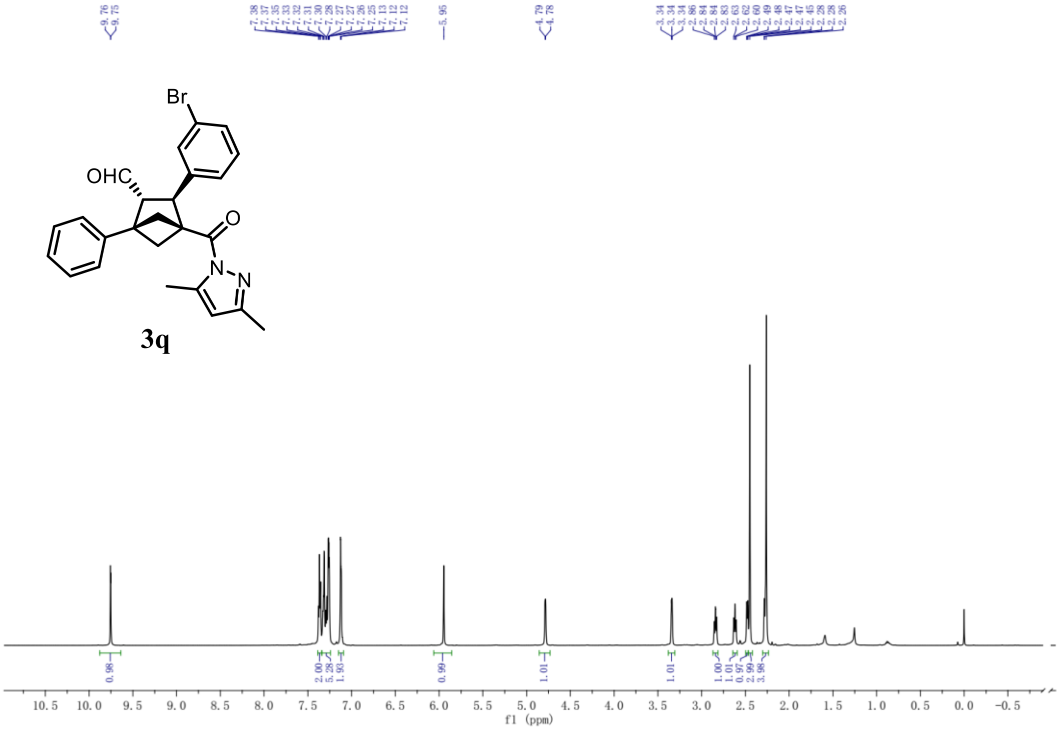

^{13}C NMR of **3q** in $CDCl_3$ (151 MHz, $CDCl_3$)

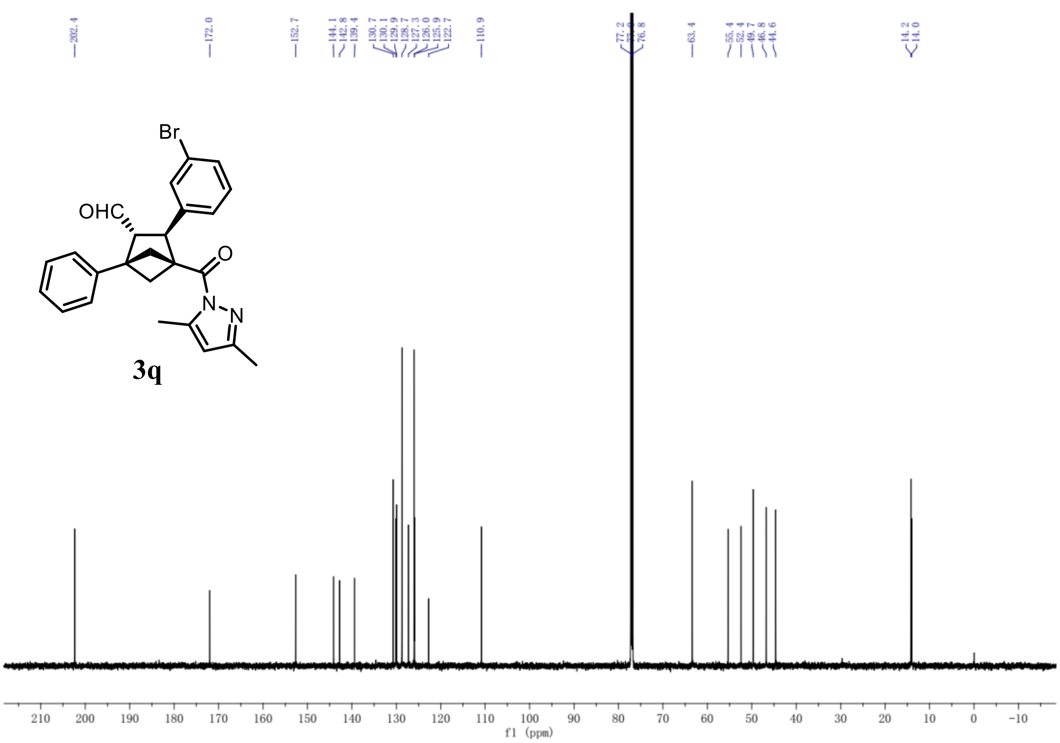

^{1}H NMR of **3r** in $CDCl_3$ (600 MHz, $CDCl_3$)

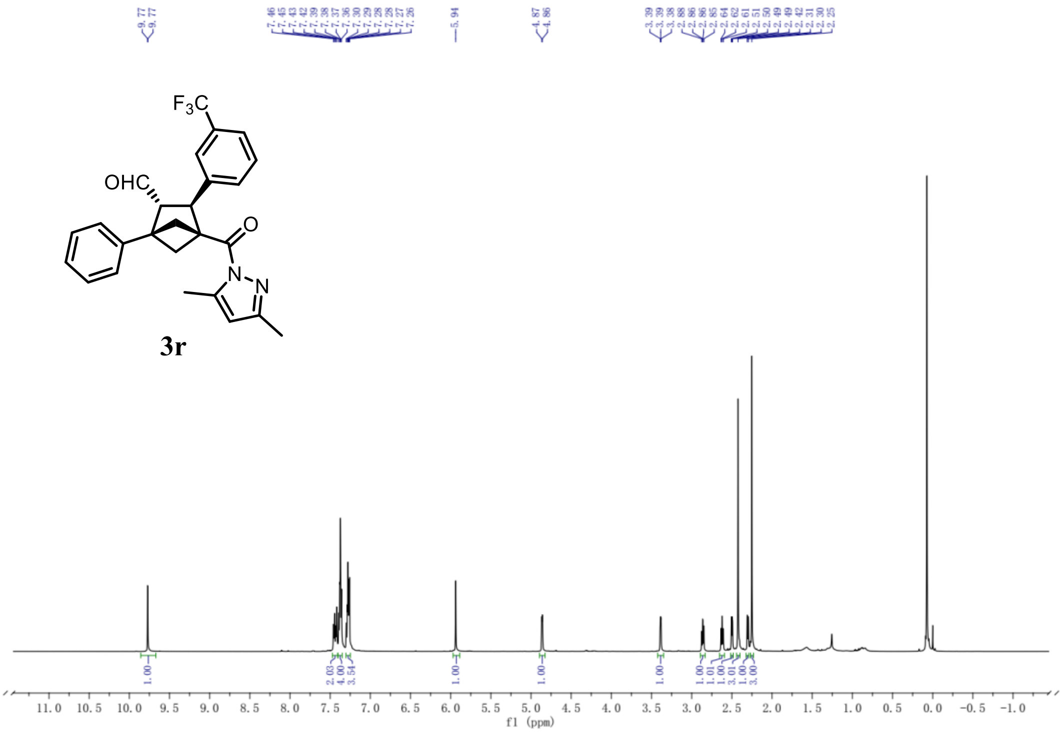

^{13}C NMR of **3r** in $CDCl_3$ (151 MHz, $CDCl_3$)

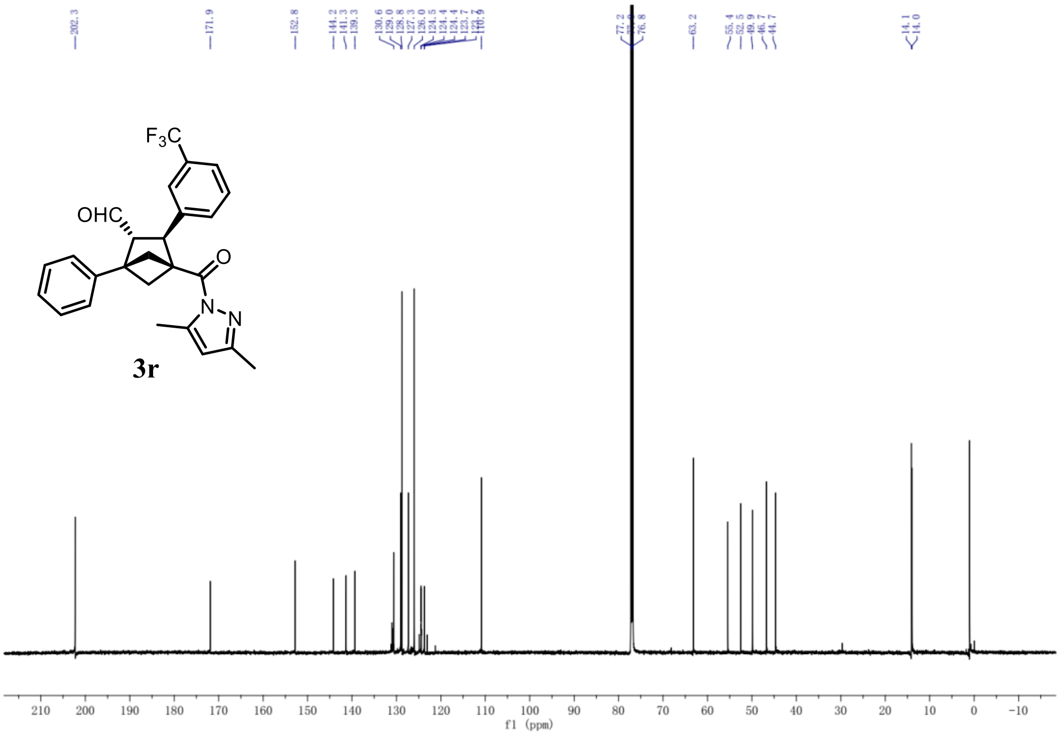

^{19}F NMR of **3r** in $CDCl_3$ (565 MHz, $CDCl_3$)

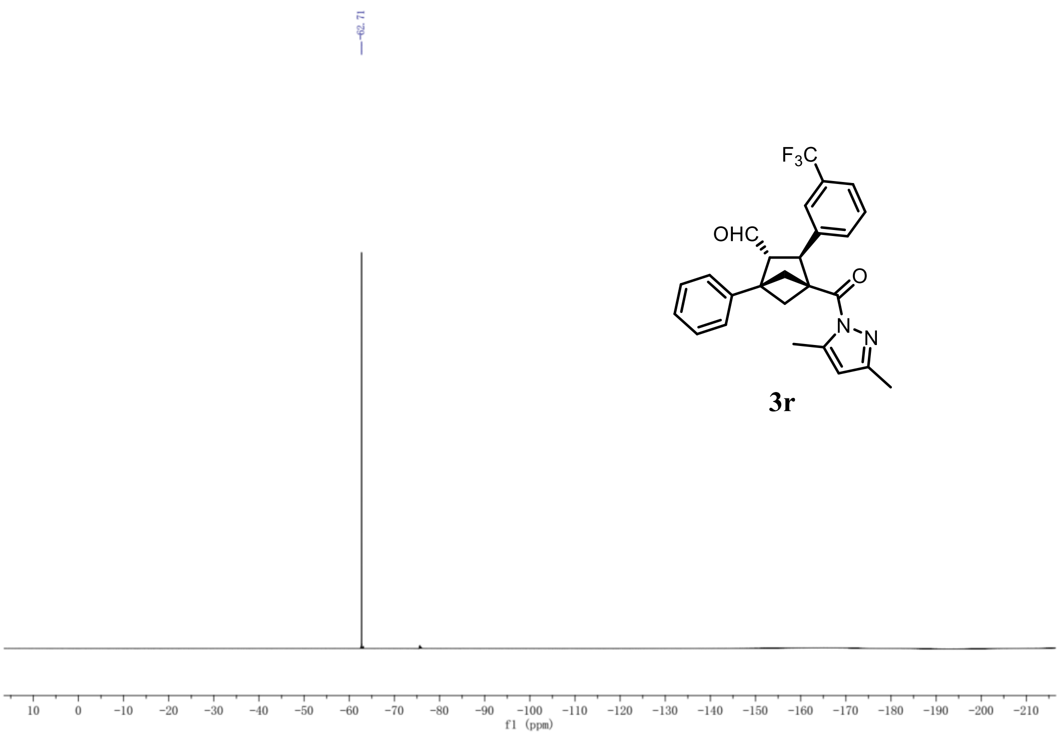

^{1}H NMR of **3s** in $CDCl_3$ (600 MHz, $CDCl_3$)

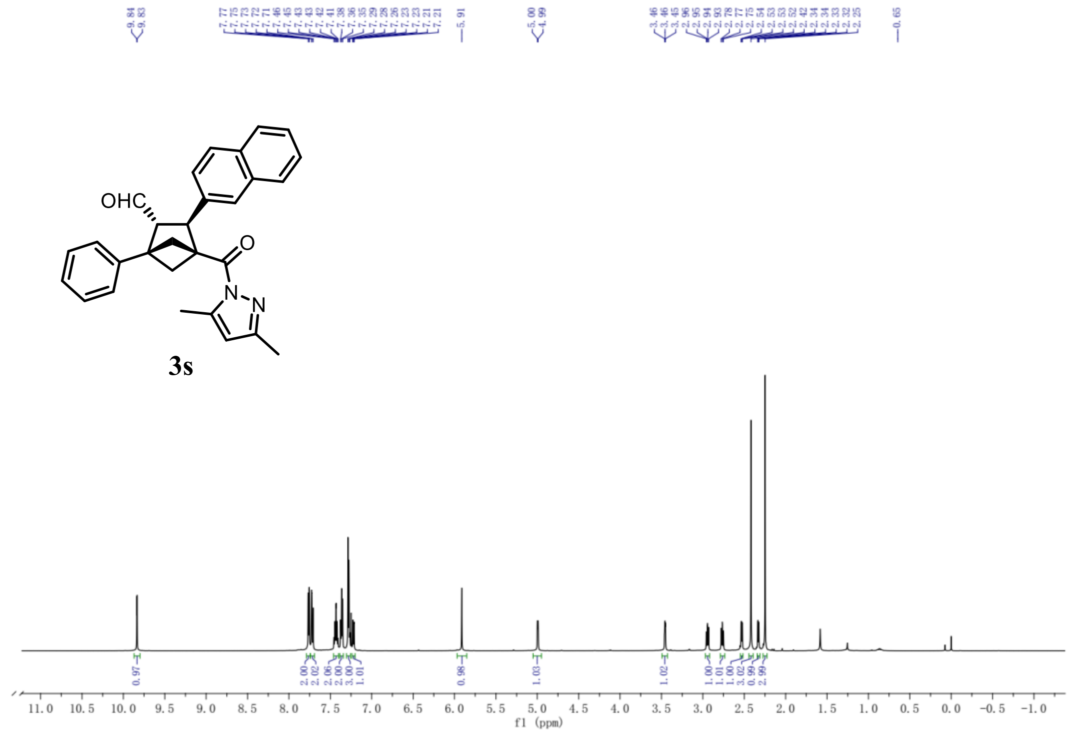

^{13}C NMR of **3s** in $CDCl_3$ (151 MHz, $CDCl_3$)

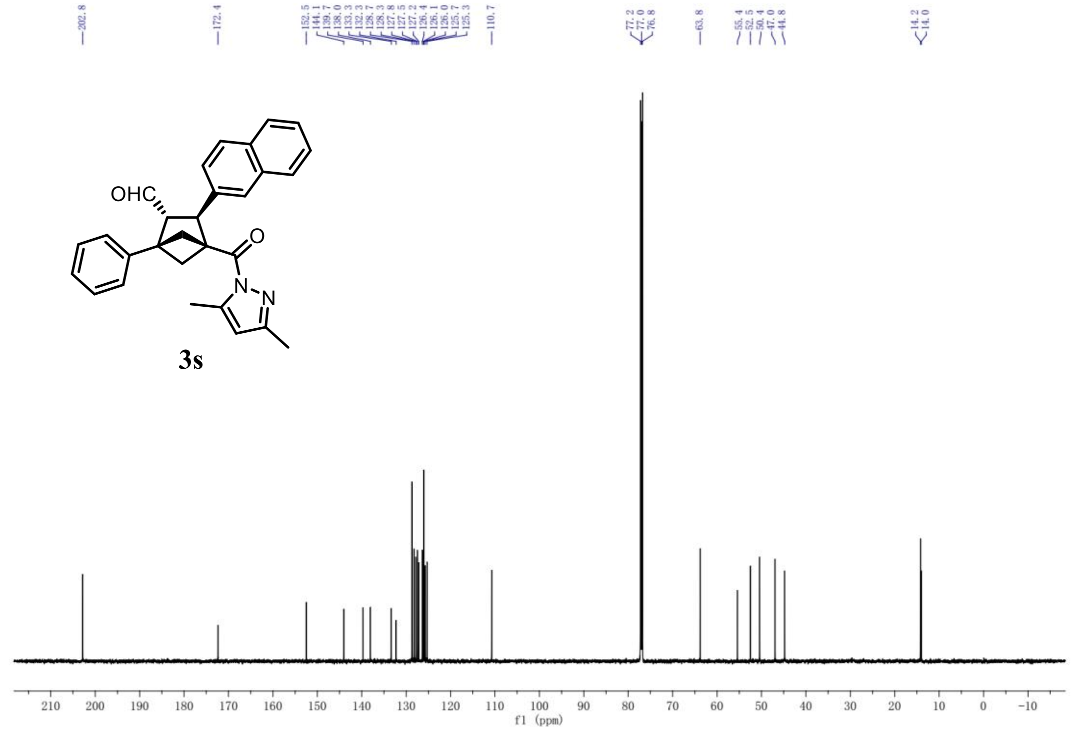

^{1}H NMR of **3t** in CDCl$_3$ (600 MHz, CDCl$_3$)

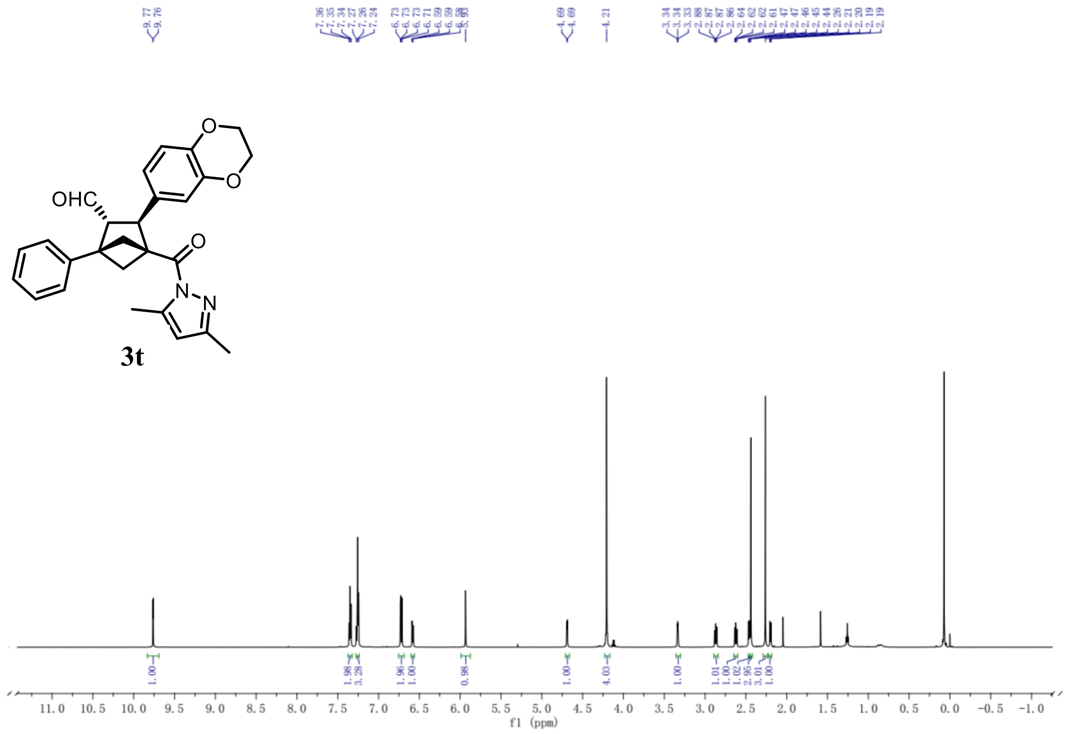

^{13}C NMR of **3t** in CDCl$_3$ (151 MHz, CDCl$_3$)

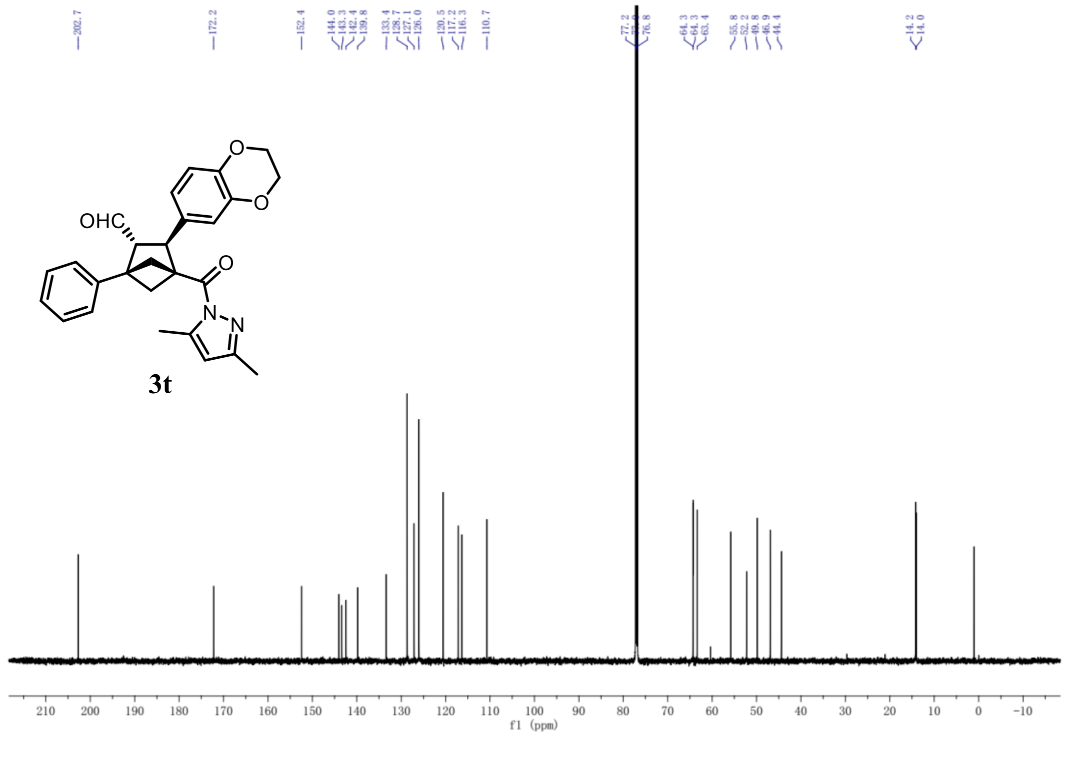

^{1}H NMR of **3u** in $CDCl_3$ (600 MHz, $CDCl_3$)

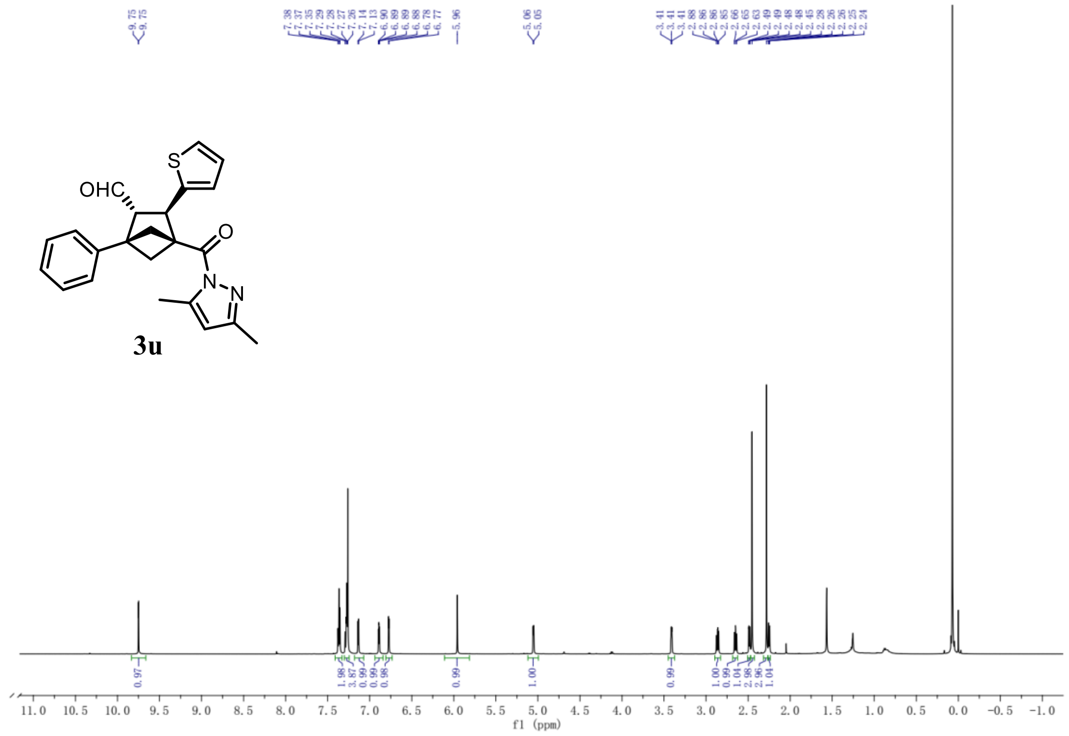

^{13}C NMR of **3u** in $CDCl_3$ (151 MHz, $CDCl_3$)

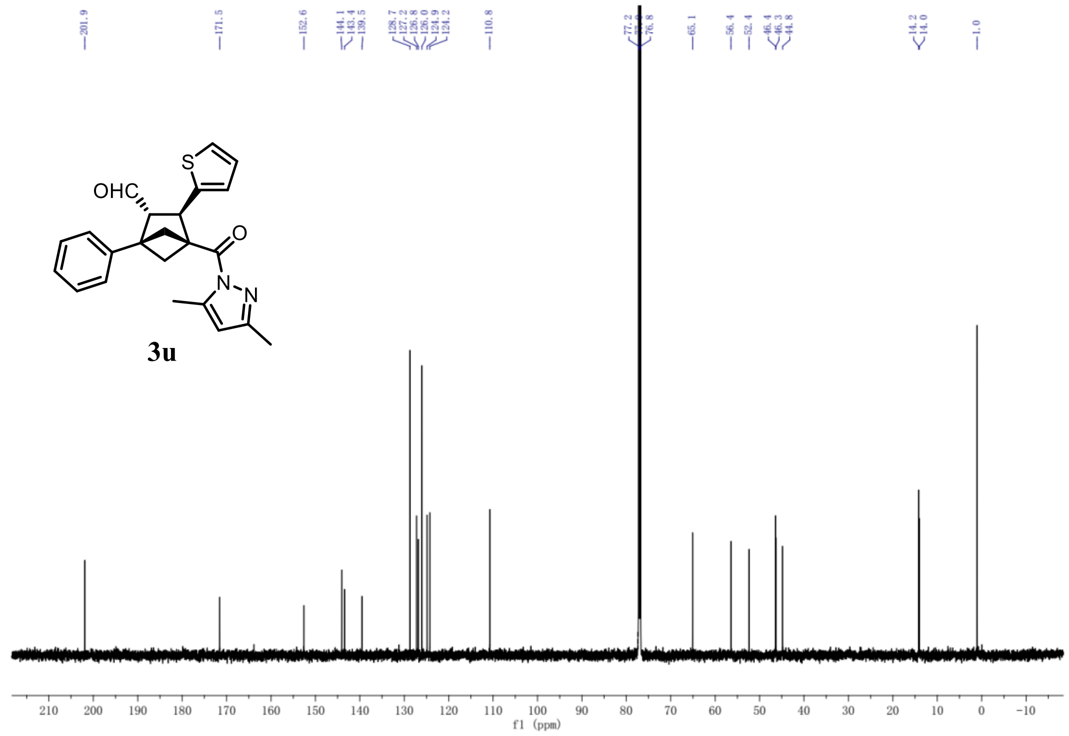

^{1}H NMR of **3v** in CDCl$_3$ (600 MHz, CDCl$_3$)

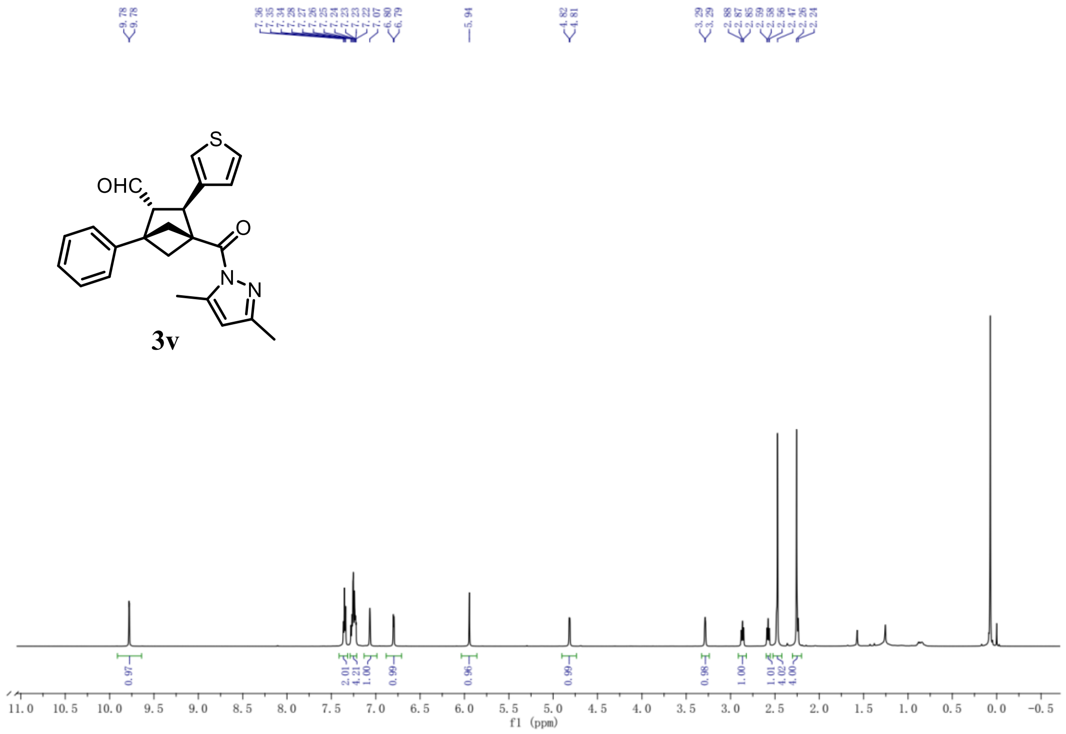

^{13}C NMR of **3v** in CDCl$_3$ (151 MHz, CDCl$_3$)

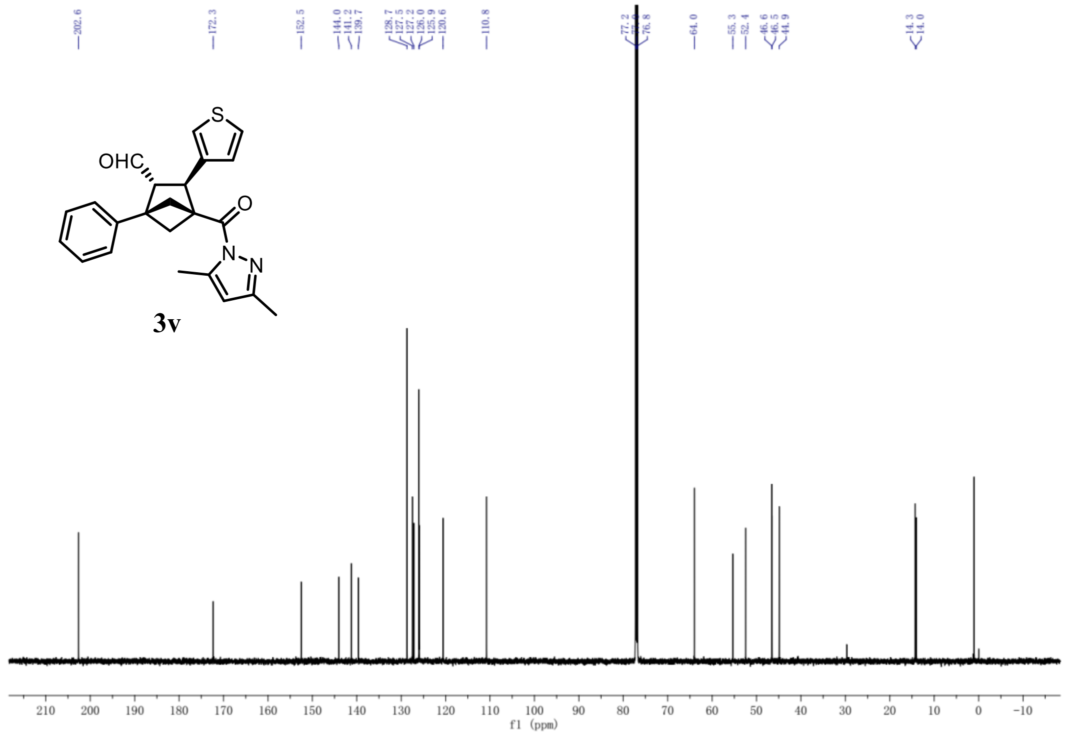

^{1}H NMR of **3w** in $CDCl_3$ (600 MHz, $CDCl_3$)

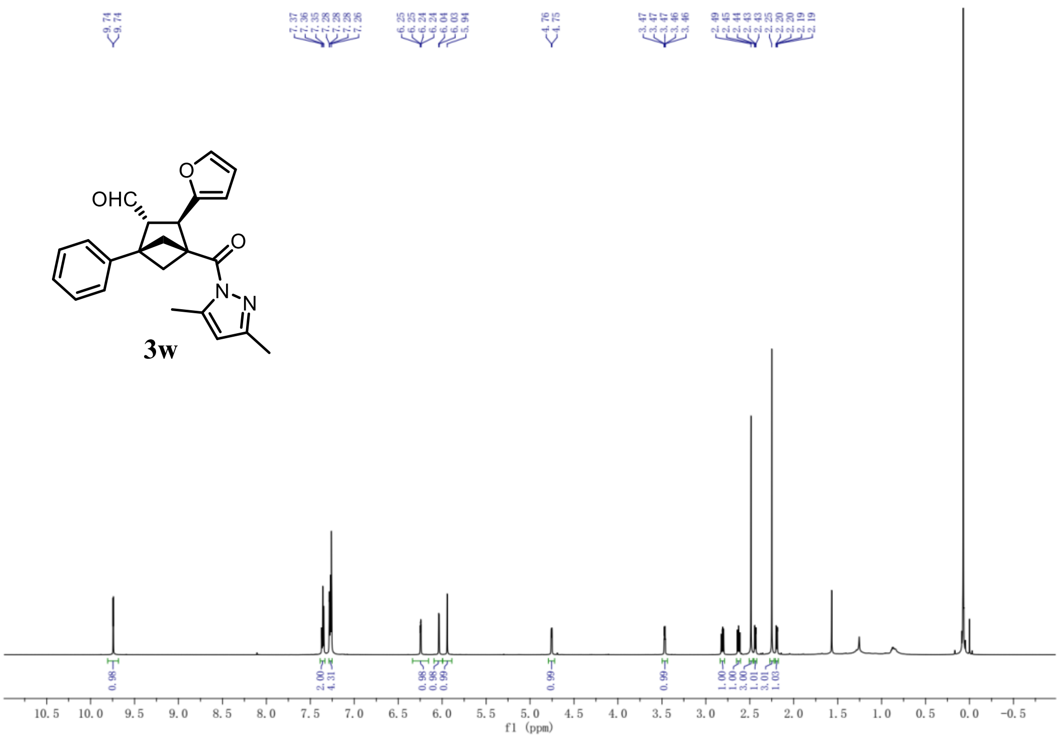

^{13}C NMR of **3w** in $CDCl_3$ (151 MHz, $CDCl_3$)

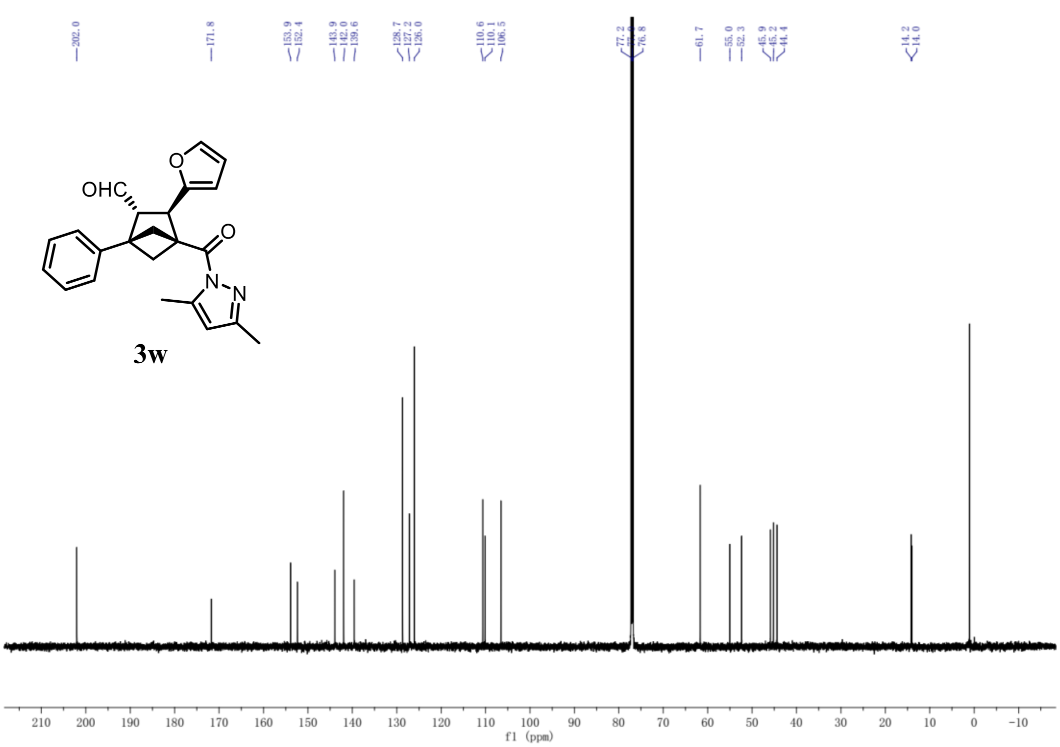

^{1}H NMR of **3x** in $CDCl_3$ (600 MHz, $CDCl_3$)

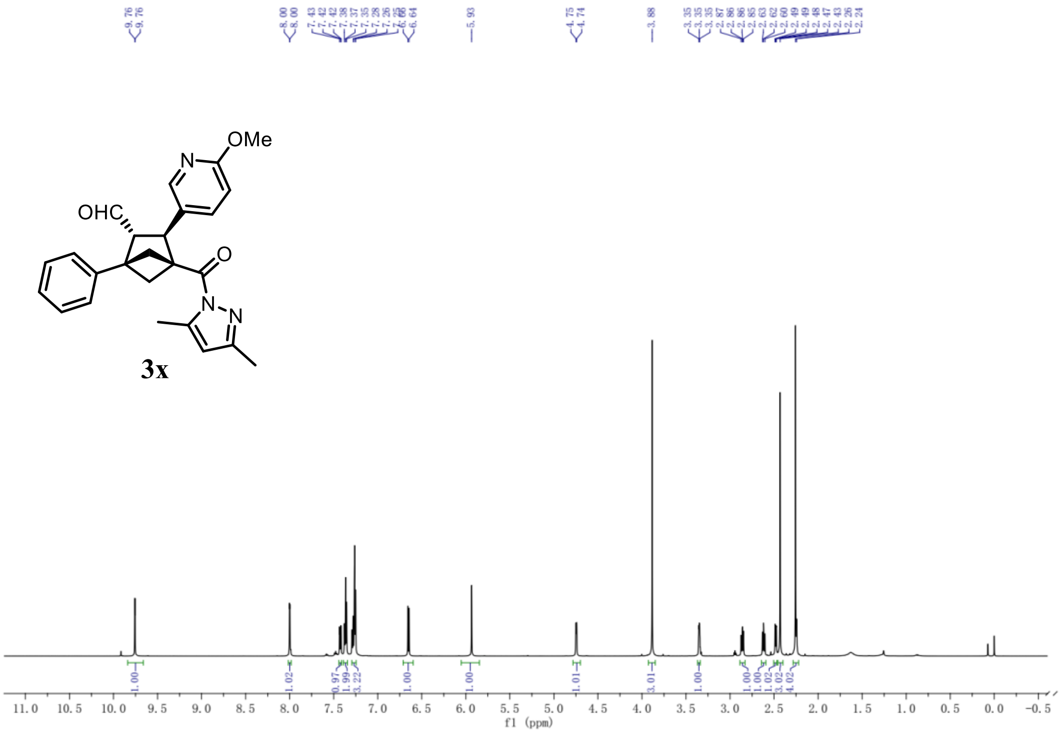

^{13}C NMR of **3x** in $CDCl_3$ (151 MHz, $CDCl_3$)

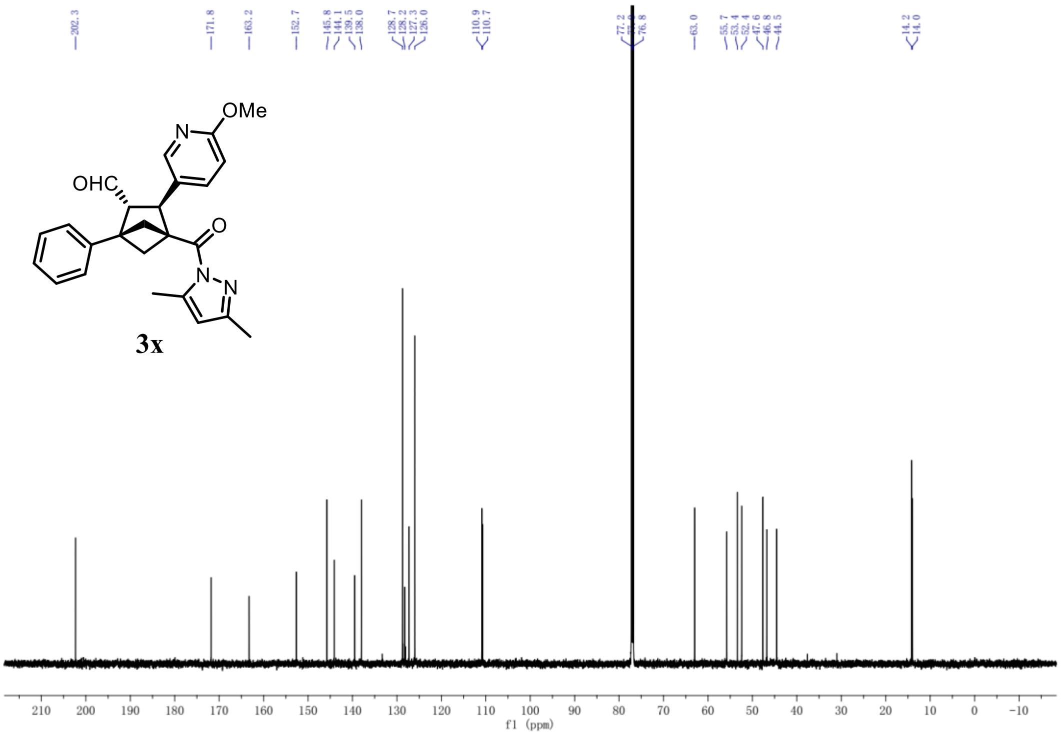

^{1}H NMR of **3y** in $CDCl_3$ (600 MHz, $CDCl_3$)

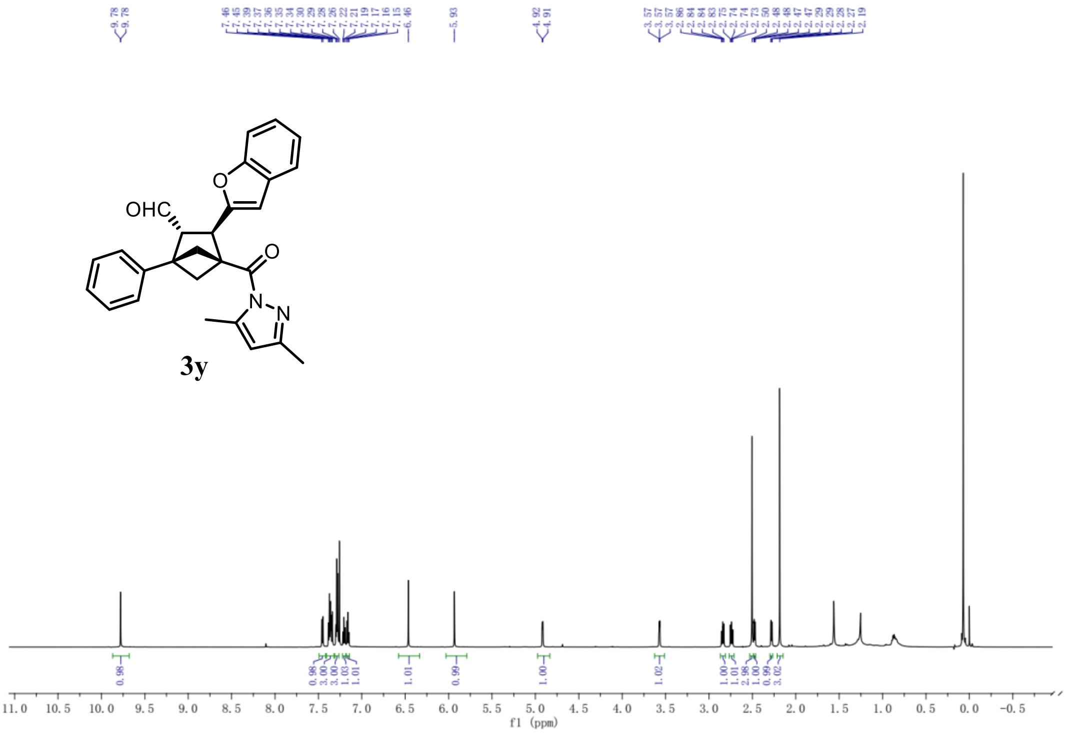

^{13}C NMR of **3y** in $CDCl_3$ (151 MHz, $CDCl_3$)

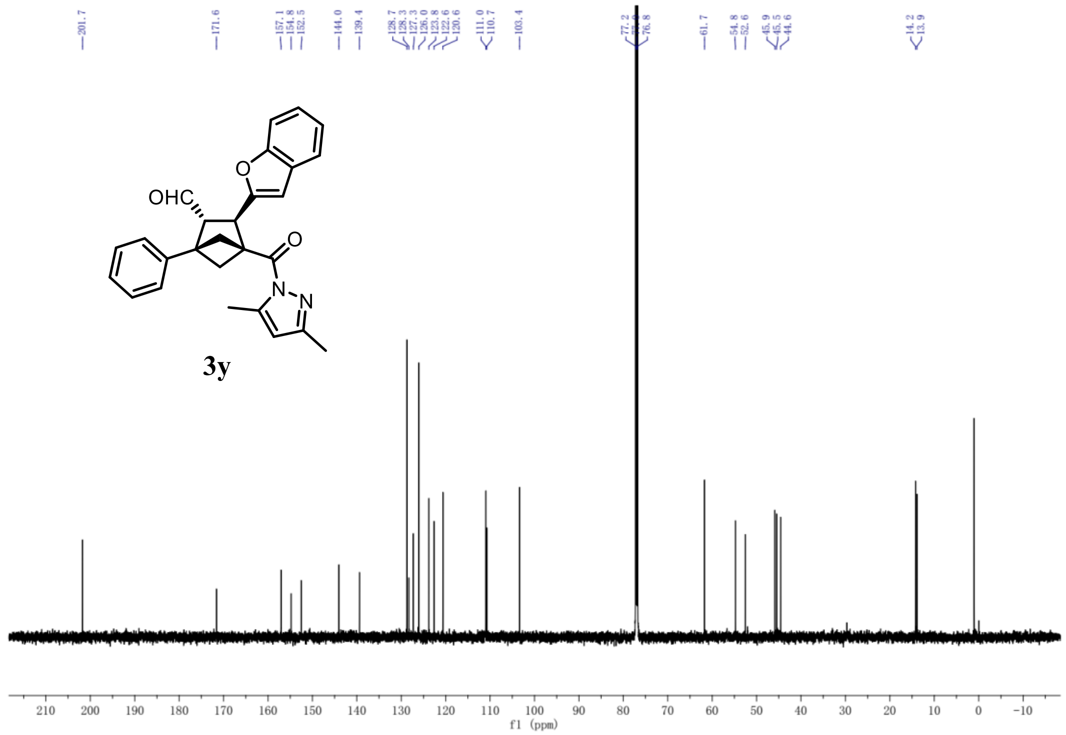

^{1}H NMR of **3zd** in $CDCl_3$ (600 MHz, $CDCl_3$)

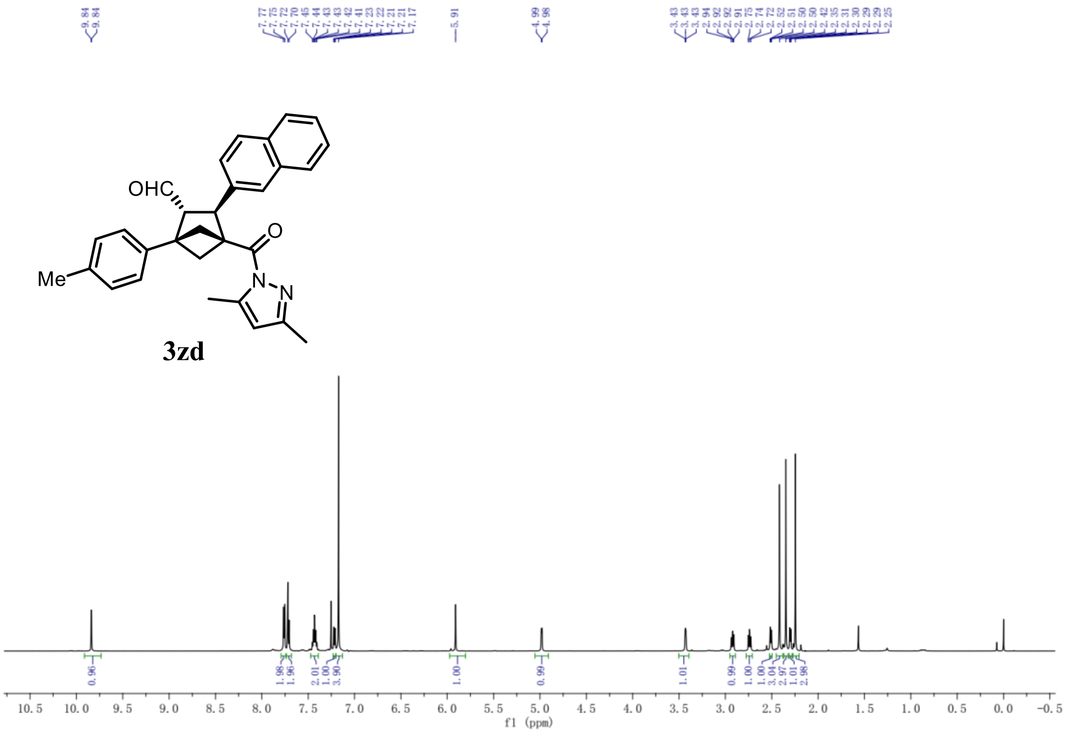

^{13}C NMR of **3zd** in $CDCl_3$ (151 MHz, $CDCl_3$)

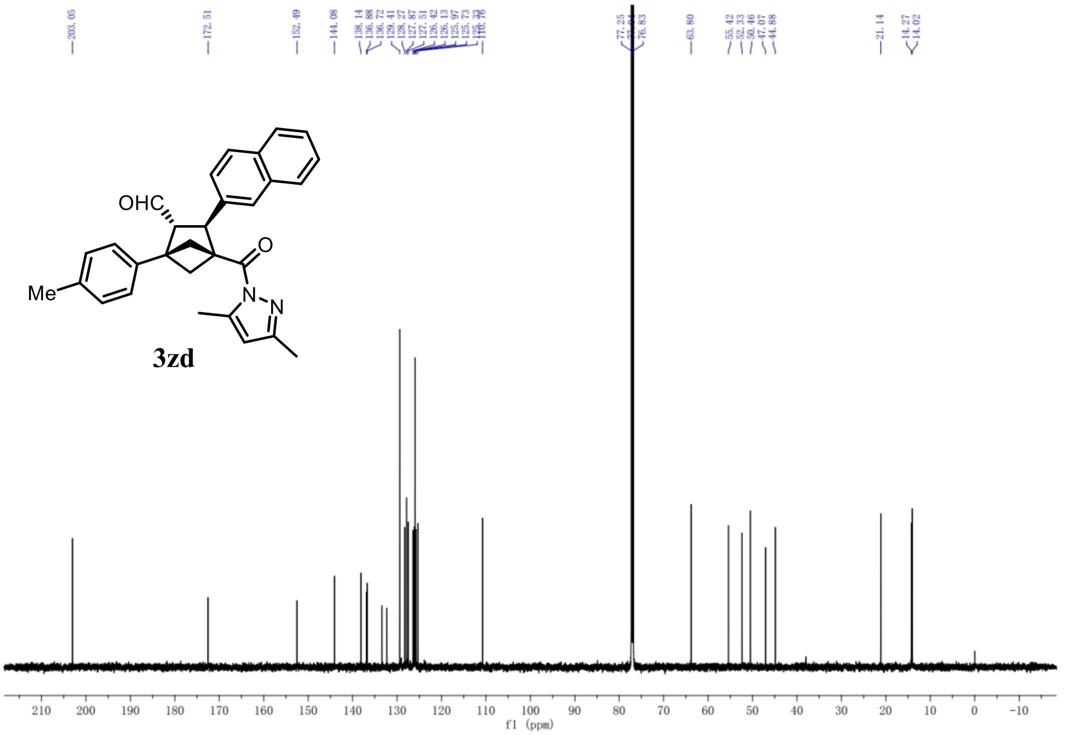

^{1}H NMR of **3ze** in $CDCl_3$ (600 MHz, $CDCl_3$)

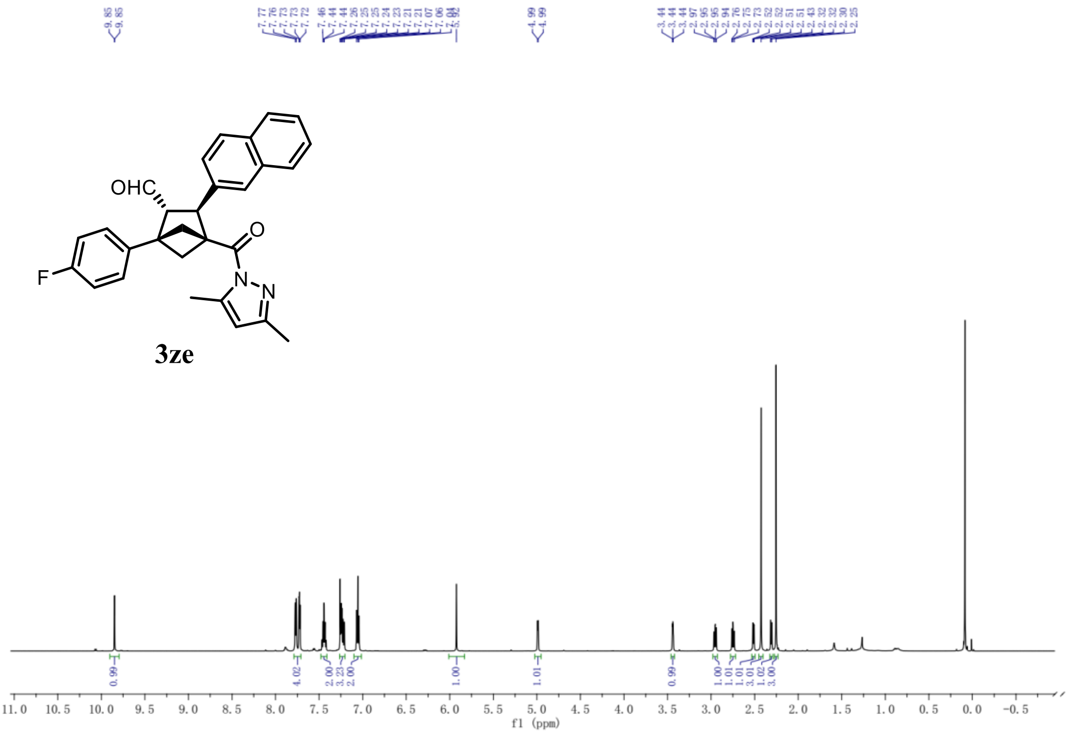

^{13}C NMR of **3ze** in $CDCl_3$ (151 MHz, $CDCl_3$)

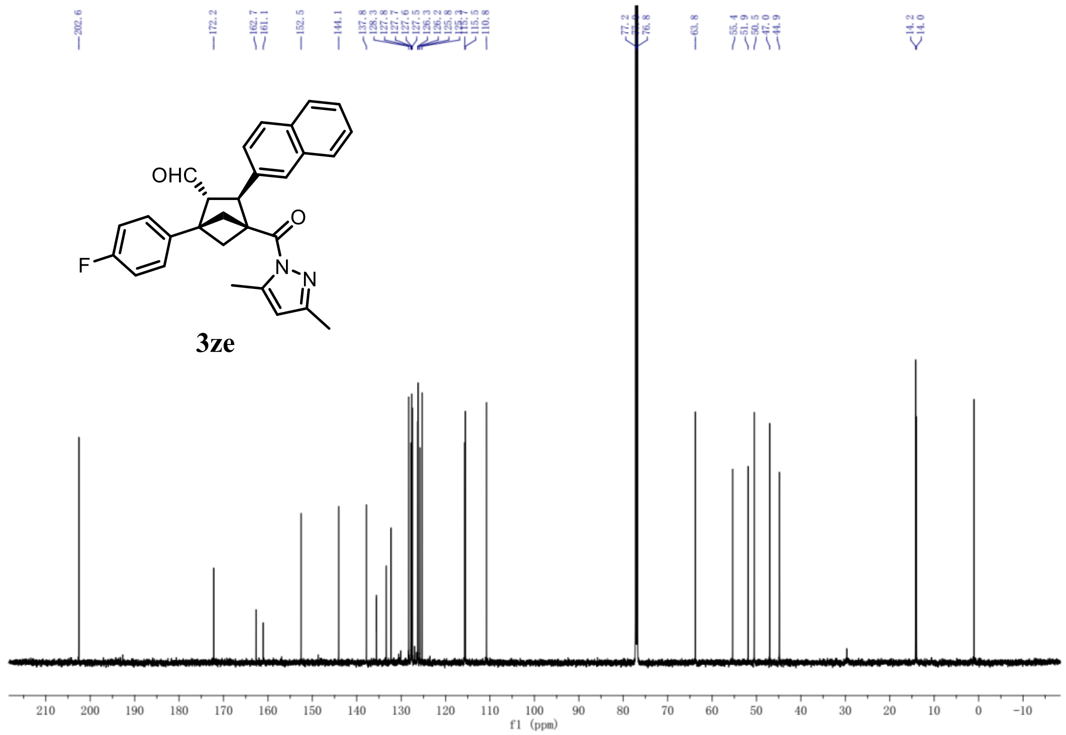

^{19}F NMR of **3ze** in $CDCl_3$ (565 MHz, $CDCl_3$)

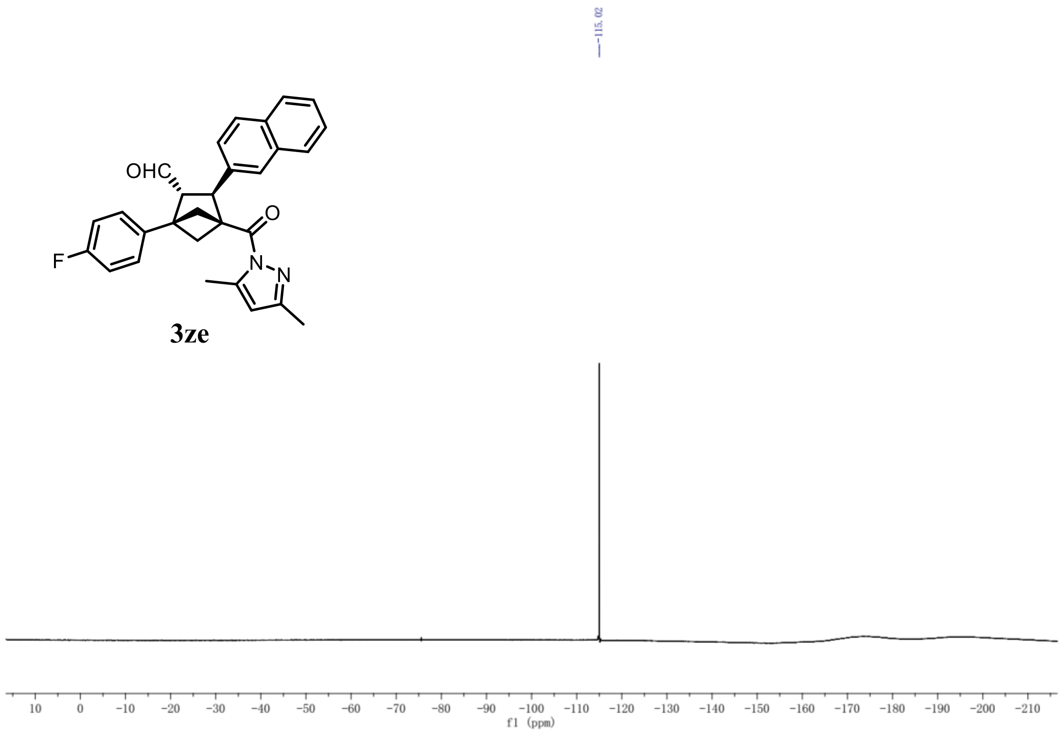

^{1}H NMR of **3zf** in $CDCl_3$ (600 MHz, $CDCl_3$)

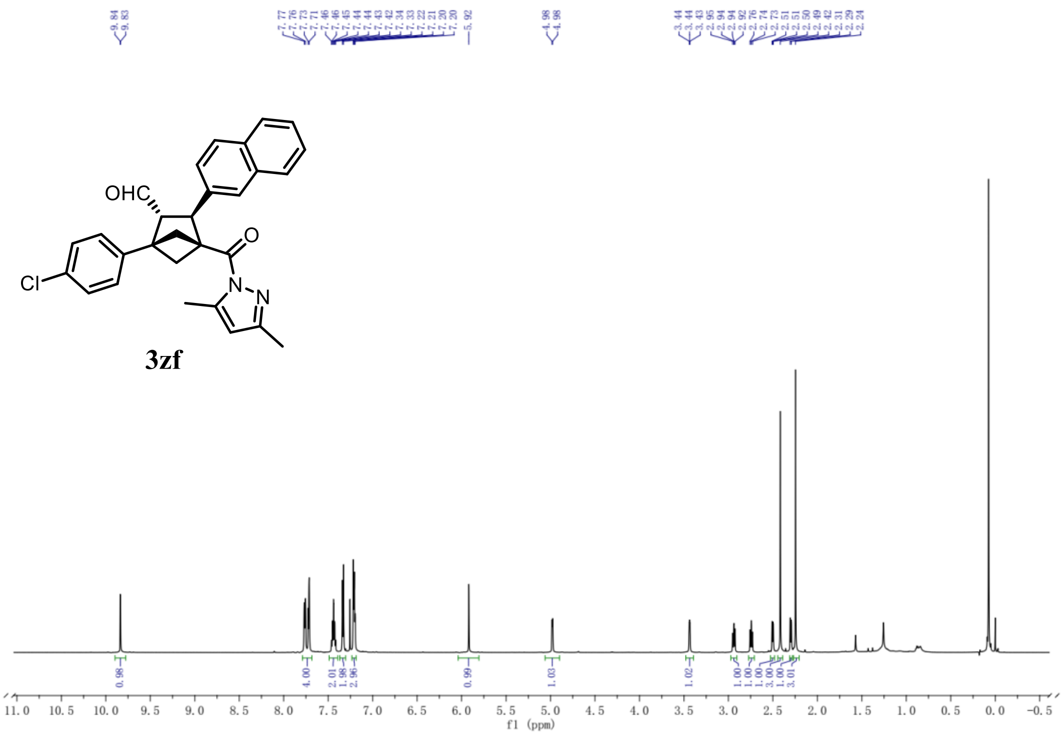

^{13}C NMR of **3zf** in $CDCl_3$ (151 MHz, $CDCl_3$)

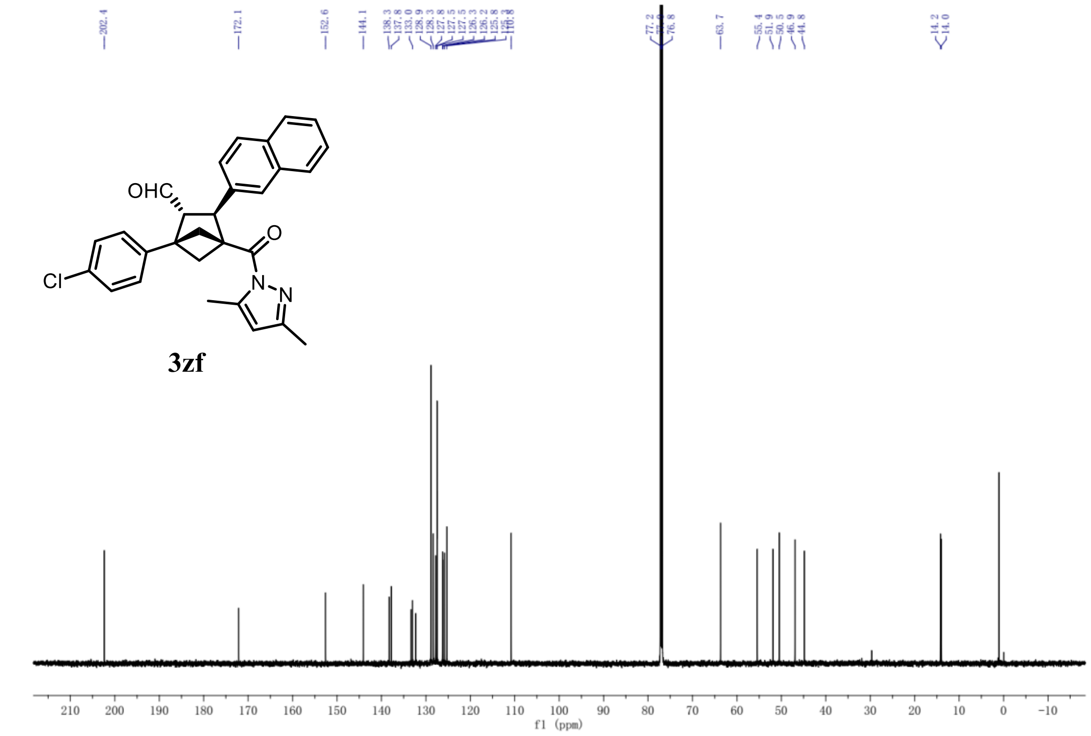

^{1}H NMR of **3zg** in $CDCl_3$ (600 MHz, $CDCl_3$)

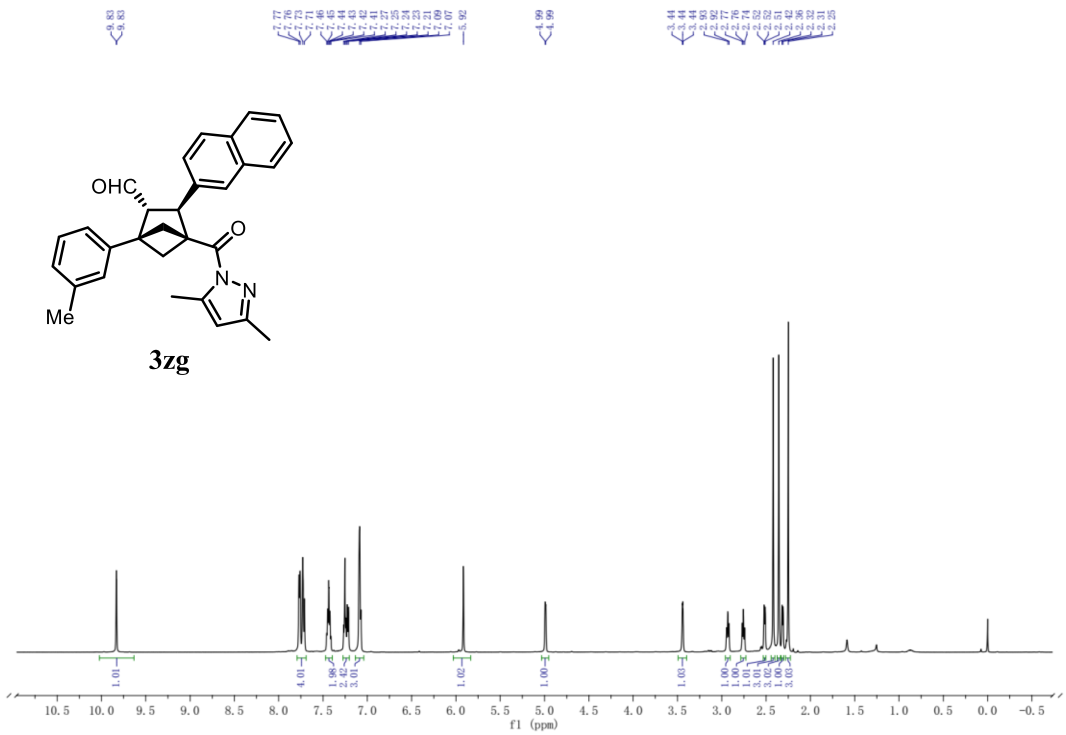

^{13}C NMR of **3zg** in $CDCl_3$ (151 MHz, $CDCl_3$)

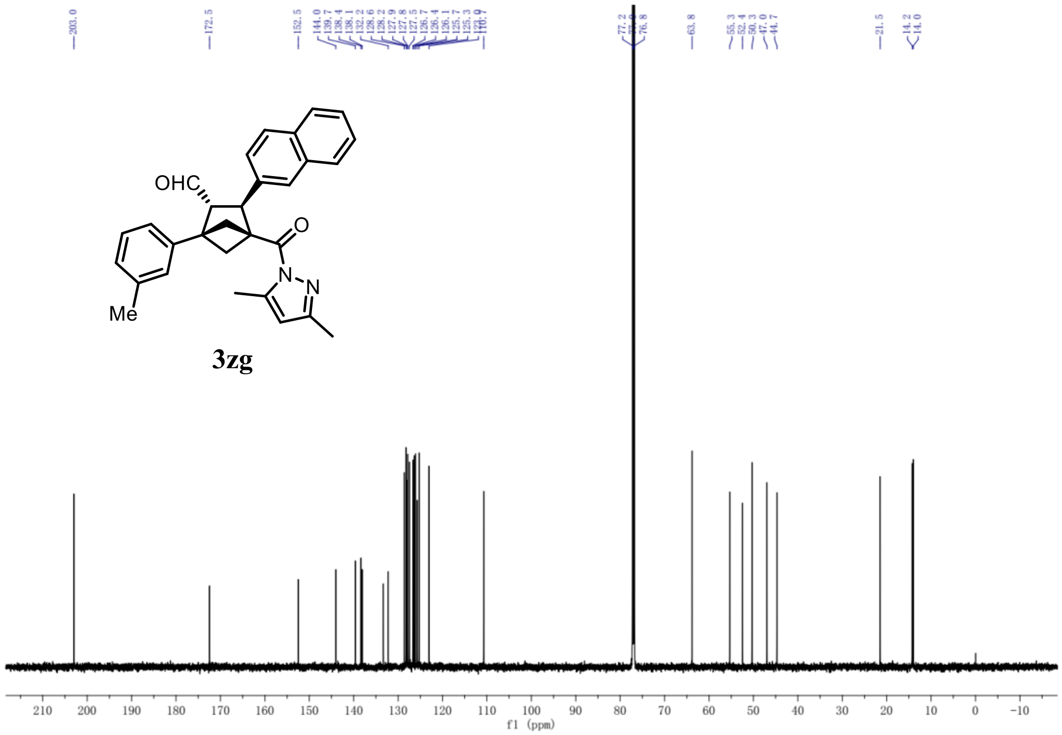

^{1}H NMR of **3zh** in $CDCl_3$ (600 MHz, $CDCl_3$)

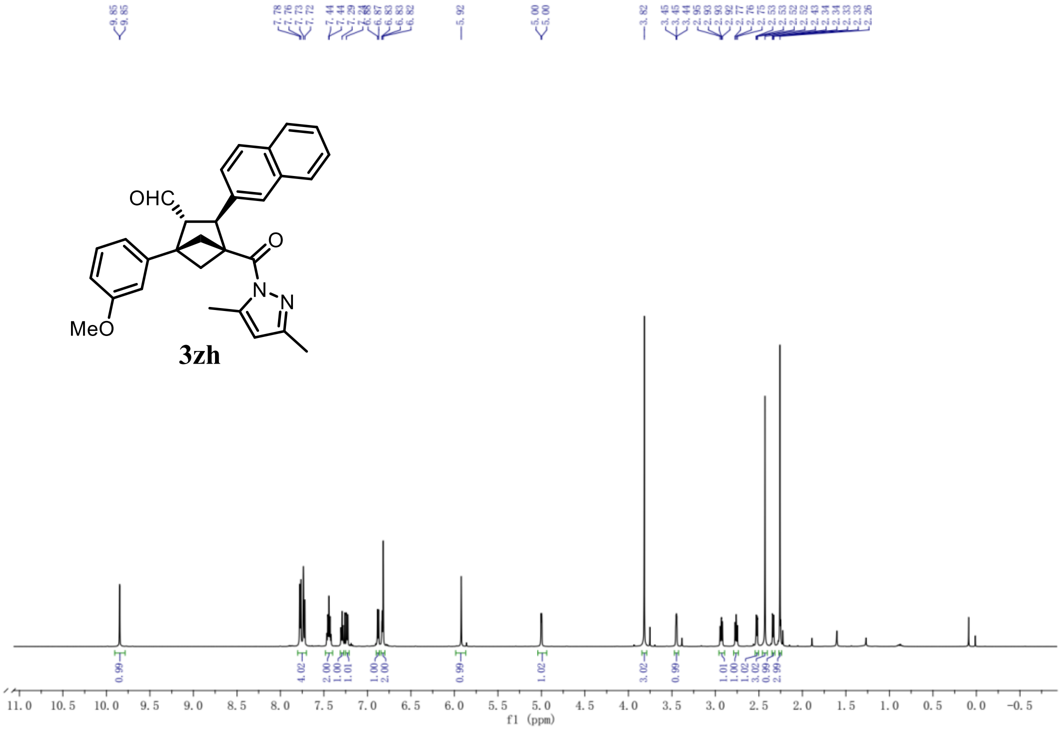

^{13}C NMR of **3zh** in $CDCl_3$ (151 MHz, $CDCl_3$)

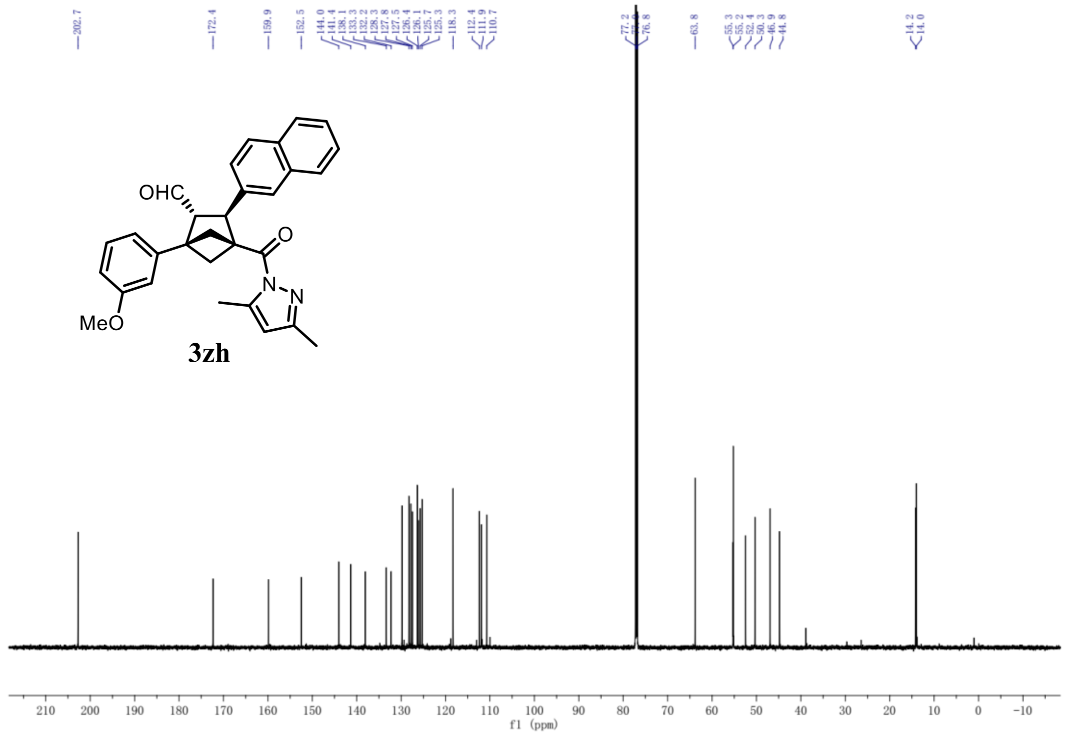

^{1}H NMR of **3zi** in $CDCl_3$ (600 MHz, $CDCl_3$)

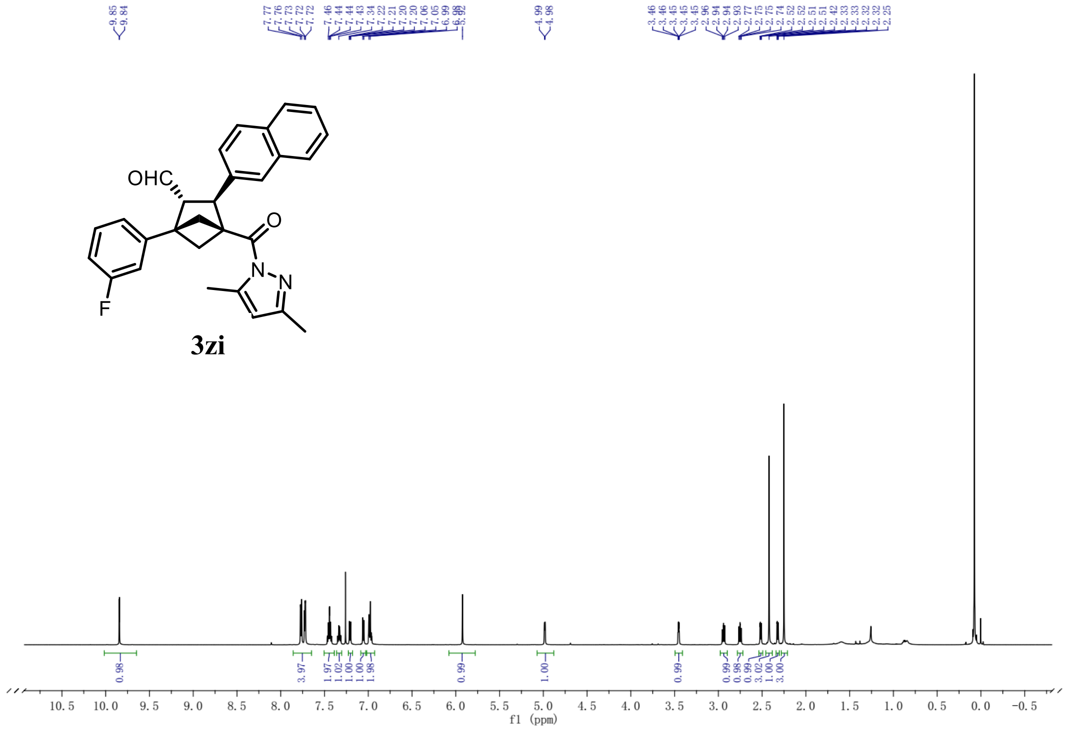

^{13}C NMR of **3zi** in $CDCl_3$ (151 MHz, $CDCl_3$)

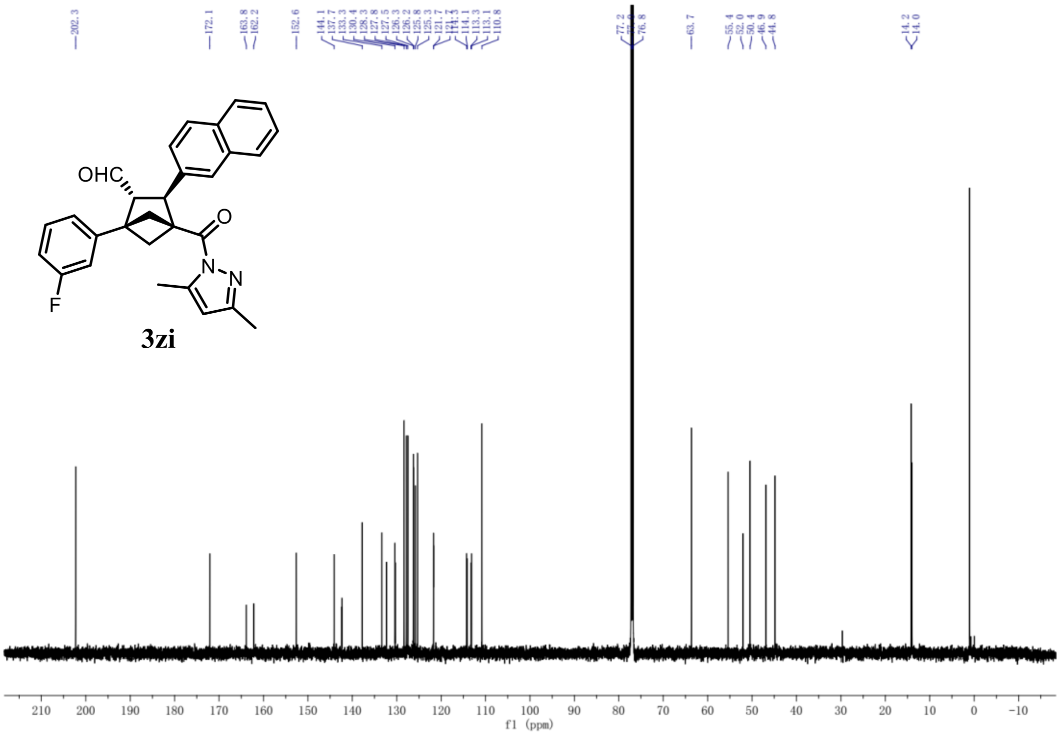

^{19}F NMR of **3zi** in $CDCl_3$ (565 MHz, $CDCl_3$)

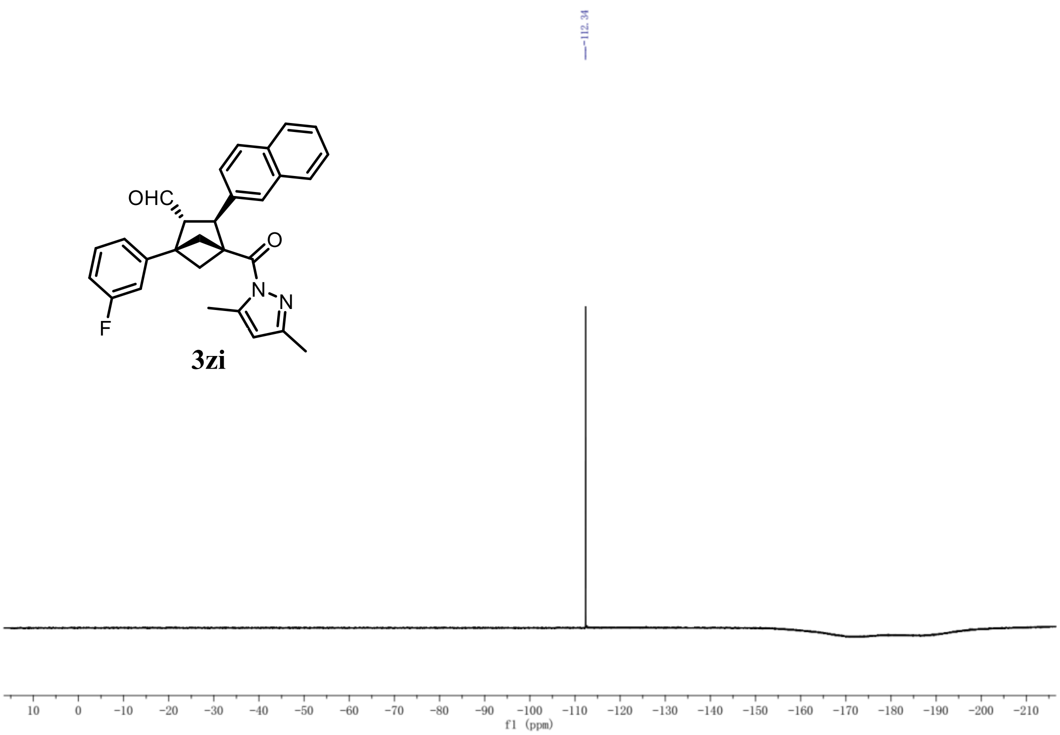

^{1}H NMR of **6** in $CDCl_3$ (600 MHz, $CDCl_3$)

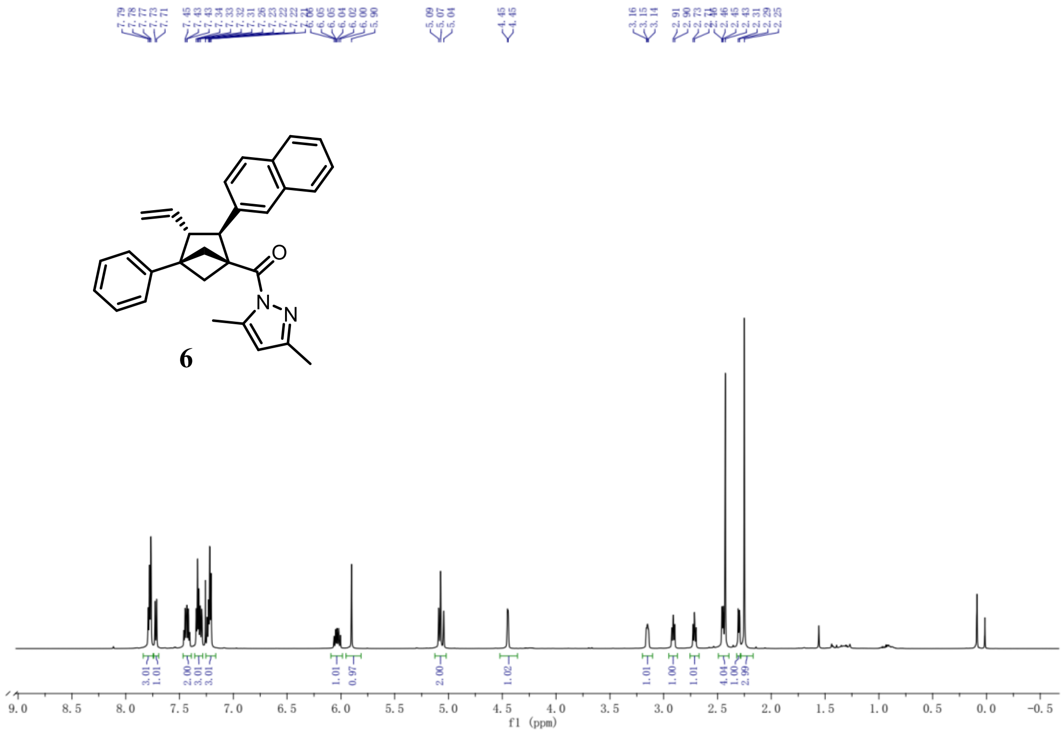

^{13}C NMR of **6** in $CDCl_3$ (151 MHz, $CDCl_3$)

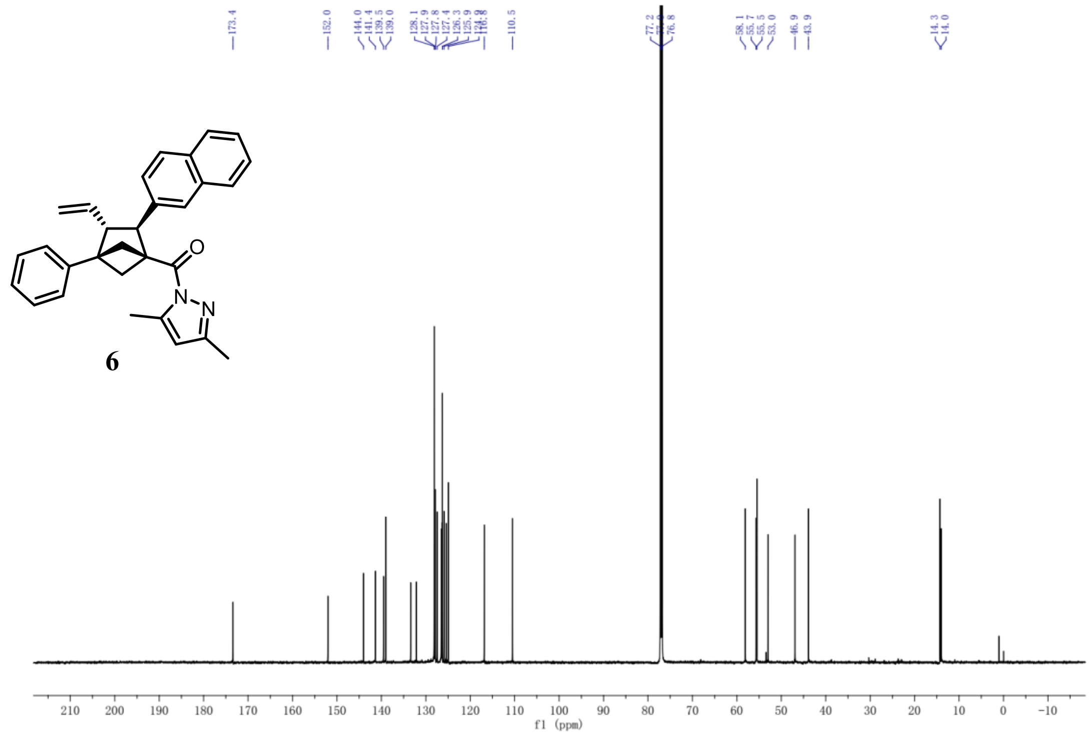

^{1}H NMR of **7** in $CDCl_3$ (600 MHz, $CDCl_3$)

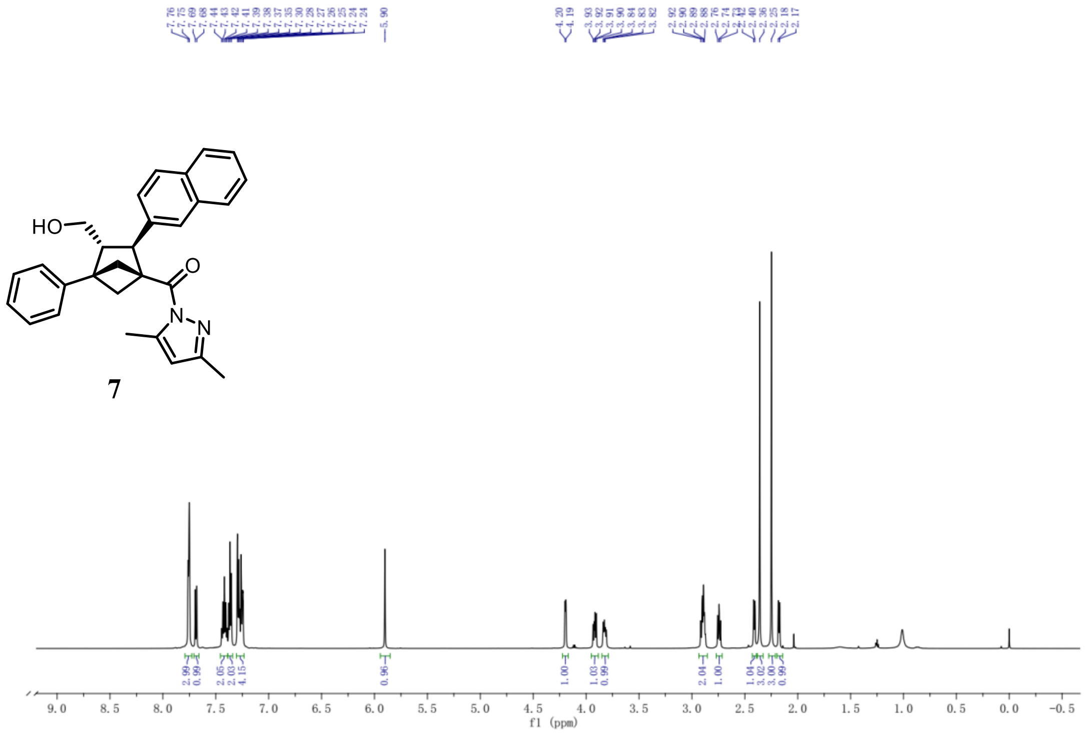

7

^{13}C NMR of **7** in $CDCl_3$ (151 MHz, $CDCl_3$)

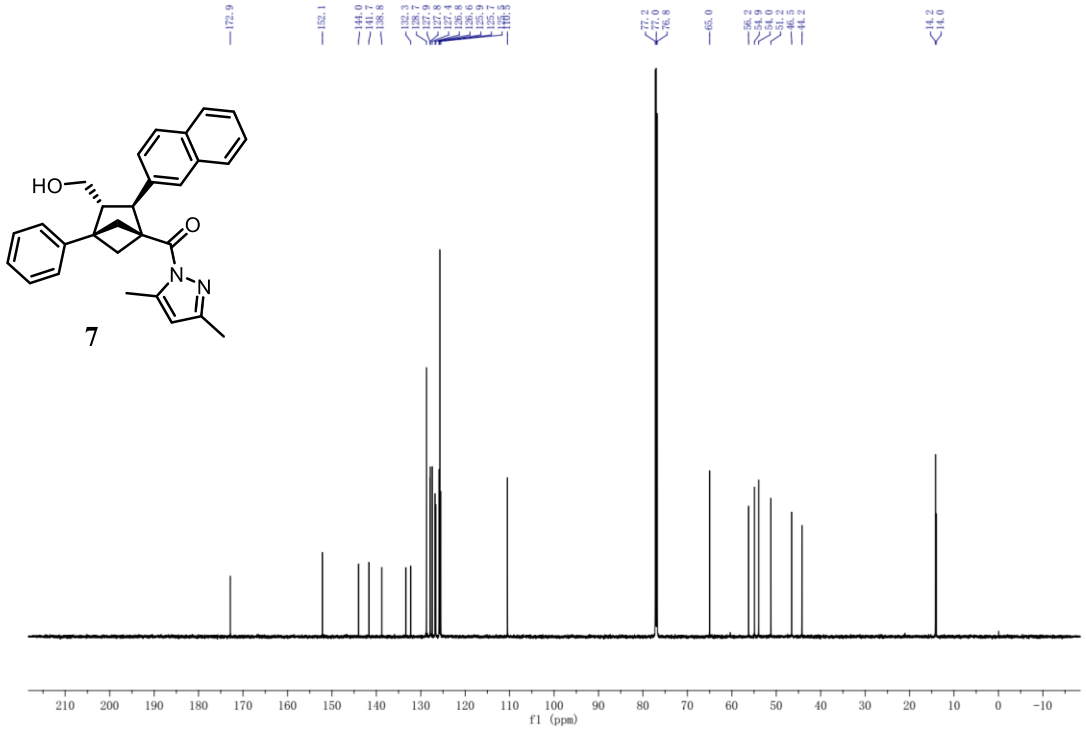

7

^{1}H NMR of **8** in $CDCl_3$ (600 MHz, $CDCl_3$)

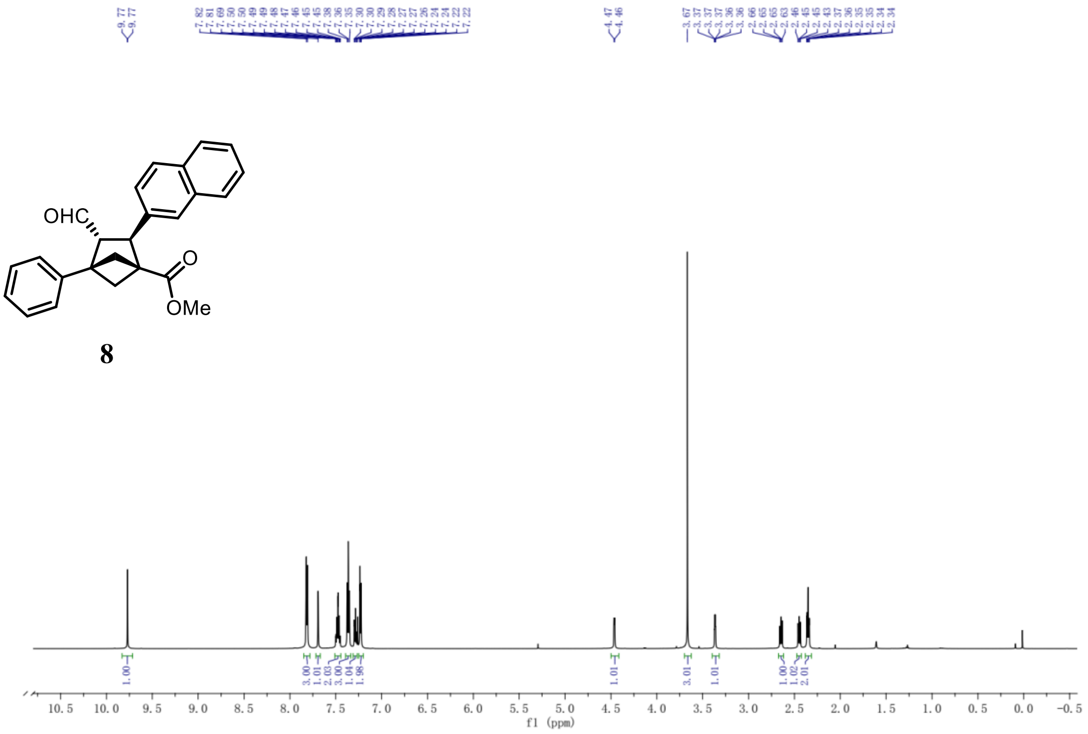

^{13}C NMR of **8** in $CDCl_3$ (151 MHz, $CDCl_3$)

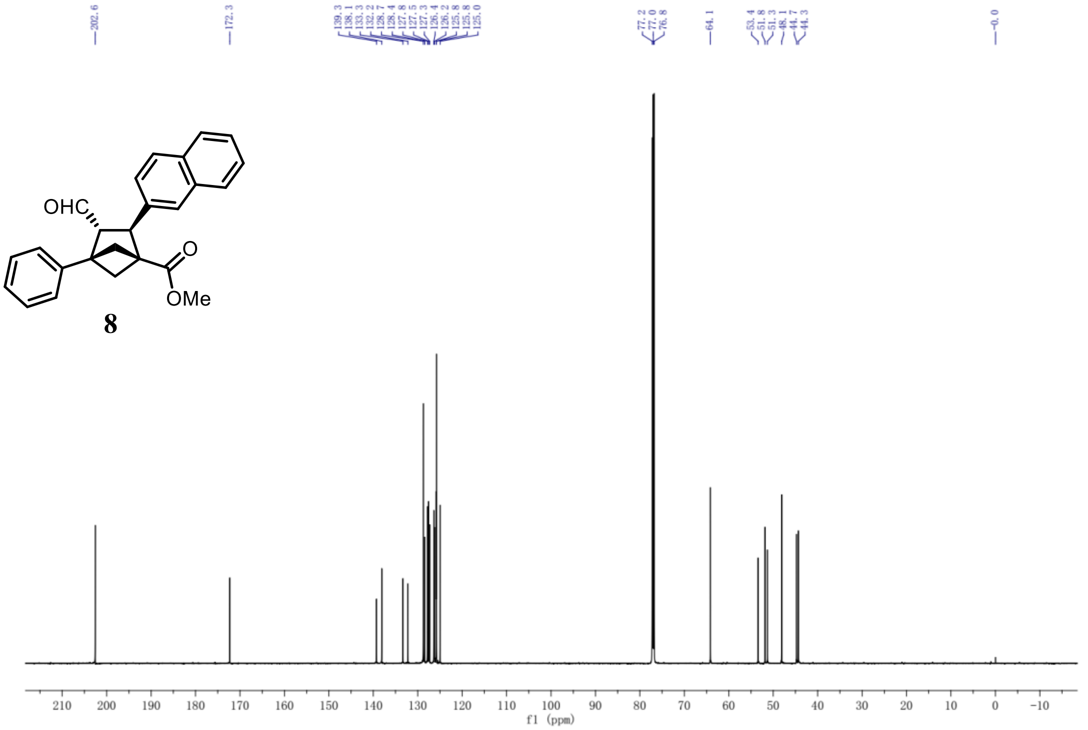

^{1}H NMR of **9** in $CDCl_3$ (600 MHz, $CDCl_3$)

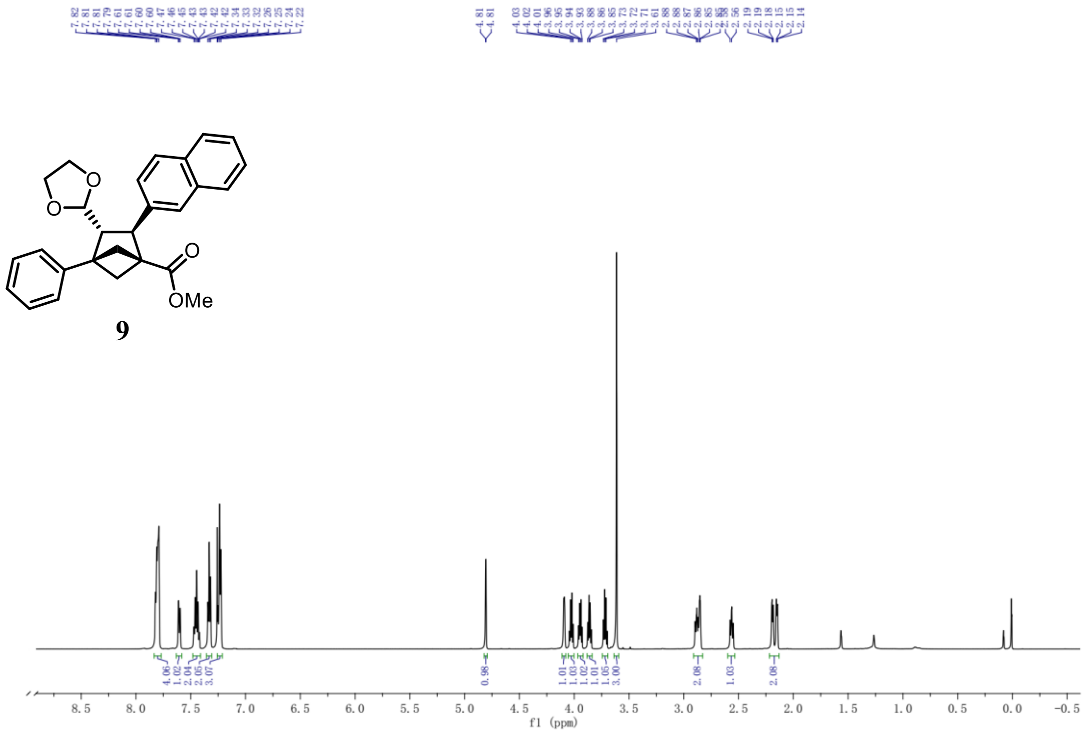

^{13}C NMR of **9** in $CDCl_3$ (151 MHz, $CDCl_3$)

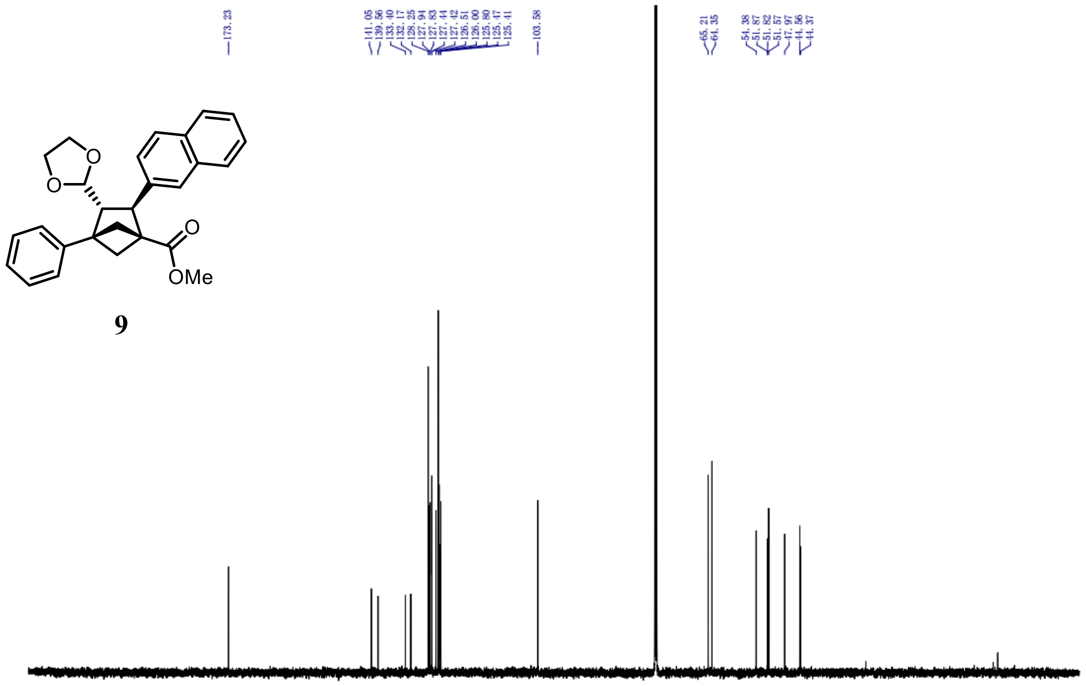

^{1}H NMR of **10** in $CDCl_3$ (600 MHz, $CDCl_3$)

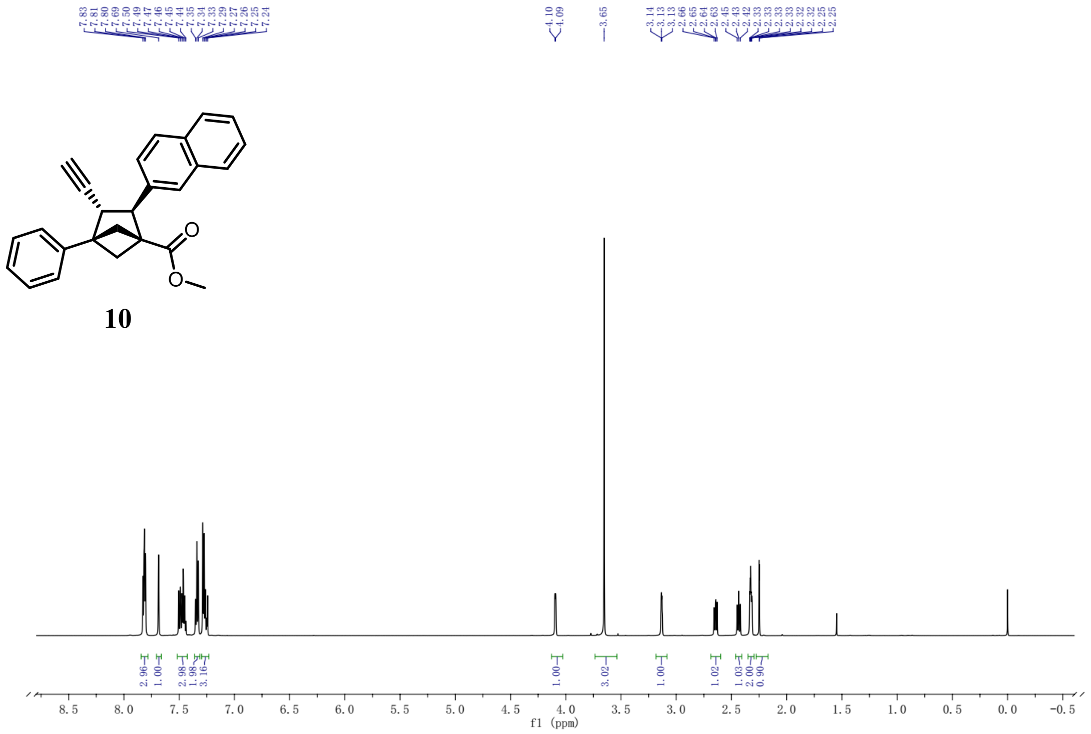

^{13}C NMR of **10** in $CDCl_3$ (151 MHz, $CDCl_3$)

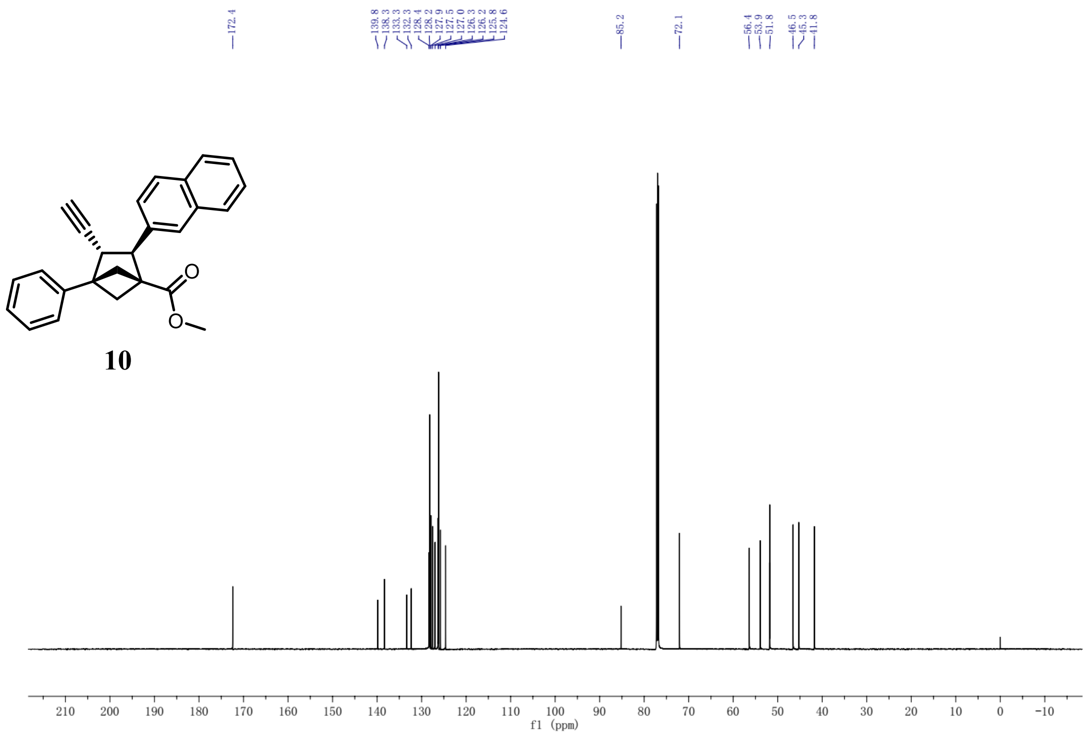

^{1}H NMR of **11** in $CDCl_3$ (600 MHz, $CDCl_3$)

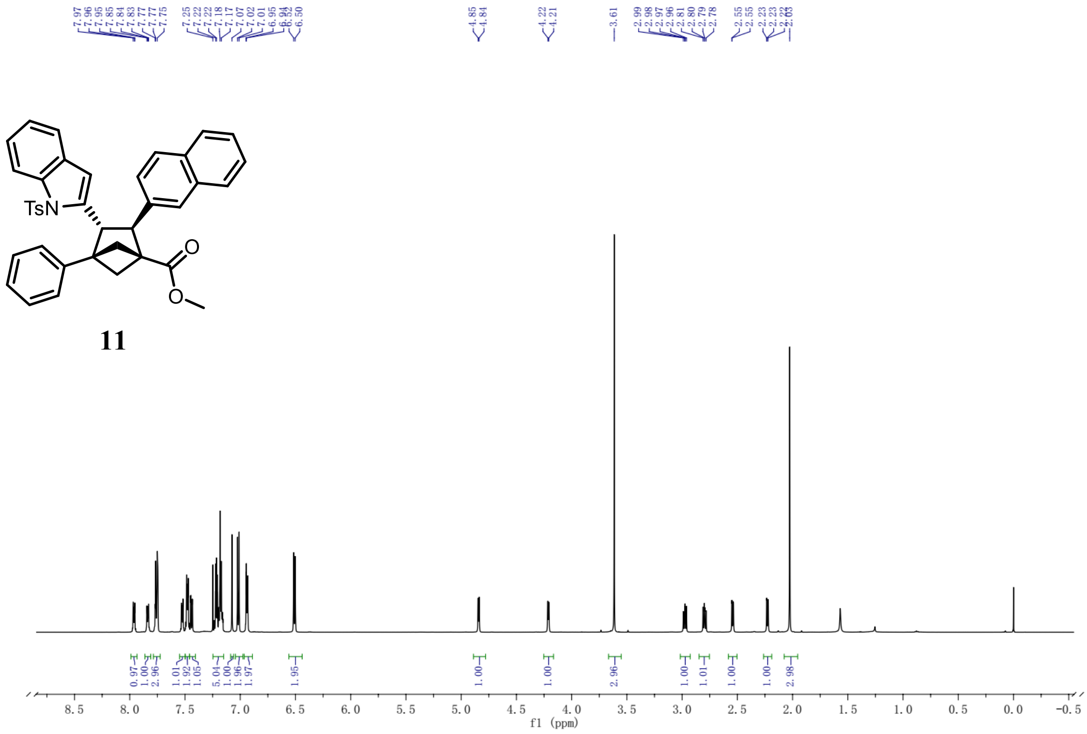

^{13}C NMR of **11** in $CDCl_3$ (151 MHz, $CDCl_3$)

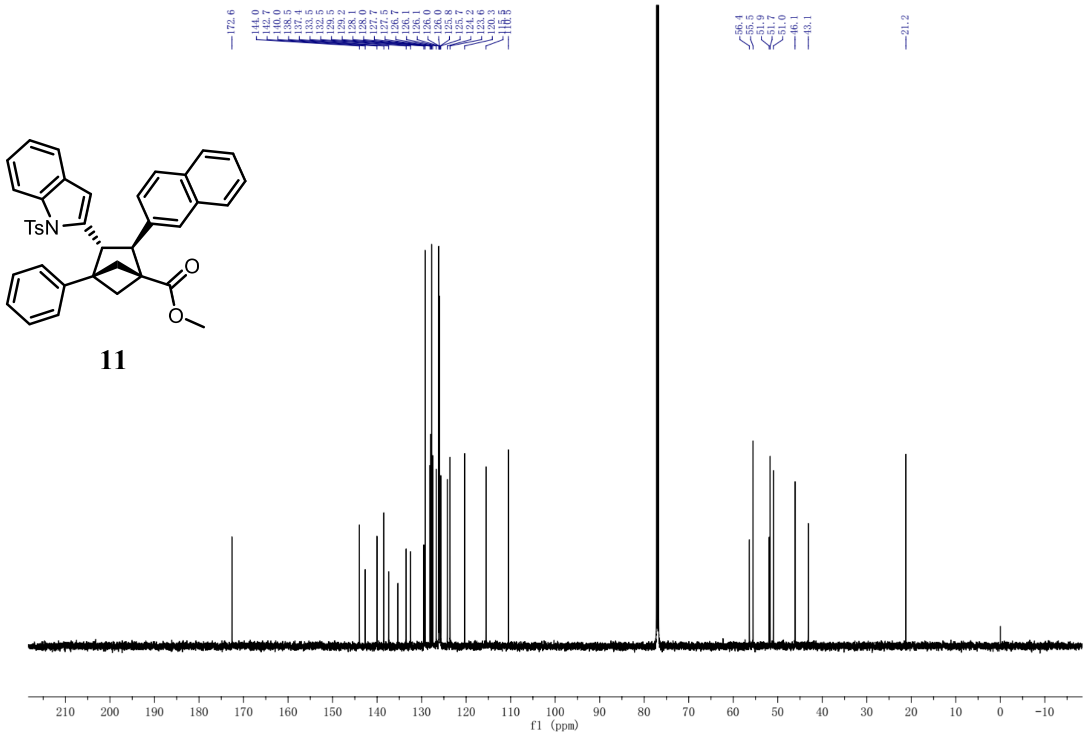

^{1}H NMR of **12** in $CDCl_3$ (600 MHz, $CDCl_3$)

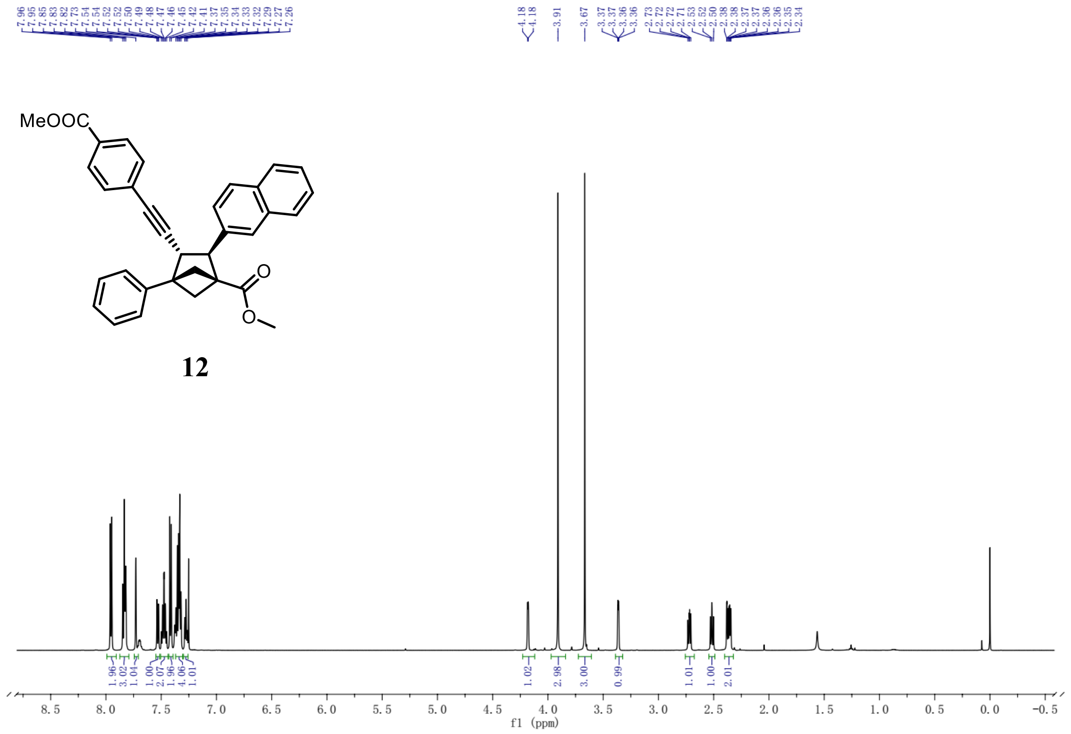

^{13}C NMR of **12** in $CDCl_3$ (151 MHz, $CDCl_3$)

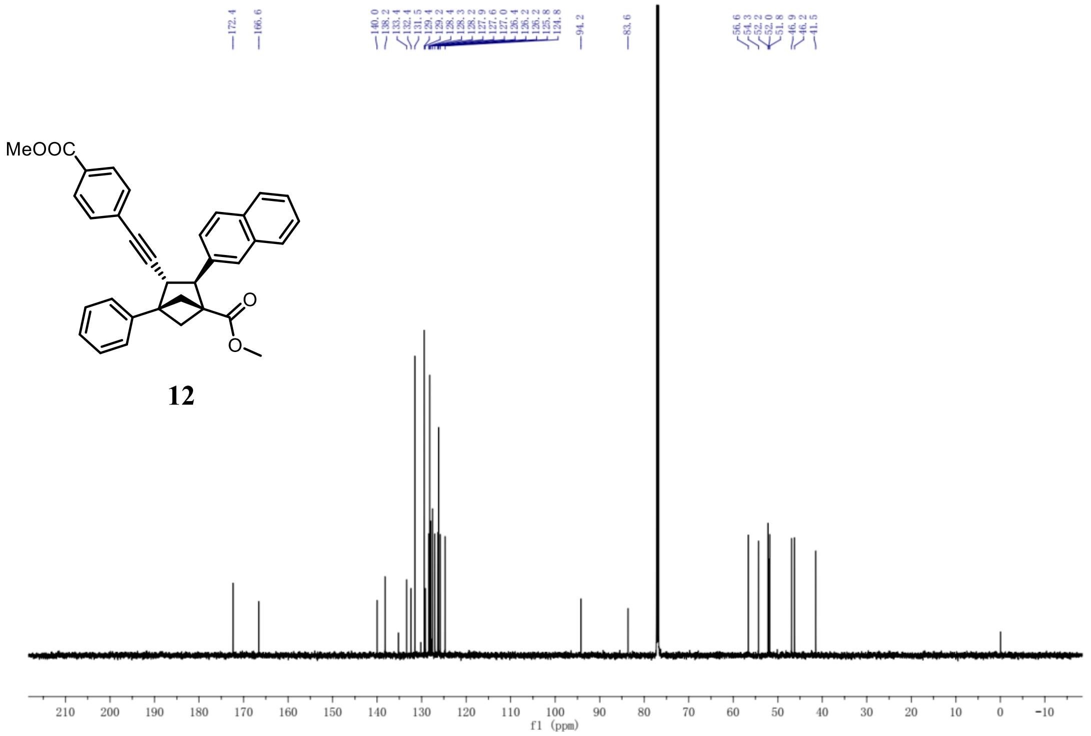

^{1}H NMR of **13** in $CDCl_3$ (600 MHz, $CDCl_3$)

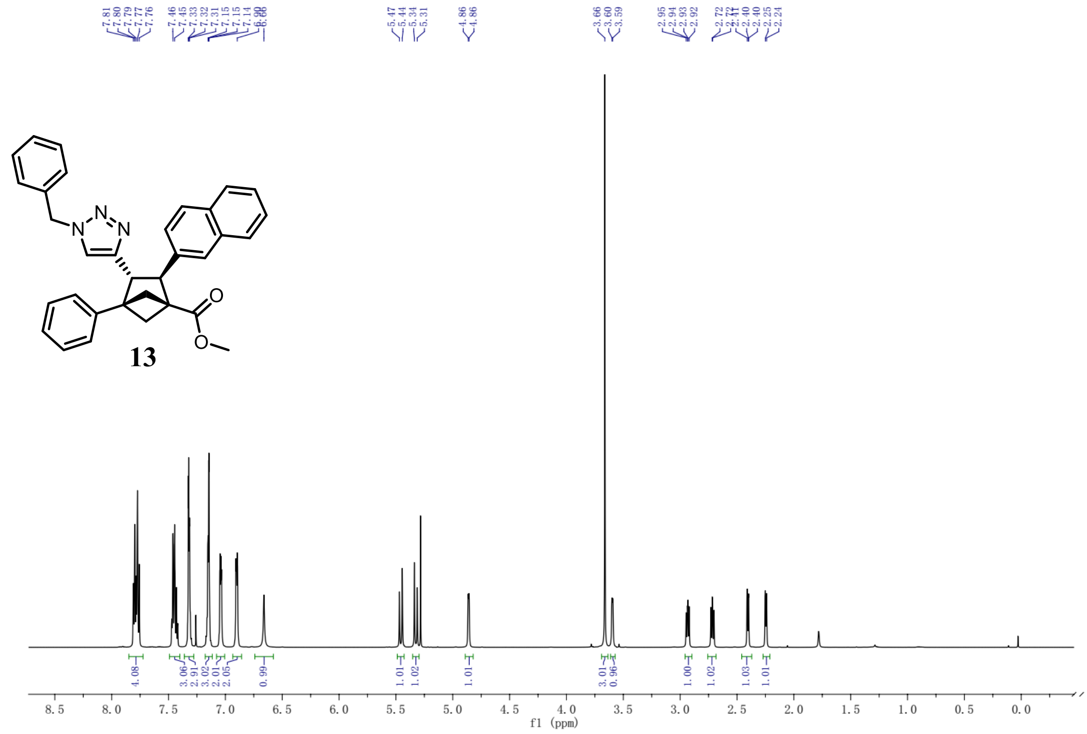

^{13}C NMR of **13** in $CDCl_3$ (151 MHz, $CDCl_3$)

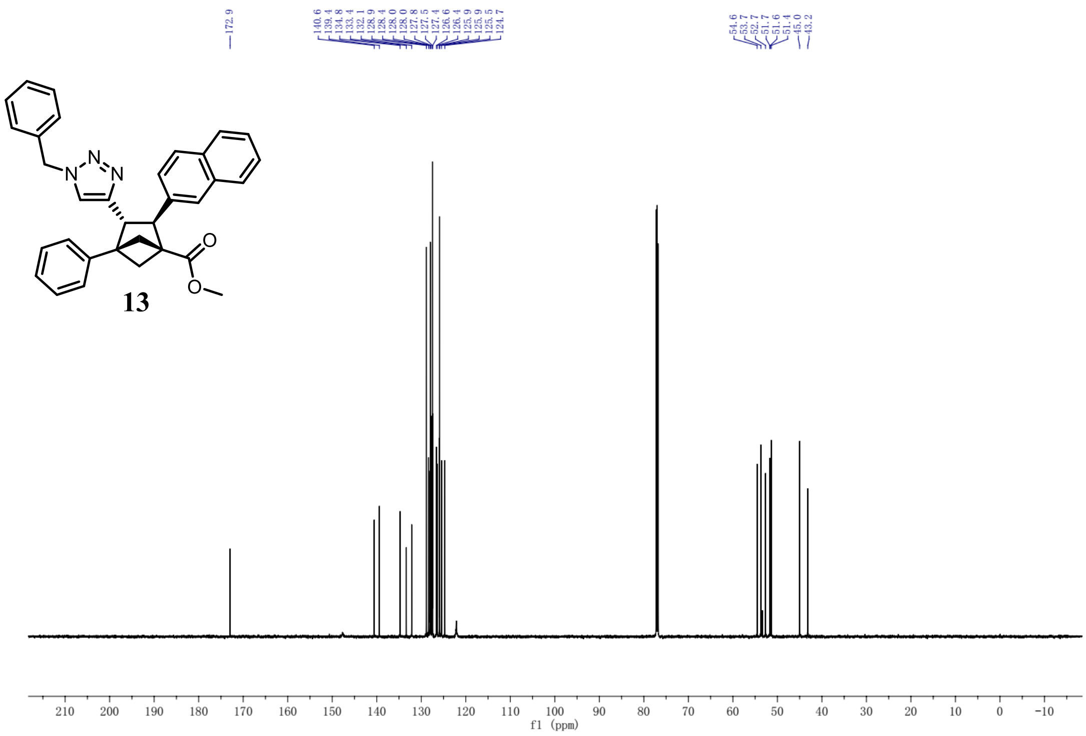

^{1}H NMR of **15** in CDCl3 (600 MHz, CDCl3)

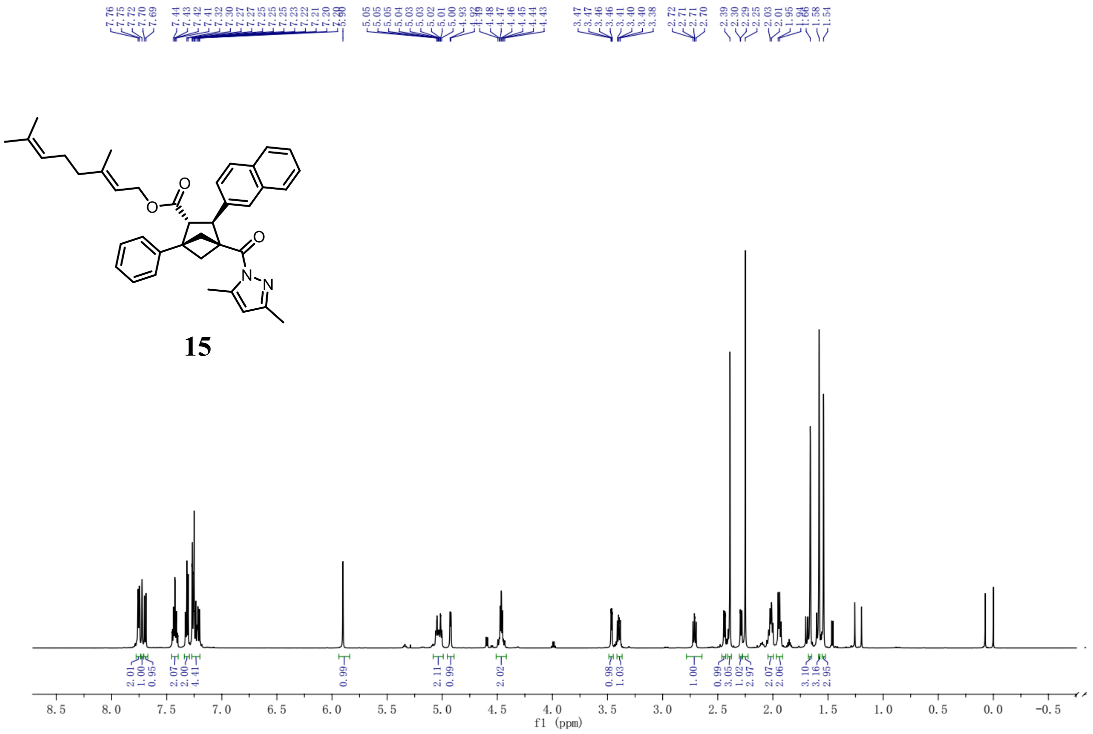

15

^{13}C NMR of **15** in CDCl3 (151 MHz, CDCl3)

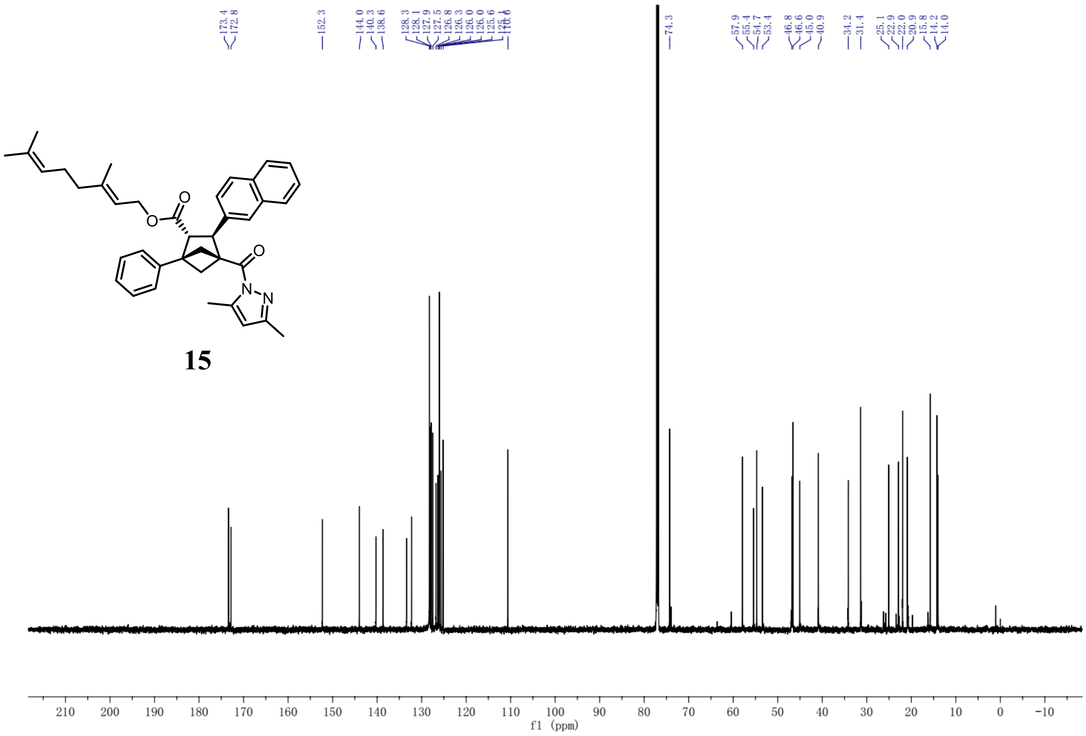

15

^{1}H NMR of **16** in $CDCl_3$ (600 MHz, $CDCl_3$)

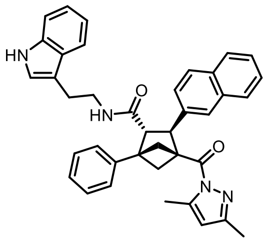

16

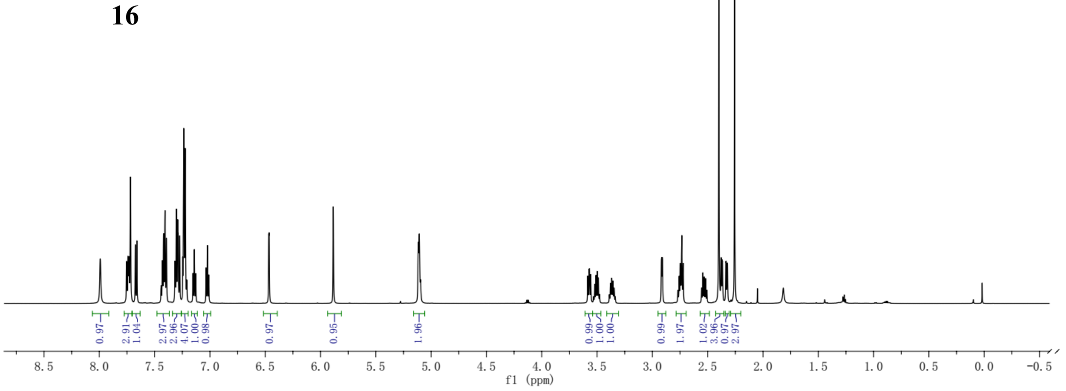

^{13}C NMR of **16** in $CDCl_3$ (151 MHz, $CDCl_3$)

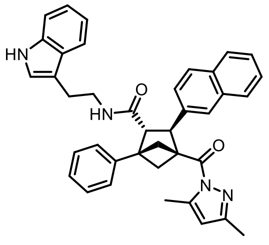

16

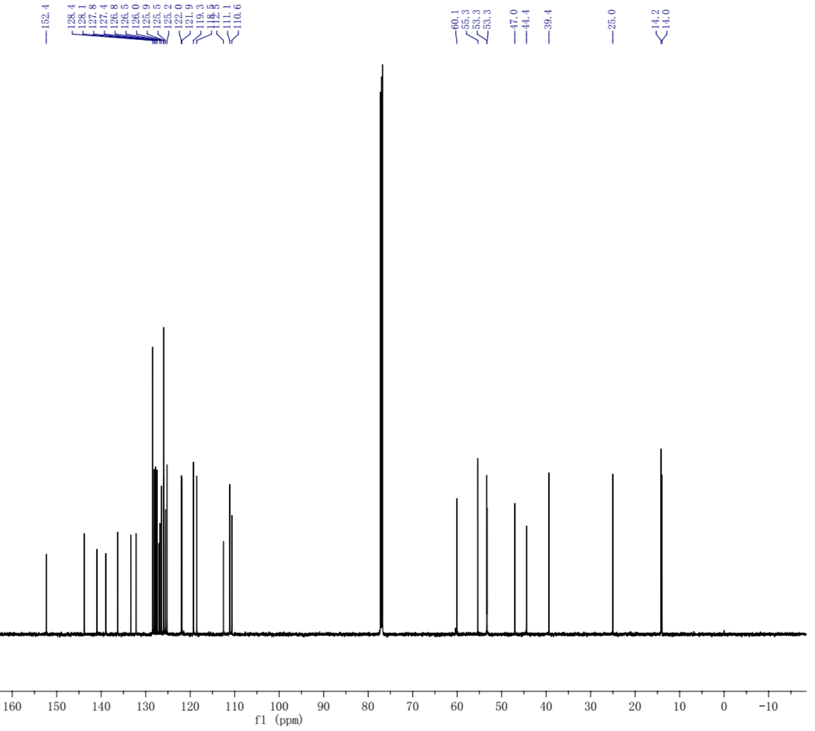

^{1}H NMR of **17** in CDCl3 (600 MHz, CDCl3)

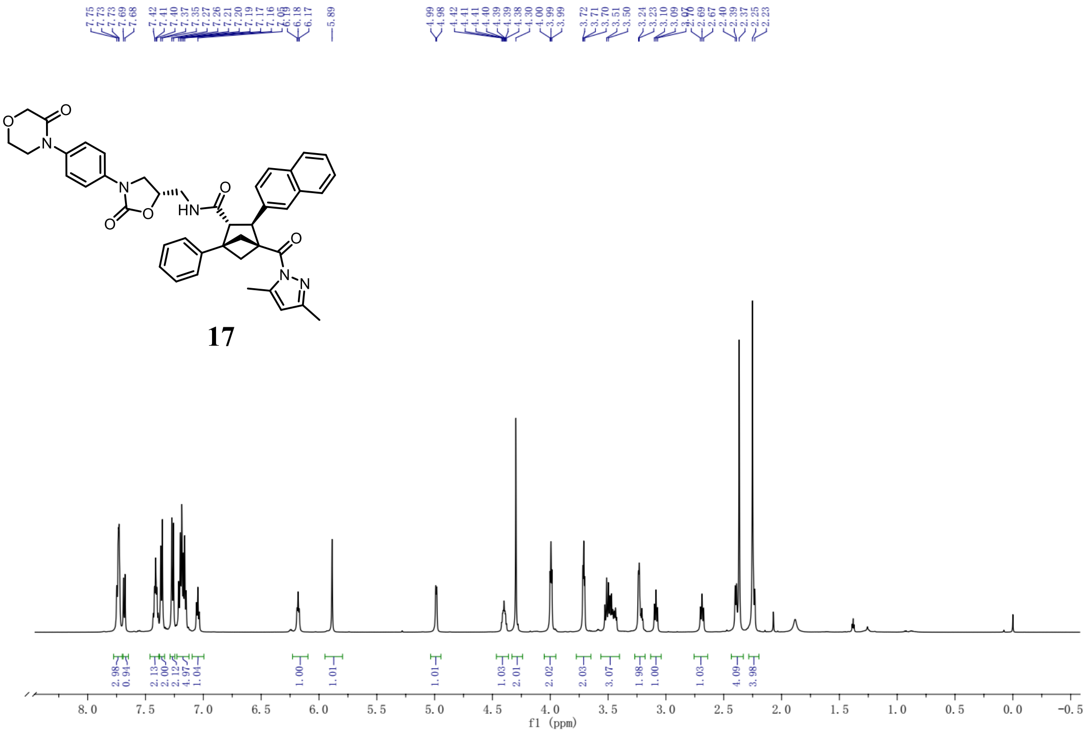

^{13}C NMR of **17** in CDCl3 (151 MHz, CDCl3)

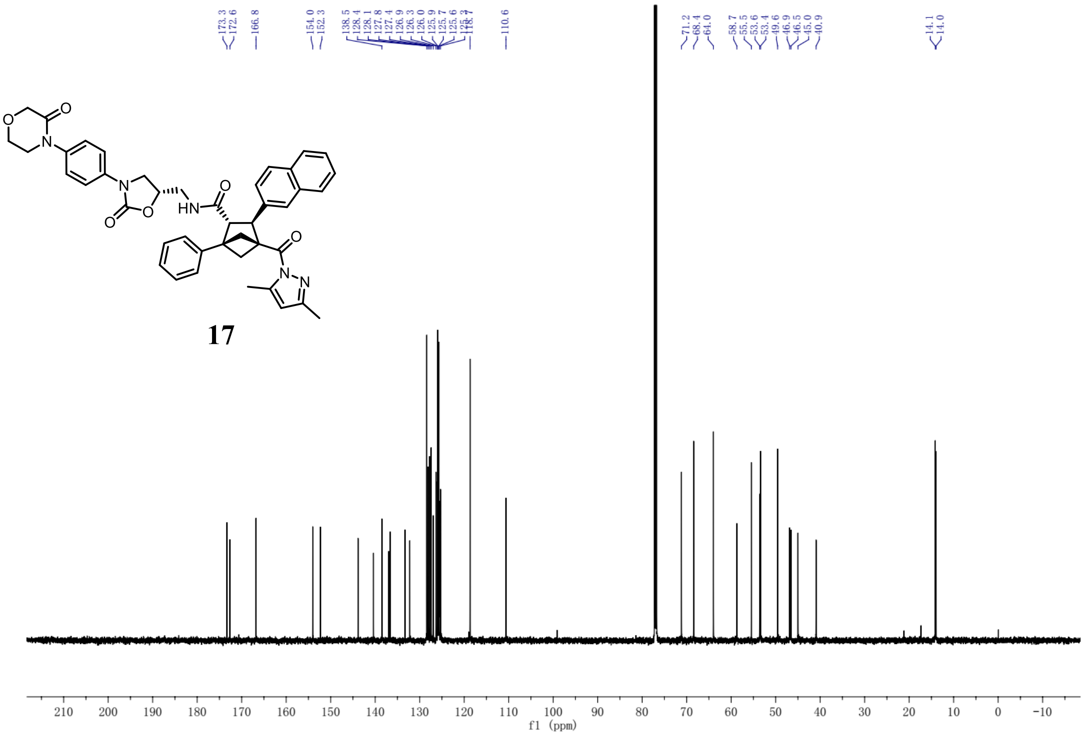

^{1}H NMR of **18** in $CDCl_3$ (600 MHz, $CDCl_3$)

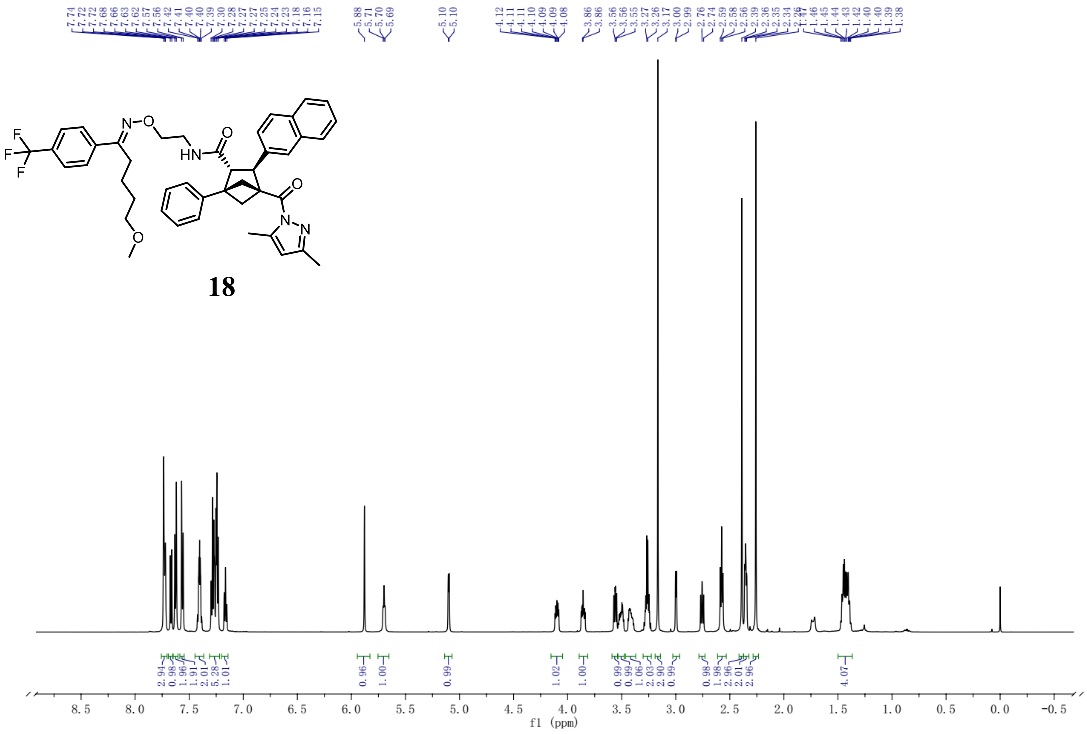

^{13}C NMR of **18** in $CDCl_3$ (151 MHz, $CDCl_3$)

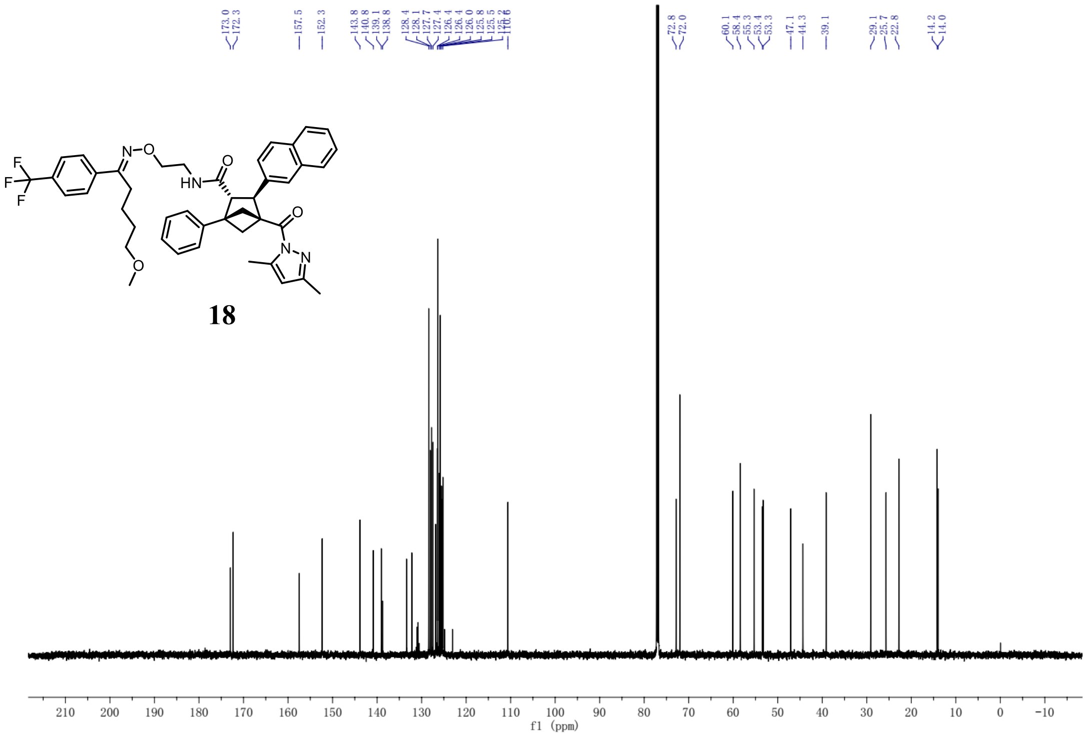

^{19}F NMR of **18** in $CDCl_3$ (565 MHz, $CDCl_3$)

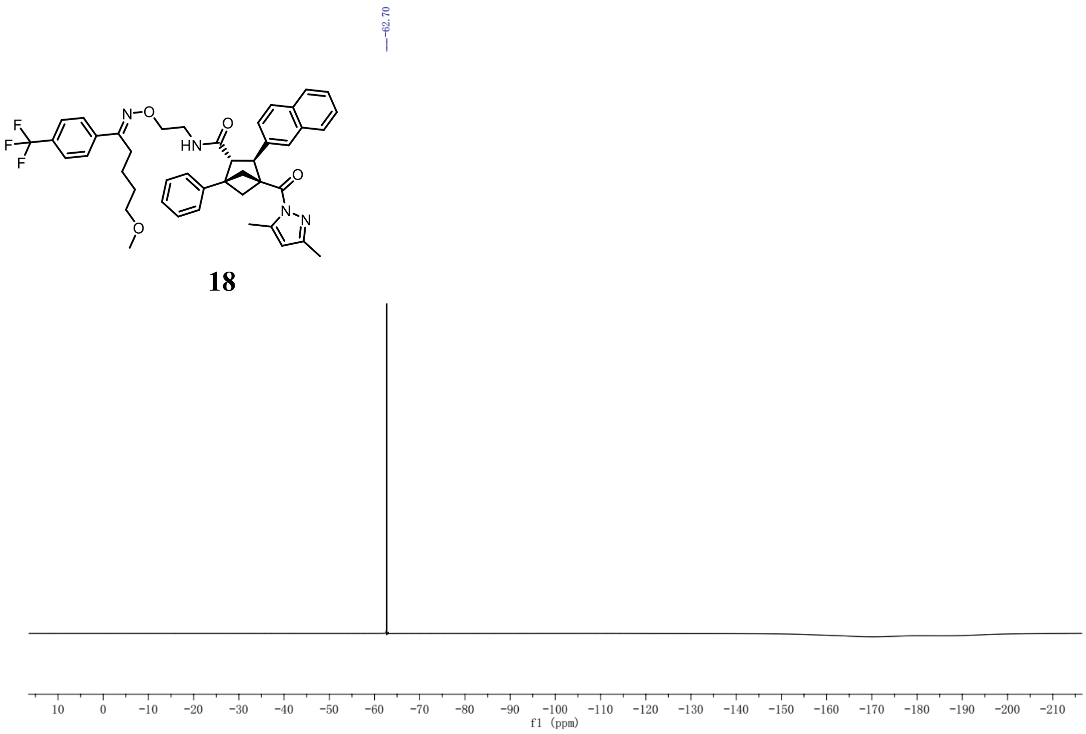

18

^{1}H NMR of **19** in $CDCl_3$ (600 MHz, $CDCl_3$)

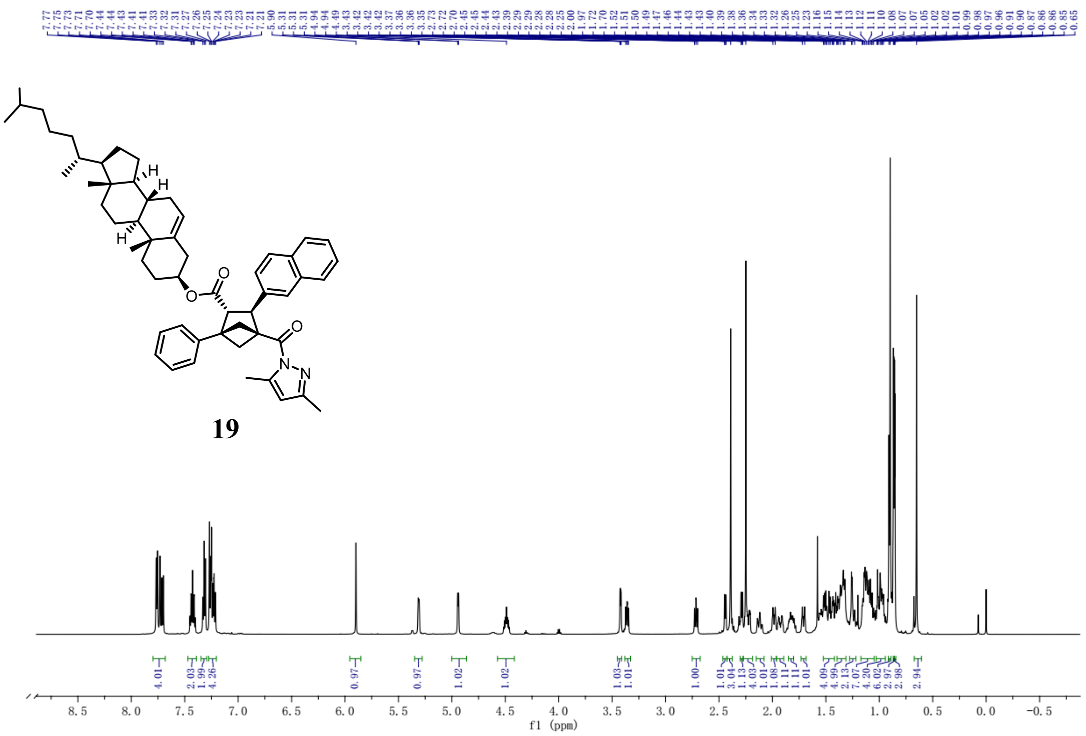

19

^{13}C NMR of **19** in CDCl$_3$ (151 MHz, CDCl$_3$)

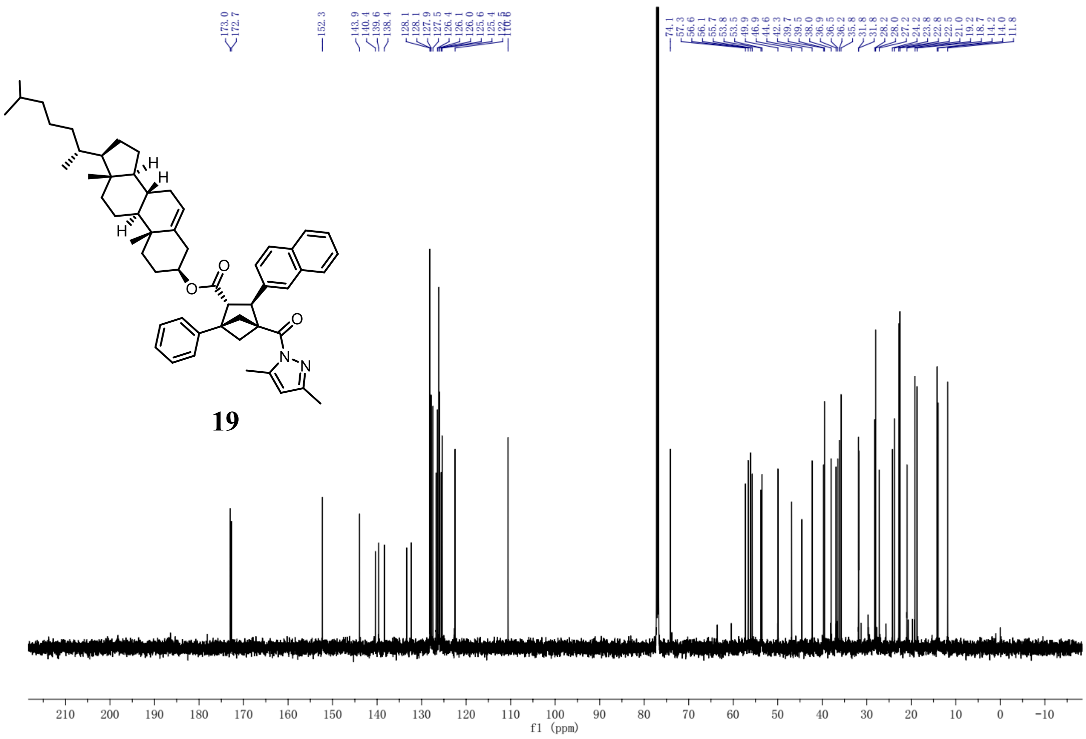

19

^{1}H NMR of **20** in CDCl$_3$ (600 MHz, CDCl$_3$)

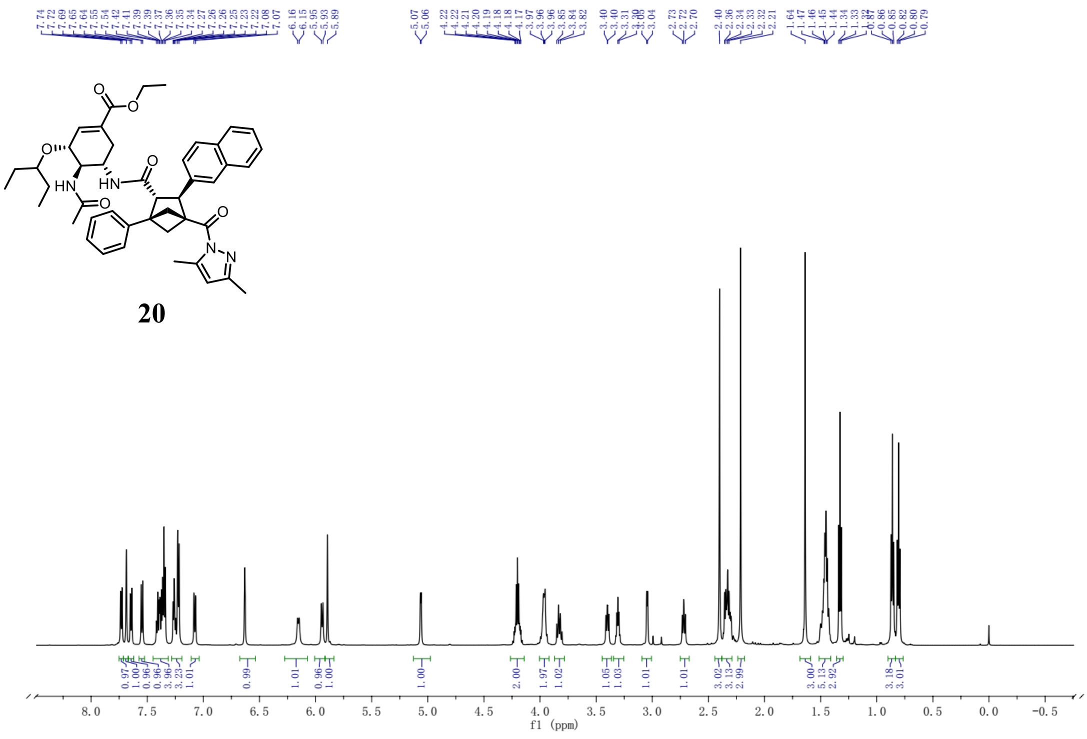

20

[13] C NMR of **20** in $CDCl_3$ (151 MHz, $CDCl_3$)

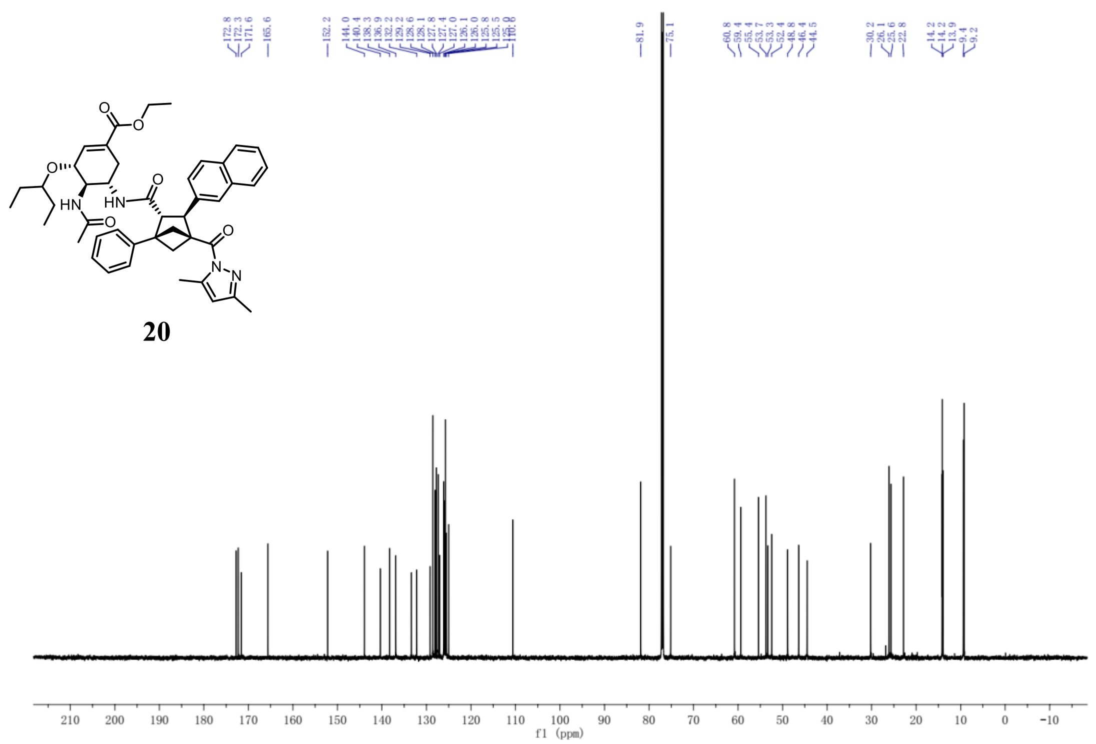

[1] H NMR of **21** in $CDCl_3$ (600 MHz, $CDCl_3$)

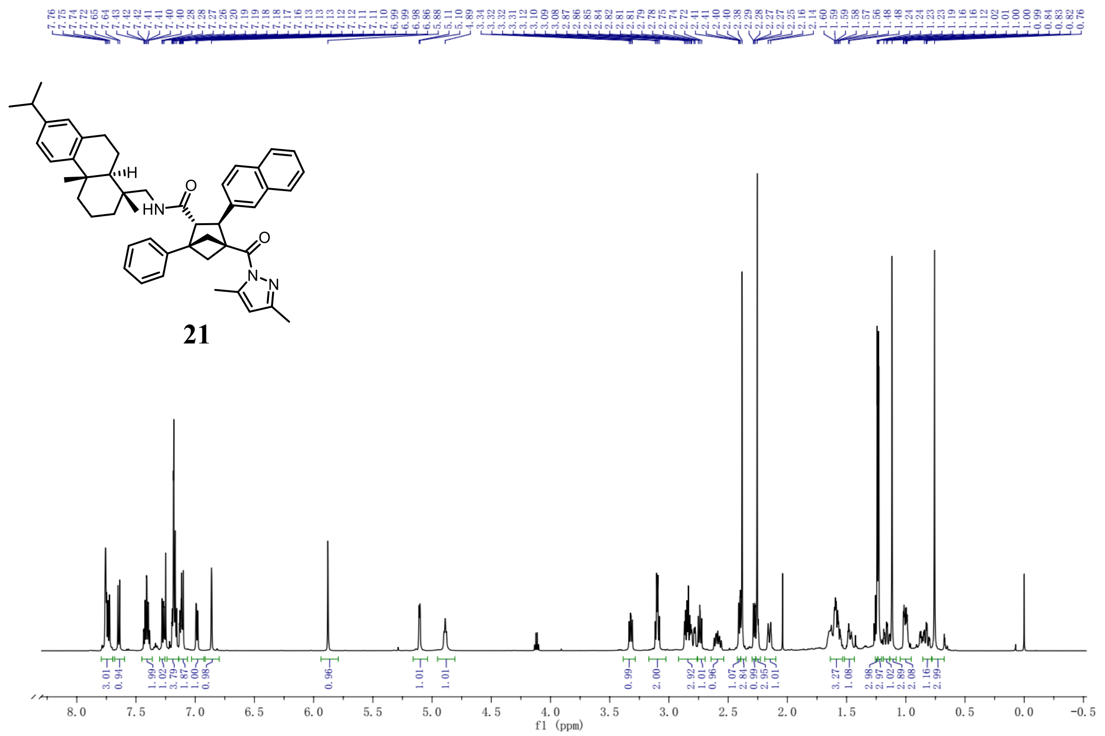

^{13}C NMR of **21** in $CDCl_3$ (151 MHz, $CDCl_3$)

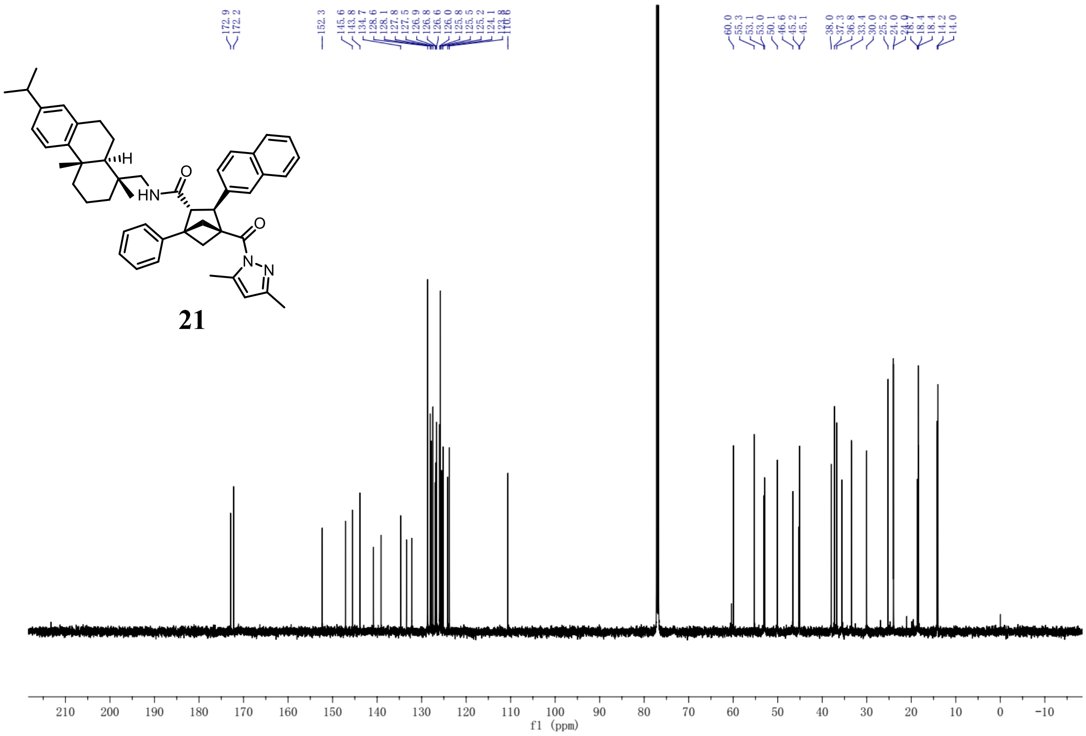

21

^{1}H NMR of **22** in $CDCl_3$ (600 MHz, $CDCl_3$)

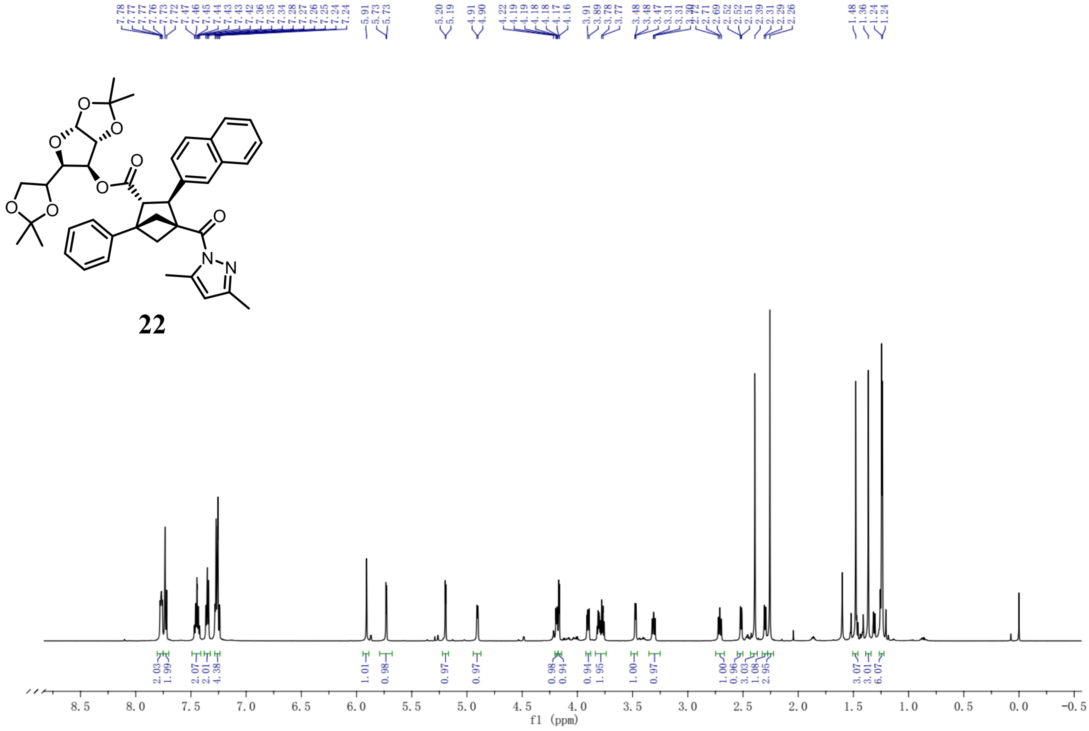

22

^{13}C NMR of **22** in $CDCl_3$ (151 MHz, $CDCl_3$)

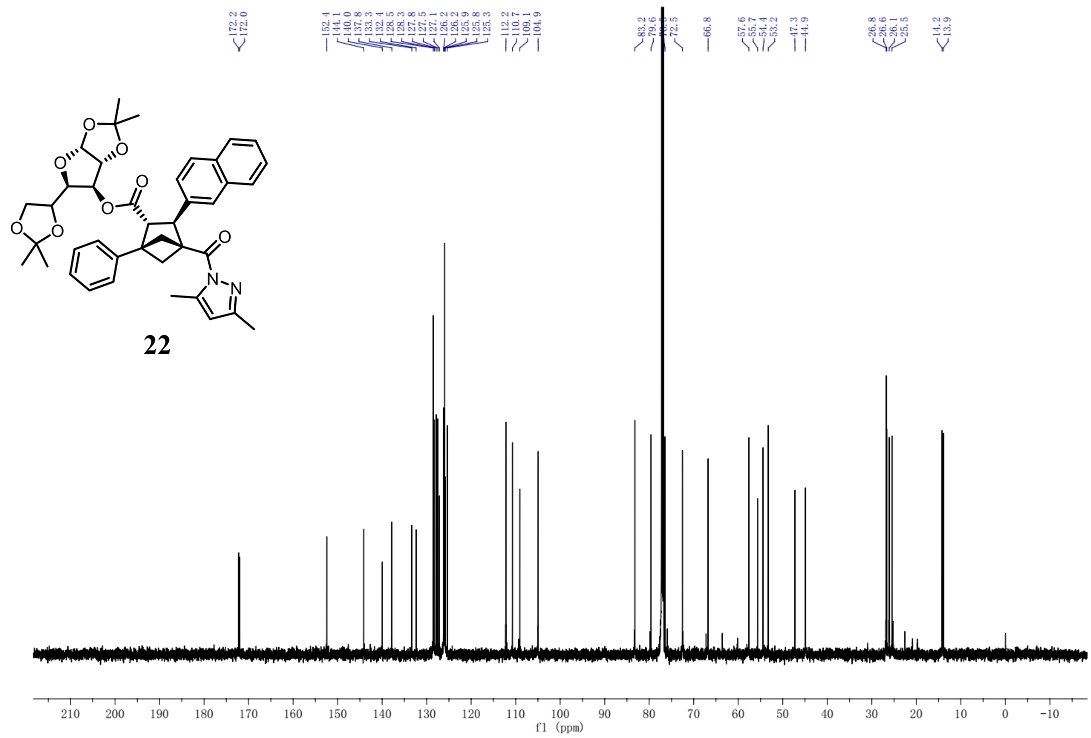

^{1}H NMR of **23** in $CDCl_3$ (600 MHz, $CDCl_3$)

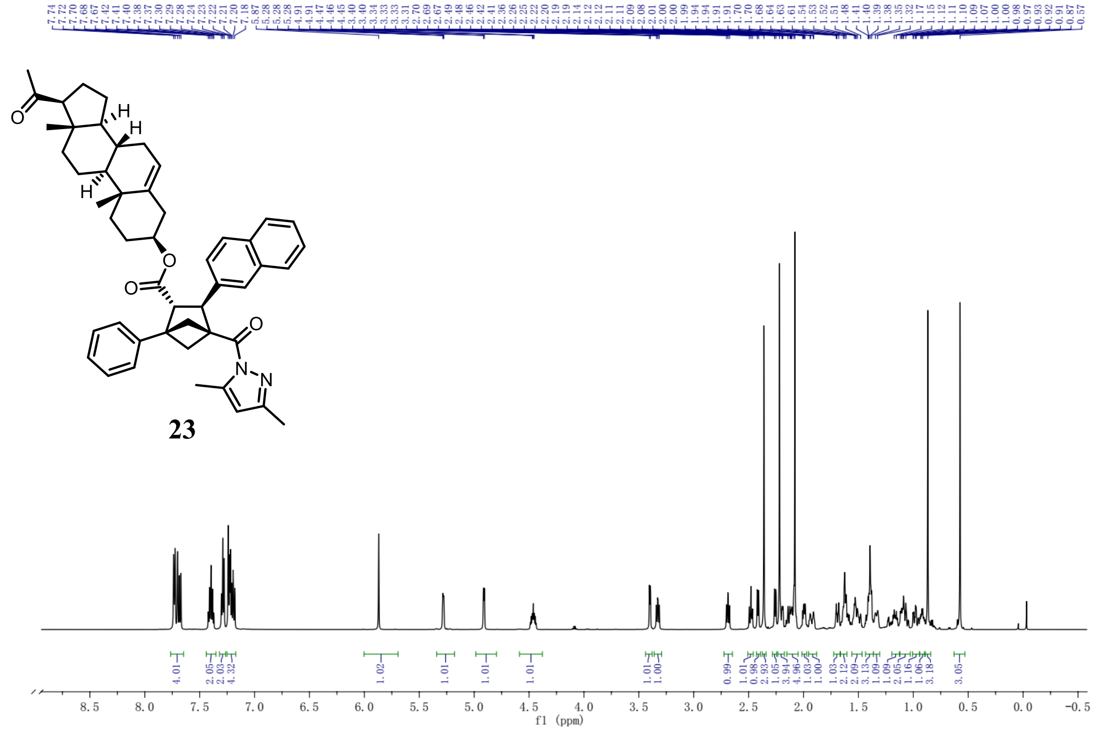

^{13}C NMR of **23** in $CDCl_3$ (151 MHz, $CDCl_3$)

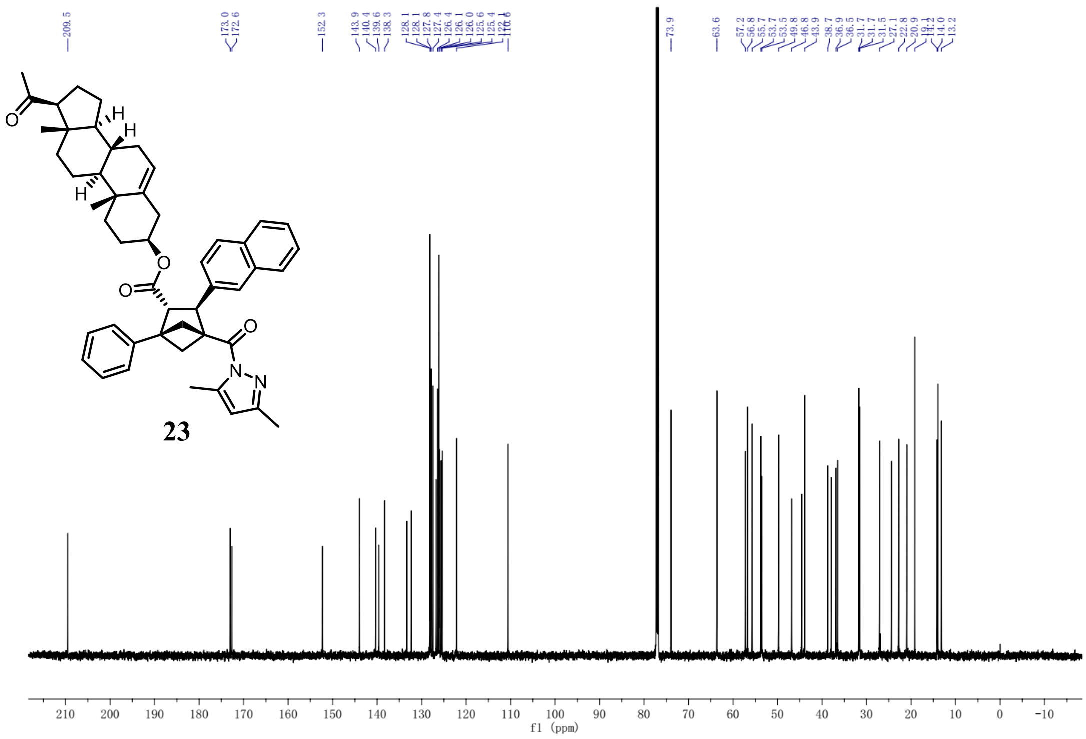

23

^{1}H NMR of **24** in $CDCl_3$ (600 MHz, $CDCl_3$)

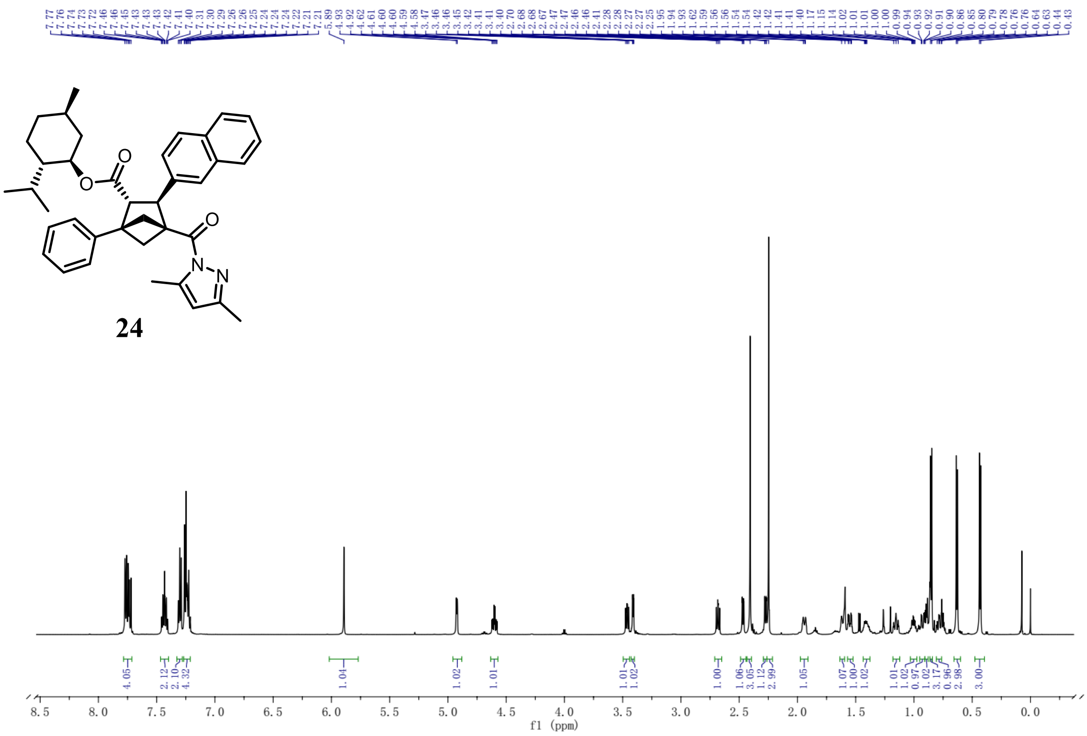

24

^{13}C NMR of **24** in $CDCl_3$ (151 MHz, $CDCl_3$)

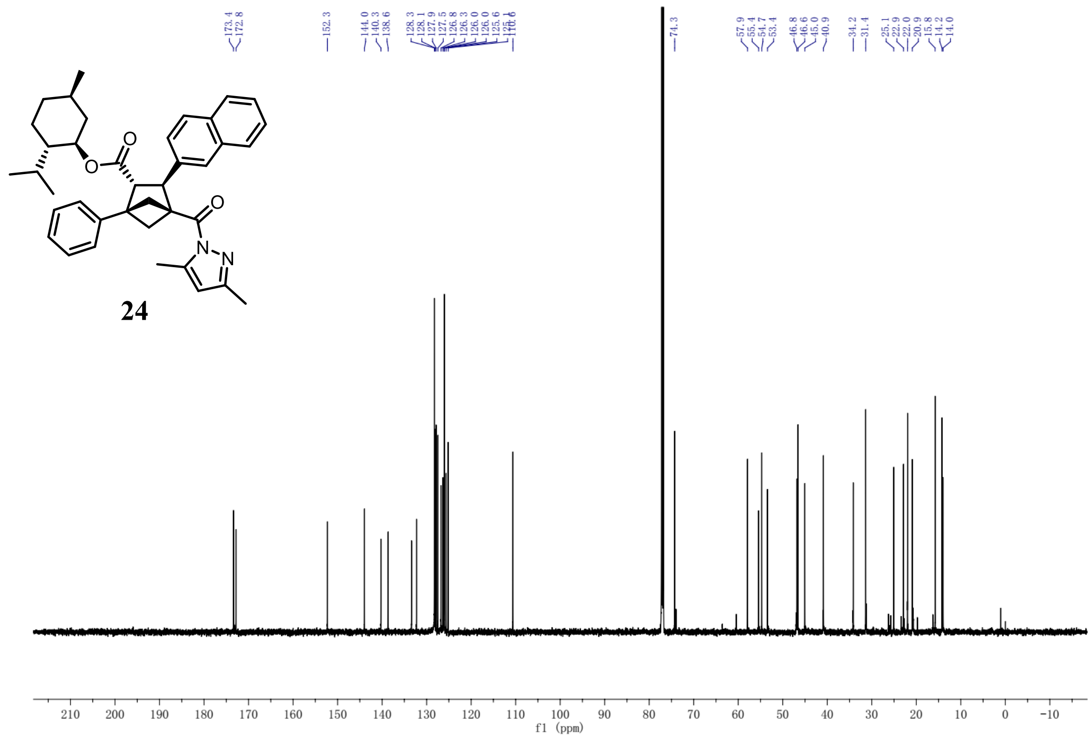

^{1}H NMR of **25** in $CDCl_3$ (600 MHz, $CDCl_3$)

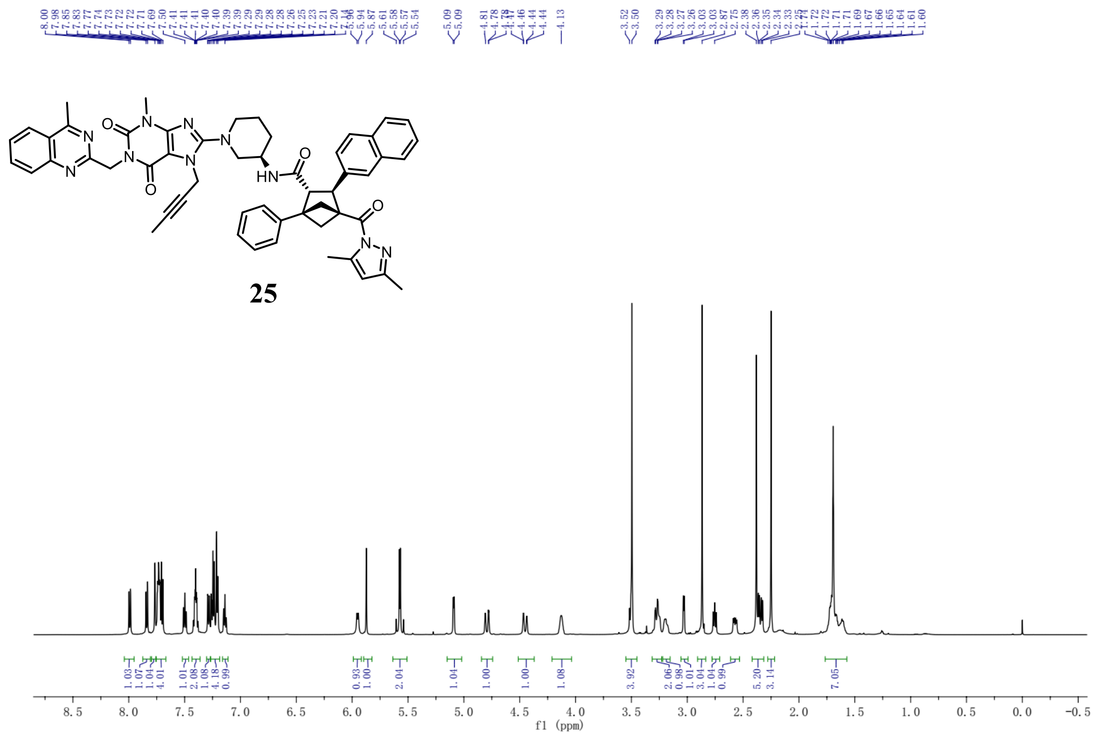

^{13}C NMR of **25** in $CDCl_3$ (151 MHz, $CDCl_3$)

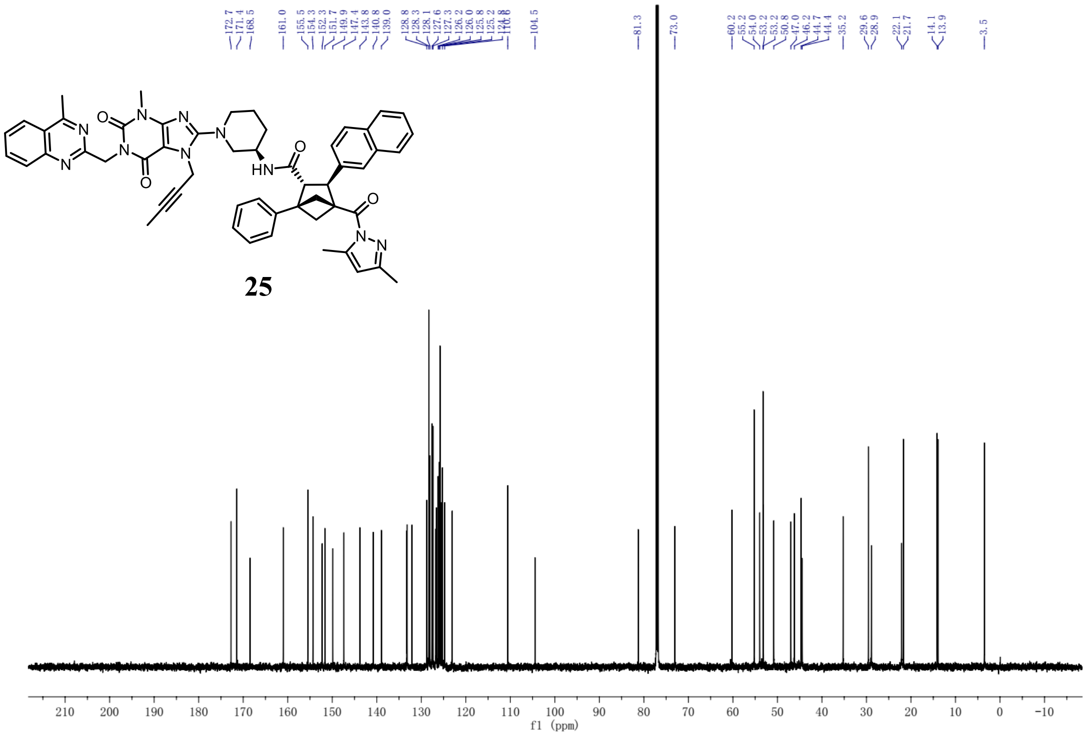

25

^{1}H NMR of **26** in $CDCl_3$ (600 MHz, $CDCl_3$)

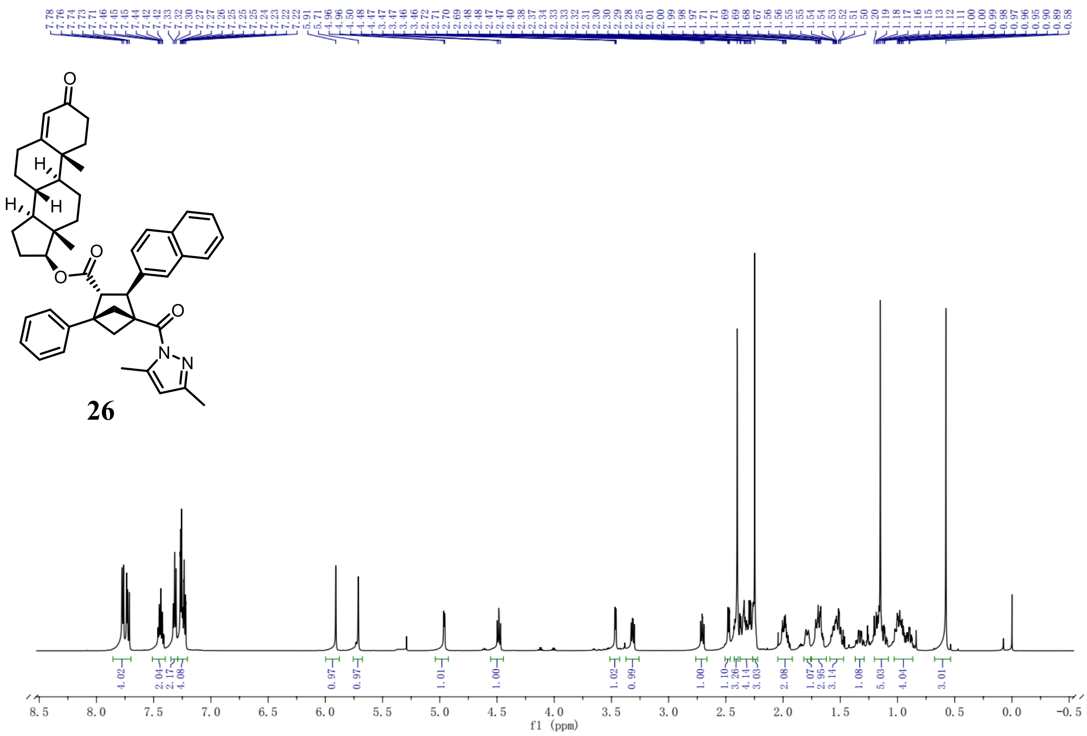

26

^{13}C NMR of **26** in $CDCl_3$ (151 MHz, $CDCl_3$)

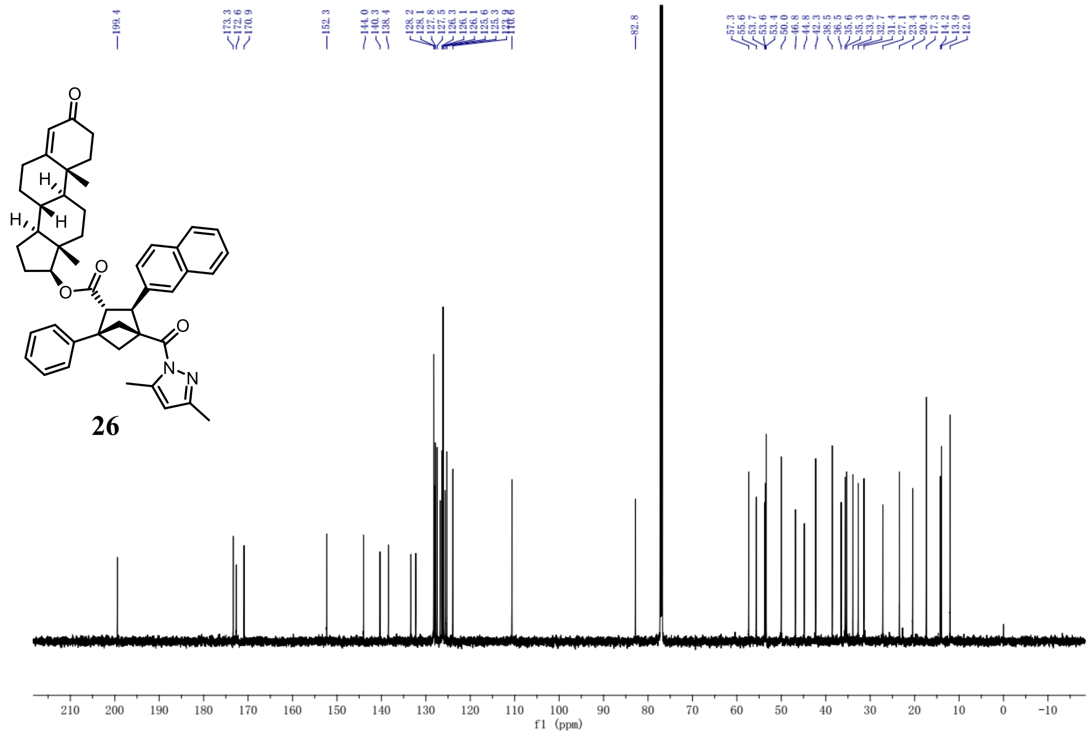

^{1}H NMR of 27 in $CDCl_3$ (600 MHz, $CDCl_3$)

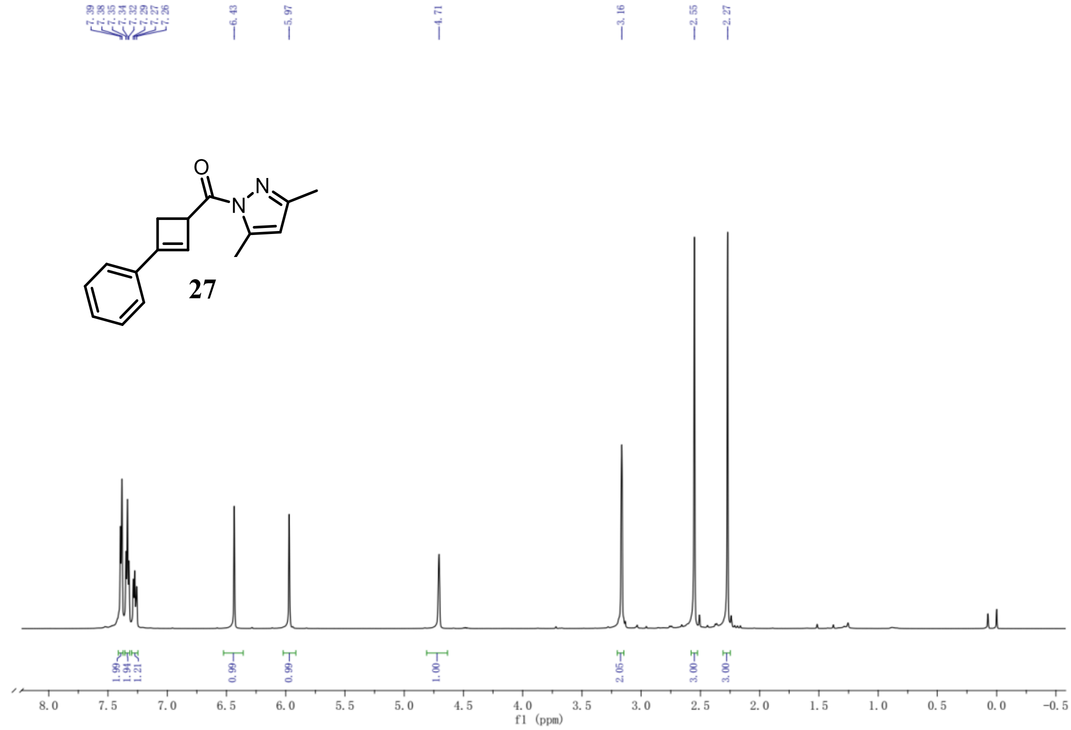

^{13}C NMR of **27** in $CDCl_3$ (151 MHz, $CDCl_3$)

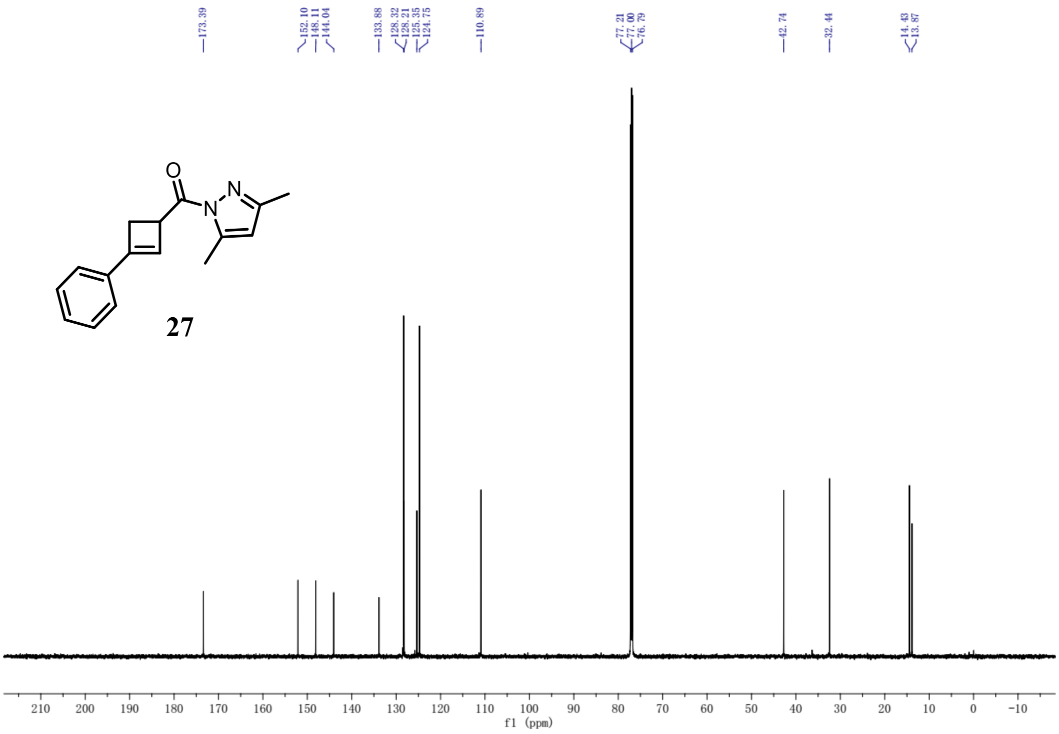

8. HPLC Spectra of Compound

Racemic **3a**

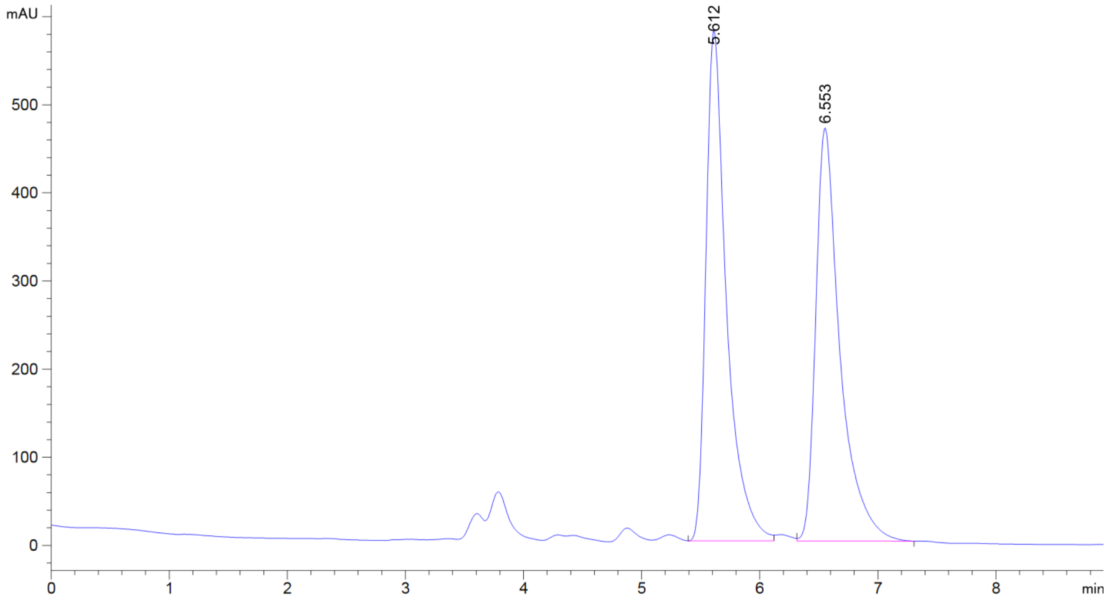

Peak #	RetTime [min]	Type	Width [min]	Area [mAU*s]	Height [mAU]	Area %
1	5.612	BV	0.1799	7041.62988	579.02667	51.2303
2	6.553	VB	0.2108	6703.40625	468.74686	48.7697

Enantioenriched **3a**

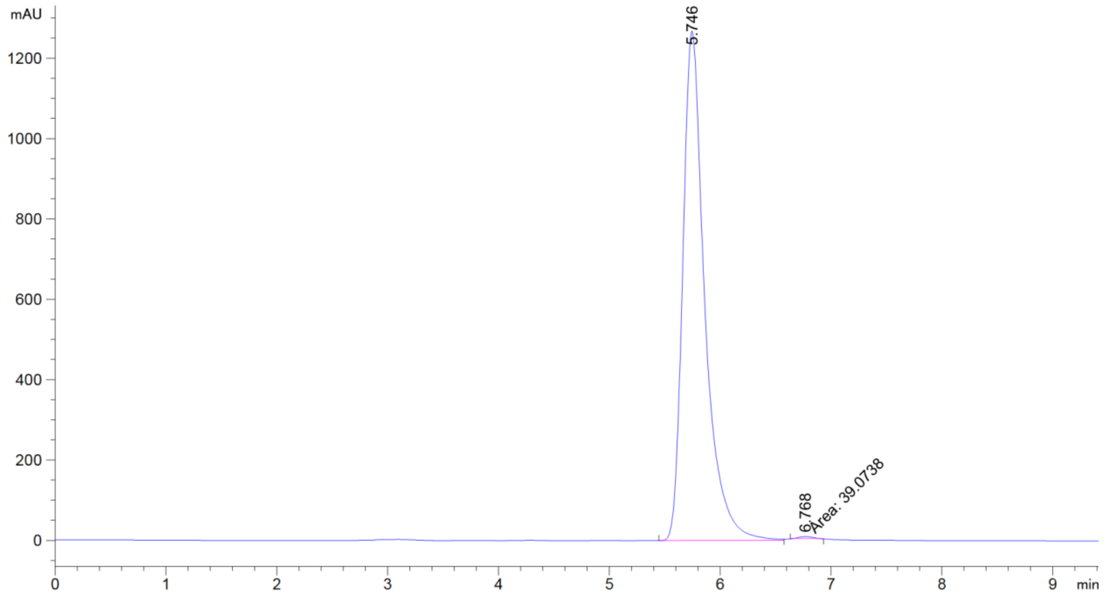

Peak #	RetTime [min]	Type	Width [min]	Area [mAU*s]	Height [mAU]	Area %
1	5.746	BV	0.2046	1.74552e4	1268.09705	99.7766
2	6.768	MM	0.1396	39.07383	4.66505	0.2234

Racemic **3b**

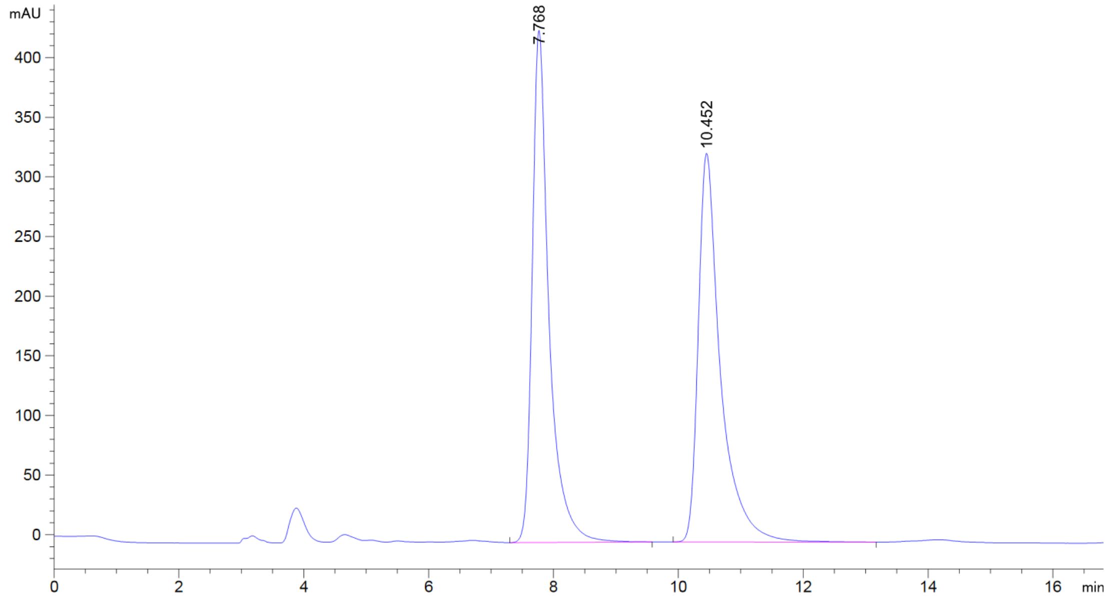

Peak #	RetTime [min]	Type	Width [min]	Area [mAU*s]	Height [mAU]	Area %
1	7.768	BB	0.2734	8002.43066	429.40131	49.7786
2	10.452	BB	0.3631	8073.61426	325.80118	50.2214

Enantioenriched **3b**

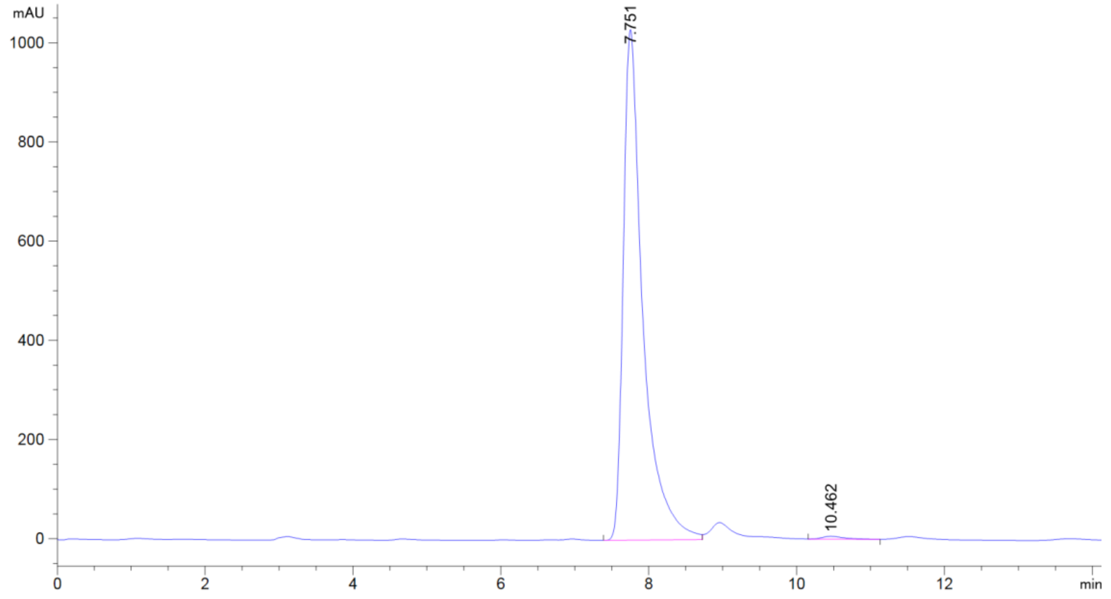

Peak #	RetTime [min]	Type	Width [min]	Area [mAU*s]	Height [mAU]	Area %
1	7.751	BV	0.2699	1.90551e4	1029.46106	99.3787
2	10.462	BB	0.3087	119.13261	5.68070	0.6213

Racemic **3c**

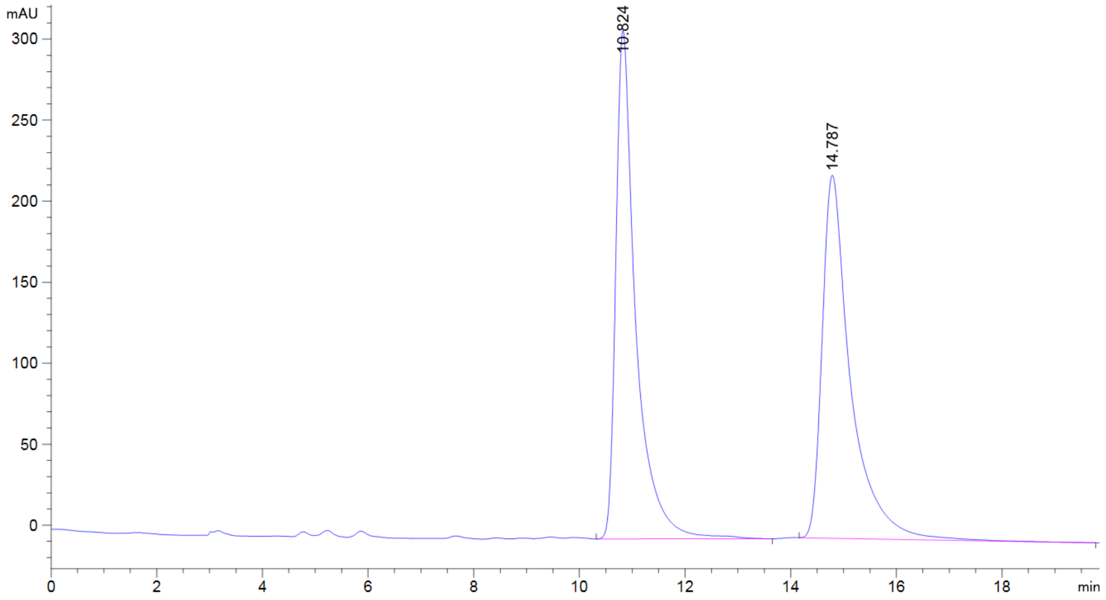

Peak #	RetTime [min]	Type	Width [min]	Area [mAU*s]	Height [mAU]	Area %
1	10.824	BB	0.3813	8266.77441	313.82730	49.5945
2	14.787	BBA	0.5436	8401.94238	224.02292	50.4055

Enantioenriched **3c**

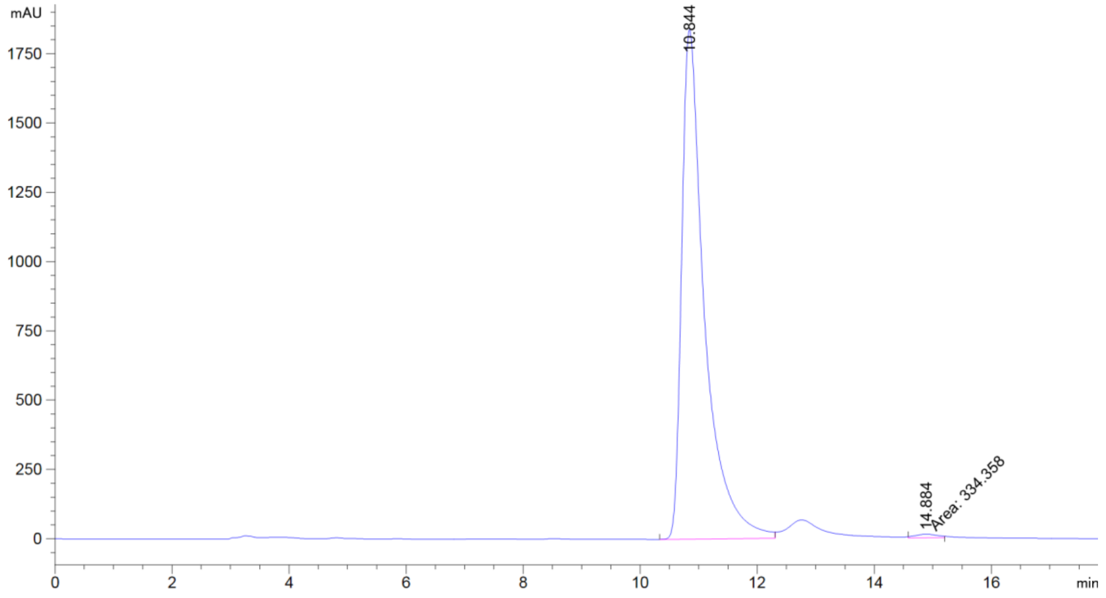

Peak #	RetTime [min]	Type	Width [min]	Area [mAU*s]	Height [mAU]	Area %
1	10.844	BV	0.3958	5.00600e4	1837.68396	99.3365
2	14.884	MM	0.4497	334.35803	12.39116	0.6635

Racemic **3d**

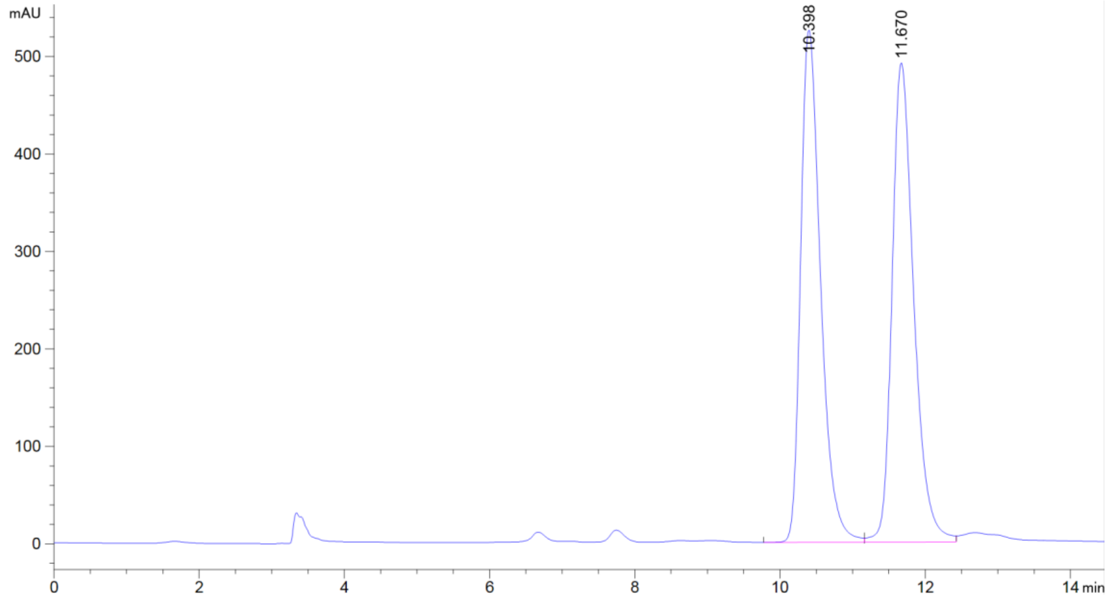

Peak #	RetTime [min]	Type	Width [min]	Area [mAU*s]	Height [mAU]	Area %
1	10.398	BV	0.2919	9997.06348	525.50830	49.4077
2	11.670	VV	0.3170	1.02367e4	491.54431	50.5923

Enantioenriched **3d**

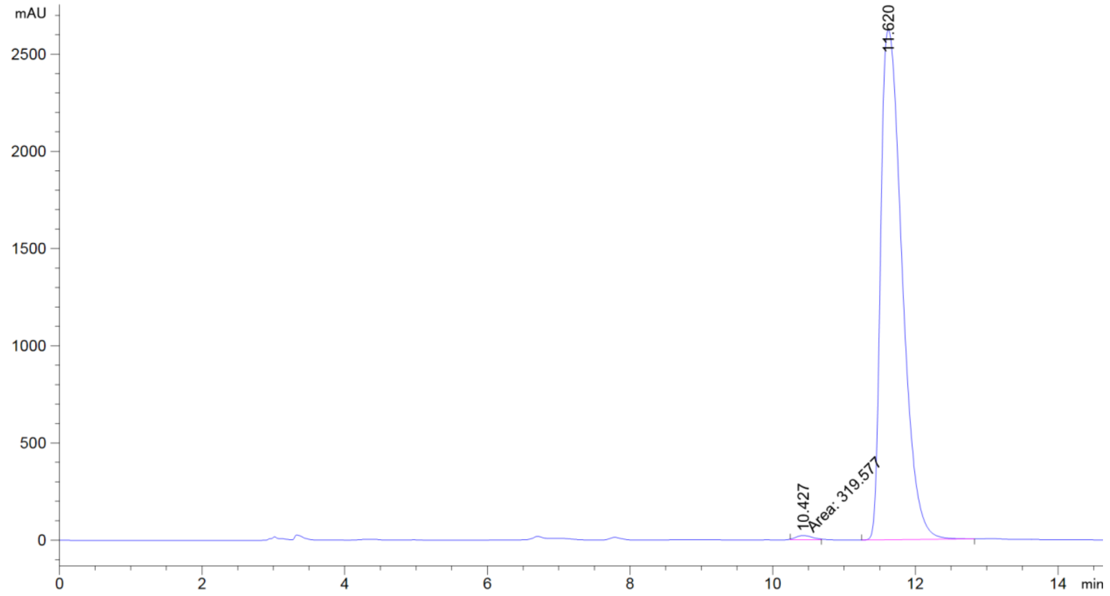

Peak #	RetTime [min]	Type	Width [min]	Area [mAU*s]	Height [mAU]	Area %
1	10.427	MM	0.2550	319.57663	20.88462	0.5839
2	11.620	BB	0.3242	5.44081e4	2621.33179	99.4161

Racemic **3e**

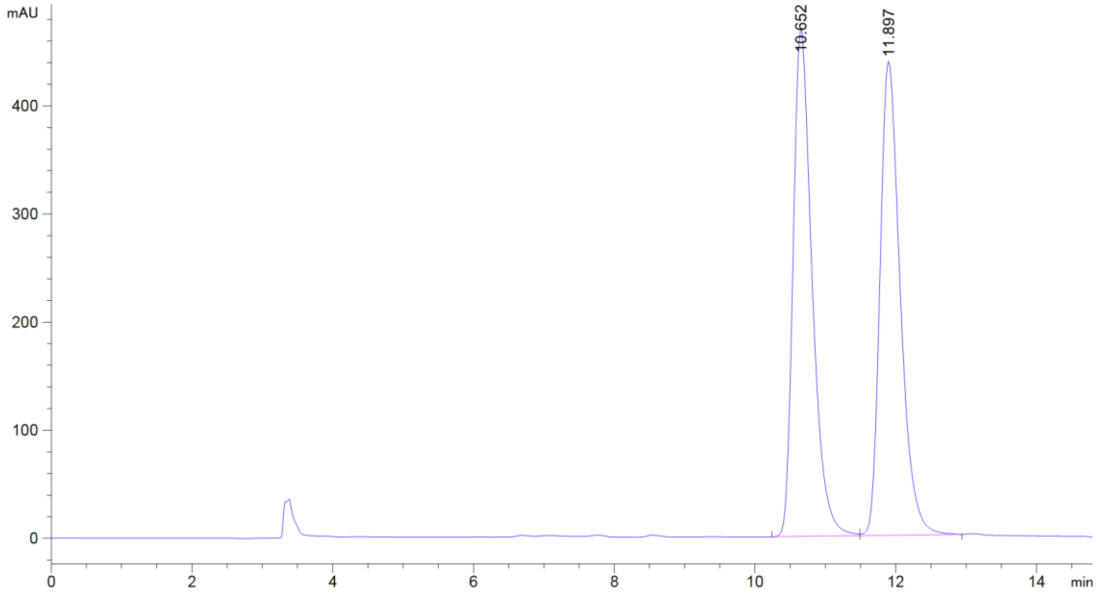

Peak #	RetTime [min]	Type	Width [min]	Area [mAU*s]	Height [mAU]	Area %
1	10.652	BV	0.2844	8772.31348	468.66232	50.0893
2	11.897	VB	0.3046	8741.04883	438.45844	49.9107

Enantioenriched **3e**

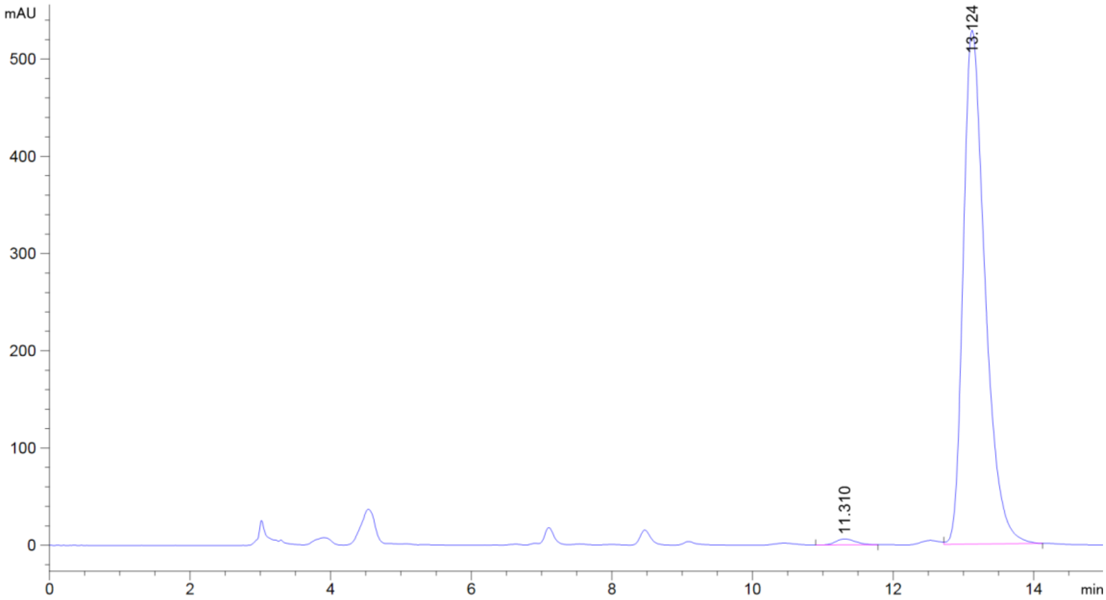

Peak #	RetTime [min]	Type	Width [min]	Area [mAU*s]	Height [mAU]	Area %
1	11.310	BB	0.2972	119.31319	6.23766	1.0379
2	13.124	VB	0.3271	1.13764e4	528.57794	98.9621

Racemic **3f**

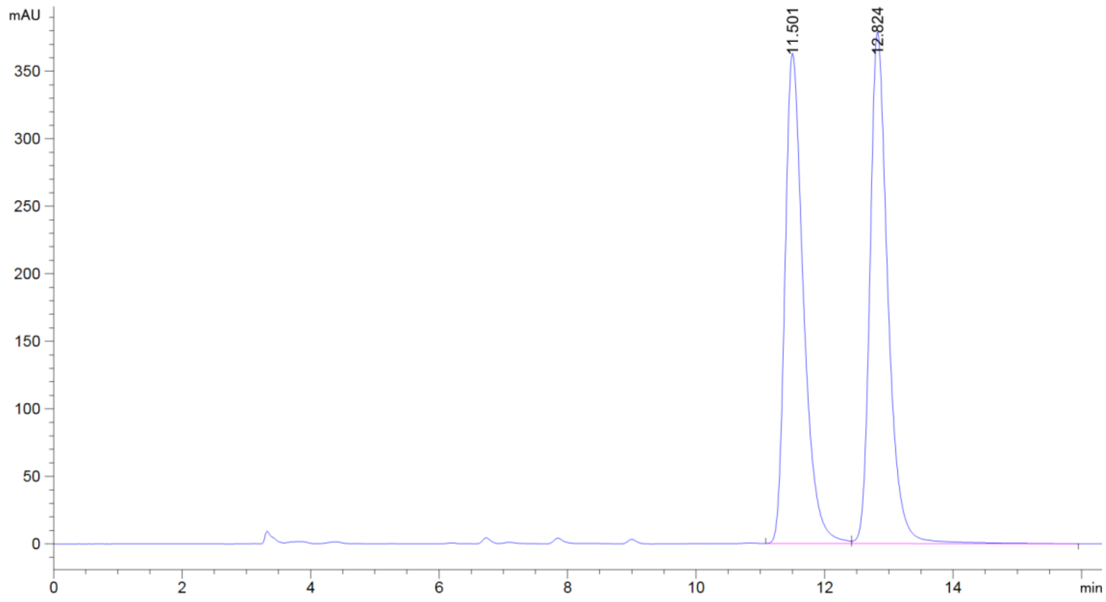

Peak #	RetTime [min]	Type	Width [min]	Area [mAU*s]	Height [mAU]	Area %
1	11.501	BV	0.3031	7253.21387	363.03268	49.6879
2	12.824	VB	0.2921	7344.33447	378.89169	50.3121

Enantioenriched **3f**

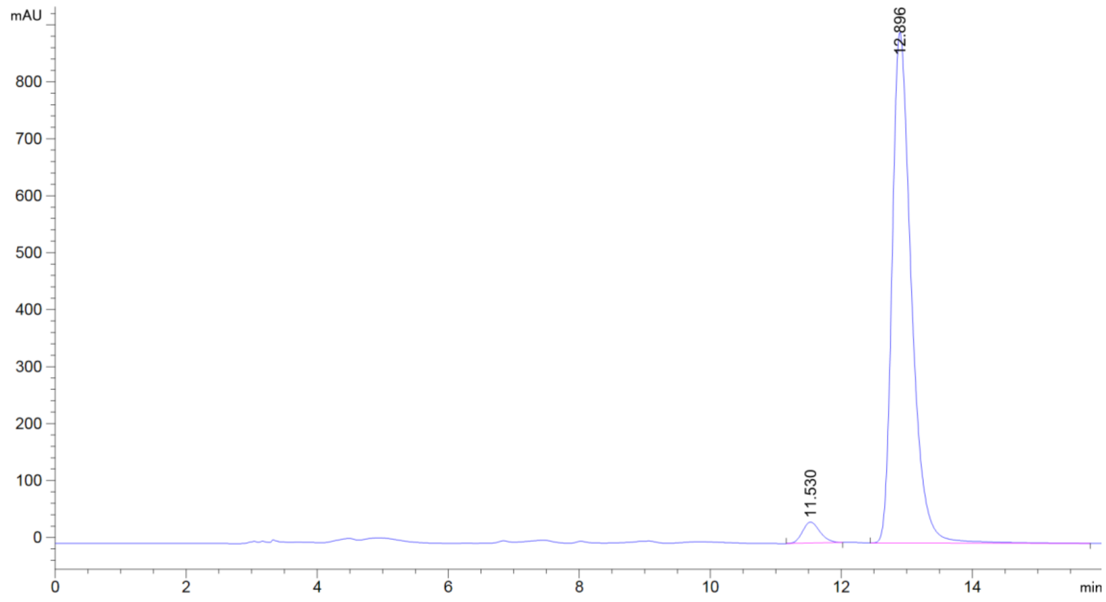

Peak #	RetTime [min]	Type	Width [min]	Area [mAU*s]	Height [mAU]	Area %
1	11.530	BB	0.2850	680.93665	36.94440	3.5898
2	12.896	BB	0.3099	1.82879e4	896.76563	96.4102

Racemic **3g**

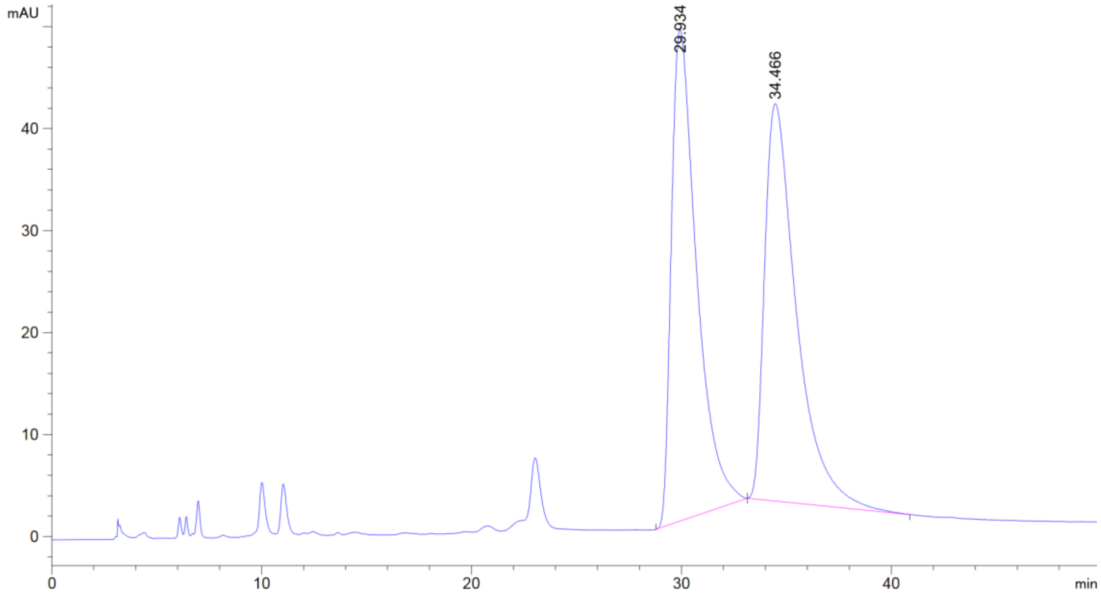

Peak #	RetTime [min]	Type	Width [min]	Area [mAU*s]	Height [mAU]	Area %
1	29.934	BB	1.2156	4045.57275	48.18189	49.4774
2	34.466	BB	1.4408	4131.03906	38.96763	50.5226

Enantioenriched **3g**

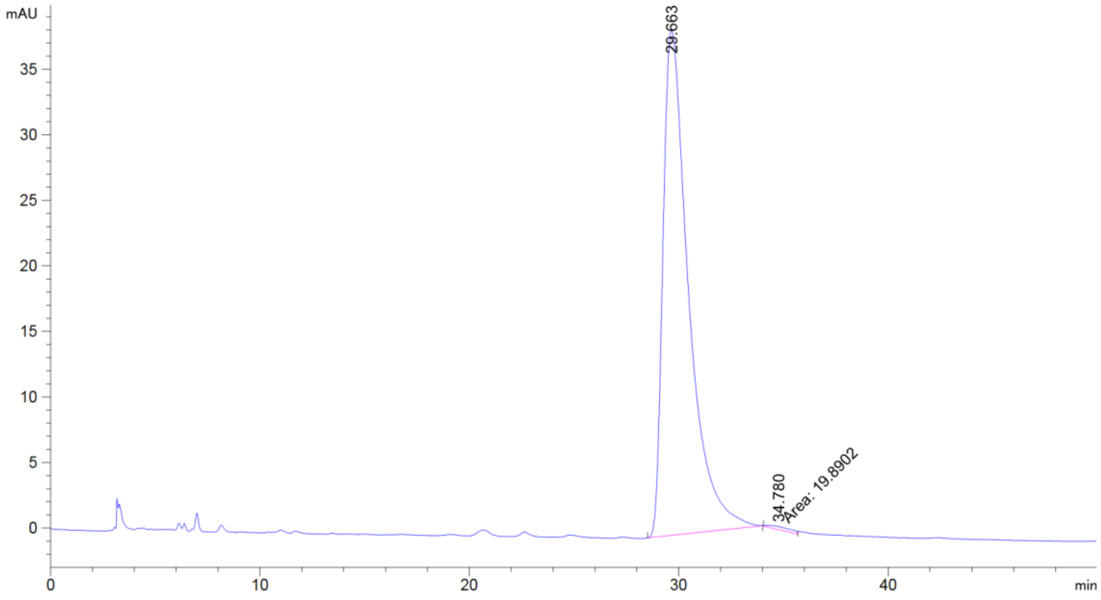

Peak #	RetTime [min]	Type	Width [min]	Area [mAU*s]	Height [mAU]	Area %
1	29.663	BB	1.1736	3273.30249	38.51019	99.3960
2	34.780	MM	1.3102	19.89018	2.53017e-1	0.6040

Racemic **3h**

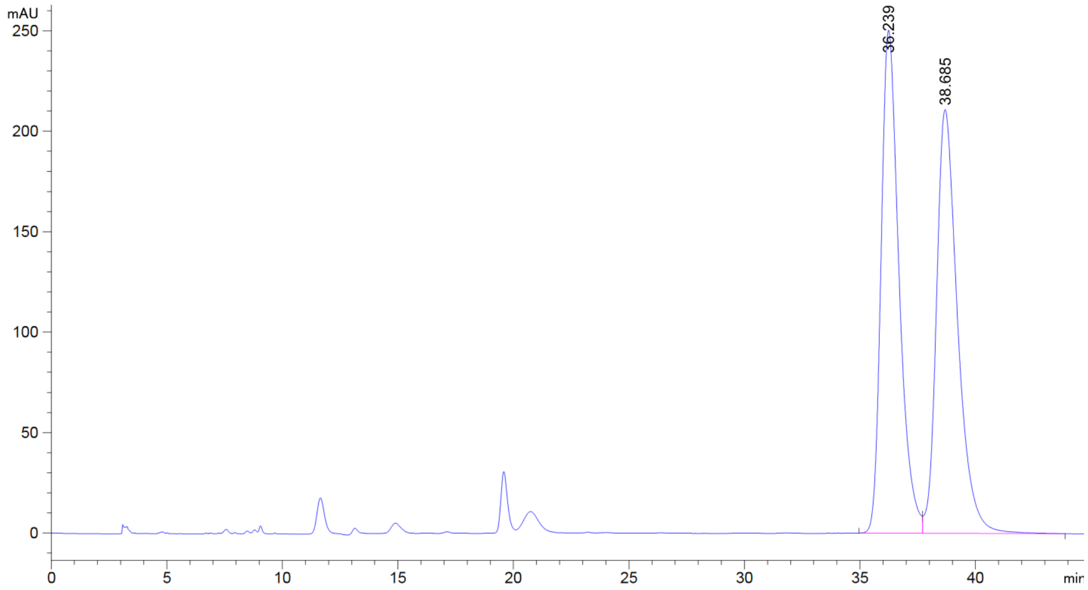

Peak #	RetTime [min]	Type	Width [min]	Area [mAU*s]	Height [mAU]	Area %
1	36.239	BV	0.8199	1.35195e4	250.38400	50.1747
2	38.685	VB	0.9607	1.34254e4	210.90175	49.8253

Enantioenriched **3h**

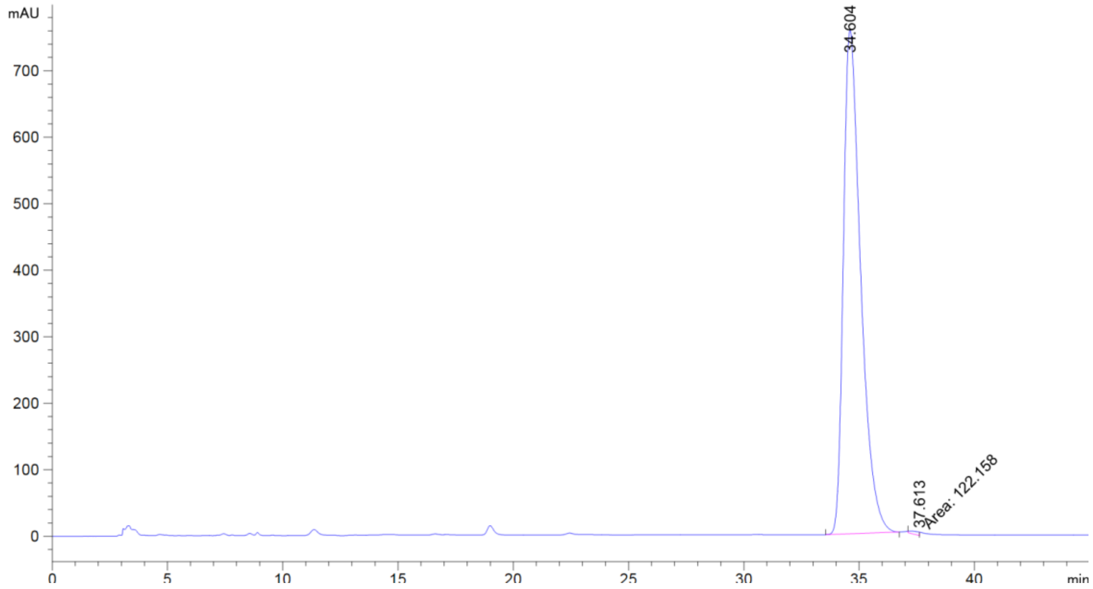

Peak #	RetTime [min]	Type	Width [min]	Area [mAU*s]	Height [mAU]	Area %
1	34.604	BB	0.7902	3.96329e4	757.73712	99.6927
2	37.613	MM	0.4087	122.15807	4.98135	0.3073

Racemic **3i**

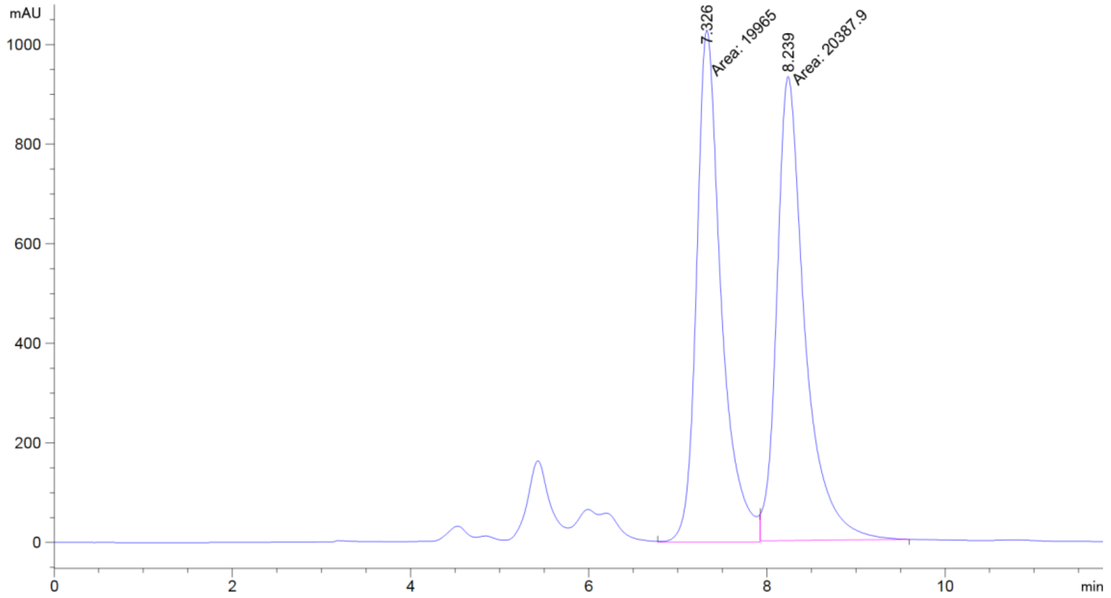

Peak #	RetTime [min]	Type	Width [min]	Area [mAU*s]	Height [mAU]	Area %
1	7.326	MM	0.3235	1.99650e4	1028.47827	49.4761
2	8.239	MM	0.3646	2.03879e4	931.89697	50.5239

Enantioenriched **3i**

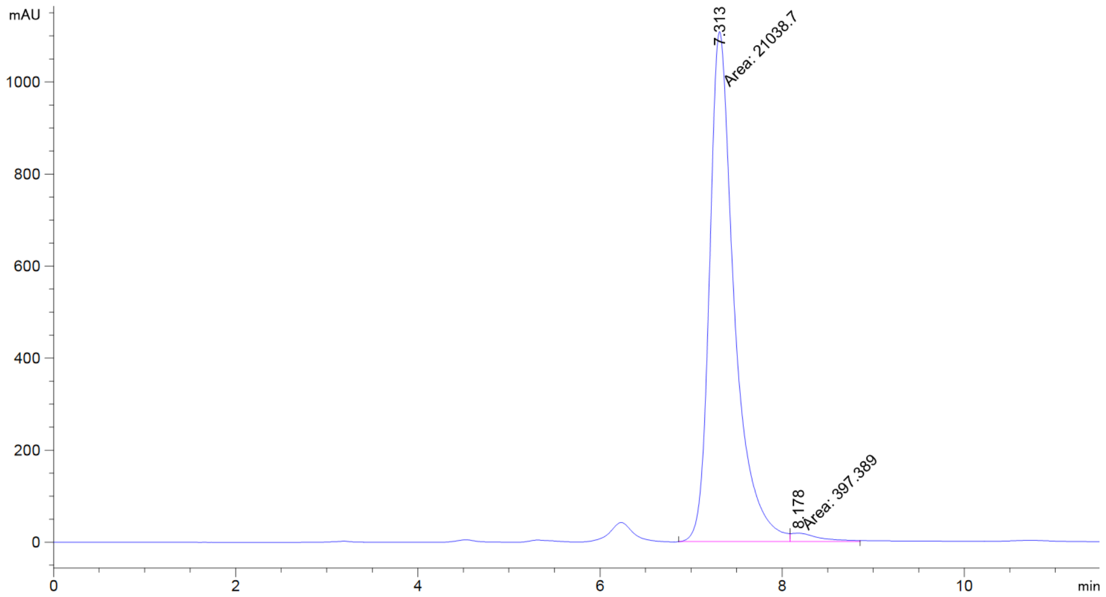

Peak #	RetTime [min]	Type	Width [min]	Area [mAU*s]	Height [mAU]	Area %
1	7.313	MM	0.3164	2.10387e4	1108.10461	98.1462
2	8.178	MM	0.3644	397.38910	18.17388	1.8538

Racemic **3j**

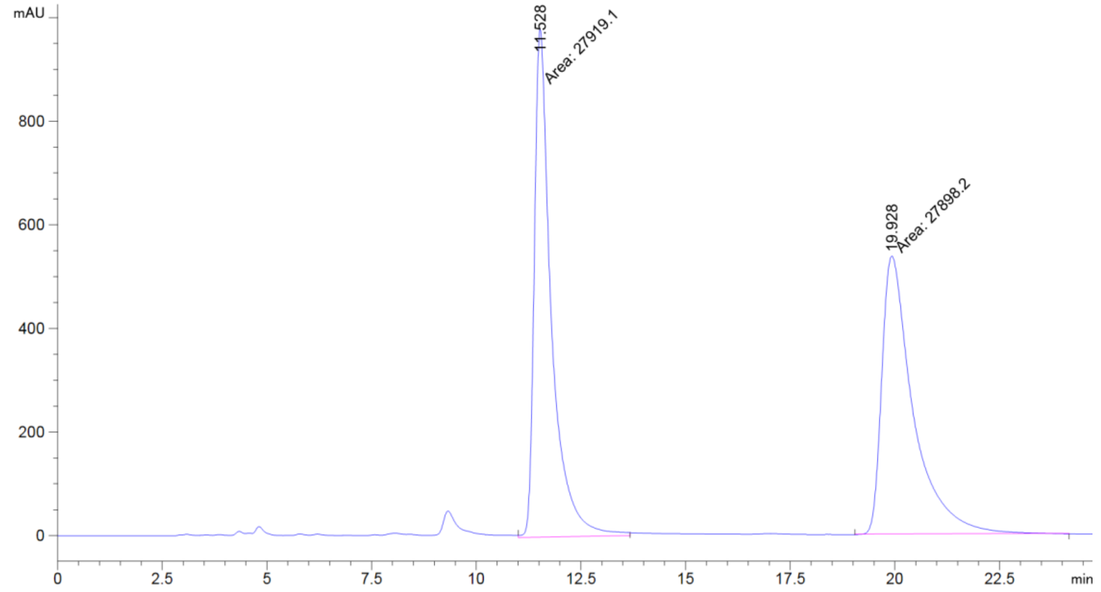

Peak #	RetTime [min]	Type	Width [min]	Area [mAU*s]	Height [mAU]	Area %
1	11.528	MM	0.4749	2.79191e4	979.86011	50.0187
2	19.928	MM	0.8667	2.78982e4	536.48987	49.9813

Enantioenriched **3j**

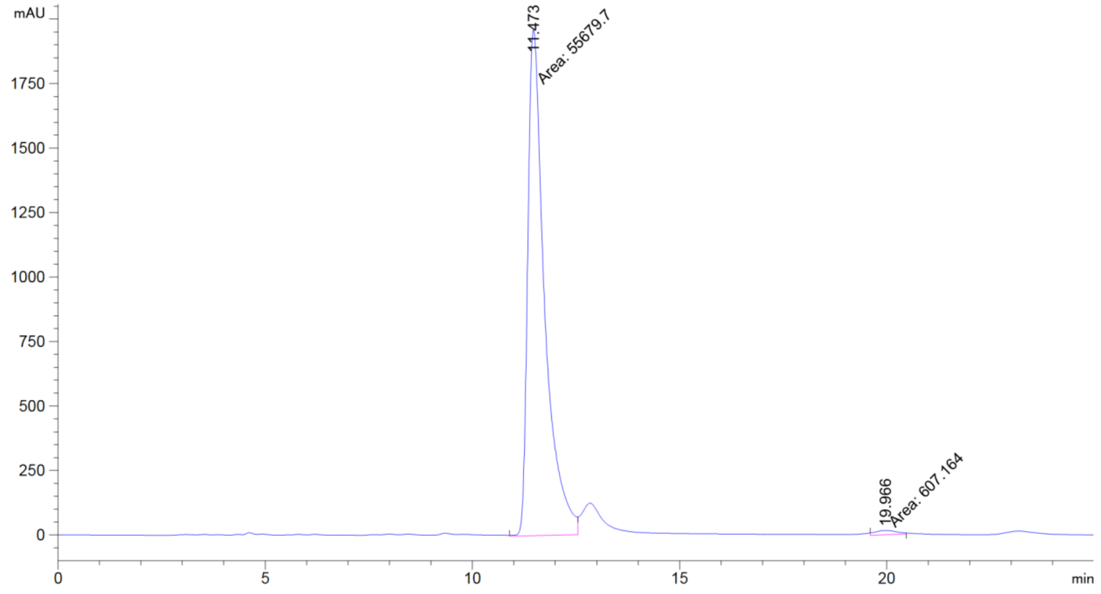

Peak #	RetTime [min]	Type	Width [min]	Area [mAU*s]	Height [mAU]	Area %
1	11.473	MM	0.4731	5.56797e4	1961.33008	98.9213
2	19.966	MM	0.6117	607.16425	16.54200	1.0787

Racemic **3k**

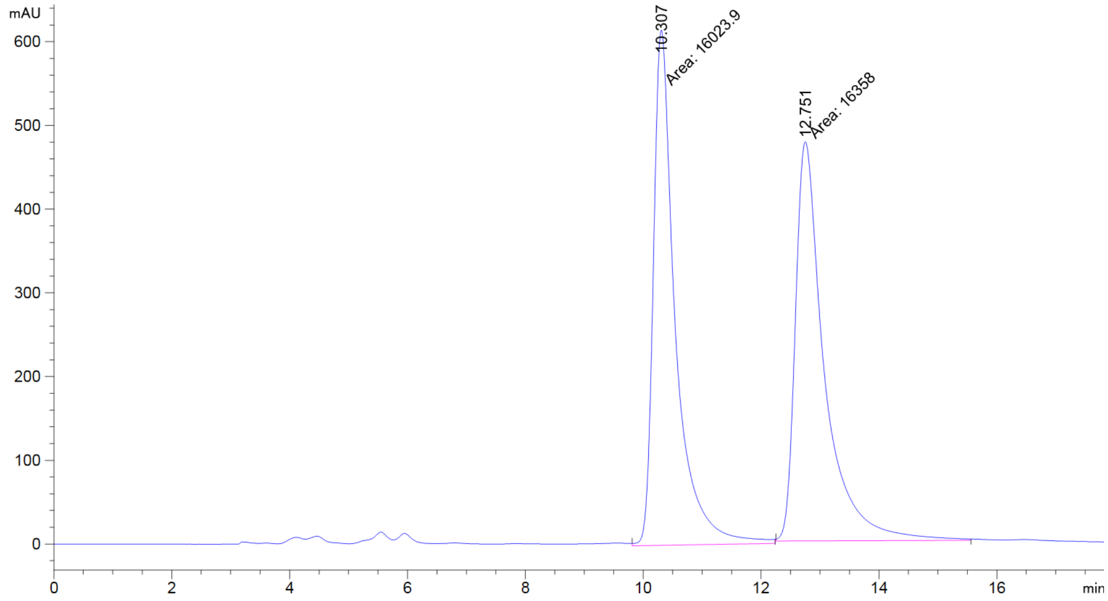

Peak #	RetTime [min]	Type	Width [min]	Area [mAU*s]	Height [mAU]	Area %
1	10.307	MM	0.4339	1.60239e4	615.45892	49.4841
2	12.751	MM	0.5719	1.63580e4	476.74402	50.5159

Enantioenriched **3k**

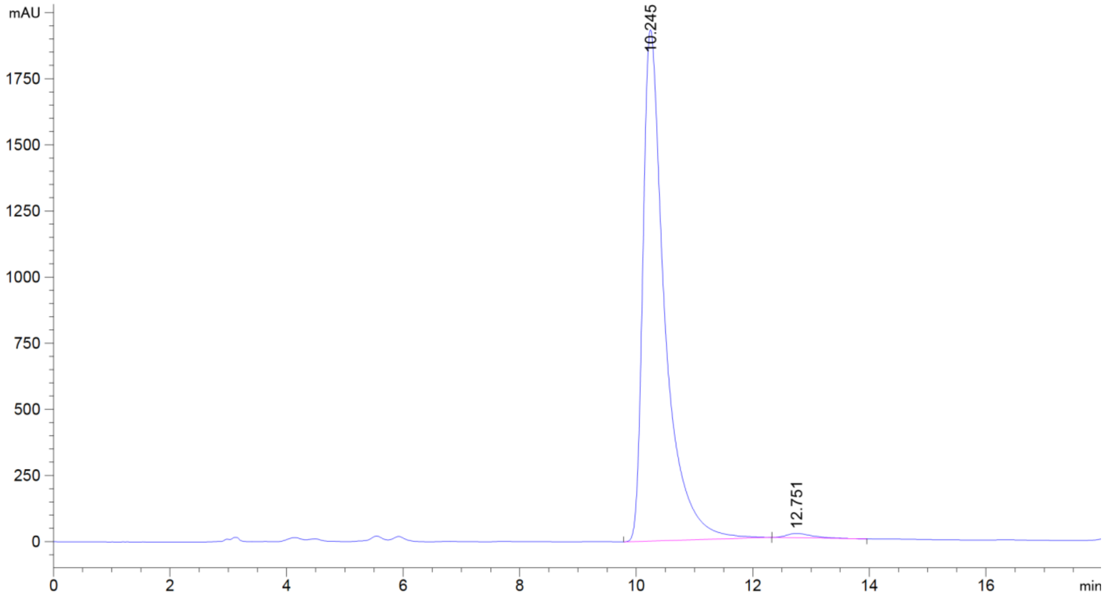

Peak #	RetTime [min]	Type	Width [min]	Area [mAU*s]	Height [mAU]	Area %
1	10.245	BB	0.3823	5.04134e4	1932.92432	99.0207
2	12.751	BB	0.4563	498.57062	16.32659	0.9793

Racemic **3l**

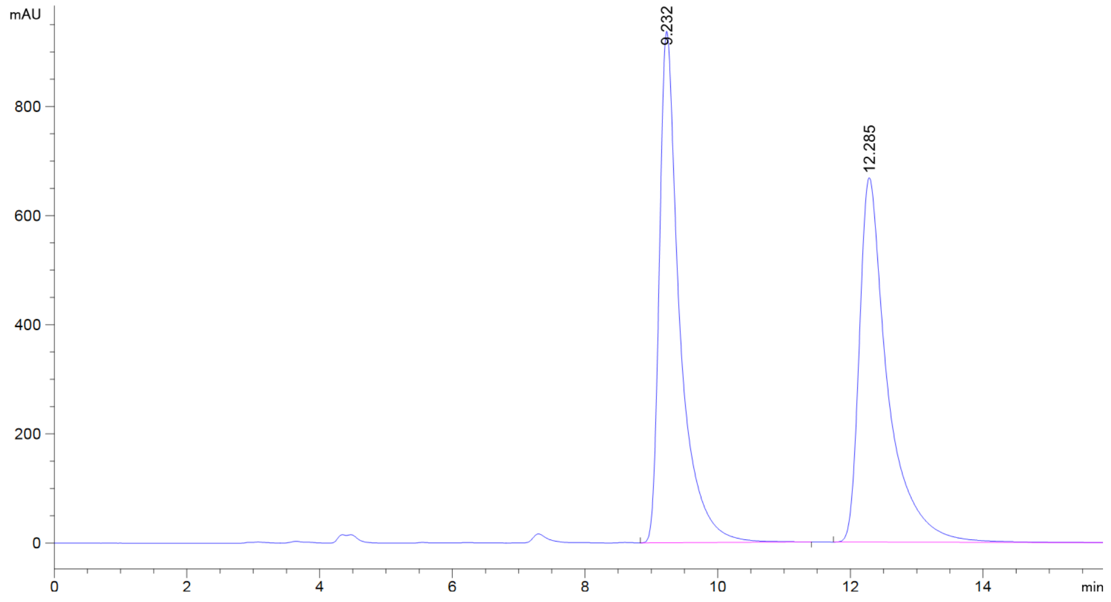

Peak #	RetTime [min]	Type	Width [min]	Area [mAU*s]	Height [mAU]	Area %
1	9.232	BB	0.3111	2.00176e4	937.40094	50.3171
2	12.285	BBA	0.4314	1.97653e4	667.56805	49.6829

Enantioenriched **3l**

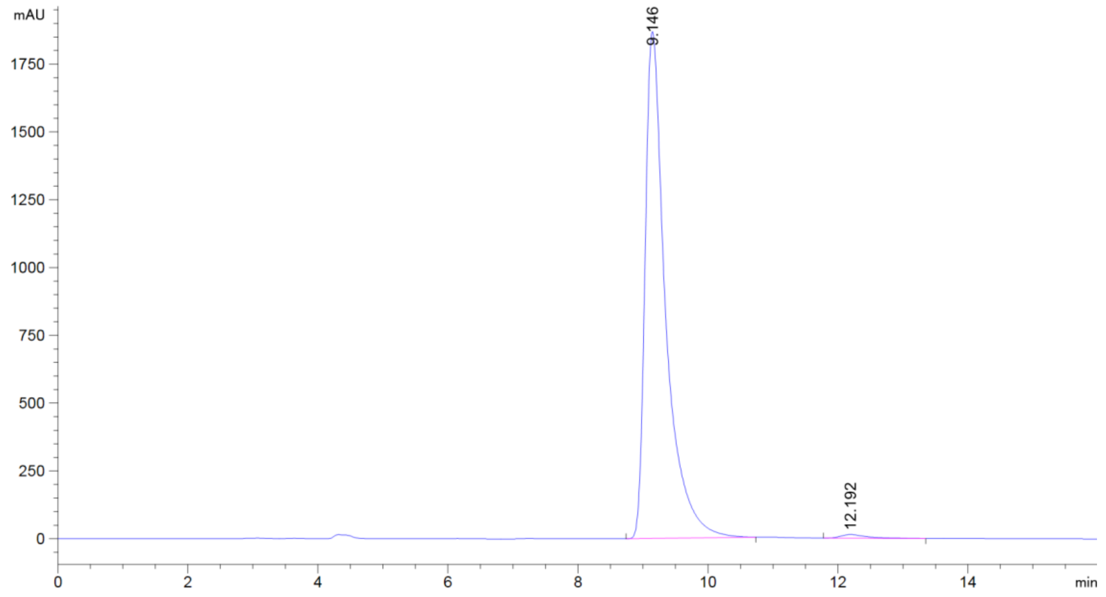

Peak #	RetTime [min]	Type	Width [min]	Area [mAU*s]	Height [mAU]	Area %
1	9.146	BB	0.3221	4.10248e4	1868.89490	99.1256
2	12.192	BB	0.3982	361.88556	13.35232	0.8744

Racemic **3m**

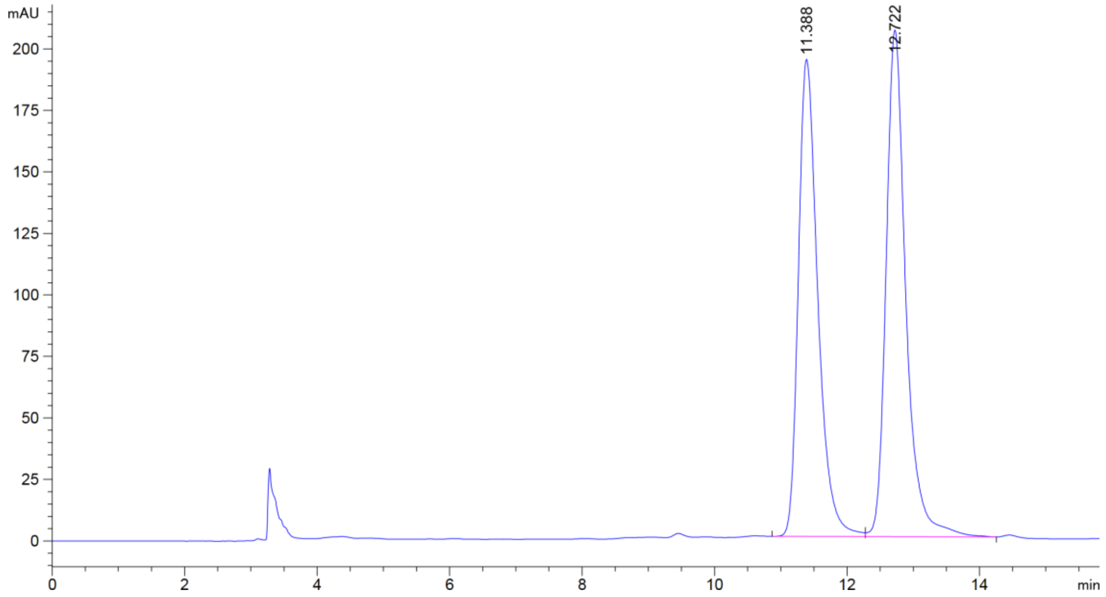

Peak #	RetTime [min]	Type	Width [min]	Area [mAU*s]	Height [mAU]	Area %
1	11.388	BV	0.3101	3958.46313	193.94432	48.1117
2	12.722	VB	0.3139	4269.18994	205.94028	51.8883

Enantioenriched **3m**

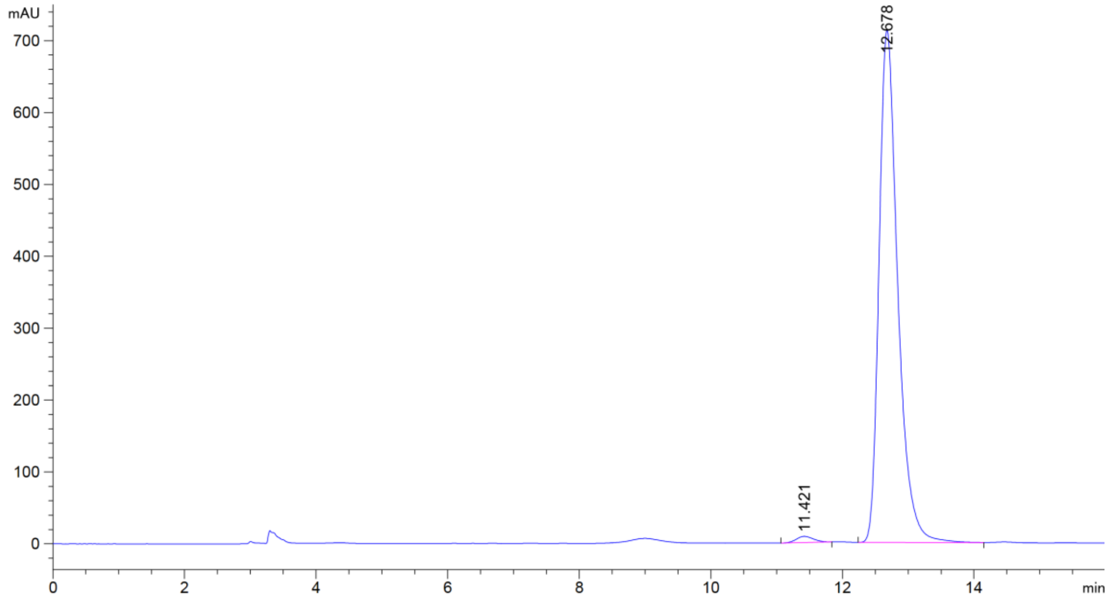

Peak #	RetTime [min]	Type	Width [min]	Area [mAU*s]	Height [mAU]	Area %
1	11.421	BB	0.2779	152.81087	8.49497	1.0749
2	12.678	BB	0.3003	1.40641e4	712.49487	98.9251

Racemic **3n**

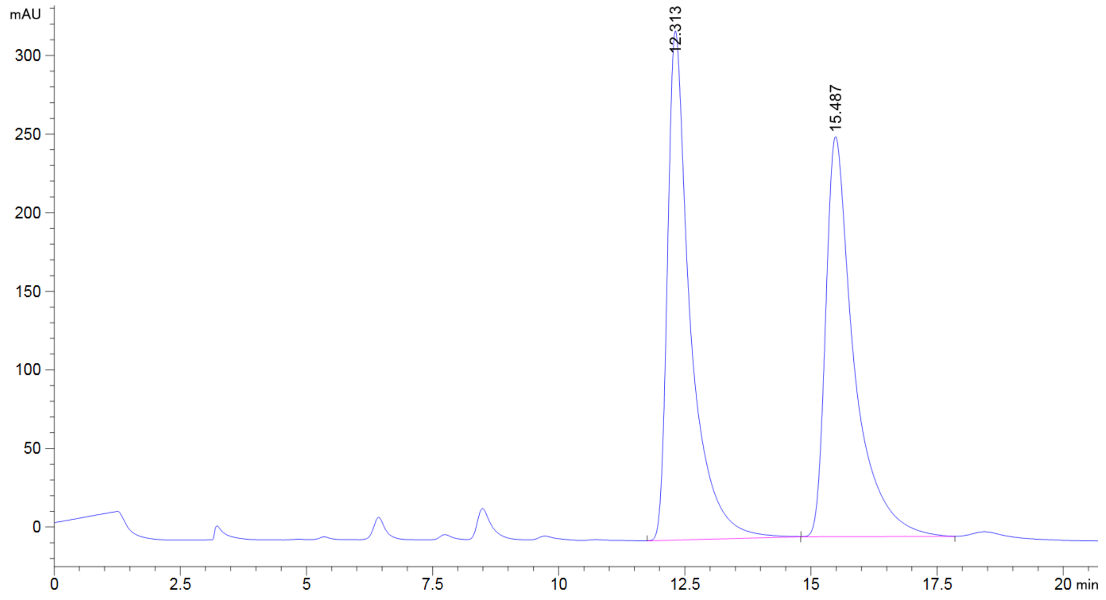

Peak #	RetTime [min]	Type	Width [min]	Area [mAU*s]	Height [mAU]	Area %
1	12.313	BB	0.4568	1.01792e4	323.75281	50.6555
2	15.487	BB	0.5668	9915.76270	254.35667	49.3445

Enantioenriched **3n**

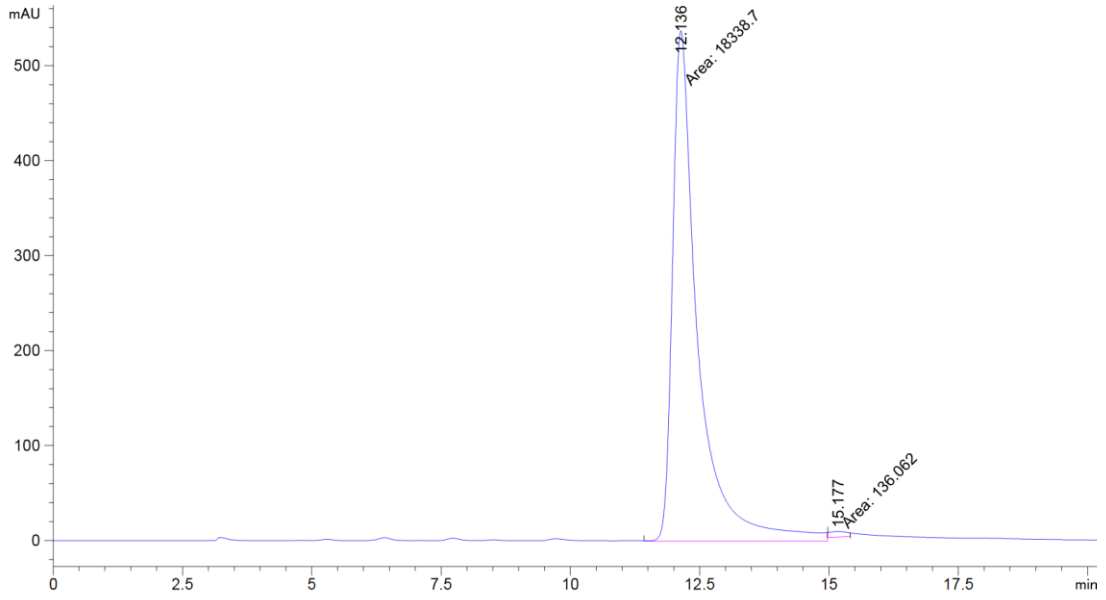

Peak #	RetTime [min]	Type	Width [min]	Area [mAU*s]	Height [mAU]	Area %
1	12.136	MM	0.5684	1.83387e4	537.69177	99.2635
2	15.177	MM	0.3966	136.06169	5.71821	0.7365

Racemic **3o**

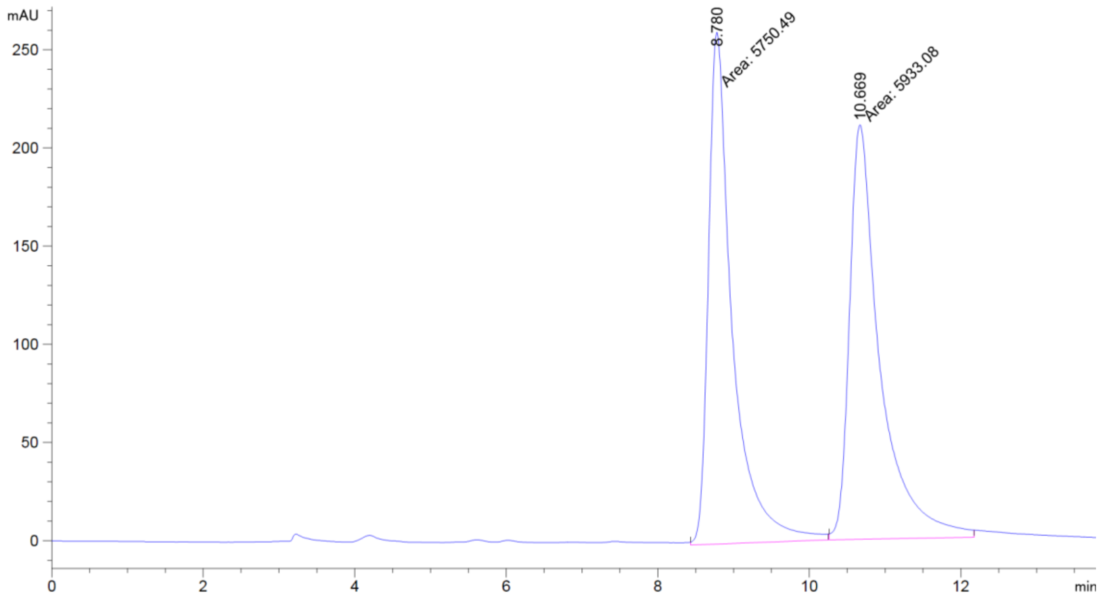

Peak #	RetTime [min]	Type	Width [min]	Area [mAU*s]	Height [mAU]	Area %
1	8.780	MM	0.3676	5750.49023	260.71390	49.2186
2	10.669	MM	0.4683	5933.07520	211.14032	50.7814

Enantioenriched **3o**

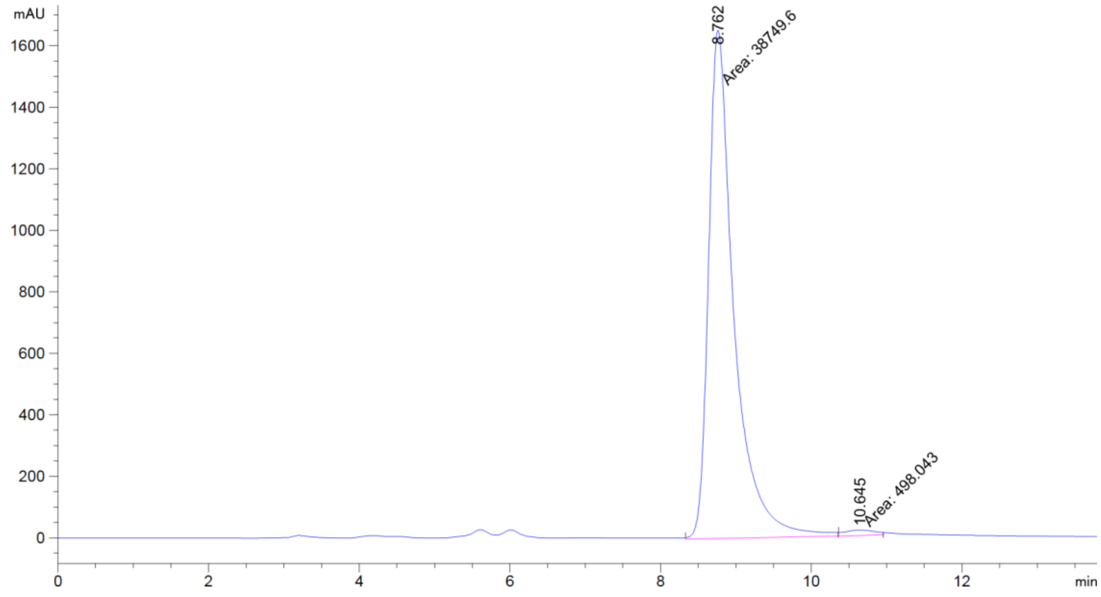

Peak #	RetTime [min]	Type	Width [min]	Area [mAU*s]	Height [mAU]	Area %
1	8.762	MM	0.3910	3.87496e4	1651.66248	98.7310
2	10.645	MM	0.4722	498.04333	17.57867	1.2690

Racemic **3p**

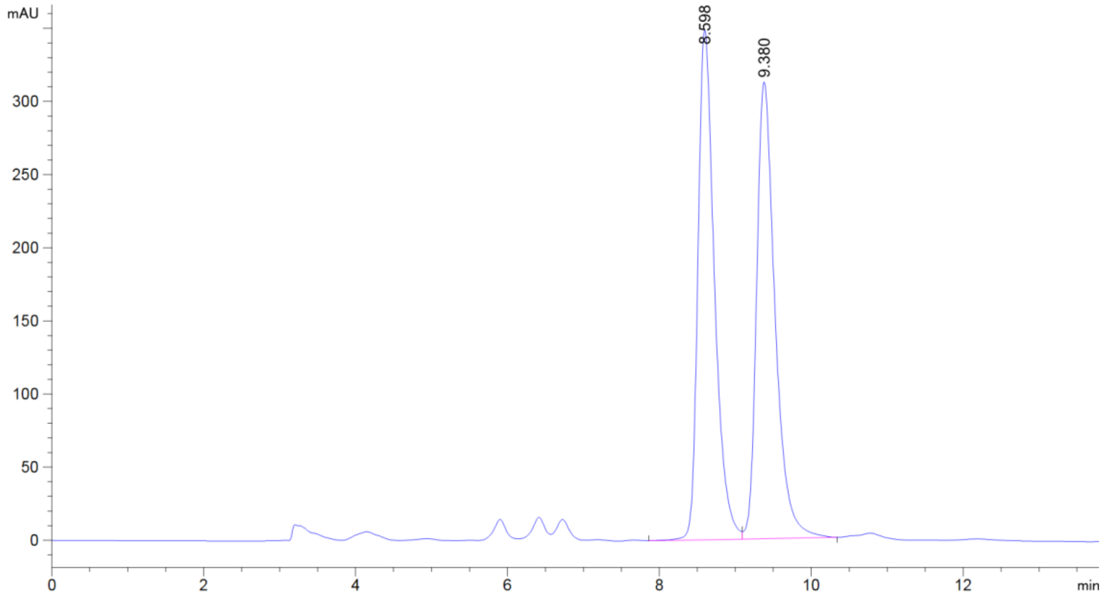

Peak #	RetTime [min]	Type	Width [min]	Area [mAU*s]	Height [mAU]	Area %
1	8.598	BV	0.2308	5279.66553	348.39252	50.0586
2	9.380	VB	0.2548	5267.30518	312.28577	49.9414

Enantioenriched **3p**

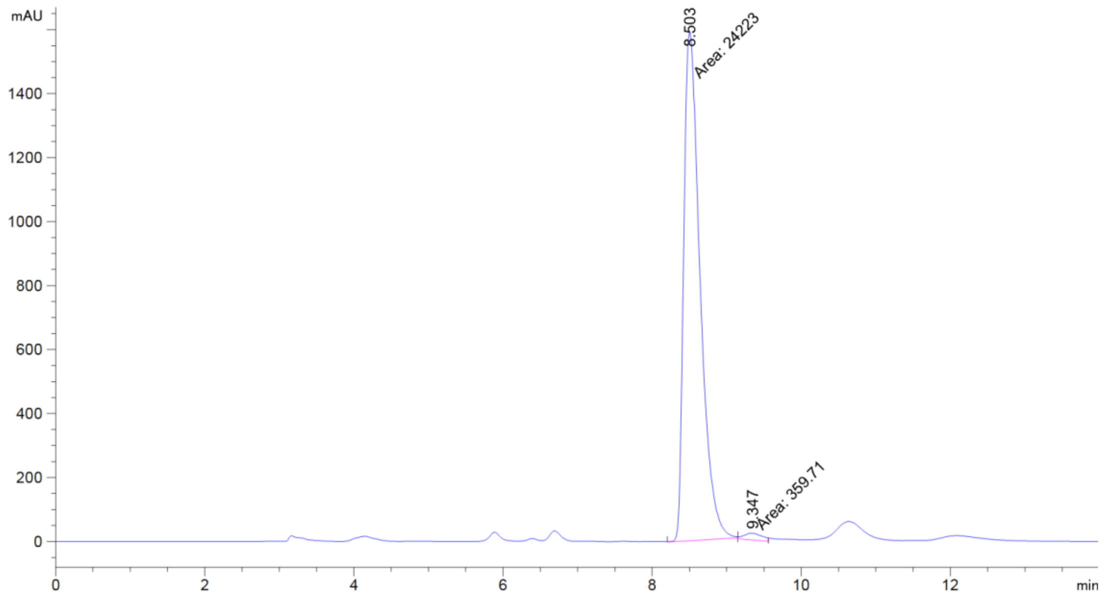

Peak #	RetTime [min]	Type	Width [min]	Area [mAU*s]	Height [mAU]	Area %
1	8.503	MM	0.2540	2.42230e4	1589.26599	98.5367
2	9.347	MM	0.2878	359.71017	20.83055	1.4633

Racemic **3q**

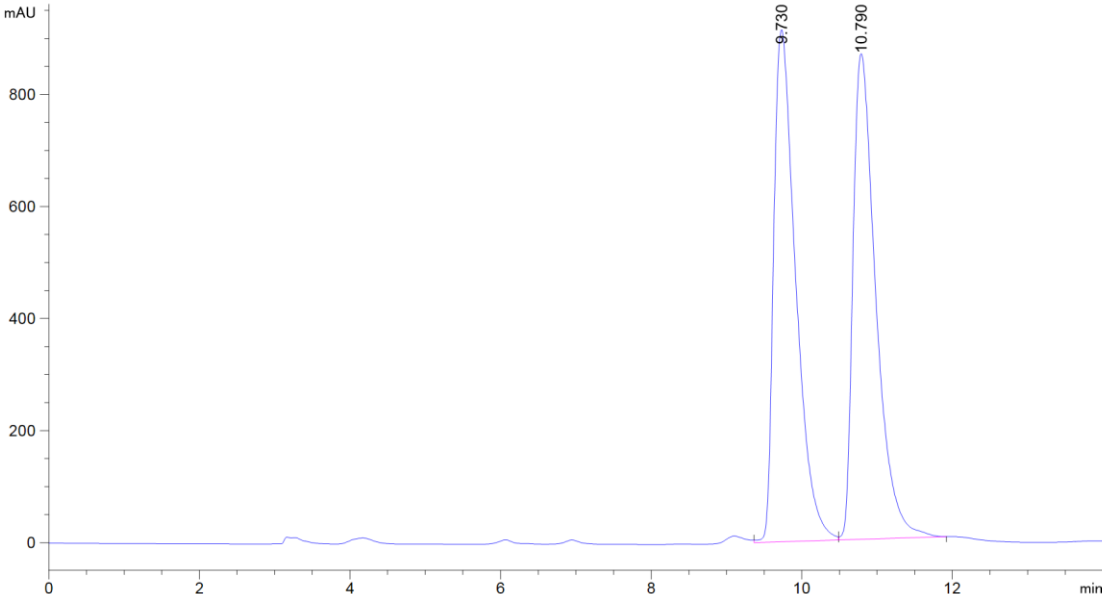

Peak #	RetTime [min]	Type	Width [min]	Area [mAU*s]	Height [mAU]	Area %
1	9.730	VV	0.3084	1.85297e4	914.36755	50.3623
2	10.790	VB	0.3218	1.82631e4	866.94238	49.6377

Enantioenriched **3q**

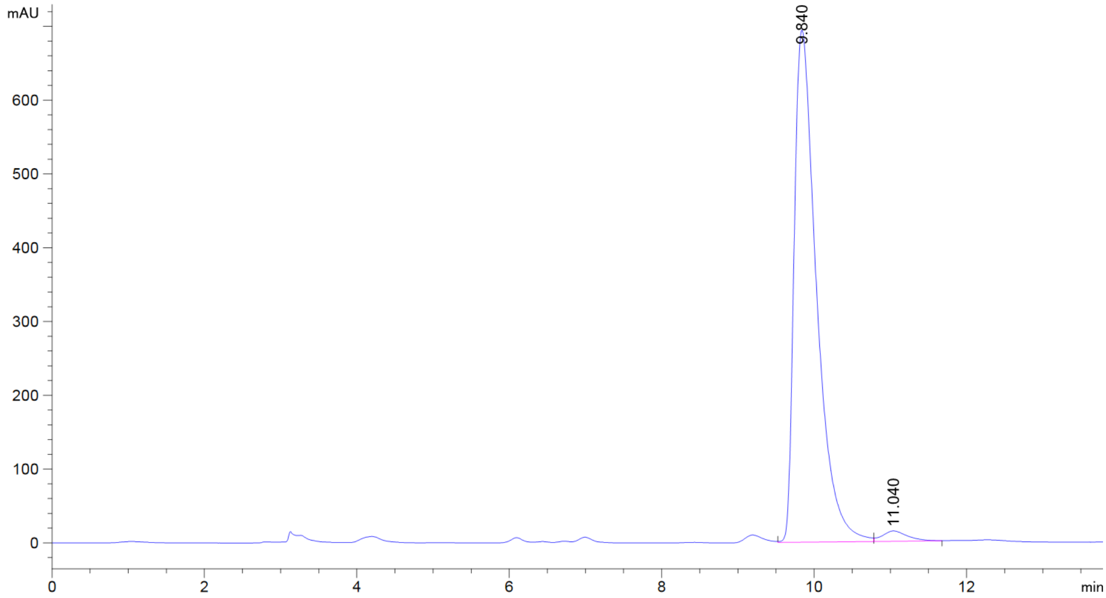

Peak #	RetTime [min]	Type	Width [min]	Area [mAU*s]	Height [mAU]	Area %
1	9.840	VV	0.3066	1.40819e4	694.24622	97.7821
2	11.040	VB	0.3401	319.41101	14.00332	2.2179

Racemic **3r**

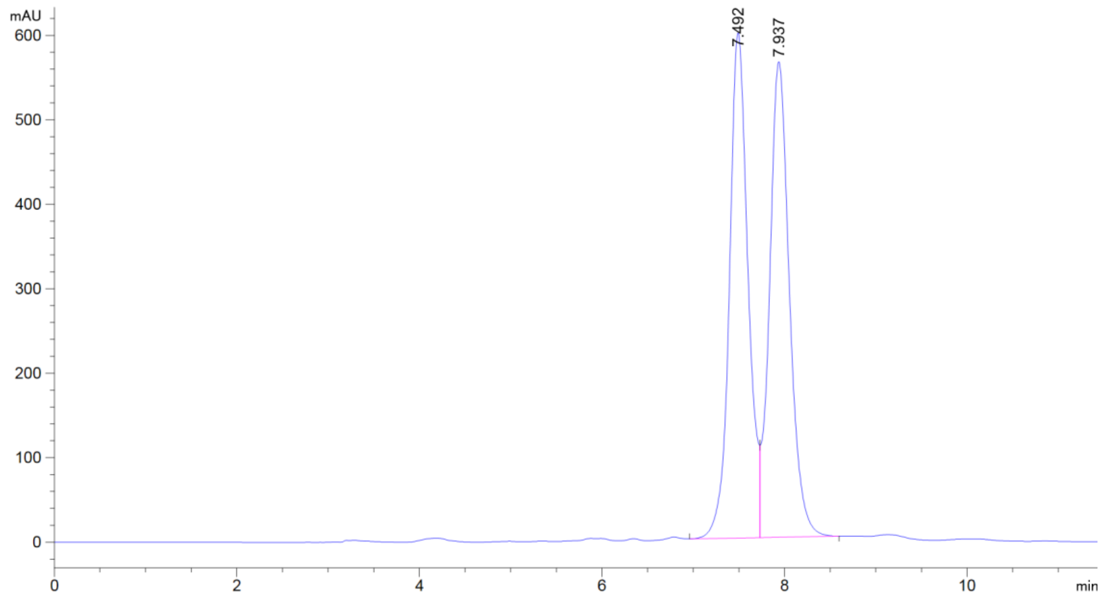

Peak #	RetTime [min]	Type	Width [min]	Area [mAU*s]	Height [mAU]	Area %
1	7.492	BV	0.2131	8465.09277	598.34686	49.8402
2	7.937	VB	0.2305	8519.36621	563.16846	50.1598

Enantioenriched **3r**

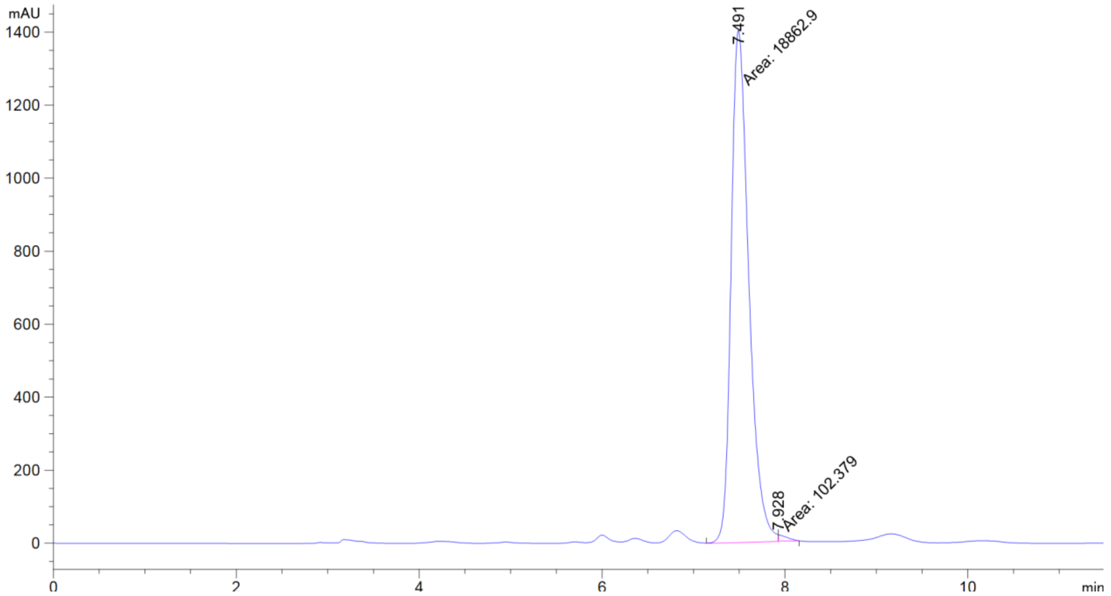

Peak #	RetTime [min]	Type	Width [min]	Area [mAU*s]	Height [mAU]	Area %
1	7.491	MM	0.2239	1.88629e4	1403.84253	99.4602
2	7.928	MM	0.0747	102.37944	17.18518	0.5398

Racemic **3s**

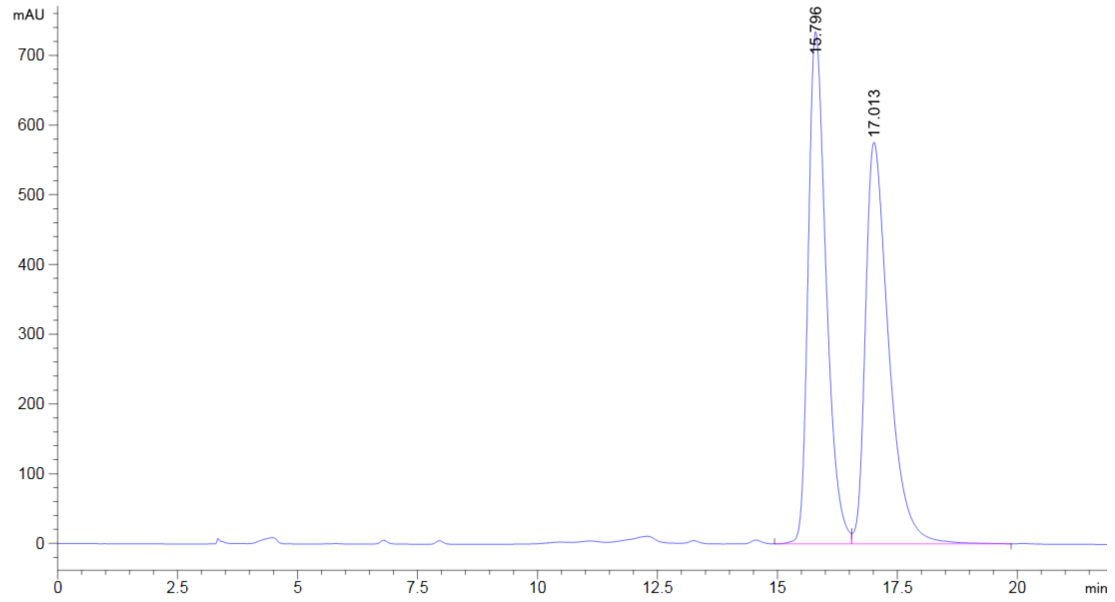

Peak #	RetTime [min]	Type	Width [min]	Area [mAU*s]	Height [mAU]	Area %
1	15.796	BV	0.3980	1.91407e4	734.63055	49.5715
2	17.013	VB	0.5104	1.94716e4	576.03461	50.4285

Enantioenriched **3s**

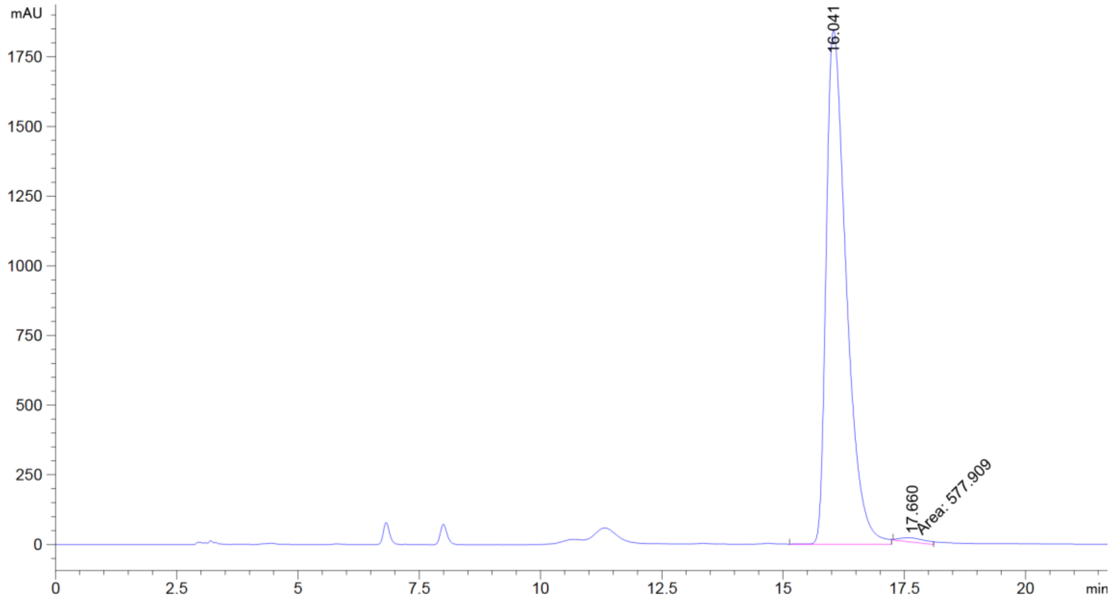

Peak #	RetTime [min]	Type	Width [min]	Area [mAU*s]	Height [mAU]	Area %
1	16.041	BV	0.4403	5.31633e4	1844.54504	98.9246
2	17.660	MM	0.6269	577.90942	15.36457	1.0754

Racemic **3t**

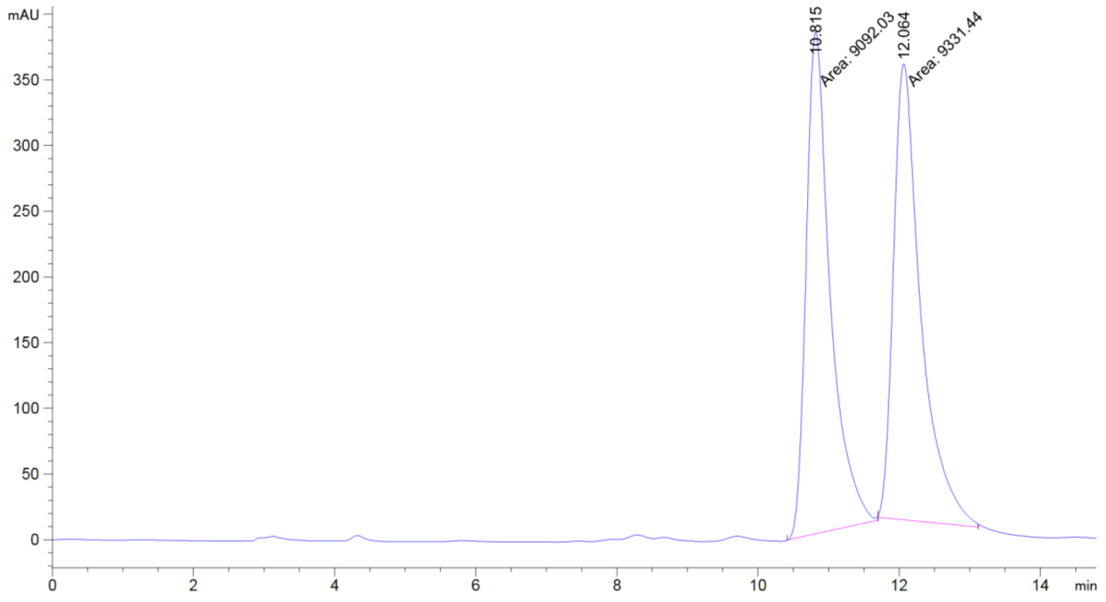

Peak #	RetTime [min]	Type	Width [min]	Area [mAU*s]	Height [mAU]	Area %
1	10.815	MM	0.3966	9092.02734	382.10431	49.3502
2	12.064	MM	0.4482	9331.44043	346.98788	50.6498

Enantioenriched **3t**

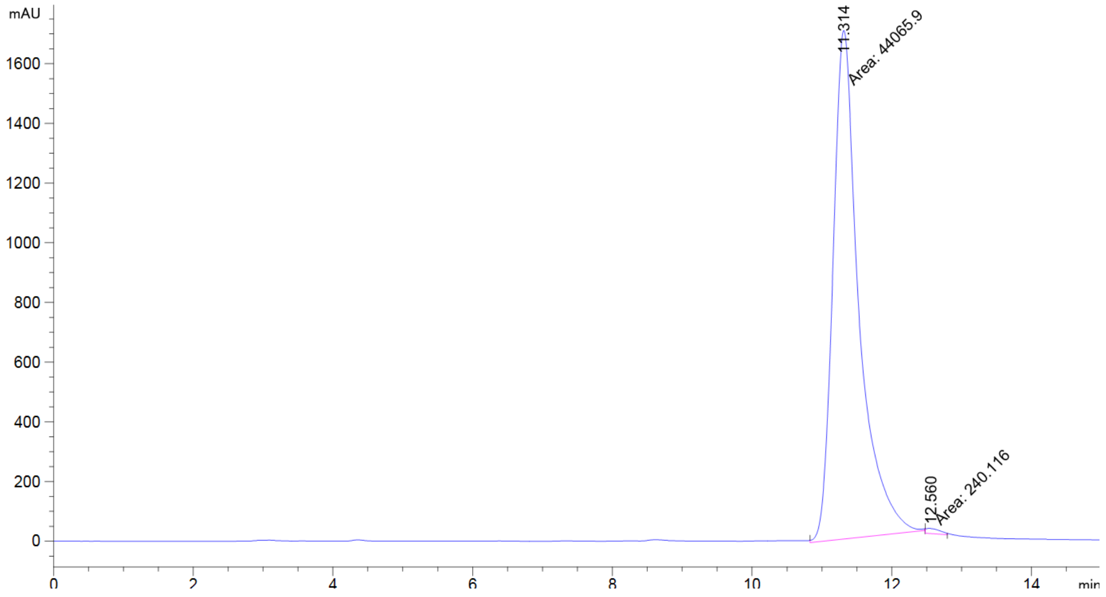

Peak #	RetTime [min]	Type	Width [min]	Area [mAU*s]	Height [mAU]	Area %
1	11.314	MM	0.4308	4.40659e4	1704.63525	99.4581
2	12.560	MM	0.2388	240.11555	16.75808	0.5419

Racemic **3u**

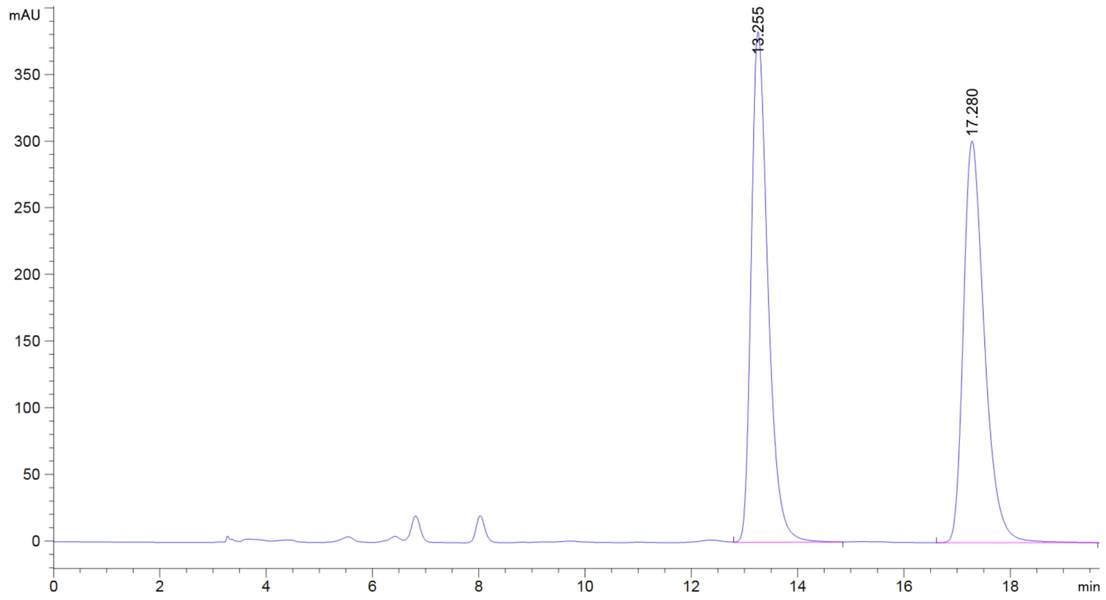

Peak #	RetTime [min]	Type	Width [min]	Area [mAU*s]	Height [mAU]	Area %
1	13.255	VB	0.3350	8370.58594	382.91998	50.2794
2	17.280	BBA	0.4205	8277.56250	301.33051	49.7206

Enantioenriched **3u**

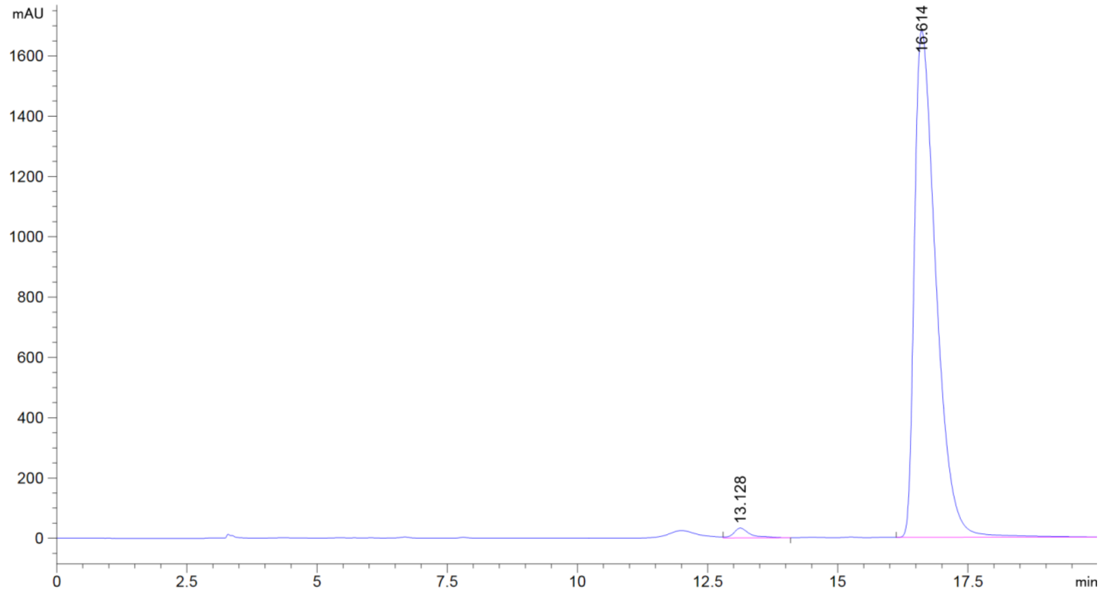

Peak #	RetTime [min]	Type	Width [min]	Area [mAU*s]	Height [mAU]	Area %
1	13.128	VB	0.3132	702.40729	32.61851	1.4350
2	16.614	BBA	0.4388	4.82474e4	1681.40613	98.5650

Racemic **3v**

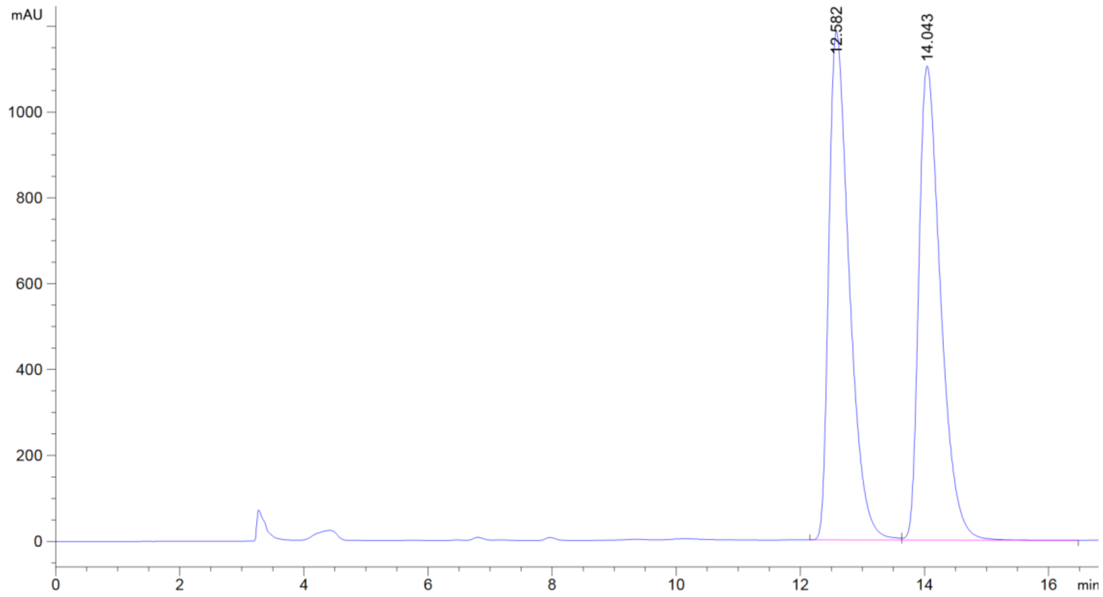

Peak #	RetTime [min]	Type	Width [min]	Area [mAU*s]	Height [mAU]	Area %
1	12.582	BV	0.3484	2.70371e4	1184.08167	50.1335
2	14.043	VB	0.3722	2.68931e4	1104.15295	49.8665

Enantioenriched **3v**

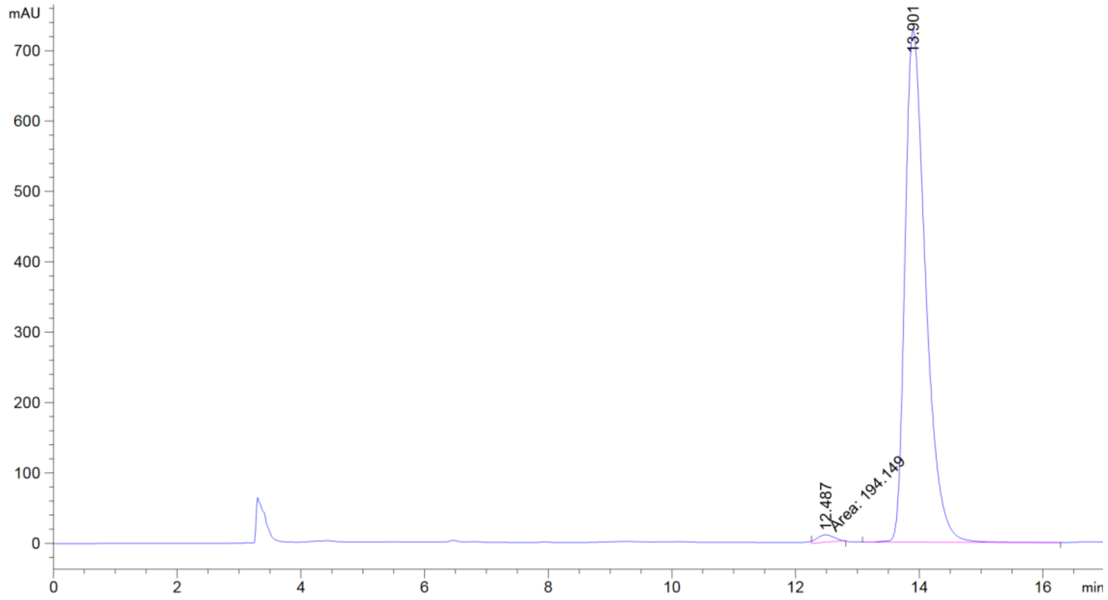

Peak #	RetTime [min]	Type	Width [min]	Area [mAU*s]	Height [mAU]	Area %
1	12.487	MM	0.3100	194.14893	10.43882	1.1395
2	13.901	BB	0.3540	1.68440e4	727.77344	98.8605

Racemic **3w**

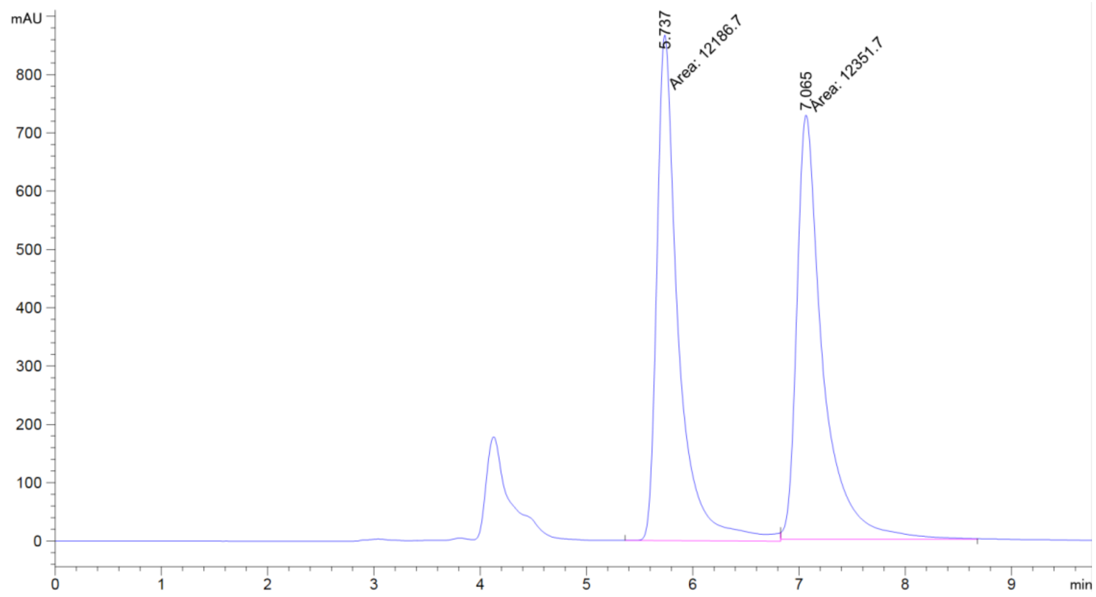

Peak #	RetTime [min]	Type	Width [min]	Area [mAU*s]	Height [mAU]	Area %
1	5.737	MM	0.2340	1.21867e4	867.87457	49.6638
2	7.065	MM	0.2829	1.23517e4	727.67188	50.3362

Enantioenriched **3w**

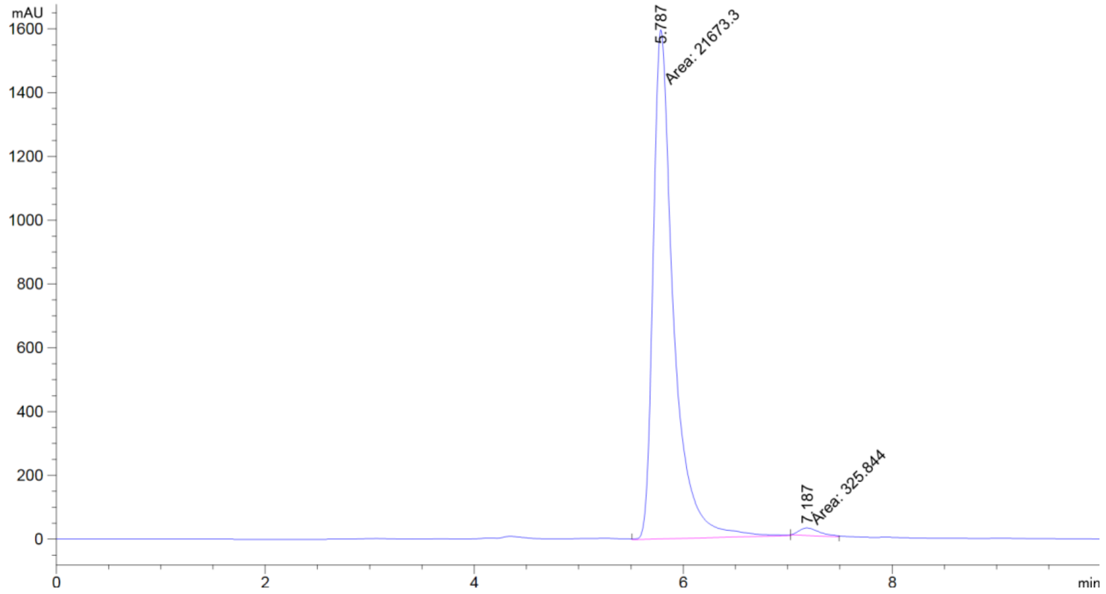

Peak #	RetTime [min]	Type	Width [min]	Area [mAU*s]	Height [mAU]	Area %
1	5.787	MM	0.2262	2.16733e4	1596.73730	98.5188
2	7.187	MM	0.2289	325.84415	23.72260	1.4812

Racemic **3x**

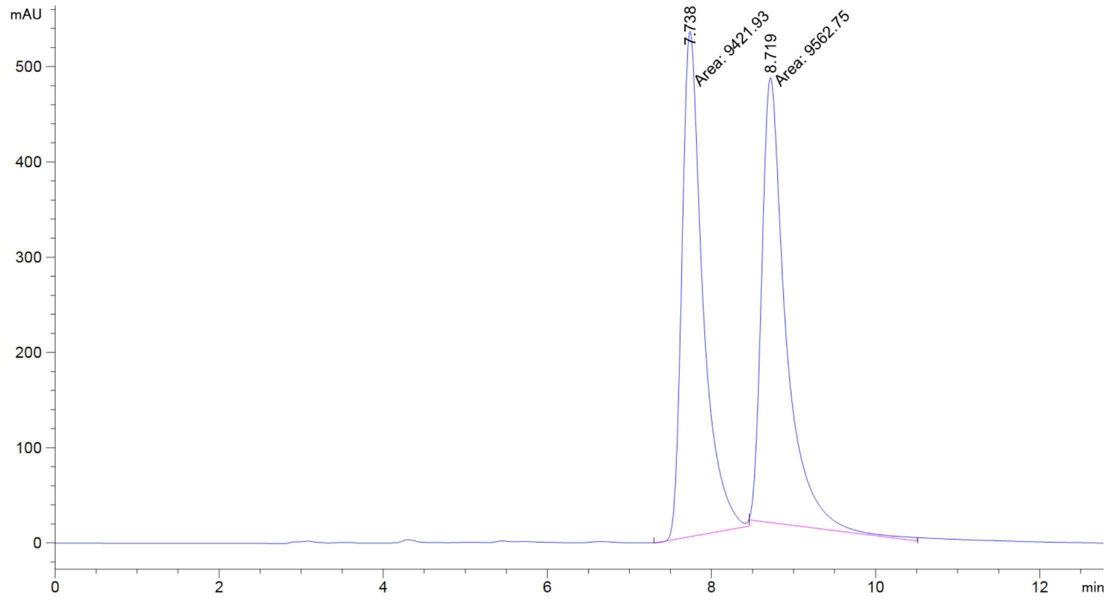

Peak #	RetTime [min]	Type	Width [min]	Area [mAU*s]	Height [mAU]	Area %
1	7.738	MM	0.2959	9421.92969	530.77502	49.6291
2	8.719	MM	0.3411	9562.74902	467.24365	50.3709

Enantioenriched **3x**

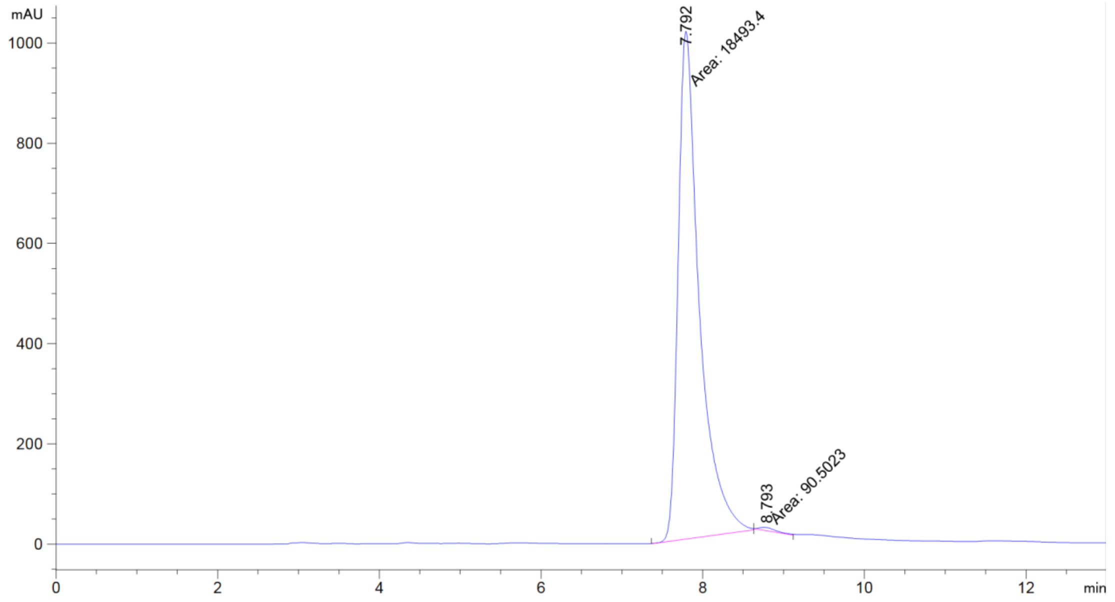

Peak #	RetTime [min]	Type	Width [min]	Area [mAU*s]	Height [mAU]	Area %
1	7.792	MM	0.3040	1.84934e4	1013.86816	99.5130
2	8.793	MM	0.2399	90.50228	6.28646	0.4870

Racemic **3y**

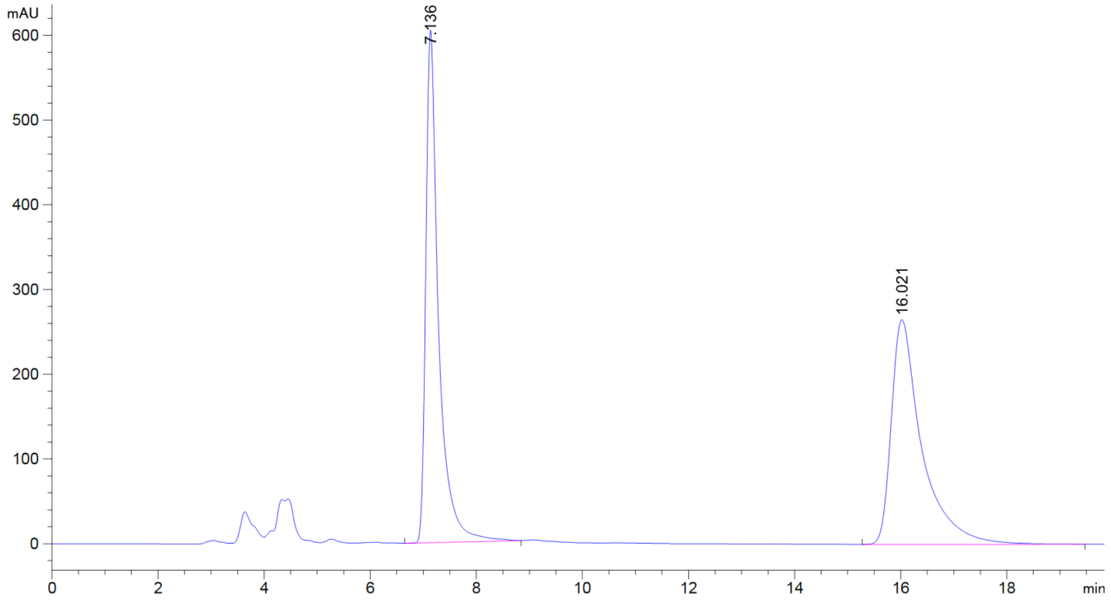

Peak #	RetTime [min]	Type	Width [min]	Area [mAU*s]	Height [mAU]	Area %
1	7.136	BB	0.2434	1.01393e4	605.25330	49.0005
2	16.021	BB	0.5763	1.05530e4	265.17331	50.9995

Enantioenriched **3y**

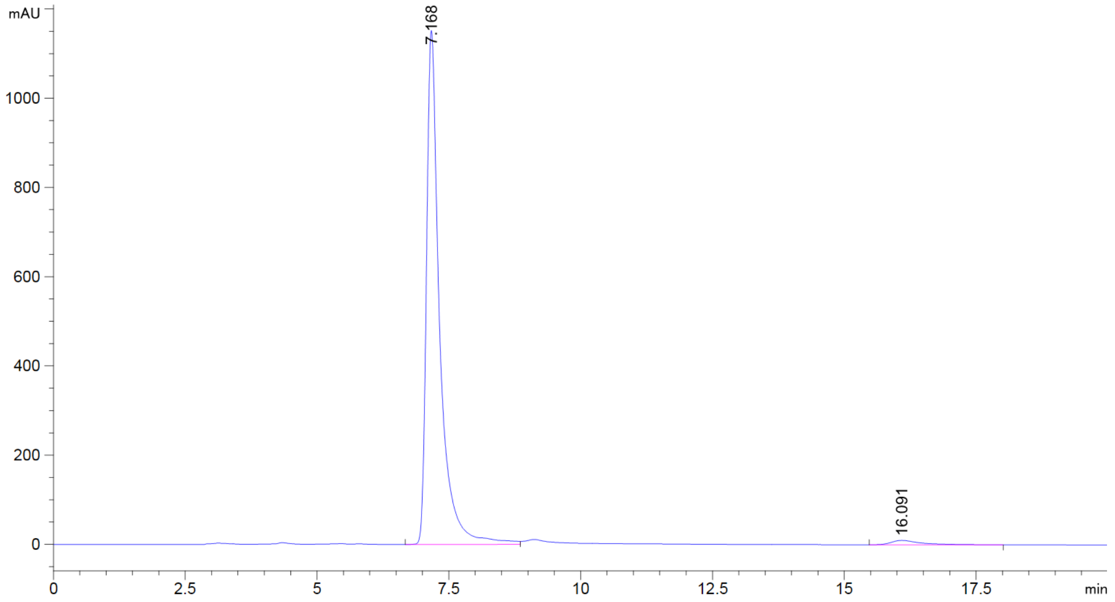

Peak #	RetTime [min]	Type	Width [min]	Area [mAU*s]	Height [mAU]	Area %
1	7.168	BV	0.2555	2.00991e4	1152.35254	98.1094
2	16.091	BB	0.5485	387.32547	9.94216	1.8906

Racemic **3zd**

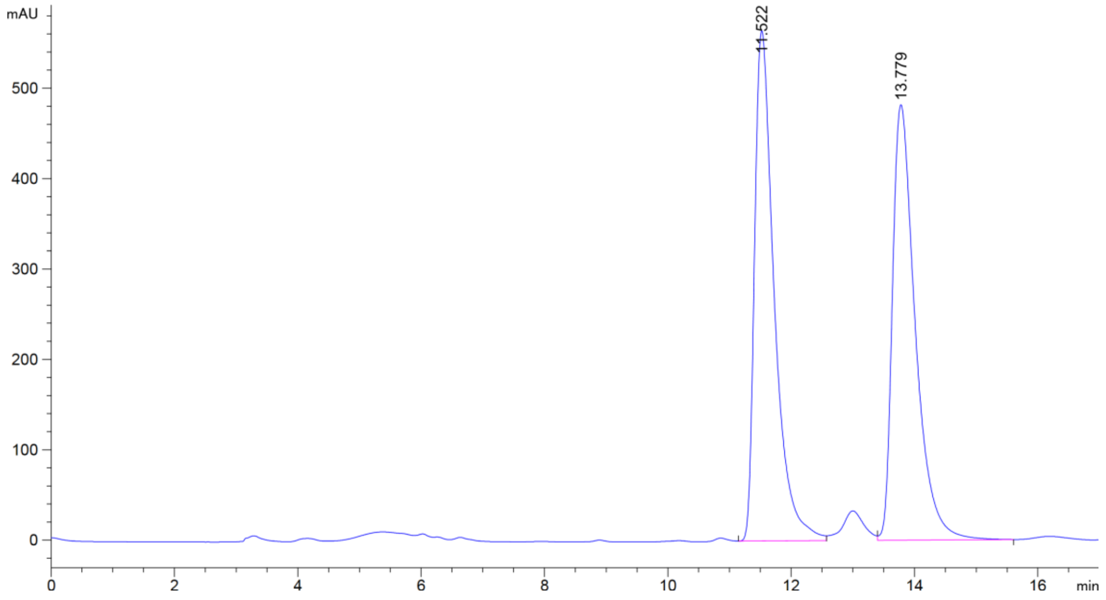

Peak #	RetTime [min]	Type	Width [min]	Area [mAU*s]	Height [mAU]	Area %
1	11.522	BV	0.3346	1.25129e4	564.57935	50.3204
2	13.779	VB	0.3892	1.23535e4	481.73816	49.6796

Enantioenriched **3zd**

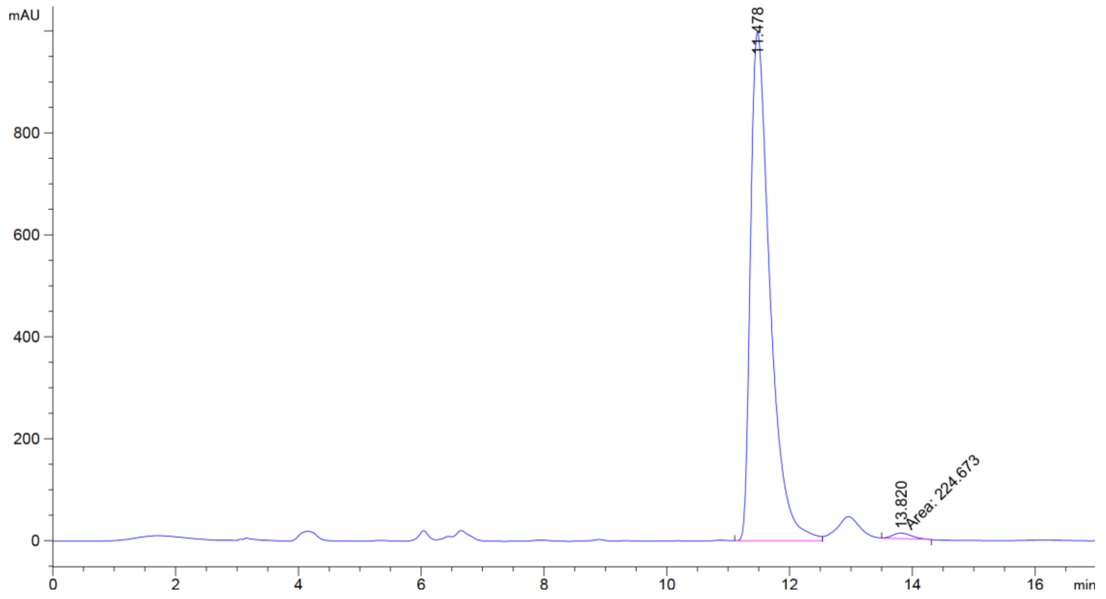

Peak #	RetTime [min]	Type	Width [min]	Area [mAU*s]	Height [mAU]	Area %
1	11.478	BV	0.3361	2.20777e4	997.83002	98.9926
2	13.820	MM	0.3466	224.67255	10.80520	1.0074

Racemic **3ze**

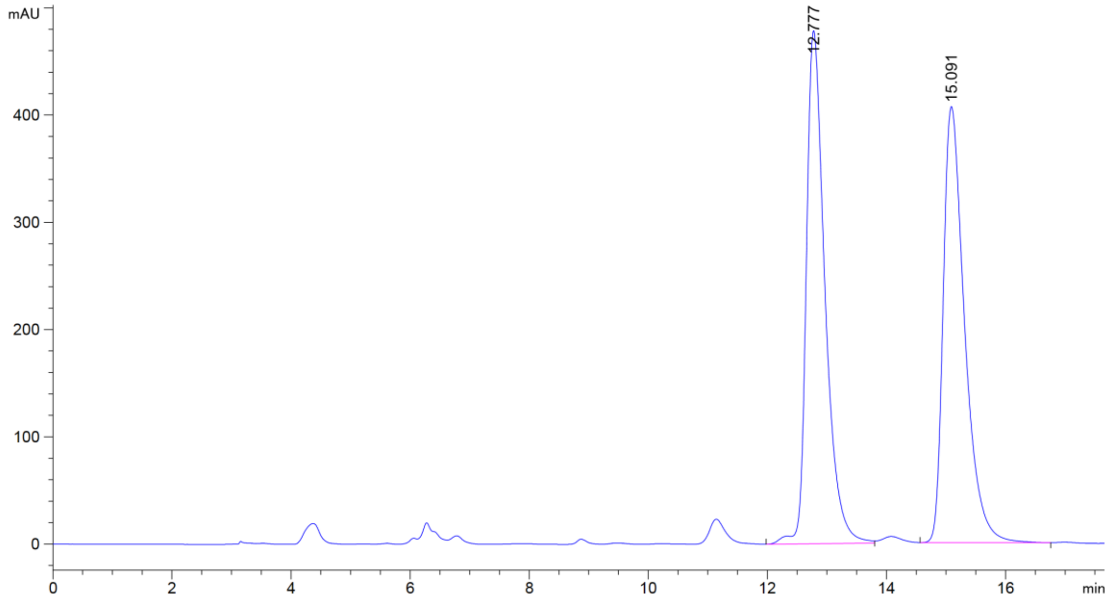

Peak #	RetTime [min]	Type	Width [min]	Area [mAU*s]	Height [mAU]	Area %
1	12.777	BV	0.3280	1.04147e4	478.34445	50.6802
2	15.091	BB	0.3789	1.01351e4	406.48047	49.3198

Enantioenriched **3ze**

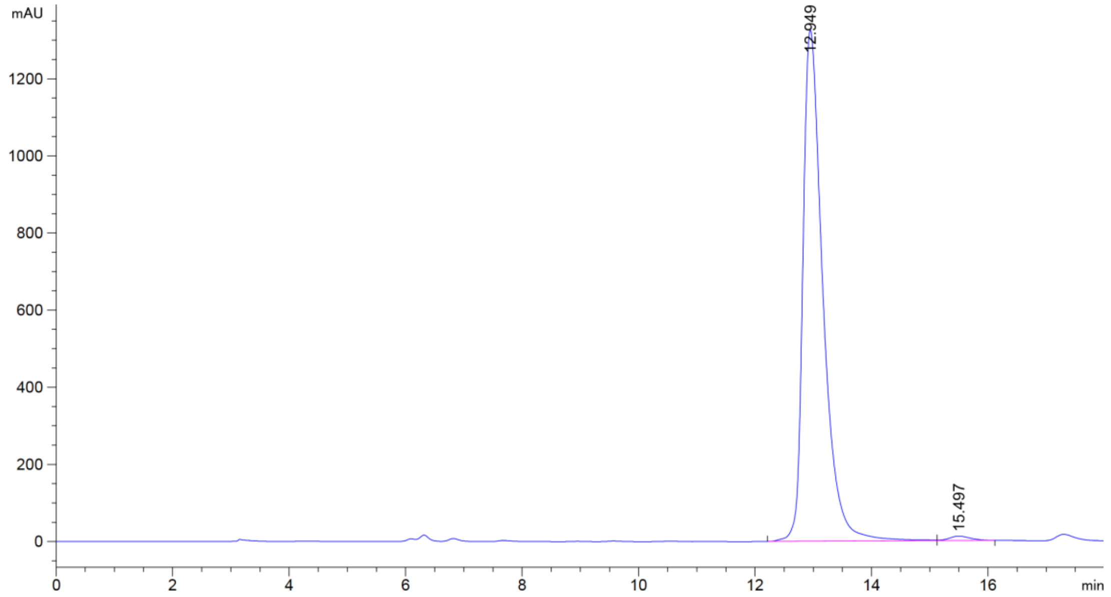

Peak #	RetTime [min]	Type	Width [min]	Area [mAU*s]	Height [mAU]	Area %
1	12.949	BV	0.3621	3.15912e4	1325.29822	99.1086
2	15.497	VB	0.3755	284.13412	11.37197	0.8914

Racemic **3zf**

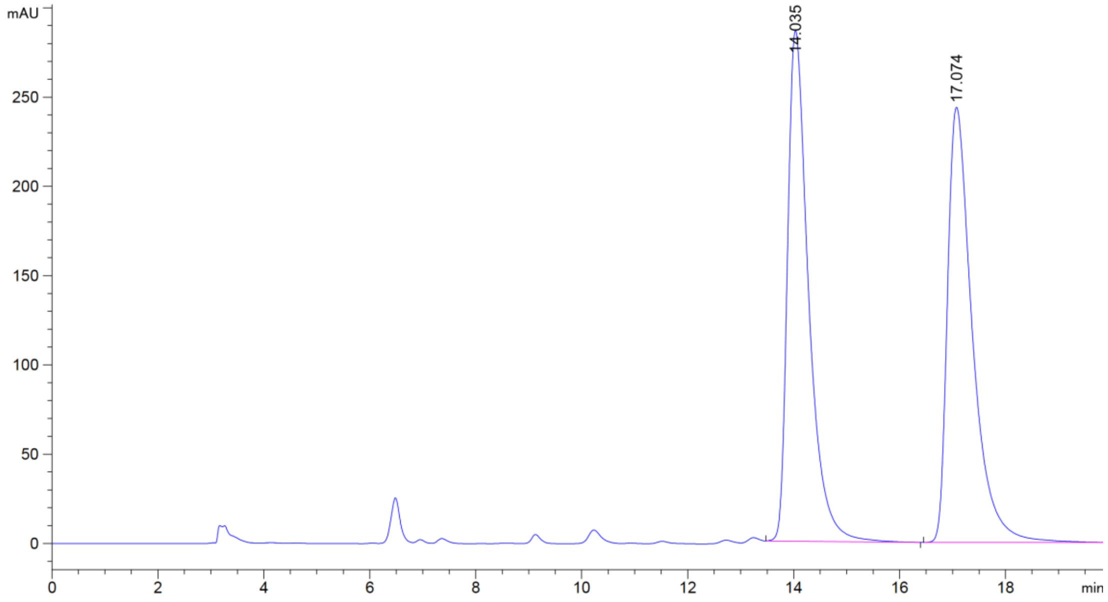

Peak #	RetTime [min]	Type	Width [min]	Area [mAU*s]	Height [mAU]	Area %
1	14.035	BB	0.4155	7884.90527	286.15787	49.8102
2	17.074	BBA	0.4923	7944.99170	243.73462	50.1898

Enantioenriched **3zf**

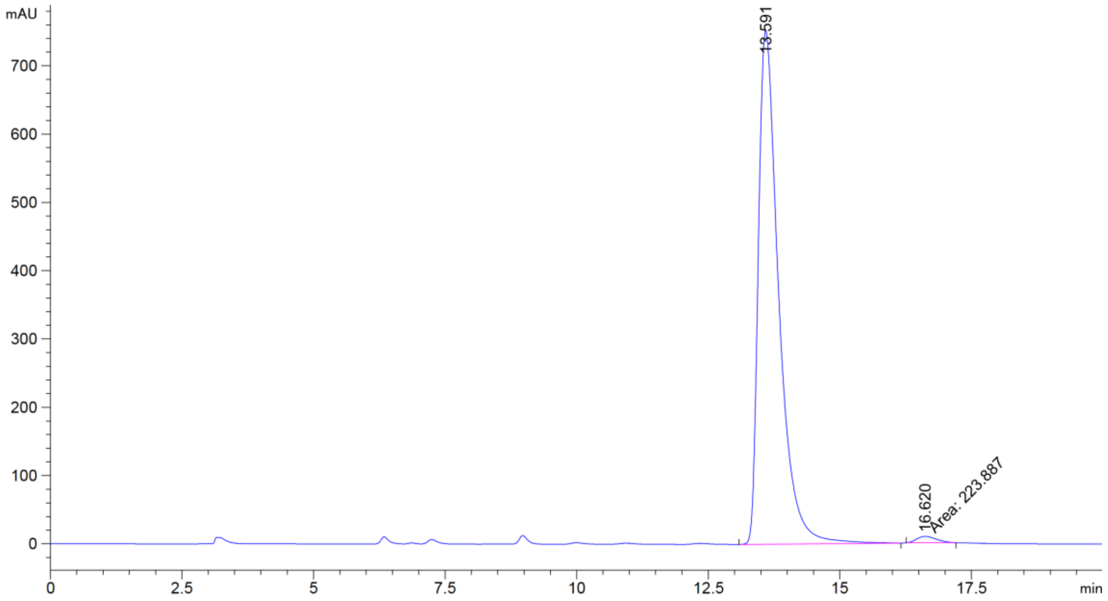

Peak #	RetTime [min]	Type	Width [min]	Area [mAU*s]	Height [mAU]	Area %
1	13.591	BB	0.4058	2.02205e4	751.83185	98.9049
2	16.620	MM	0.4135	223.88702	9.02336	1.0951

Racemic **3zg**

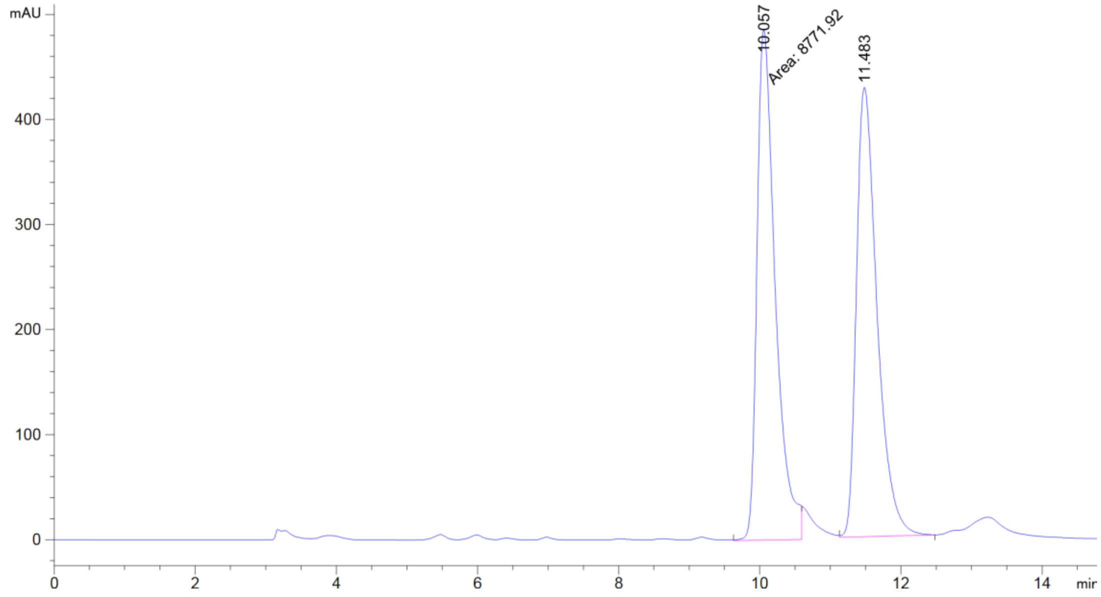

Peak #	RetTime [min]	Type	Width [min]	Area [mAU*s]	Height [mAU]	Area %
1	10.057	MM	0.3007	8771.91602	486.21970	50.2329
2	11.483	VB	0.3090	8690.58691	427.81180	49.7671

Enantioenriched **3zg**

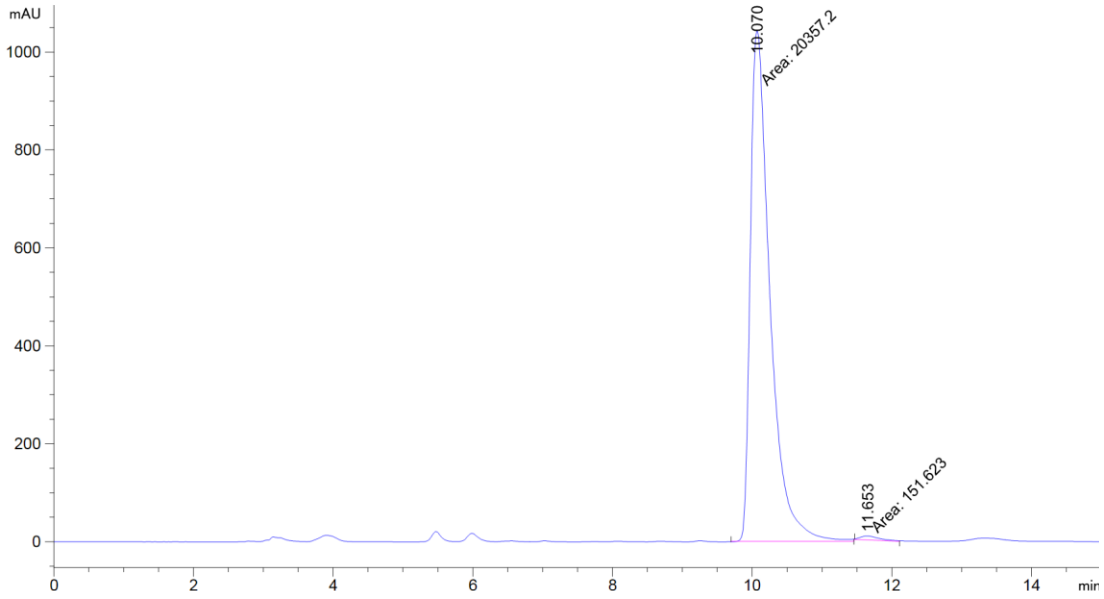

Peak #	RetTime [min]	Type	Width [min]	Area [mAU*s]	Height [mAU]	Area %
1	10.070	MM	0.3252	2.03572e4	1043.37854	99.2607
2	11.653	MM	0.3202	151.62303	7.89260	0.7393

Racemic **3zh**

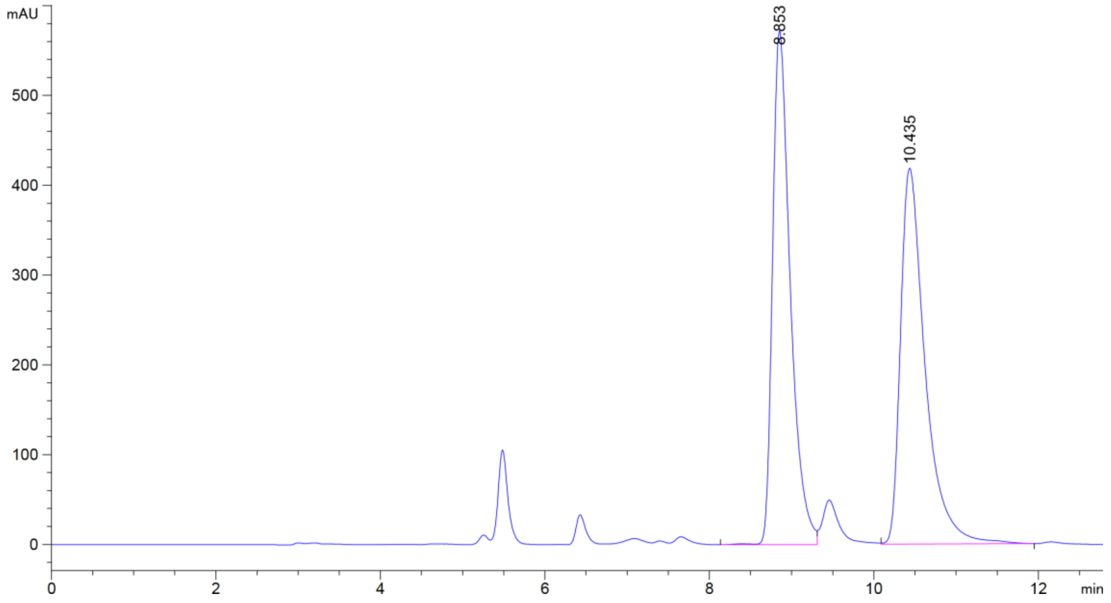

Peak #	RetTime [min]	Type	Width [min]	Area [mAU*s]	Height [mAU]	Area %
1	8.853	BV	0.2247	8571.79785	572.29114	49.6133
2	10.435	VB	0.3106	8705.40527	418.58215	50.3867

Enantioenriched **3zh**

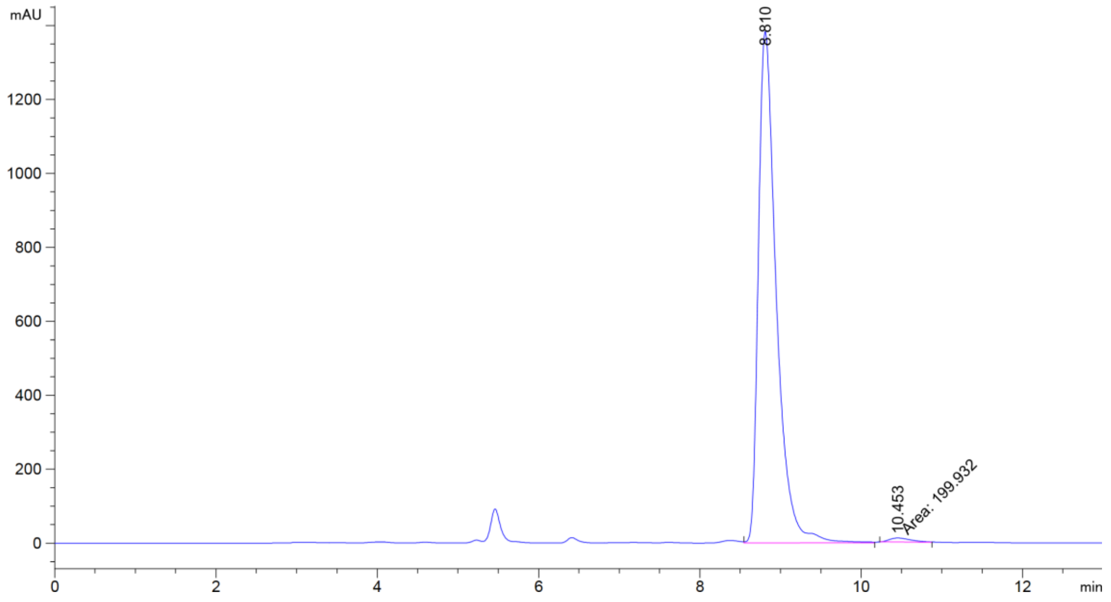

Peak #	RetTime [min]	Type	Width [min]	Area [mAU*s]	Height [mAU]	Area %
1	8.810	VV	0.2321	2.13702e4	1384.04224	99.0731
2	10.453	MM	0.3087	199.93233	10.79396	0.9269

Racemic **3zi**

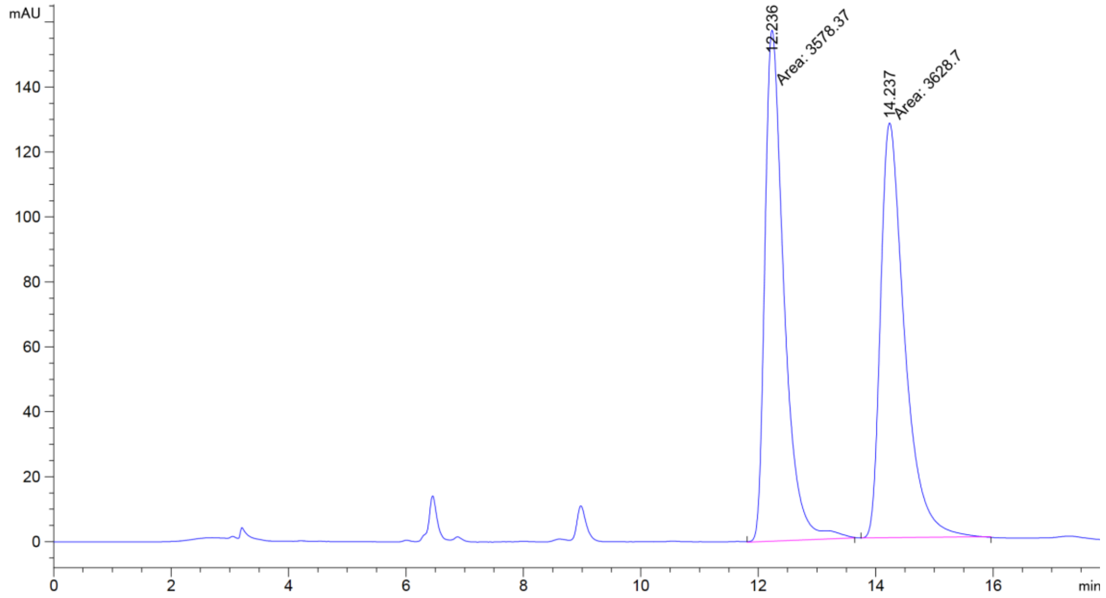

Peak #	RetTime [min]	Type	Width [min]	Area [mAU*s]	Height [mAU]	Area %
1	12.236	MM	0.3789	3578.36572	157.39326	49.6508
2	14.237	MM	0.4735	3628.70068	127.71703	50.3492

Enantioenriched **3zi**

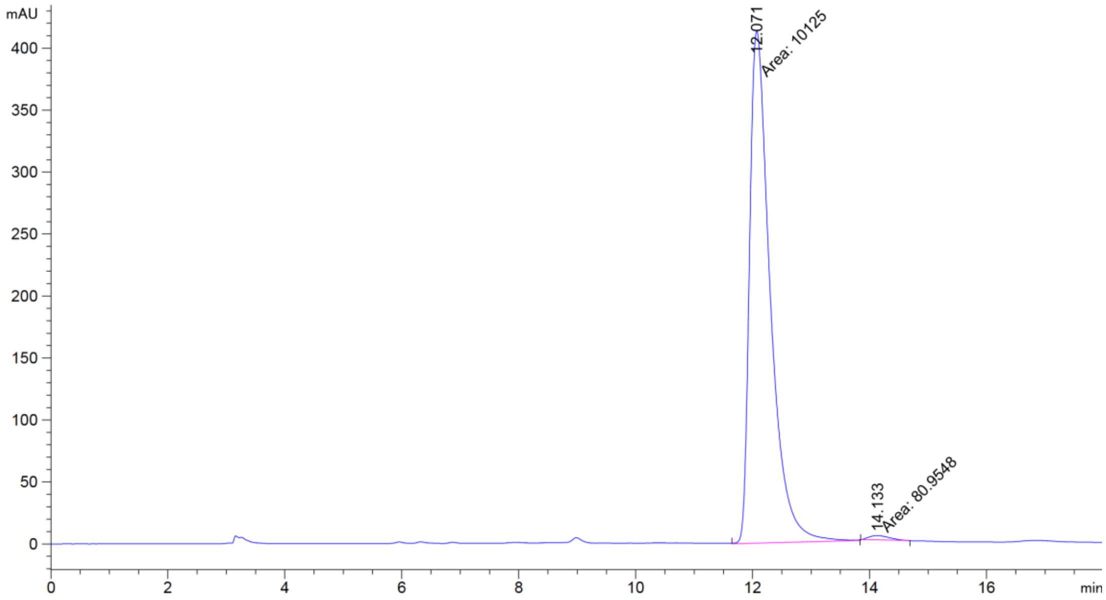

Peak #	RetTime [min]	Type	Width [min]	Area [mAU*s]	Height [mAU]	Area %
1	12.071	MM	0.4082	1.01250e4	413.39389	99.2068
2	14.133	MM	0.3946	80.95477	3.41939	0.7932

Racemic **6**

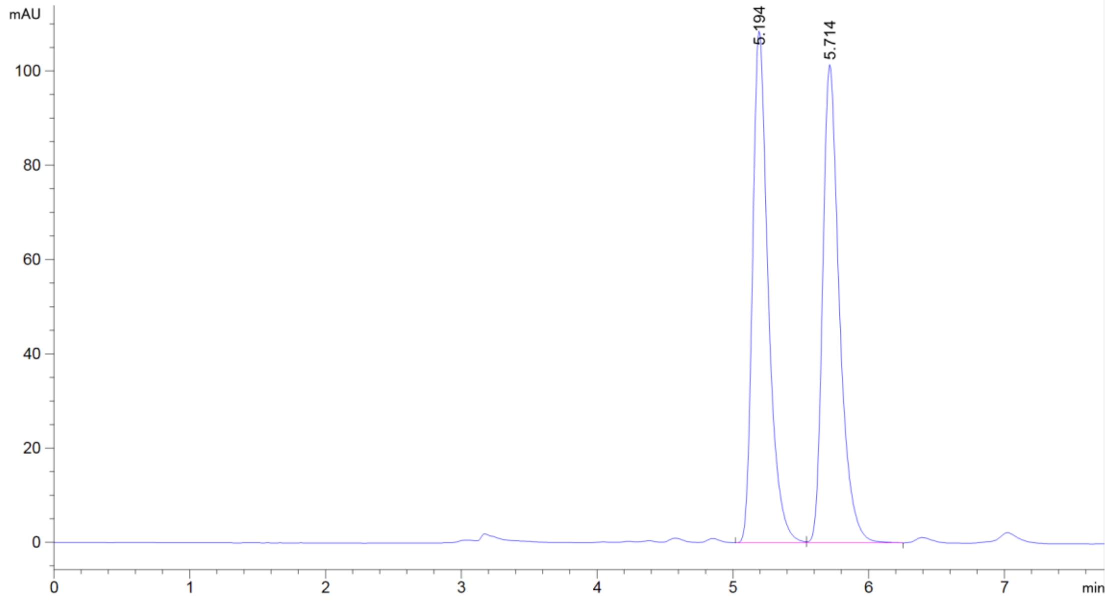

Peak #	RetTime [min]	Type	Width [min]	Area [mAU*s]	Height [mAU]	Area %
1	5.194	BV	0.1196	871.10480	108.59711	50.0150
2	5.714	VB	0.1301	870.58331	101.50835	49.9850

Enantioenriched **6**

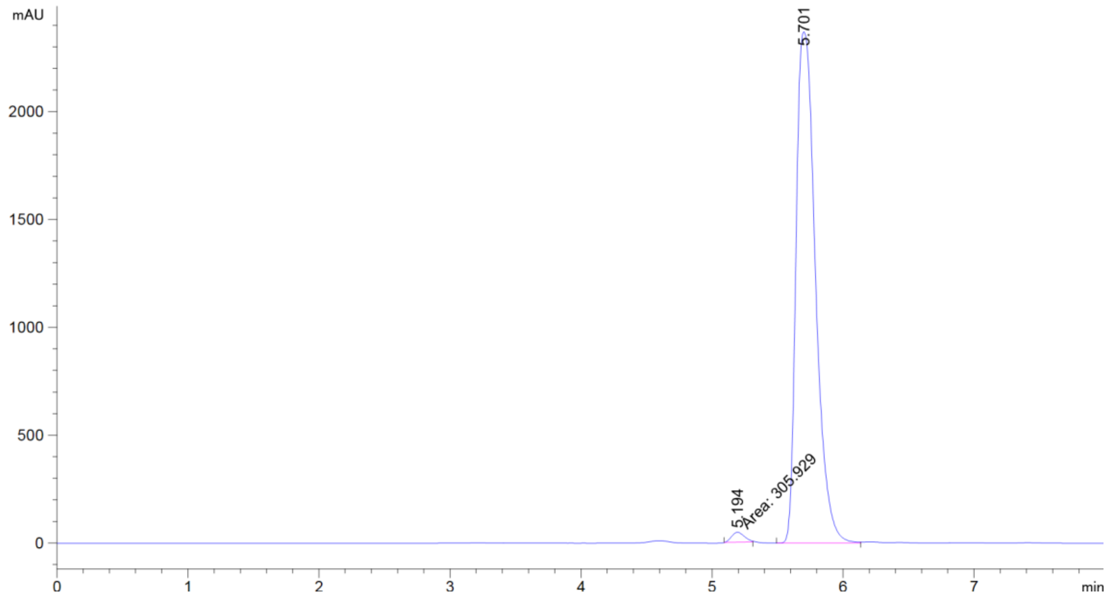

Peak #	RetTime [min]	Type	Width [min]	Area [mAU*s]	Height [mAU]	Area %
1	5.194	MM	0.1120	305.92899	45.53996	1.2448
2	5.701	BV	0.1614	2.42697e4	2371.00049	98.7552

Racemic **7**

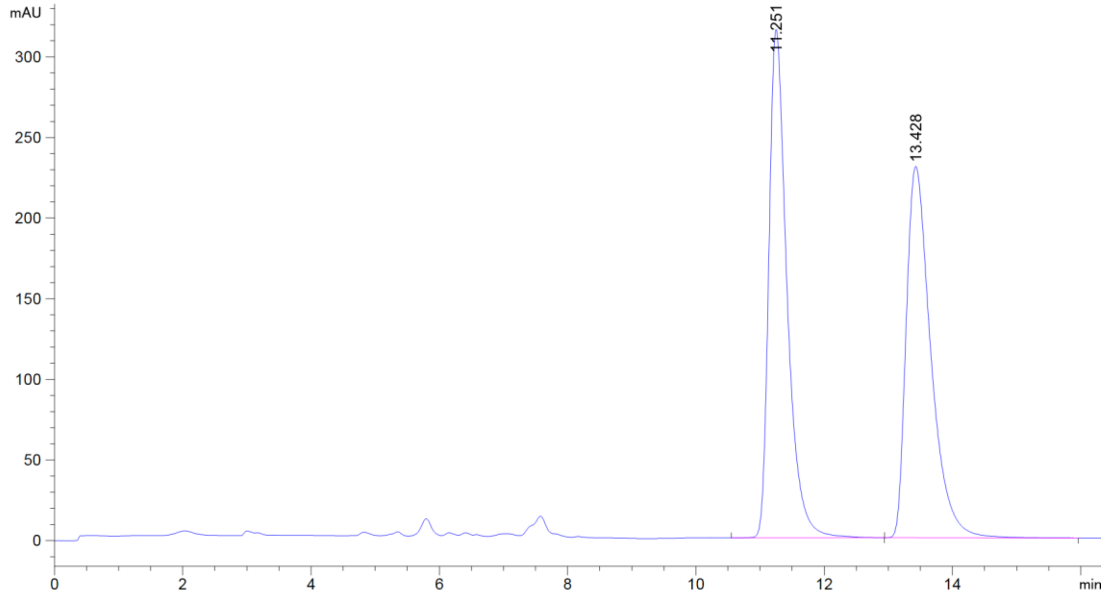

Peak #	RetTime [min]	Type	Width [min]	Area [mAU*s]	Height [mAU]	Area %
1	11.251	BB	0.2971	6192.31396	315.34213	50.2064
2	13.428	BB	0.4070	6141.39014	230.36980	49.7936

Enantioenriched **7**

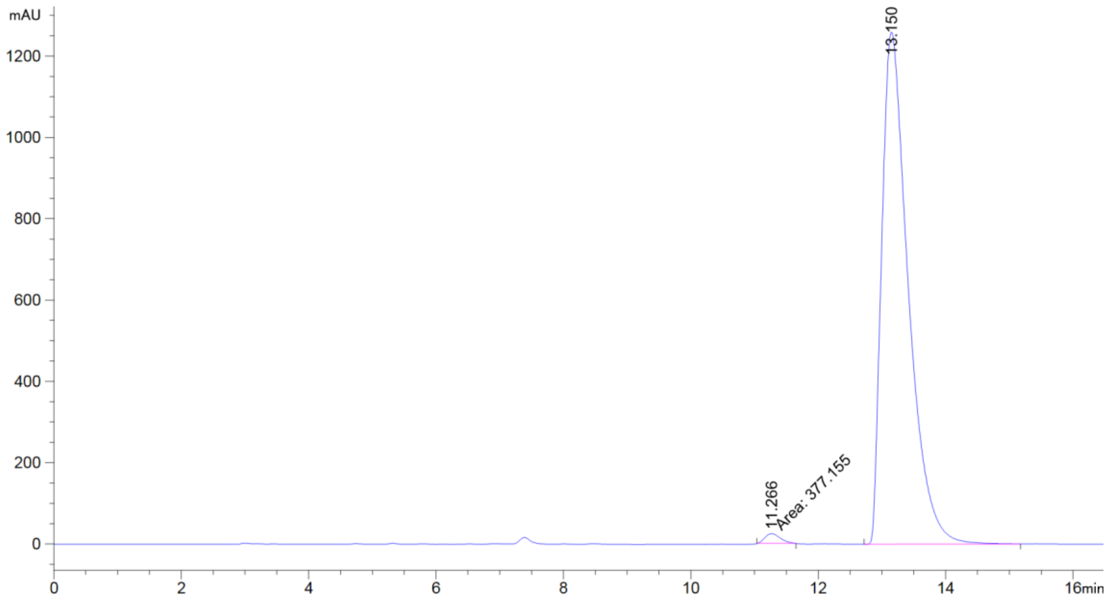

Peak #	RetTime [min]	Type	Width [min]	Area [mAU*s]	Height [mAU]	Area %
1	11.266	MM	0.2687	377.15509	23.39069	1.0278
2	13.150	BB	0.4463	3.63175e4	1259.72913	98.9722

Racemic **8**

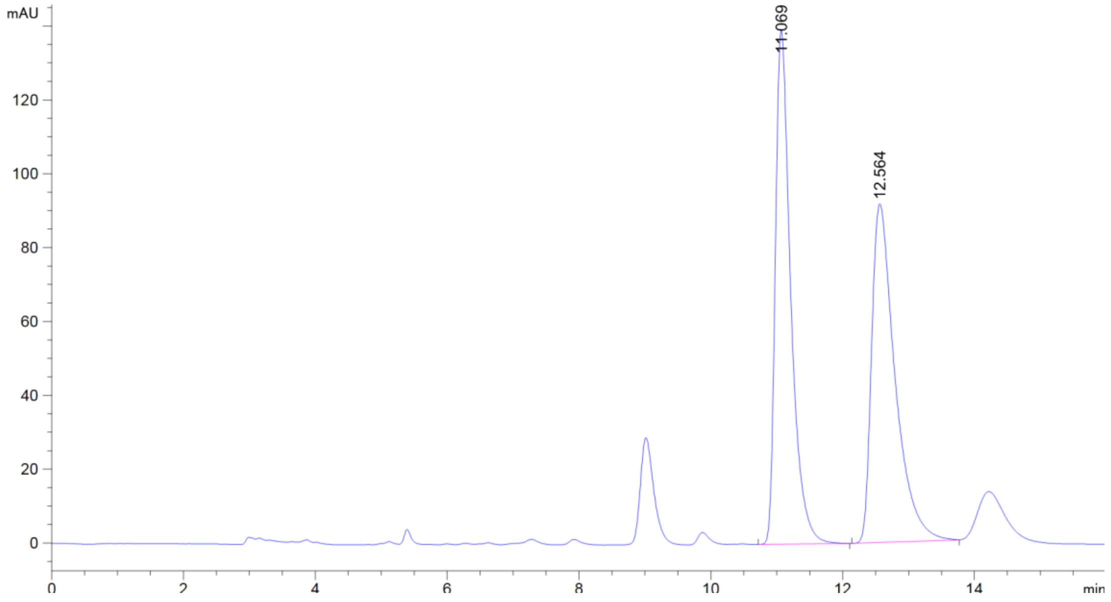

Peak #	RetTime [min]	Type	Width [min]	Area [mAU*s]	Height [mAU]	Area %
1	11.069	BB	0.2454	2259.97803	139.20186	50.0374
2	12.564	BB	0.3672	2256.59863	91.67870	49.9626

Enantioenriched **8**

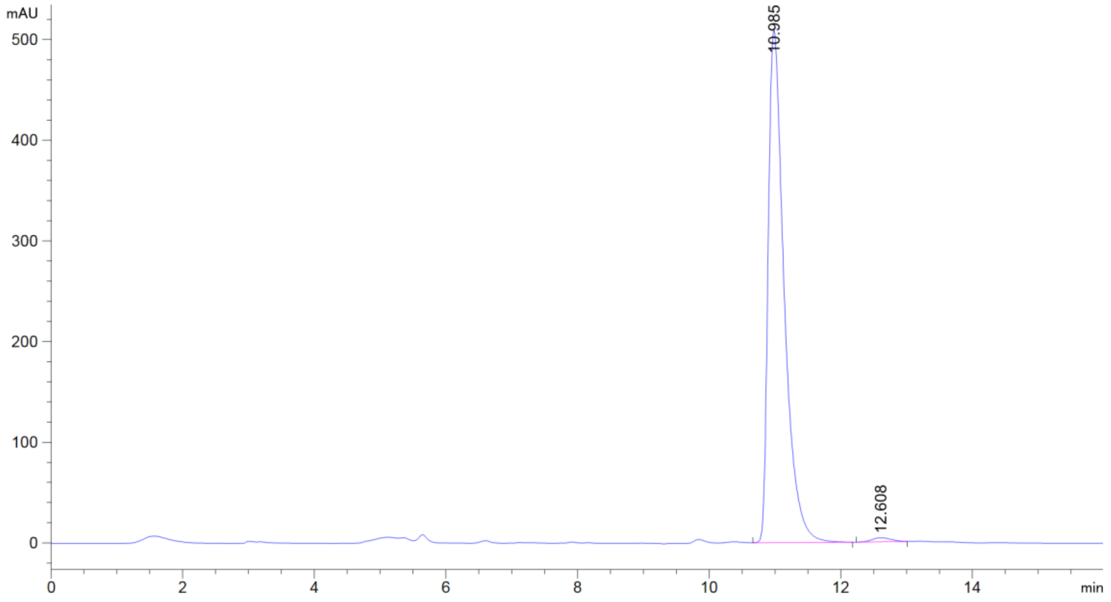

Peak #	RetTime [min]	Type	Width [min]	Area [mAU*s]	Height [mAU]	Area %
1	10.985	BB	0.2547	8583.42773	509.07983	99.0443
2	12.608	BB	0.3039	82.82517	4.09568	0.9557

Racemic **9**

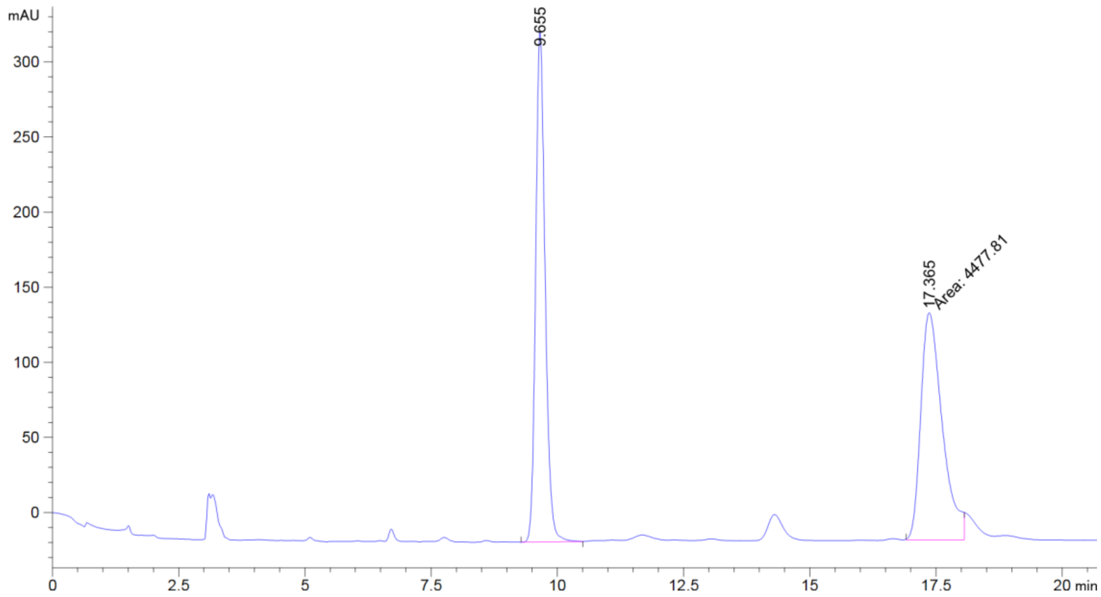

Peak #	RetTime [min]	Type	Width [min]	Area [mAU*s]	Height [mAU]	Area %
1	9.655	BB	0.2004	4433.96436	339.38297	49.7540
2	17.365	MM	0.4939	4477.81006	151.09077	50.2460

Enantioenriched **9**

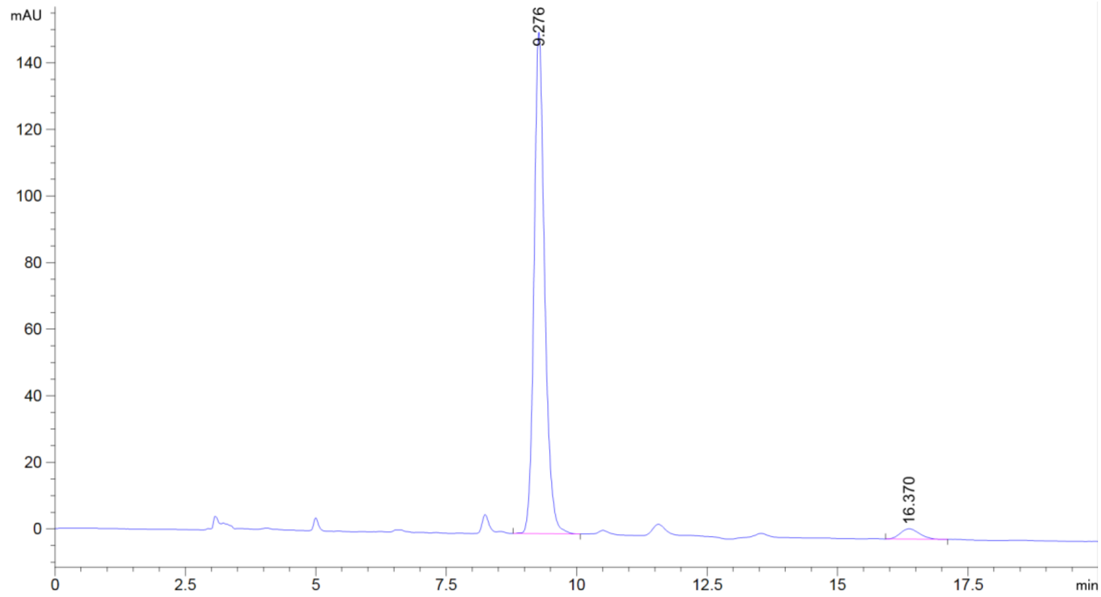

Peak #	RetTime [min]	Type	Width [min]	Area [mAU*s]	Height [mAU]	Area %
1	9.276	BB	0.2131	2133.57861	150.75815	96.4348
2	16.370	BB	0.3731	78.87936	3.11805	3.5652

Racemic **10**

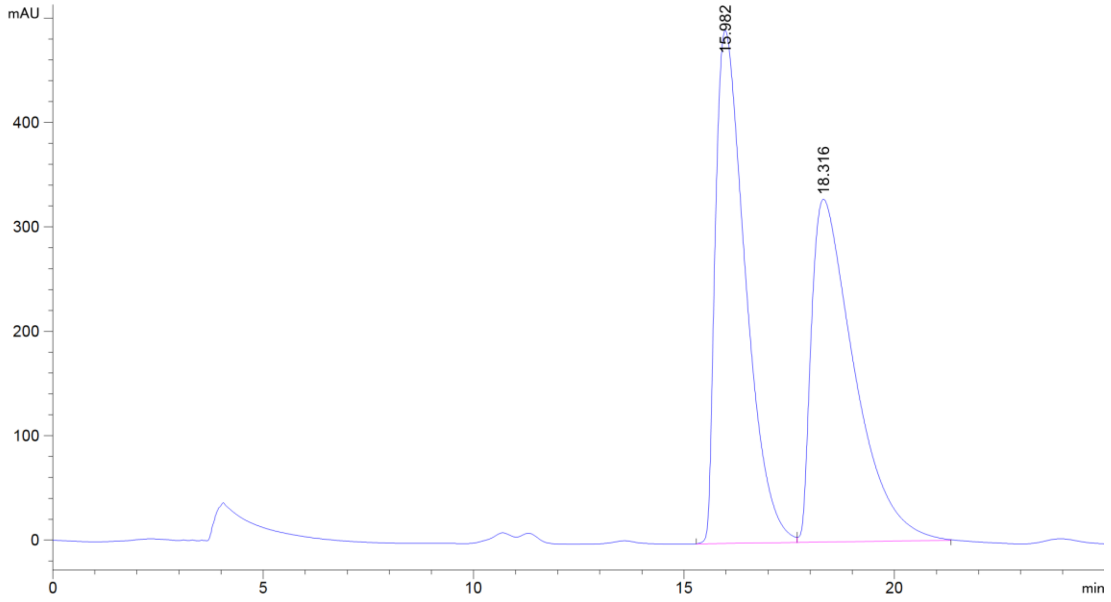

Peak #	RetTime [min]	Type	Width [min]	Area [mAU*s]	Height [mAU]	Area %
1	15.982	BV	0.7702	2.43650e4	491.62036	51.1711
2	18.316	VB	1.0598	2.32498e4	328.48212	48.8289

Enantioenriched **10**

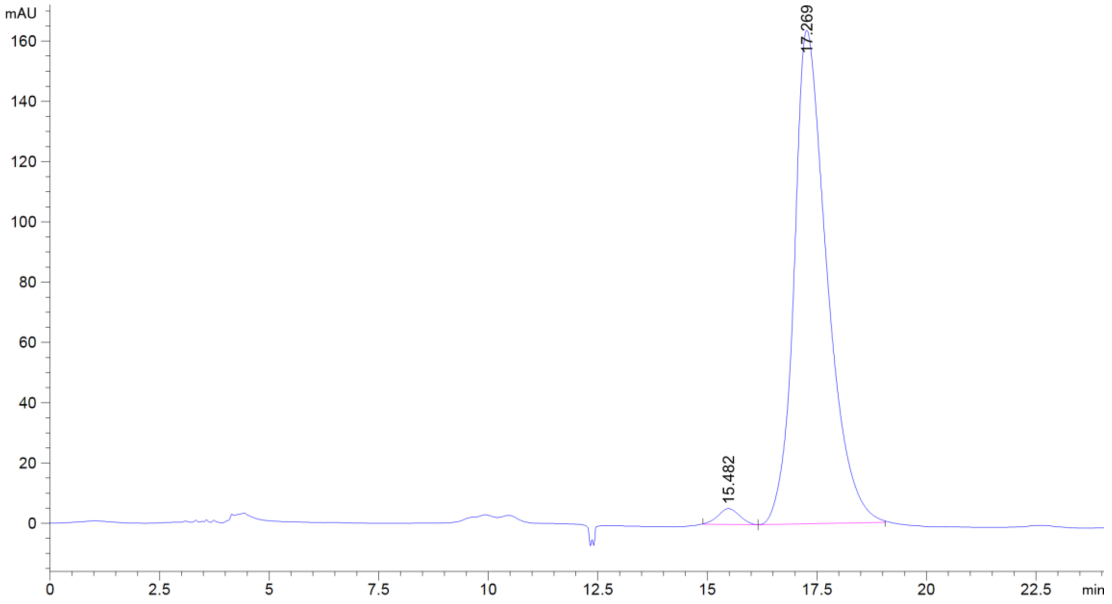

Peak #	RetTime [min]	Type	Width [min]	Area [mAU*s]	Height [mAU]	Area %
1	15.482	BV	0.4972	179.06532	5.25974	2.0485
2	17.269	VB	0.7780	8562.08496	163.76654	97.9515

Racemic **11**

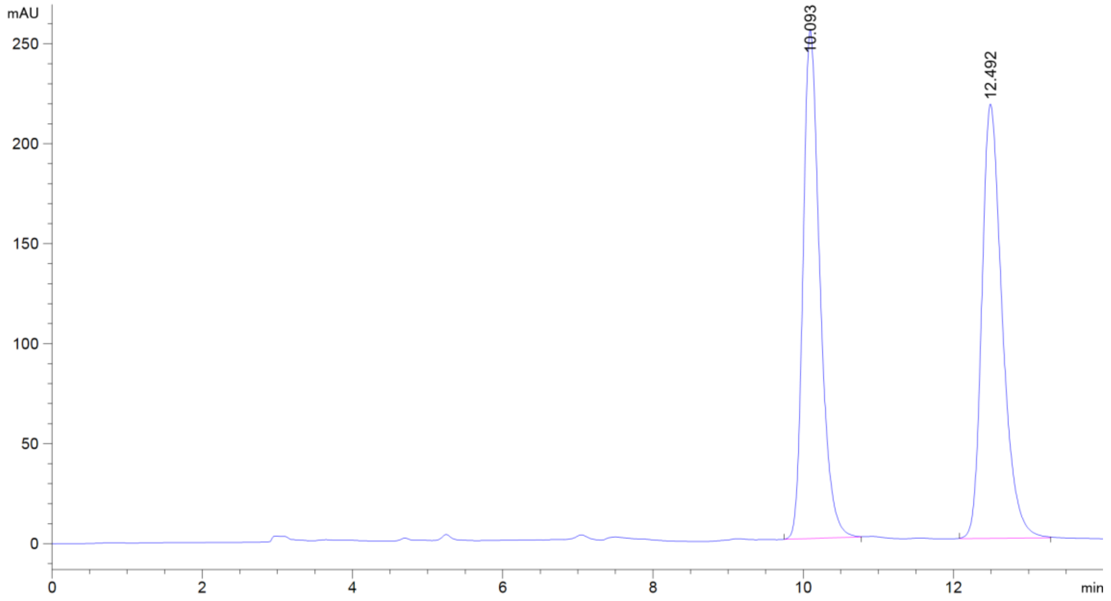

Peak #	RetTime [min]	Type	Width [min]	Area [mAU*s]	Height [mAU]	Area %
1	10.093	BB	0.2360	3964.84790	254.20882	49.0993
2	12.492	BB	0.2867	4110.31348	217.30687	50.9007

Enantioenriched **11**

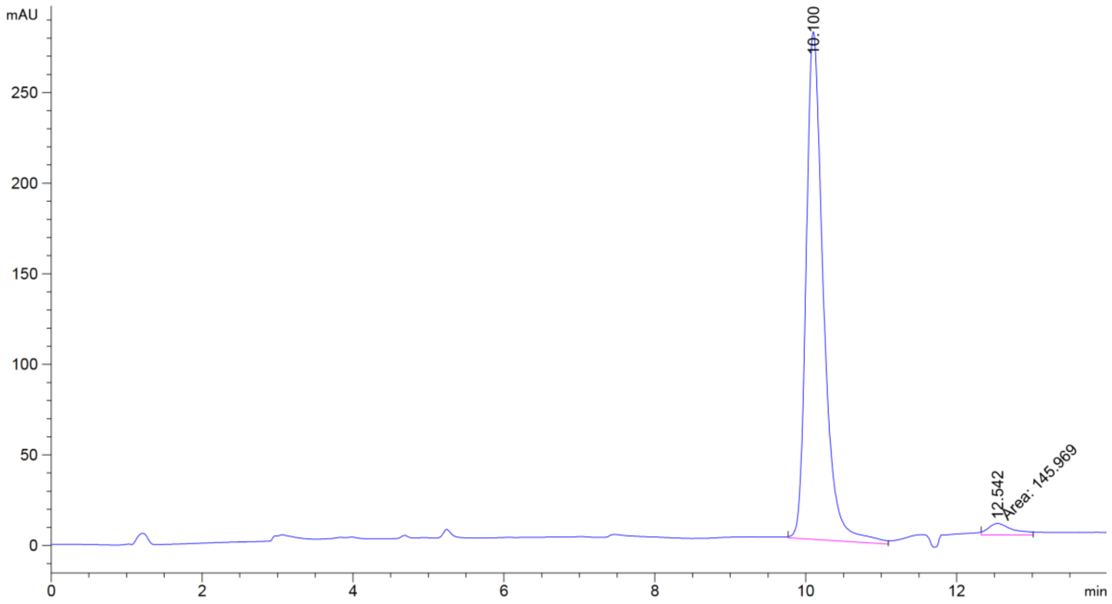

Peak #	RetTime [min]	Type	Width [min]	Area [mAU*s]	Height [mAU]	Area %
1	10.100	BB	0.2399	4463.46875	280.16452	96.8333
2	12.542	MM	0.3871	145.96904	6.28408	3.1667

Racemic **12**

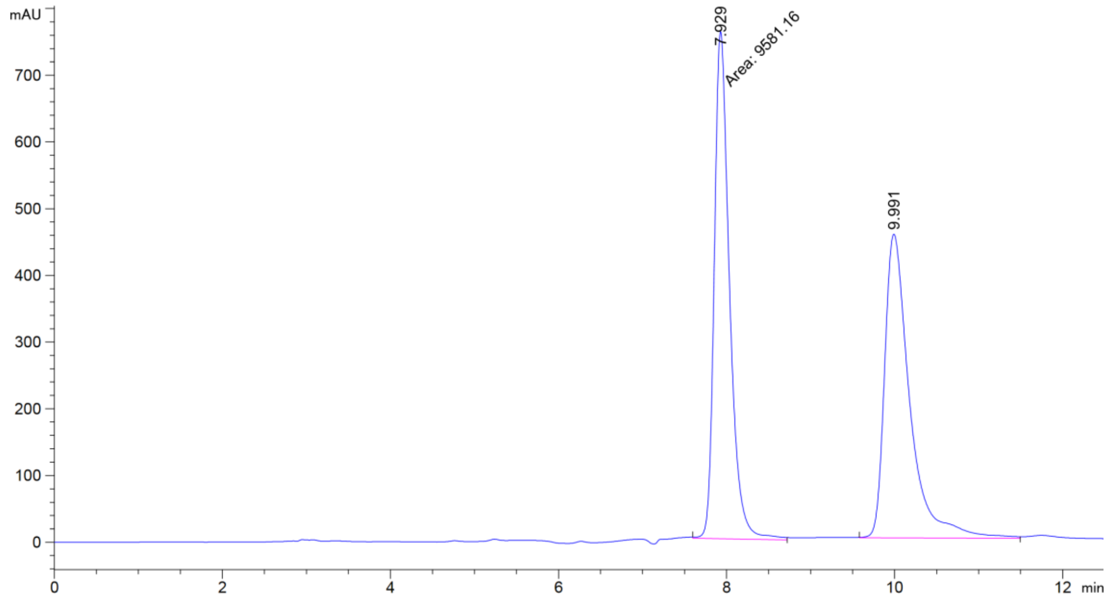

Peak #	RetTime [min]	Type	Width [min]	Area [mAU*s]	Height [mAU]	Area %
1	7.929	MM	0.2098	9581.16406	761.09845	50.3355
2	9.991	BB	0.3083	9453.44043	455.22180	49.6645

Enantioenriched **12**

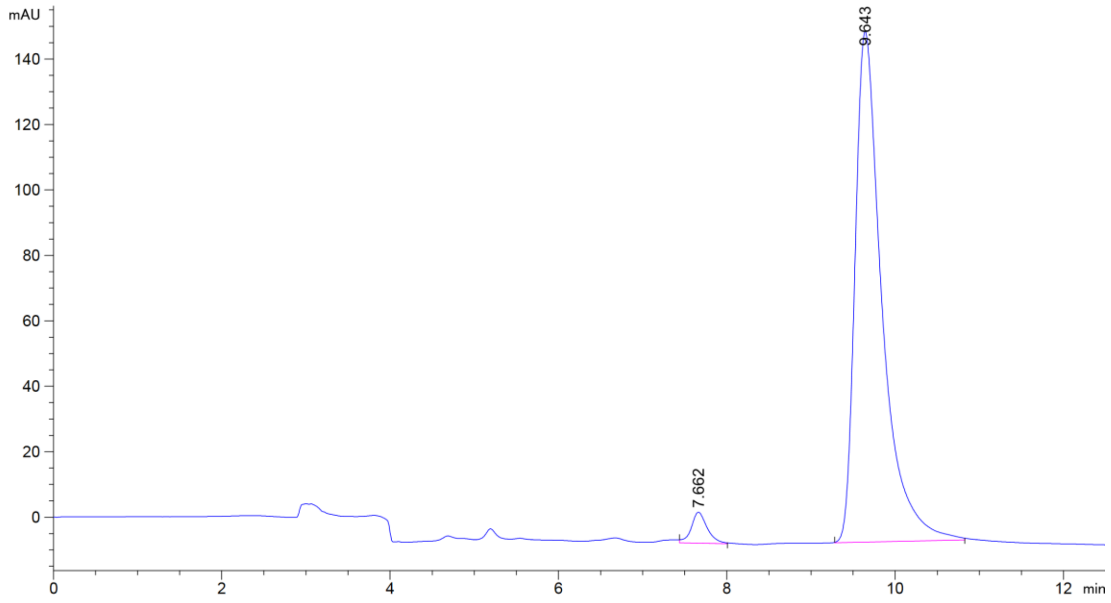

Peak #	RetTime [min]	Type	Width [min]	Area [mAU*s]	Height [mAU]	Area %
1	7.662	BB	0.1936	124.15815	9.43089	3.4579
2	9.643	BB	0.3312	3466.36304	156.02187	96.5421

Racemic **13**

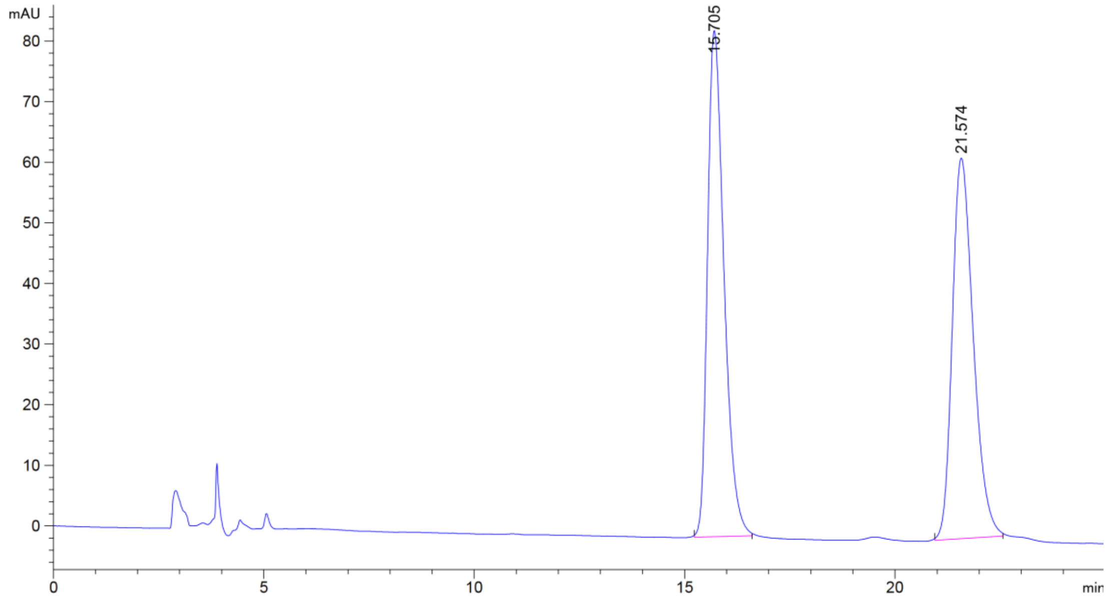

Peak #	RetTime [min]	Type	Width [min]	Area [mAU*s]	Height [mAU]	Area %
1	15.705	BB	0.4170	2268.35254	83.49072	51.0137
2	21.574	BB	0.5367	2178.20728	62.78817	48.9863

Enantioenriched **13**

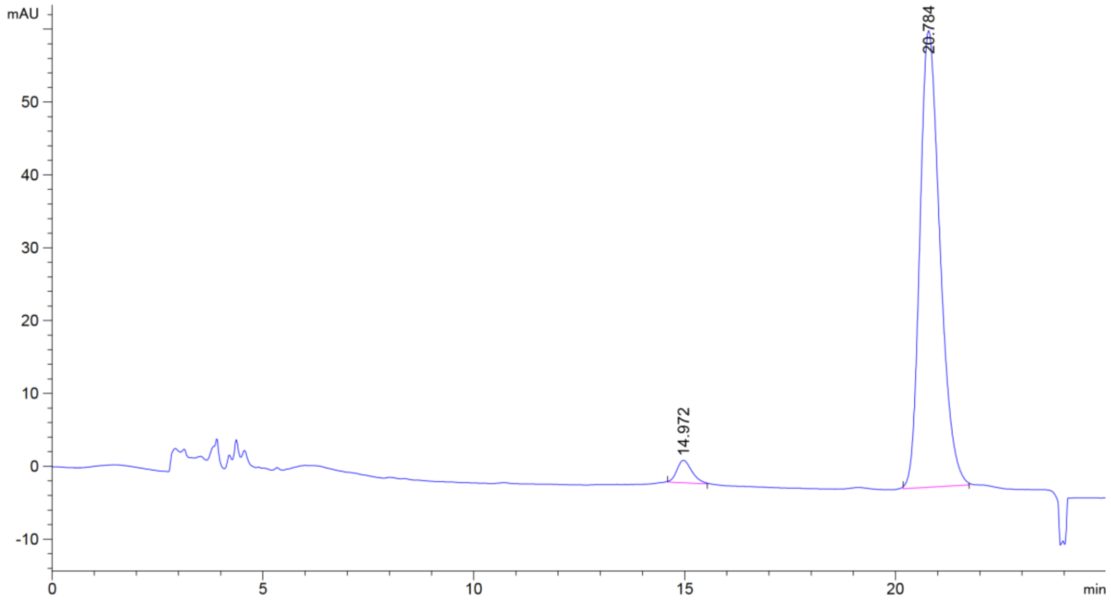

Peak #	RetTime [min]	Type	Width [min]	Area [mAU*s]	Height [mAU]	Area %
1	14.972	BB	0.3735	76.28110	3.07364	3.5441
2	20.784	BB	0.5064	2076.04346	62.67709	96.4559

9. References

(1) Plachinski, E. F.; Qian, R. Z.; Villanueva, R.; Poole, D. L.; Rosenthal, T.; Yoon, T. P. Enantioselective [2π + 2σ] Photocycloaddition Enabled by Brønsted Acid Catalyzed Chromophore Activation. *J. Am. Chem. Soc.*, **146**, 31400–31404 (2024).

(2) Li, T.; Wang, Y.; Xu, Y.; Ren, H.; Lin, Z.; Li, Z.; Zheng, J. Zwitterionic π-Allyl-Pd Species Enabled [2σ + 2π] Cycloaddition Reactions of Vinylbicyclo[1.1.0]Butanes (VBCBs) with Alkenes, Carbonyls, and Imines. *ACS Catal.*, **14**, 18799–18809 (2024).

(3) Qin, T.; Zi, W. Palladium-Catalyzed Enantioselective [2σ + 2π] Cycloadditions of Vinyl-Carbonyl-Bicyclo[1.1.0]Butanes with Arylidenemalononitriles. *Chinese Chemical Letters*, 111072 (2025).

(4) Shen, W.-J.; Zou, X.-X.; Li, M.; Cheng, Y.-Z.; You, S.-L. Enantioselective Dearomative [2π + 2σ] Photocycloaddition of Naphthalene Derivatives with Bicyclo[1.1.0]Butanes Enabled by Gd(III) Catalysis. *J. Am. Chem. Soc.*, **147**, 11667–11674 (2025).

(5) Meisner, J. S.; Sedbrook, D. F.; Krikorian, M.; Chen, J.; Sattler, A.; Carnes, M. E.; Murray, C. B.; Steigerwald, M.; Nuckolls, C. Functionalizing Molecular Wires: A Tunable Class of α,ω-Diphenyl-μ,ν-Dicyano-Oligoenes. *Chem. Sci.*, **3**, 1007–1014 (2012).

(6) Guo, R.; Chang, Y.-C.; Herter, L.; Salome, C.; Braley, S. E.; Fessard, T. C.; Brown, M. K. Strain-Release [2π + 2σ] Cycloadditions for the Synthesis of Bicyclo[2.1.1]Hexanes Initiated by Energy Transfer. *J. Am. Chem. Soc.*, **144**, 7988–7994 (2022).

(7) Liang, Y.; Paulus, F.; Daniliuc, C. G.; Glorius, F. Catalytic Formal [2π + 2σ] Cycloaddition of Aldehydes with Bicyclobutanes: Expedient Access to Polysubstituted 2-Oxabicyclo[2.1.1]Hexanes. *Angewandte Chemie International Edition*, **62**, e202305043 (2023).

(8) Fu, Q.; Cao, S.; Wang, J.; Lv, X.; Wang, H.; Zhao, X.; Jiang, Z. Enantioselective [2π + 2σ] Cycloadditions of Bicyclo[1.1.0]Butanes with Vinylazaarenes through Asymmetric Photoredox Catalysis. *J. Am. Chem. Soc.*, **146**, 8372–8380 (2024).

(9) Zhao, Y.; Truhlar, D. G., Density Functionals with Broad Applicability in Chemistry. *Accounts of Chemical Research*, **41**, 157-167 (2008).

(10) Zhao, Y.; Truhlar, D. G., The M06 suite of density functionals for main group thermochemistry, thermochemical kinetics, noncovalent interactions, excited states, and transition elements: two new functionals and systematic testing of four M06-class functionals and 12 other functionals. *Theoretical Chemistry Accounts*, **120**, 215-241 (2008).

(11) Grimme, S.; Antony, J.; Ehrlich, S.; Krieg, H.; A consistent and accurate ab initio parametrization of density functional dispersion correction (DFT-D) for the 94 elements H-Pu. *Journal of Chemical Physics*., **132**, 154104 (2010).

(12) Frisch, M. J.; Trucks, G. W.; Schlegel, H. B.; Scuseria, G. E.; Robb, M. A.; Cheeseman, J. R.; Scalmani, G.; Barone, V.; Petersson, G. A.; Nakatsuji, H.; Li, X.; Caricato, M.; Marenich, A. V.; Bloino, J.; Janesko, B. G.; Gomperts, R.; Mennucci, B.; Hratchian, H. P.; Ortiz, J. V.; Izmaylov, A. F.; Sonnenberg, J. L.; Williams; Ding, F.; Lipparini, F.; Egidi, F.; Goings, J.; Peng, B.; Petrone, A.; Henderson, T.; Ranasinghe, D.; Zakrzewski, V. G.; Gao, J.; Rega, N.; Zheng, G.; Liang, W.; Hada, M.; Ehara, M.; Toyota, K.; Fukuda, R.; Hasegawa, J.; Ishida, M.; Nakajima, T.; Honda, Y.; Kitao, O.; Nakai, H.; Vreven, T.; Throssell, K.; Montgomery Jr., J. A.; Peralta, J. E.; Ogliaro, F.; Bearpark, M. J.; Heyd, J. J.; Brothers, E. N.; Kudin, K. N.; Staroverov, V. N.; Keith, T. A.; Kobayashi, R.; Normand, J.; Raghavachari, K.; Rendell, A. P.; Burant, J. C.; Iyengar, S. S.; Tomasi, J.; Cossi, M.; Millam, J. M.; Klene, M.; Adamo, C.; Cammi, R.; Ochterski, J. W.; Martin, R. L.; Morokuma, K.; Farkas, O.; Foresman, J. B.; Fox, D. J. Gaussian 16 Rev. C.01: Wallingford, CT, 2016.

(13) Clark, T.; Chandrasekhar, J.; Spitznagel, G. W.; Schleyer, P. V. R.; Efficient diffuse function-augmented basis sets for anion calculations. III. The 3-21+G basis set for first-row elements, Li–F. *Journal of Computational Chemistry*, **4**, 294-301 (1983).

(14) Dolg, M.; Wedig, U.; Stoll, H.; Preuss, H.; Energy-adjusted ab initio pseudopotentials for the first row transition elements. *The Journal of Chemical Physics*, **86**, 866-872 (1987).

(15) Tomasi, J.; Mennucci, B.; Cammi, R.; Quantum Mechanical Continuum Solvation Models. *Chemical Reviews*, **105**, 2999-3094 (2005).
